# A Model-Based Approach for Identifying Signatures of Ancient Balancing Selection in Genetic Data

**DOI:** 10.1371/journal.pgen.1004561

**Published:** 2014-08-21

**Authors:** Michael DeGiorgio, Kirk E. Lohmueller, Rasmus Nielsen

**Affiliations:** 1 Department of Biology, Pennsylvania State University, University Park, Pennsylvania, United States of America; 2 Department of Ecology and Evolutionary Biology, University of California, Los Angeles, Los Angeles, California, United States of America; 3 Department of Integrative Biology, University of California, Berkeley, Berkeley, California, United States of America; 4 Department of Statistics, University of California, Berkeley, Berkeley, California, United States of America; 5 Department of Biology, University of Copenhagen, Copenhagen, Denmark; University of Washington, United States of America

## Abstract

While much effort has focused on detecting positive and negative directional selection in the human genome, relatively little work has been devoted to balancing selection. This lack of attention is likely due to the paucity of sophisticated methods for identifying sites under balancing selection. Here we develop two composite likelihood ratio tests for detecting balancing selection. Using simulations, we show that these methods outperform competing methods under a variety of assumptions and demographic models. We apply the new methods to whole-genome human data, and find a number of previously-identified loci with strong evidence of balancing selection, including several HLA genes. Additionally, we find evidence for many novel candidates, the strongest of which is *FANK1*, an imprinted gene that suppresses apoptosis, is expressed during meiosis in males, and displays marginal signs of segregation distortion. We hypothesize that balancing selection acts on this locus to stabilize the segregation distortion and negative fitness effects of the distorter allele. Thus, our methods are able to reproduce many previously-hypothesized signals of balancing selection, as well as discover novel interesting candidates.

## Author Summary

In the past, balancing selection was a topic of great theoretical interest that received much attention. However, there has been little focus toward developing methods to identify regions of the genome that are under balancing selection. In this article, we present the first set of likelihood-based methods that explicitly model the spatial distribution of polymorphism expected near a site under long-term balancing selection. Simulation results show that our methods outperform commonly-used summary statistics for identifying regions under balancing selection. Finally, we performed a scan for balancing selection in Africans and Europeans using our new methods and identified a gene called *FANK1* as our top candidate outside the HLA region. We hypothesize that the maintenance of polymorphism at *FANK1* is the result of segregation distortion.

## Introduction

Balancing selection maintains variation within a population. Multiple processes can lead to balancing selection. In overdominance, the heterozygous genotype has higher fitness than either of the homozygous genotypes [1], [2]. In frequency-dependent balancing selection, the fitness of an allele is inversely related to its frequency in the population [2], [3]. In a fluctuating or spatially-structured environment, balancing selection can occur when different alleles are favored in different environments over time or geography [2], [4], [5]. Finally, balancing selection can also be a product of opposite directed effects of segregation distortion balanced by negative selection against the distorter [6]. That is, segregation distortion leads to one allele increasing in frequency. However, if that allele is deleterious, then it is reduced in frequency by negative selection. The combined effect of these opposing forces can lead to a balanced polymorphism.

The genetic signatures of long-term balancing selection at a locus can roughly be divided into three categories [2]. The first signature is that the distribution of allele frequencies will be enriched for intermediate frequency alleles. This occurs because the selected locus itself is likely at moderate frequency within the population and, thus, neutral linked loci will also be at intermediate frequency. The second signature is the presence of trans-specific polymorphisms, which are polymorphisms that are shared among species [7]. This is a result of alleles being maintained over long evolutionary time periods, sometimes for millions of years [8]–[10]. The third signature is an increased density of polymorphic sites. This is due to linked neutral loci sharing similar deep genealogies as that of the selected site, increasing the probability of observing mutations at the neutral loci.

The majority of selection scans in humans have focused on positive and negative directional selection. These studies have found evidence of both types of selection, with negative selection being ubiquitous, and the amount and mechanism of positive selection currently being debated [11]–[13]. However, it is unclear how much balancing selection exists in the human genome. Some scans for balancing selection (e.g., Bubb *et al.* [14] and Andrés *et al.* [15]) have been carried out using summary statistics such as the Hudson-Kreitman-Aguadé (HKA) test [16] and Tajima's 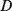 [17] as well as combinations of summary statistics [15], [18] (though see Ségural *et al*. [7] and Leffler *et al.* [19] for recent complementary approaches). The power of such approaches in unclear, and so it is uncertain how important balancing selection is in the human genome. Because balancing selection shapes the genealogy of a sample around a selected locus, more power can be gained by implementing a model of the genealogical process under balancing selection [20], [21]. Composite likelihood methods have proven to be extremely useful for the analysis of genetic variation data using complex population genetic models. [22]–[28]. This approach allows estimation under models without requiring full likelihood calculations, permitting many complex models to be investigated.

In this article, we develop two composite likelihood ratio methods to detect balancing selection, which we denote by 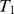 and 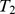. These methods are based on modeling the effect of balancing selection on the genealogy at linked neutral loci (e.g., Kaplan *et al.* (1988) [20] and Hudson and Kaplan (1988) [21]) and take into consideration the spatial distributions of polymorphisms and substitutions around a selected site. Through simulations, we show that our methods outperform both HKA and Tajima's 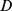 under a variety of demographic assumptions. Further, we apply our methods to autosomal whole-genome sequencing data consisting of nine unrelated European (CEU) and nine unrelated African (YRI) individuals. We find support for multiple targets of balancing selection in the human genome, including previously hypothesized regions such as the human leukocyte antigen (HLA) locus. Additionally, we find evidence for balancing selection at the *FANK1* gene, which we hypothesize to result from segregation distortion.

## Results

### Theory

#### A new test for balancing selection

In this section, we provide a basic overview of a new test for balancing selection, and we describe the method in greater detail in the sections entitled *Kaplan-Darden-Hudson model*, *Solving the recursion relation*, *A composite likelihood ratio test based on polymorphism and substitution*, and *A composite likelihood ratio test based on frequency spectra and substitutions* sections. We have developed a new statistical method for detecting balancing selection, which is based on the model of Kaplan, Darden, and Hudson [20], [21] (full details provided in the *Kaplan-Darden-Hudson model* section). Under this model, we calculate the expected distribution of allele frequencies using simulations, and approximate the probability of observing a fixed difference or polymorphism at a site as a function of its genomic distance to a putative site under balancing selection. Using these calculations, we construct composite likelihood tests that can be used to identify sites under balancing selection, similar to the approaches by Kim and Stephan [23] and Nielsen *et al.* [26] for detecting selective sweeps.

#### Basic framework

**Figure 1:**
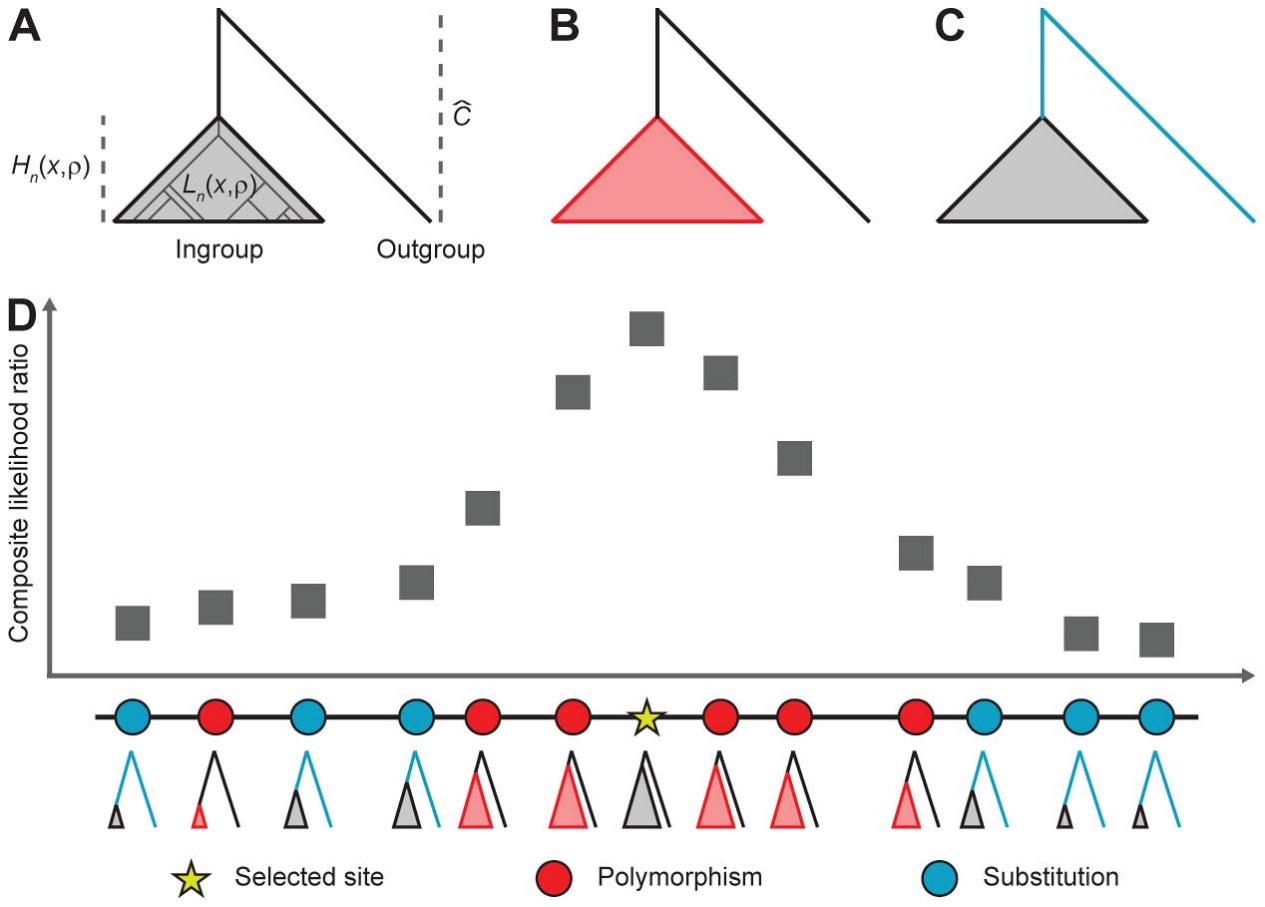
Calculation of probabilities of polymorphism and substitution under a model of balancing selection and the incorporation of these probabilities into a genome scan. (*A*) Relationship among tree length 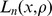, tree height 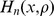 and inter-specific coalescence time 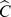. (*B*) A site is polymorphic if a mutation occurred on the 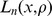 length of branches until the most recent common ancestor of the ingroup sample (red region). (*C*) A site is a substitution if a mutation occurred on the 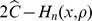 length of branches that represent the divergence between the outgroup species and the most recent common ancestor of the ingroup species (blue region). (*D*) Height and length of genealogies in relationship to their spatial proximity to a selected site and how the shapes of these genealogies affect the pattern of polymorphism around the site. The composite likelihood ratio is high near a selected site as there is an excess of polymorphisms close to the site and a deficit far from the site.

Consider a biallelic site 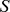 that is under strong balancing selection and maintains an allele 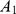 at frequency 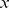 and an allele 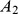 at frequency 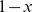. Consider a neutral locus 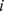 that is linked to the selected locus 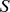. Denote the scaled recombination rate between the selected locus and the neutral locus as 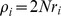, where 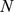 is the diploid population size and 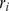 is the per-generation recombination rate. Assume we have a sample of 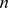 genomes from an ingroup species (e.g., humans) and a single genome from an outgroup species (e.g., chimpanzee). From these data, we can estimate the genome-wide expected coalescence time 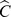 between the ingroup and outgroup species (see *Materials and Methods* for details). Also, under the Kaplan-Darden-Hudson model, we can obtain the expected tree length 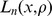 and height 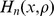 for a sample of 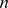 lineages affected by balancing selection by solving a set of recursive equations using the numerical approach described in the *Solving the recursion relation*. The relationship among 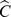, 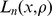, and 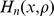 is depicted in Figure 1*A*. Assuming a small mutation rate, the probability that a site is polymorphic under a model of balancing selection, given that it contains either a polymorphism or a substitution (fixed difference), is 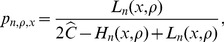
(1)and the conditional probability that it contains a substitution is 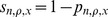. That is, conditional on a mutation occurring on the genealogy relating the 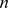 ingroup genomes and the outgroup genome, the probability that a site is polymorphic is the probability that a mutation occurs before the most recent common ancestor of the 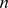 ingroup species (*i.e.*, mutation occurs on red branches indicated in Fig. 1*B*), and the probability that a site contains a substitution is the probability that a mutation occurs along the branch leading from the outgroup sequence to the most recent common ancestor of the 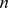 ingroup species (*i.e.*, mutation occurs on blue branches indicated in Fig. 1*C*).

Figure 1*D* shows how the spatial distribution of polymorphism around a selected site is influenced by the underlying genealogy at the site and how this spatial distribution of polymorphism can be used to provide evidence for balancing selection. Within a window of sites, we can obtain the composite likelihood that a particular site is under selection by multiplying the conditional probability of observing a polymorphism or a substitution at every other neutral site as a function of the distance of the neutral site to the balanced polymorphism.

#### Kaplan-Darden-Hudson model

The genealogy of a neutral locus 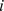 linked to the selected locus 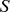 can be traced back in time using the Kaplan, Darden, and Hudson [20], [21] model, which provides a framework for modeling the coalescent process at a neutral locus that is linked to a locus under balancing selection. This model assumes that the selected locus maintains a balanced polymorphism that is infinitely old. Their framework involves modeling selection as a structured population containing two demes representing each of the two allelic classes and migration taking the role of recombination and mutation. Lineages within the first deme are linked to 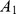 alleles and lineages within the second deme are linked to 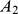 alleles. Lineages migrate between demes by changing their genomic background. That is, a lineage in the first deme will migrate to the second deme if there was a mutation that changed an 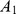 allele to an 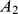 allele or if there was a recombination event that transferred a lineage linked to an 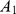 allele to an 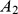 background. Similarly, a lineage in the second deme will migrate to the first deme if there was a mutation that changed an 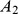 allele to an 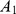 allele or if there was a recombination event that transferred a lineage linked to an 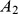 allele to an 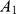 background. The rate at which a lineage linked to an 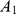 background transfers to an 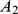 background is 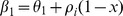 and the rate at which a lineage linked to an 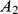 background transfers to an 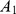 background is 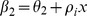.

Consider a sample of 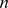 lineages with 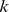 lineages linked to allele 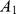 (*i.e.*, in the first deme) and 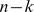 lineages linked to allele 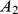 (*i.e.*, in the second deme). Given this configuration, only four events are possible. The first event involves a coalescence of a pair of lineages linked to 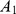 alleles, the second involves a coalescence of a pair of lineages linked to 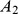 alleles, the third involves the transfer of a lineage from an 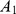 background to an 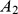 background, and the fourth involves the transfer of a lineage from an 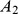 background to an 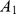 background. The time until the first event (*i.e.*, a coalescence or a transfer of background) is exponentially distributed with rate 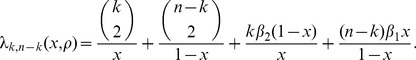
(2)


The probability that the event is a coalescence of a pair of 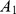-linked lineages is 
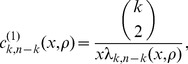
(3)the event is a coalescence of a pair of 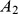-linked lineages is 
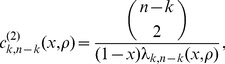
(4)the event is a transfer from an 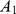 to an 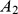 background is 
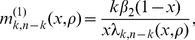
(5)and the event is a transfer from an 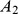 to an 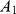 background is 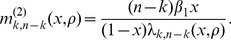
(6)Note that in the notation of Kaplan *et al.* (1988) [20], 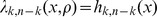, 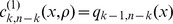, 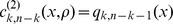, 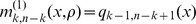, and 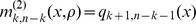.

Let 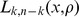 denote the expected tree length given a sample with 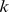 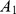-linked lineages and 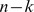 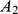-linked lineages. Using eq. 18 of Kaplan *et al.* (1988) [20], the expected total tree length can be expressed using the recursion relation 
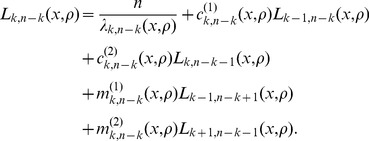
(7)


Similarly, the expected tree height 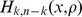 given a sample with 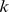 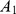-linked lineages and 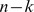 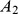-linked lineages can be expressed by 
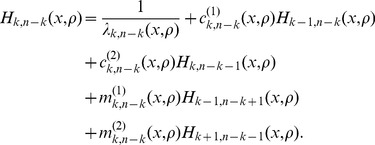
(8)


### Solving the recursion relation

Consider a sample of 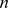 lineages. Denote the 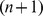-dimensional vector of tree lengths for a sample of size 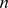 as 
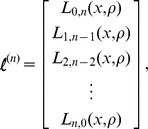
such that element 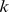, 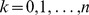, of 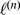 is 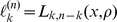. Next, define the (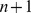)-dimensional vector 
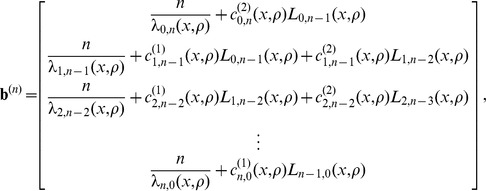
such that element 0 is 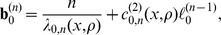
element 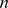 is 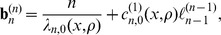
and element 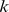, 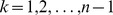 is 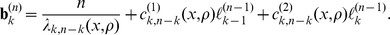



Further, consider an 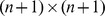-dimensional tridiagonal matrix of migration rates 
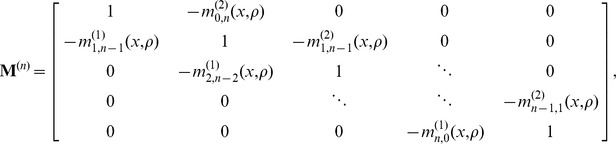
with 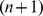-dimensional main diagonal 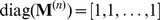, 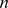-dimensional lower diagonal 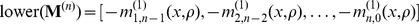, and 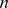-dimensional upper diagonal 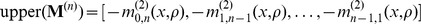. All elements that do not fall on the main, lower, and upper diagonals of 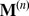 are zero.

Given 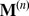, 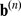, and 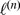, we can rewrite the recursion relation in eq. 7 as system of equations 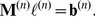
(9)


Because we can calculate eqs. 5 and 6, 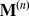 is a constant matrix. For a sample of size 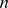, suppose we know 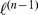 for a sample of size 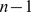. Therefore, 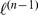 is now a constant vector and hence, because we can calculate eqs. 2–4, 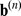 is also a constant vector. Therefore, eq. 9 is a tridiagonal system of 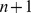 equations with 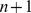 unknowns, which can be solved in 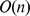 time using the tridiagonal matrix algorithm [29].

The base case for the recursion in eq. 8 is when the number of lineages equals one. That is, when all lineages have coalesced and the most recent common ancestor is linked either to an 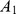 allele or to an 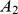 allele. This base case can be represented by 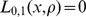 and 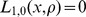. Given these values, set 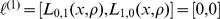 and solve the system of equations 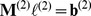 for 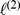. Next, given 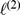, solve the system of equations 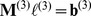 for 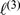. Iterate this processes until 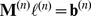 is solved for 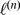. An analogous process can be used to solve the recursion (eq. 8) for the expected tree height.

Using the framework in this section for a sample of size 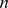, we can obtain values for 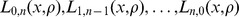. Given that the 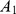 allele has frequency 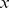 and the 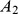 allele has frequency 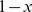, the expected tree length for a sample of size 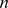 is 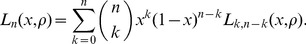
(10)


Similarly, we can obtain the expected tree height 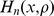 for a sample of size 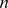. The tree heights and total branch lengths are then used in eq. 1 to compute the likelihood of the data under the selection model.

#### A composite likelihood ratio test based on polymorphism and substitution

In this section, we illustrate how eq. 1 can be incorporated into a composite likelihood. We will then describe a likelihood ratio test that compares the balancing selection model described above to a neutral model based on the background genome patterns of polymorphism. Consider a window of 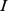 sites that are either polymorphisms or substitutions and consider a putatively selected site 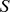 located within the window. Suppose site 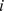 within the window has 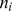 sampled alleles, 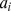 observed ancestral alleles, and is a recombination distance of 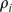 from 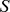. Let 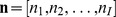, 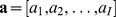, and 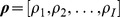. Define the indicator random variable 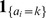 that site 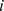 has 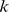 ancestral alleles. Using the Kaplan-Darden-Hudson model, the probability that site 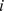 is polymorphic is 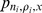 and the probability that the site is a substitution (or fixed difference) is 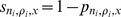. Under the model, the composite likelihood that site 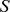 is under balancing selection is 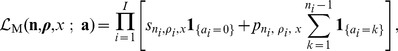
(11)which is maximized at 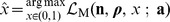. Notice that sampling distribution for a site depends on the distance to the selected locus. In this method, as in previous composite likelihood methods for detecting selection, there is therefore no need for weighting sites depending on their distance from the selected sites. Such weighting is already incorporated in the probabilistic model. Similarly, there is no need for sliding windows, or the use of Hidden Markov Models (HMMs) to indicate the selected region. The likelihood ratio can, in principle, be calculated for any point in the genome, taking all other points in the genome into account. However, for practical computational reasons, we only calculate the likelihood ratio for a site using nearby sites in a fixed window of 100 substitutions or polymorphisms upstream and downstream of the focal site. As the distance from the selected site increases, little is gained by incorporating information from more sites.

Further, suppose that for a sample of size 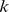, 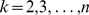, conditioning only on sites that are polymorphisms or substitutions, the proportion of loci across the genome that are polymorphic is 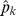 and the proportion of loci that are substitutions is 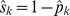. Then the composite likelihood that site 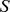 is evolving neutrally is 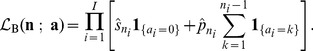
(12)


It follows that the composite likelihood ratio test statistic that site 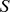 is under balancing selection is 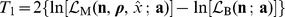.

#### A composite likelihood ratio test based on frequency spectra and substitutions

A balanced polymorphism not only increases the number of polymorphisms at linked neutral sites, but also leads to an increase in minor allele frequencies at these sites. Therefore, power can be gained by using frequency spectra information in addition to information on the density of polymorphisms and substitutions.

Given a sample of size 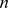, an 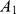 allele at frequency 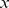, 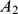 allele at frequency 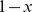, and a polymorphic neutral site that is 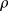 recombination units from a selected site, we can obtain the probability 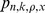 that there are 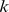, 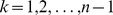, ancestral alleles observed at the neutral site. The composite likelihood that site 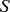 is under balancing selection is 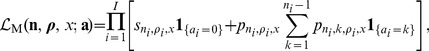
(13)which is maximized at 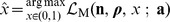.

Further, suppose that for a sample of size 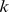, 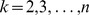, conditioning only on sites that are polymorphisms or substitutions, the proportion of polymorphic loci across the genome that have 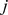, 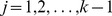, ancestral alleles is 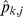. Then the composite likelihood that site 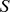 is evolving neutrally is 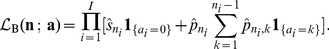
(14)


It follows that the composite likelihood ratio test statistic that site 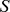 is under balancing selection is 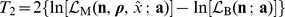. Because it is computationally difficult to derive analytical formulas for frequency spectra under the Hudson-Darden-Kaplan model, we approximate these distributions by simulating frequency spectra under the Hudson-Darden-Kaplan model for a range of equilibrium frequencies 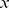 and recombination parameters 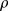. We then use a look-up table to identify the optimal spectrum to use, and if the optimum is intermediate between two spectra, the two closest distributions are employed. The two new methods, 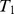 and 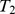, have been implemented in the software package *BALLET* (BALancing selection LikElihood Test), which is written in C and is available at http://www.personal.psu.edu/mxd60/software.html.

### Evaluating the methods using simulations

To evaluate the performance of 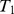 and 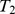 relative to HKA and Tajima's 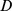, we carried out extensive simulations of balancing selection using different selection and demographic parameters. We simulated genomic data for a pair of species that diverged 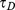 years ago. We introduced a site that is under balancing selection at time 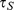, and the mode of balancing selection at the site is overdominance with selection strength 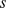 and dominance parameter 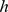. In the simulations discussed in this article, we varied the demographic history in the target ingroup species, the strength of selection 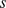, the dominance parameter 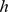, and the time at which the selected allele arises 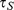. We consider two values for the strength of selection, 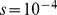 and 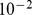, five values for the dominance parameter, 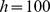, 10, 3, 1.5, and 1.125, and three times at which the selected allele arises, 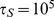, 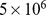, and 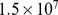 years ago. Under the overdominance model considered here, the equilibrium frequency occurs at 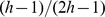 yielding equilibrium frequencies of 0.50, 0.47, 0.40, 0.25, and 0.10 for 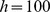, 10, 3, 1.5, and 1.125, respectively. These parameters were chosen to represent strong (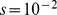) and substantially weaker (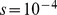) selection coefficients and a range of equilibrium frequencies. In addition, the time 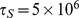 years ago was meant to represent an ancient balanced polymorphism, whereas the other two values for 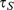 represent violations of assumptions of our methods. That is, the trans-species polymorphism occurring at 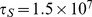 years ago violates the assumption that lineages from the ingroup species are necessarily monophyletic, and the recent balanced polymorphism arising 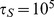 years ago represents balancing selection on an allele that is young relative to the average coalescence time for the ingroup species. Details of how the simulations were implemented are further described in the *Materials and Methods* section.

#### Ancient balanced polymorphism

**Figure 2:**
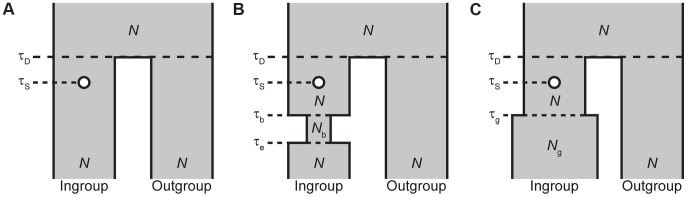
Demographic models used in simulations in which a selected allele arises after the split a pair of species. (*A*) Divergence model. Model parameters are a diploid effective population size 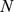, divergence time 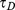 of the ingroup and outgroup species, and the time 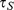 when the selected allele arises. (*B*) Divergence model with a recent bottleneck within the ingroup species. Additional model parameters are the diploid effective population size 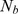 during the bottleneck, the time 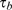 when the bottleneck began, and the time  when the bottleneck ended. (*C*) Divergence model with recent population growth within the ingroup species. Additional model parameters are the current diploid effective population size 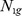 after recent growth and the time 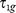 when the growth occurred.

**Figure 3:**
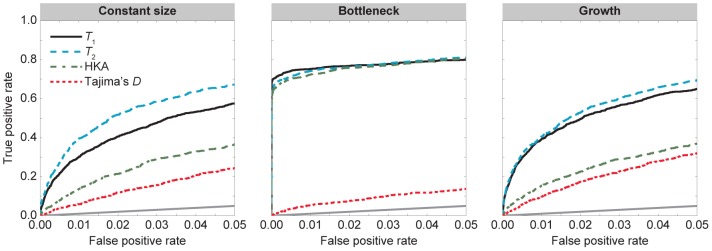
Performance of 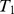, 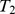, HKA, and Tajima's 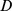 under the demographic models in Figure 2 with selection parameter  and dominance parameter 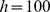. The first column is the divergence model in Figure 2*A*. The second column is the divergence model in Figure 2*B* with a recent bottleneck within the ingroup species. The third column is the divergence model in Figure 2*C* with recent population growth within the ingroup species.

We performed simulations under each of the three demographic models depicted in Figure 2. For these simulations, we constructed receiver operator characteristic (ROC) curves to illustrate relationships between the true and false positive rates of each method. Figure 3 displays ROC curves for 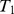, 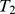, HKA, and Tajima's 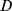 for simulations where 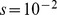 and 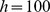. Under a model of constant population size (left panel of Fig. 3), 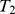 tends to obtain more true positives than 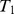,  more true positives than HKA, and HKA more true positives than Tajima's 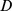 (left panel of Fig. 3). In practice, however, we are typically concerned with a method's performance at low false positive rates. For a false positive rate of 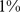, 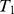, 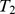, HKA, and Tajima's 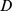 have true positive rates of 30, 40, 14, and 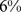, respectively. Similarly, at a false positive rate of 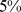, 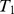, 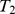, HKA, and Tajima's 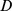 have true positive rates of 58, 67, 37, and 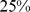, respectively. These results show that 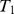 and 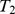 each vastly outperforms both HKA and Tajima's 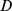, with 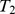 performing better than 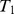. However, these simulations were performed using the standard neutral model, which is also the demographic model assumed in 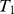 and 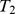. Thus, to examine the robustness of our methods, we next considered two complex demographic scenarios that could potentially affect the results of our methods—a population bottleneck (Fig. 2*B*) and a population expansion (Fig. 2*C*).

The middle panel of Figure 3 displays ROC curves under a model in which the ingroup species experiences a recent severe bottleneck (Fig. 2*B*). For a false positive rate of 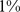, the true positive rates of 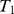, 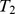, HKA, and Tajima's 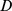 are 75, 74, 72, and 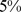, respectively. Similarly, for a false positive rate of 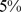, the true positive rates of 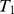, 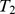, HKA, and Tajima's 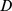 are 80, 81, 80, and 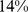, respectively. Thus, aside from Tajima's 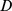, all methods perform well under this demographic model. This is because a severe population bottleneck decreases levels of diversity across the genome, resulting in a lower polymorphism-to-substitution ratio. Because 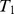, 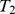, and HKA all compare levels of polymorphism and divergence at a putatively selected site to those of the corresponding genomic background, these methods are able to identify the increased diversity at a site under balancing selection. In contrast, Tajima's 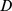 does not perform such a comparison and, thus, has little power to detect balancing selection under this demographic scenario.

The right panel of Figure 3 depicts ROC curves under a demographic model in which the ingroup species experiences recent population growth (Fig. 2*C*). As with constant population size, 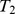 tends to obtain more true positives than 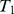, 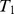 more true positives than HKA, and HKA more true positives than Tajima's 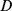 for a given false positive rate. At a false positive rate of 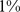, 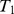, 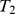, HKA, and Tajima's 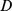 have true positive rates of 39, 41, 15, and 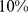, respectively, and at a false positive rate of 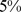, 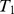, 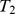, HKA, and Tajima's 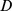 have true positive rates of 65, 69, 37, and 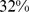, respectively. Interestingly, all four methods perform better under a recent population growth than under a constant population size. This result is potentially due to less fluctuation in the frequency of a selected allele in the recent past when the population size is large.

By considering the demographic models in Figure 2, we have shown that 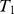 and 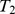 generally outperform both HKA and Tajima's 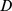. Next, we investigated the effect of varying 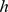 (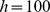, 10, 3, and 1.5) when 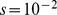 (Fig. S1). Under a model with constant population size (Fig. 2*A*), 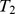 outperforms 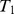, 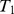 outperforms HKA, and HKA outperforms Tajima's 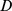. As 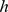 decreases, the performances of HKA and Tajima's 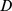 decrease, whereas the performances of 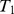 and 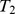 are not dramatically affected. Under a model with a recent population bottleneck (Fig. 2*B*), 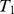, 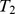, and HKA all perform well, whereas Tajima's 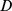 performs poorly. In this scenario, 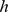 appears to have little influence on the relative performance of these methods. Finally, under a model with a recent population expansion (Fig. 2*C*), 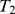 outperforms 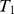, 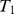 outperforms HKA, and HKA outperforms Tajima's 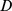. Decreasing 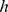 results in a decrease in the performance of Tajima's 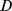, but has little influence on the performances of all other methods. Moreover, the performances of 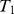 and 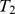 are similar for all 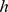, whereas the perforances of HKA and Tajima's 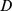 are similar for large 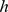 (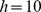 and 100), and dissimilar for low 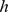 (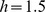 and 3).

**Figure 4:**
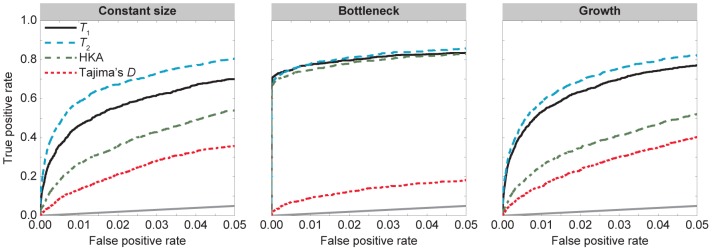
Performance of 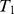, 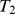, HKA, and Tajima's 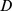 under the demographic models in Figure 2 with selection parameter 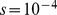 and dominance parameter 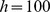. The first column is the divergence model in Figure 2*A*. The second column is the divergence model in Figure 2*B* with a recent bottleneck within the ingroup species. The third column is the divergence model in Figure 2*C* with recent population growth within the ingroup species.

For 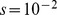, 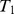 and 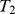 generally perform quite well (Figs. 3 and S1). However, because 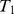 and 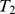 were developed to detect long-term balancing selection of infinite strength, it is unclear how the methods perform under weak selection. To investigate this scenario, we considered 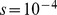, with 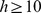 representing relatively strong balancing selection (*i.e.*, relatively high 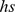) and 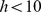 representing relatively weak balancing selection (*i.e.*, relatively low 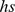). For 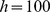 (Fig. 4), we find that the relative performance of the four methods are similar to those in the case of strong selection (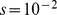). Curiously, all methods perform better when 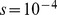 (Fig. 4) than when 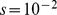 (Fig. 3). To investigate the factors influencing this strange behavior, we plotted the mean difference in the number of polymorphic sites for a scenario with 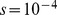 and 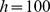 verses one with 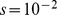 and 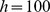 as function of the distance from the site under balancing selection (Fig. S2). We find that, on average, there are more polymorphic sites when the selection coefficient is weak, with the difference in numbers of polymorphic sites disappearing with increasing distance from the site under selection. This phenomenon is due to a drop in local effective population size near the site under balancing selection for the scenario with strong selection. Because 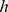 is so large (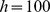) and the population size is finite, heterozygous individuals leave a disproportionately large fraction of offspring in the next generation, therefore causing an apparent drop in local effective size near the site under selection.

When 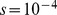 under a model of constant population size (Fig. 2*A*), 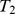 outperforms 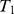, 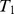 outperforms HKA, and HKA outperforms Tajima's 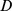 when 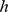 is large (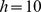 and 100; Fig. S3), similar to what we observe when 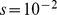 (Fig. S1). In contrast to our observations when 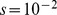, all methods perform poorly when 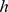 is small (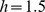 and 3), each identifying signatures of selection only slightly better than random (Fig. S3). Hence, when the selection coefficient is weak and the level of overdominance is low, 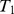 and 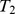 cannot extract enough information from the data to make meaningful predictions. However, HKA and Tajima's 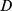 perform just as poorly, and therefore 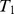 and 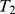 generally outperform HKA and Tajima's 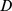 under a demographic model with constant population size.

Next, when 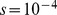 under a model with a recent population bottleneck (Fig. 2*B*), 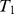, 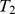, and HKA all perform well, whereas Tajima's 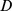 performs poorly (Fig. S3), similar to what we observe when 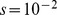 (Fig. S1). In contrast to the results for 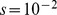, 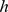 has some influence on the relative performance of these methods. As 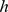 decreases, the performance of all methods decreases—though not substantially. In addition, similarly to what we observe when 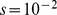, the performances of 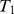, 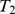, and HKA are approximately the same. Hence, even under weak selection coefficients, population bottlenecks tend to enhance the performance of 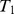, 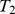, and HKA, whereas they inhibit the performance of Tajima's 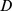.

Finally, when 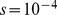 under a model with a recent population expansion (Fig. 2*C*), 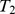 outperforms 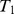, 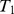 outperforms HKA, and HKA outperforms Tajima's 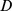 for large 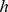 (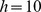 and 100; Fig S3), as observed when 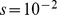 (Fig. S1). In contrast to the results for the case of 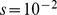, all methods perform poorly when 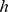 is small (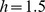 and 3). Hence, like the case under constant population size, when the selection coefficient is weak and the level of overdominance is low, 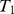 and 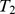 cannot extract enough information from the data to make meaningful predictions. However, HKA and Tajima's 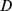 perform just as poorly, and therefore 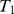 and 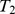 generally outperform HKA and Tajima's 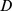 under a demographic model with recent population growth.

So far the lowest dominance parameter considered here was 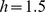, which has an equilibrium frequency of 0.25. To further assess the limits of our methods, we considered 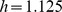, which has a substantially smaller equilibrium frequency of 0.10. When 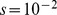, we find that all four methods perform poorly under the constant population size (Fig. 2*A*) and growth (Fig. 2*C*) models (Fig. S4). In contrast, as with the higher equilibrium frequencies (Fig. S1), 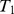, 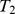, and HKA statistics performed well, whereas Tajima's 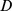 performed poorly under the bottleneck (Fig. 2*B*) model (Fig. S4).

We next examined violations in recombination rate assumptions of 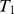 and 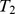 by investigating the robustness of 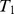 and 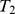 to error in recombination rate estimation. For each simulation, we assumed a recombination rate of 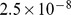 per site per generation. We first wanted to investigate whether using an incorrect recombination map would increase the chances that 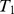 and 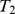 identify false positive. Figure S5 depicts results under a model with constant population size (Fig. 2*A*) in which there is no selected allele. With respect to identifying false signals of balancing selection, our results indicate that 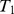 and 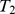 are robust to recombination rate underestimation and overestimation. We next wanted to examine whether using an incorrect recombination map would influence the power of 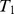 and 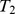 to identify ancient balanced polymorphisms. Figure S6 depicts results for a model with constant population size (Fig. 2*A*) with time of selection 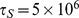, 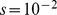, large (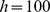) and small (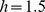) dominance parameters, and recombination rate overestimated by one or two orders of magnitude and underestimated by one or two orders of magnitude. We do not consider 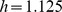 due to the poor performance of all methods considered here for that parameter setting. Incorrectly inferring an order of magnitude higher recombination rate slightly improves the performance of both 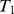 and 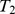. However, incorrectly inferring a two orders of magnitude higher recombination rate yields poor performance for both 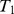 and 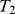 under reasonable false positive rates (e.g., less than 5%). Incorrectly inferring the recombination rate by one or two orders of magnitude lower than the truth does not vastly alter the power for 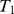, but substantially decreases the power of 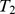.

#### Ancient trans-species balanced polymorphism

One hallmark of balancing selection is that it maintains polymorphism for a long time, potentially for millions of years [8]–[10]. Thus, some balanced polymorphisms, referred to as trans-specific polymorphisms, are shared across multiple species. Figure S7 displays the three demographic models that we consider in which a selected allele arises in the population ancestral to the split of the ingroup and outgroup species. For each demographic scenario, we set 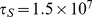 years ago, creating a selected allele that is three times as ancient as the one that we consider in Figure 2. All other model parameters are identical to those considered in Figure 2.

Figures S8 and S9 indicate that the performances of 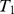, 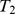, HKA, and Tajima's 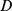 are not greatly affected by considering an ancient trans-species balanced polymorphism when compared to an ancient balanced polymorphism that occurred more recently than the split of a pair of species. This is important because the scenario of an ancient trans-species balanced polymorphism is a violation of the assumptions of the model since it forces lineages from the ingroup species to not be monophyletic with respect to the outgroup species. Hence, though 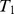 and 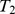 make the assumption that lineages from the ingroup species are monophyletic, this assumption does not hinder the methods in practice.

#### Young balanced polymorphism

The two methods developed in this article assume that selection is infinitely strong and that the balanced polymorphism is infinitely old. Here we consider the performance of 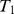, 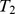, HKA, and Tajima's 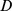 under a scenario in which a young balanced polymorphism arose 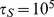 years ago. Considering selection coefficients 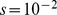 (Fig. S10) and 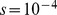 (Fig. S11), all four methods performed poorly under the constant size and growth demographic scenarios, regardless of the dominance parameter. In contrast, 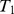, 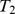, and HKA all perform well and Tajima's 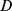 performs poorly under the bottleneck scenario, similar to the results for the ancient balanced polymorphisms. These results show that the new methods have limited power to detect young balanced polymorphisms, except under a scenario in which the background density of polymorphisms is substantially lowered—as in the case of a strong recent population bottleneck.

#### Matching the mean density of polymorphisms to a constant size model

The alternate demographic scenarios that we investigated here have focused on the performance of 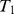, 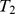, HKA, and Tajima's 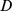 for a recent population bottleneck or growth, relative to a constant size population. However, we have not considered whether a population bottleneck or growth actually changes the absolute performance of the methods, as these demographic events not only change the density of polymorphisms relative to constant size models, but they also change the shape of the frequency spectrum. To control for the density of polymorphisms, we chose the ancestral effective size under the bottleneck and growth models so that the expected number of segregating sites under the bottleneck and growth models is the same as a constant size model of diploid effective size 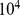. That is, we set the ancestral sizes for complex demographic models such that these complex models yield identical mean densities of polymorphic sites as a model of constant population size of 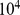 diploid individuals. The details on how we chose these ancestral effective sizes can be found in the *Materials and Methods* section, with the ancestral diploid effective sizes under the bottleneck and growth models as 14015 and 8762, respectively.

**Figure 5:**
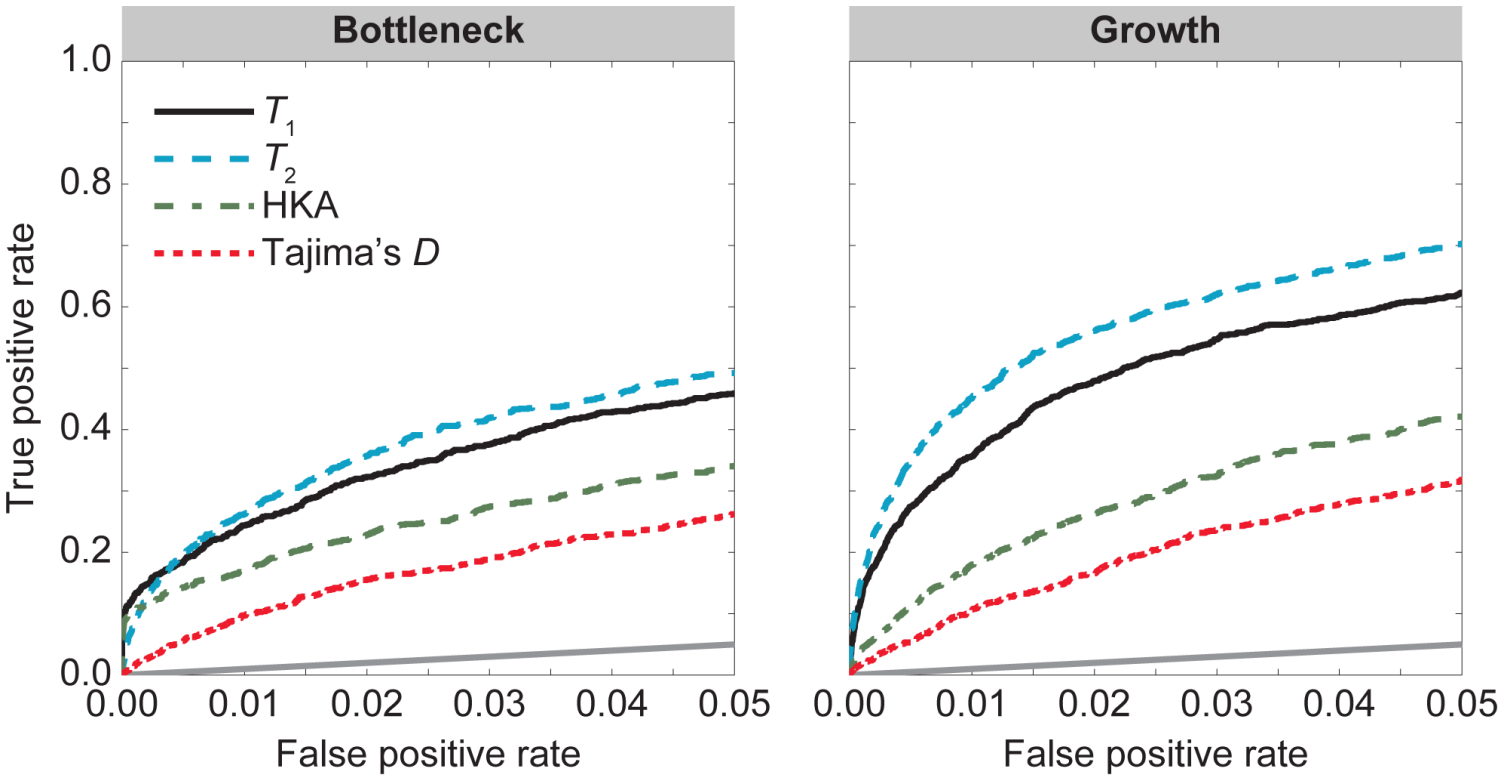
Performance of 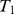, 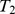, HKA, and Tajima's 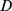 under the bottleneck and growth demographic models in Figure 2 with selection parameter 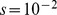 and dominance parameter 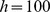. The left panel is the divergence model in Figure 2*B* with a recent bottleneck within the ingroup species. The right panel is the divergence model in Figure 2*C* with recent population growth within the ingroup species. The population sizes for the bottleneck and growth demographic histories have been scaled so that they produce the same number of segregating sites as a constant size population with diploid effective size 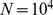 individuals.

**Figure 6:**
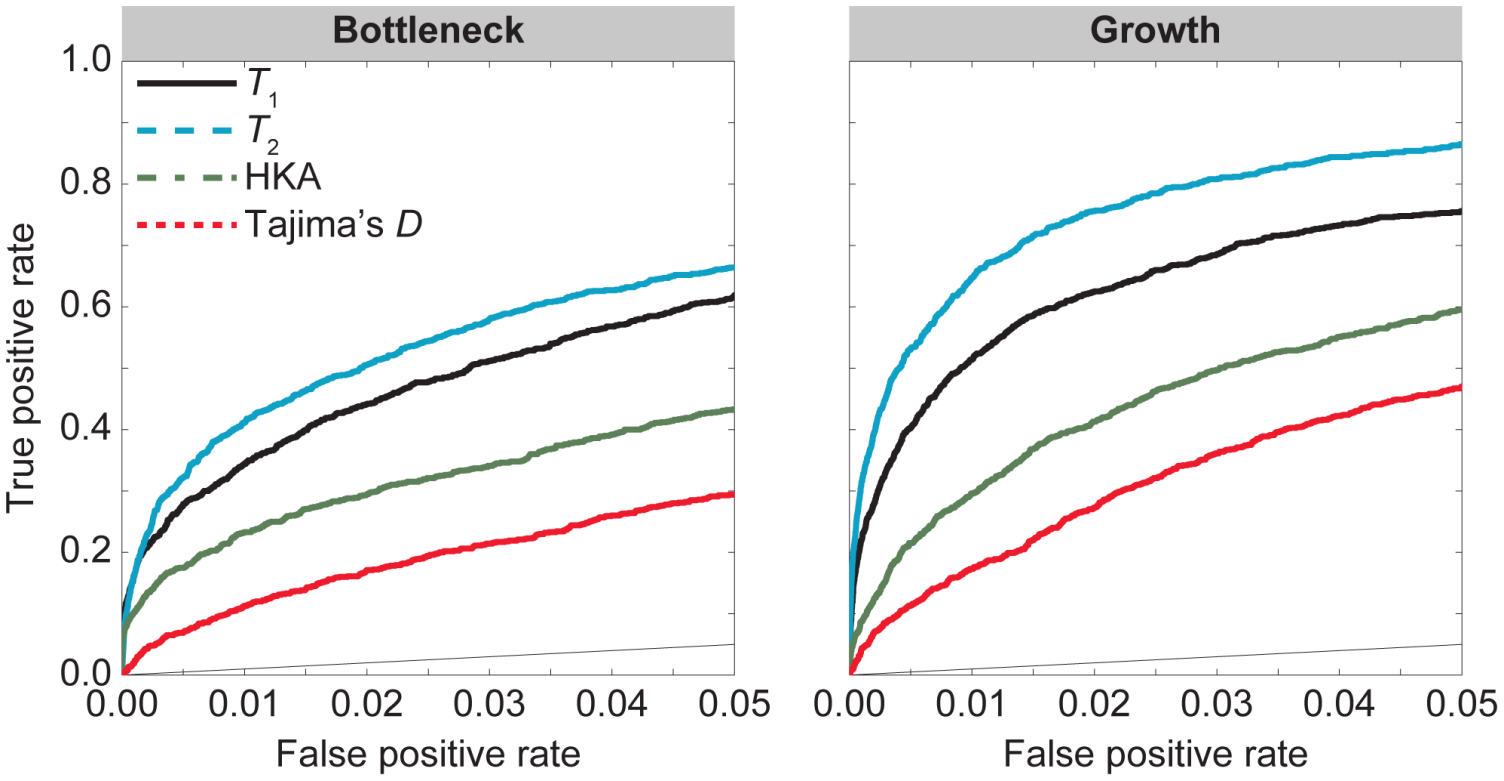
Performance of 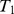, 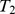, HKA, and Tajima's 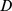 under the bottleneck and growth demographic models in Figure 2 with selection parameter 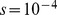 and dominance parameter 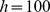. The left panel is the divergence model in Figure 2*B* with a recent bottleneck within the ingroup species. The right panel is the divergence model in Figure 2*C* with recent population growth within the ingroup species. The population sizes for the bottleneck and growth demographic histories have been scaled so that they produce the same number of segregating sites as a constant size population with diploid effective size 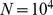 individuals.

Figures S12 and S13, Figures S14 and S15, and Figures S16 and S17 display results for times 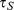 at which a balanced polymorphism arose of 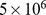, 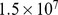, and 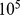 years ago, respectively. Interestingly, these results indicate that the bottleneck and growth models behave similarly to a constant size model once the mean density of polymorphic sites is matched to that of a constant size model. That is, there no longer is a substantial improvement for 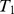, 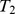, and HKA for bottleneck models relative to a constant size model. Hence, it is not the shape of the frequency spectrum that gave the apparent increase in power under the bottleneck model (e.g., compare Fig. 3 to Fig. 5 and Fig. 4 to Fig. 6). Rather, it was the large decrease in the background density of polymorphisms relative to that of the assumed effective population size under the model of balancing selection. In addition, when matching the mean density of polymorphisms, methods tended to perform better under the growth model than under the bottleneck model (e.g., Figs. 5 and 6), counter to what was observed without matching the mean density of polymorphisms (e.g., compare Fig. 3 to Fig. 5 and Fig. 4 to Fig. 6). This observation is potentially due to the increased variance in coalescence times under the new bottleneck model compared to the new growth model, when the mean density of polymorphisms is matched to a constant size model.

### Empirical analysis

#### Balancing selection in humans

We probed the effects of balancing selection in humans by using whole-genome sequencing data from nine unrelated individuals from the CEU population and nine unrelated individuals from the YRI population (see *Materials and Methods*). We performed a scan for balancing selection at each position in our dataset by considering a window of 100 substitutions or polymorphisms upstream and downstream of our focal site. This window size was taken for computational convenience, rather than by consideration of the recombination rate or polymorphism density within the region. Though we used a window size of 200 polymorphisms or substitutions for computational convenience, 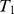 and 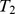 can also be computed using all sites on a chromosome. The mean window length was ∼14.7 kb for the CEU and ∼13.7 kb for the YRI populations, which should be sufficiently long because recombination quickly breaks down the signal of balancing selection at distant neutral sites. That is, under the Hudson-Darden-Kaplan model, the scale at which one would observe an increase in diversity is 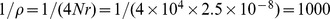 nucleotides, or a 1 kb window [21]. Manhattan plots for 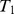 (Figs. S18 and S19) and 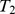 (Figs. S20 and S21) test statistics suggest that there are multiple outlier candidate regions. Intersecting the locations of these scores with those from the longest transcript of each RefSeq gene (*i.e.*, transcription start to stop including exons and introns) led to identification of many previously-hypothesized and novel genes potentially undergoing balancing selection (see Tables S1–S4, with previously-hypothesized genes highlighted in bold).

Multiple genes at the HLA region are strong outliers (top 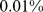 of all scores across the genome) in our scan for balancing selection (Tables S1–S4). Because this study uses high-coverage sequencing data, resolution in the HLA region is particularly fine (Figs. S22 and 7), with strong signals in classical MHC genes such as *HLA-A*, *HLA-B*, *HLA-C*, *HLA-DR*, *HLA-DQ*, and *HLA-DP* genes [14]. The HLA region, which is located on chromosome six, is a well-known site of balancing selection in humans [8]–[10]. The protein products encoded by HLA genes are involved in antigen presentation, thus playing important roles in immune system function. Genes at the HLA locus are known to be highly polymorphic and are thought to be subject to balancing selection due to frequency-dependent selection, overdominance, or fluctuating selection in a rapidly changing pathogenic environment [30], [31]. As the HLA region is so well known as a locus under balancing selection, it is important that our methods identify strong candidate candidate genes in the regions as a proof of concept.

**Figure 7:**
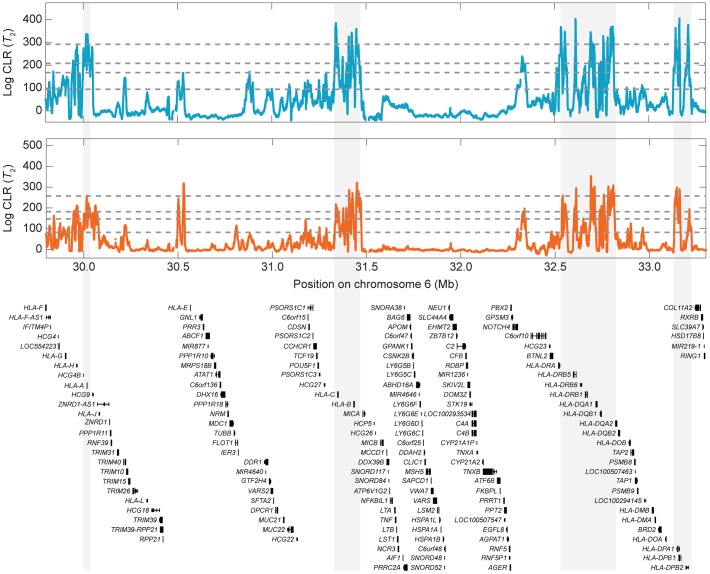
Signals of balancing selection within the HLA region for the CEU (blue) and YRI (orange) populations using the 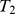 test statistic. From bottom to top, the horizontal dotted gray lines indicate the 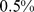, 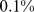, 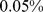, and 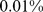 empirical cutoffs, respectively.

**Figure 8:**
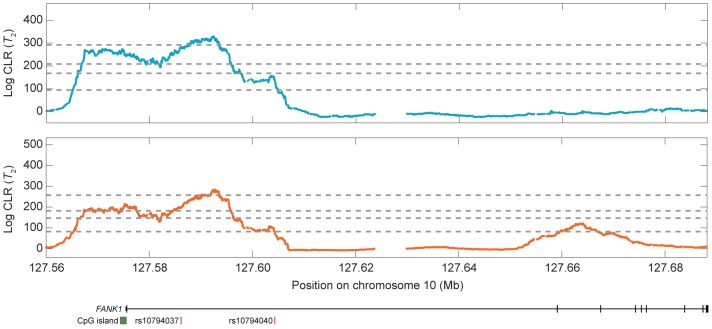
Signal of balancing selection at the *FANK1* gene for the CEU (blue) and YRI (orange) populations using the 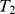 test statistic. From bottom to top, the horizontal dotted gray lines indicate the 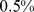, 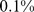, 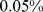, and 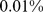 empirical cutoffs, respectively. SNPs (rsIDs) correspond to markers showing significant levels of transmission distortion within the Meyer *et al.* study [37].

One gene that we found particularly intriguing is *FANK1* (Figs. S23 and 8). This gene is one of the top four candidates in the CEU and YRI populations when using either the 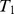 or 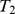 statistic (Tables S1–S4). In addition, *FANK1* is the top candidate among genes that have not been previously hypothesized to be under balancing selection when using either test in the CEU and the 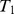 test in the YRI. *FANK1* is expressed during the transition from diploid to haploid state in meiosis [32], [33]. Though it is often identified as spermatogenesis-specific [32], [33], it is also expressed during oogenesis in cattle [34] and mice [35]. Its function is to suppress apoptosis [33], and it is one of ten to 20 genes identified as being imprinted in humans (*i.e.*, allele specific methylation) [36]. Interestingly, it also shows marginal evidence of segregation distortion (Fig. 8) [37]. Further, as a CpG island resides directly underneath our signal in both the CEU and YRI populations, we analyzed the region around *FANK1* with all 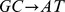 transitions on chromosome 10 removed as well as all transitions on chromosome 10 removed and we still retain the peak (Fig. S24), strongly suggesting that the signature of balancing selection that we identified around *FANK1* is not driven by CpG mutational effects. We were additionally surprised to find that the putative selection signal was approximately 40 kb wide, which is abnormally large for balancing selection. Looking back at the recombination map, we find that the rates in this region are extremely low, which explains the large width of the peak. However, Figures S5 and S6 indicate that erroneously inferring a lower recombination rate does not increase the power of detecting a selection signal, and can substantially impair the ability for 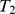 to detect a selection signal.

More broadly, a glance at the top signals for the CEU (Tables S1 and S3) and YRI (Tables S2 and S4) populations, reveals a substantial overlap in the candidate genes identified between the pair. If balancing selection has maintained a polymorphism for a long period of time, then we would expect these populations to share many signals in common due to their relatively recent population split. Tables S1–S4 indicate that our scan also identified a number of genes that were previously-hypothesized to be under balancing selection. However, the majority of this overlap is due to the HLA region. One candidate that we did not find support for was the *ABO* gene, which has been identified as a potential strong candidate using diverse complementary approaches such as summary statistics [38] and trans-specific polymorphism information [7]. A number of factors, including the small sample size for each of the CEU and YRI populations used here and potential differences in the Complete Genomics dataset relative to others, could have caused the *ABO* gene to not be at the top of our list of candidates.

#### Gene ontology analysis

To elucidate functional similarities among genes identified to be under balancing selection, we performed gene ontology (GO) enrichment analysis using *GOrilla* [39], [40]. First, we compared an unranked list of the top 100 candidate genes (Tables S1–S4) to the background list of all unique genes. Genes obtained using either test statistic are enriched for processes involved in the immune response in both the CEU and YRI populations (Tables S5–S8). Similarly, the top genes are enriched for MHC class II functional categories (Tables S9–S11), with the exception of the 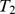 statistic applied to YRI, which has no functional enrichment. Further, these top genes tend to be components of the MHC complex and membranes (Tables S12–S15), which often directly interact with pathogens. Interestingly, removing all HLA genes from both the top 100 and background sets of genes reveals no GO enrichment for process, function, or component categories, indicating that enrichment is predominately driven by the HLA region. Because we can also provide a score for each candidate gene in our likelihood framework, we performed a second analysis in which we ranked genes by their likelihood ratio test statistic, with the goal of identifying GO categories that are enriched in top-ranked genes. Using this framework, the top candidate genes tend to be involved in immune response and cell adhesion processes (Tables S16–S19); MHC activity and membrane protein activity functions, such as transporting and binding molecules (Tables S20–S23); and MHC complex, membrane, and cell junction components (Tables S24–S27). In contrast to the case of the top 100 candidate genes, removing all HLA genes from the ranked list still resulted in GO enrichment in categories such as cell adhesion (processes), membrane protein activity (function), and components of membranes and cell junctions (component).

## Discussion

In this article, we presented two likelihood-based methods, 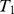 and 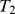, to identify genomic sites under balancing selection. These methods combine intra-species polymorphism and inter-species divergence with the spatial distribution of polymorphisms and substitutions around a selected site. Through simulations, we showed that 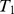 and 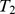 vastly outperform both the HKA test and Tajima's 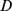 under a diverse set of demographic assumptions, such as a population bottleneck and growth. In addition, application of 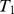 and 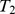 to whole-genome sequencing data from Europeans and Africans revealed many previously identified and novel loci displaying signatures of balancing selection.

Simulation results suggest that 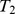 performs at least as well as 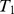, and so a natural question is whether 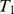 would ever be used. Based on the fact that 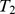 uses the allele frequency spectrum and 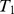 does not, then 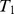 would be a valuable statistic to employ when allele frequencies cannot be estimated well. One example is a situation in which the sample size is small (e.g., one or two genomes). Under this scenario, the 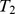 test statistic would likely provide little additional power over the 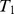 statistic. As another example, it is becoming increasingly common for studies to sequence a pooled sample of individuals rather than each individual in the sample separately. This pooled sequencing will tend to yield inaccurate estimates of allele frequencies across the genome, which could heavily influence the performance of the 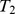 statistic. However, if there is sufficient enough evidence that a site has a pair of alleles observed in the sample, then this site can be considered polymorphic regardless of its actual allele frequency. Future developments that can statistically account for this uncertainty in allele frequency estimation could be incorporated into the 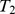 test statistic so that it can be applied to pooled sequencing data. In addition, our investigation into the robustness of 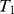 and 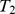 to errors in recombination rate estimates suggested that 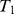 tends to perform better than 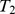 when the estimate of the recombination rate is inaccurate. Because reliable genetic maps are unavailable for most organisms that have had their genome sequenced, 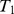 may be the preferable statistic for many current applications.

The model of balancing selection used in this article is from Hudson and Kaplan [21], and assumes that natural selection is so strong that it maintains a constant allele frequency at the selected locus forever. The simulation scenarios considered here assumed that the strength of balancing selection was also constant since the selected allele arose. However, selection coefficients can fluctuate over time, which provides the basis for future work on investigating the robustness of methods for detecting balancing selection under scenarios in which the strength of selection fluctuates or when selection is weak. Future work can use the framework developed here to construct methods for identifying balancing selection under models with more relaxed assumptions (e.g., see Barton and Etheridge [41] and Barton *et al*. [42] for potential models).

Recall that we chose a window size based on a fixed number of polymorphisms and substitutions. However, we could have chosen a window in a different way. For example, a window could have been chosen based on physical or genetic distance, rather than a fixed number of substitutions or polymorphisms. However, basing each likelihood calculation on a fixed number of substitutions or polymorphisms, rather than physical or genetic distance, enables each likelihood ratio to be based on the same number of terms, thereby letting the likelihood ratio depend on the density of polymorphisms vs. substitutions rather than the number of polymorphisms in the window. This contrasts other composite likelihood approaches for detecting positive selection (e.g., Nielsen *et al*., 2005 [26]), where the likelihood under the selection model approaches the likelihood under neutrality with increasing distance from the site under selection. This characteristic exhibited by these other composite likelihood approaches permits variable-size windows, so that at some point adding new terms to the likelihood ratio will not change its value. However, for our method, the likelihood under selection does not approach the likelihood under the background level of diversity (neutrality) with increasing distance from the putative site under selection, causing the value of the likelihood ratio to change by modifying the number of terms. If we chose a standard neutral model for the null hypothesis, then the likelihood under selection would approach the likelihood under the null model with increasing distance from the selected site. To attempt to account for demographic history, we have instead chosen to use the genome-wide level of diversity for the null hypothesis, which does not require that the likelihood under selection to approach the likelihood under the null hypothesis with increasing distance from the putative balanced polymorphism.

In our empirical analysis, we calculated the likelihood ratio (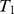 or 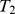) for numerous positions along the genome. We then ranked genes according to the largest likelihood ratio estimated between the annotated transcription start and stop of the gene. A consequence of ranking genes in this manner is that longer genes are more likely to be significant. However, because ancient balancing selection only impacts a relatively small region of the genome (in contrast to recent positive selection), the signal of ancient balancing selection could be masked if we instead assigned the average likelihood ratio as the score for a large gene. We therefore opted to assign the score for a gene as the highest likelihood ratio calculated within that gene.

Our methods have been shown to be substantially more powerful than HKA and Tajima's 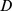 at detecting ancient balanced polymorphisms. However, a glance at Figures 3 and 4 indicates that under constant size and growth models our methods have little power to detect balanced polymorphisms at low false positive rates—a range that would be necessary to detect ancient balancing selection if it were rare. Hence, if balancing selection is relatively rare, then relying solely on statistics considered here to identify ancient balanced polymorphisms could possibly lead to an overabundance of false positives. Complementary evidence, such as considering patterns of linkage disequilibrium or trans-specific polymorphisms in candidate regions, should also be employed to hone in on true signals of ancient balancing selection.

Though we have shown that 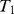 and 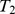 perform well under a population bottleneck and growth, they may be less robust to other forms of demographic model violations, such as population structure. Because population subdivision increases the time to coalescence and corresponding length of a genealogy, we expect higher levels of polymorphism across the genome. Under most assumptions, population subdivision affects the genome uniformly; it increases the level of background polymorphism and likely only slightly decreases the power of the new statistics. However, in some cases, such as an ancient admixture event (e.g., with Neanderthals [43] or Denisovans [44]), levels of variability may increase in only a few regions of the genome, increasing the mean coalescence time in these regions. Such regions may appear to have excess polymorphism relative to background levels and, hence, display false signals of balancing selection under the 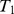 statistic. However, in non-African humans, introgressed regions typically have low population frequencies [43], [44], and, hence, it would be unlikely for polymorphic sites in these regions to harbor many introgressed alleles segregating at intermediate frequencies. Thus, the 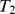 statistic, which explicitly utilizes allele frequency spectra information, would likely be able to distinguish these blocks of archaic admixture from regions of balancing selection. Further, as observed in other studies of natural selection [45], [46], increased robustness to confounding demographic processes can potentially be gained through the use of additional information. For example, population bottlenecks as well as gene flow can increase linkage disequilibrium [47], [48]. Therefore, knowledge about linkage disequilibrium in a region could aid in distinguishing population subdivision from long-term balancing selection.

Another concern when performing genomes scans for balancing selection is the possibility of false positives due to bioinformatical errors. For example, misalignment of sequence reads in duplicated regions may lead to falsely elevated levels of variability. In many cases, this problem can be alleviated by removing duplicated regions from analyses. However, a non-negligible portion of the human genome is not represented in standard reference sequences and, thus, there may be many unidentified paralogs in the genome. Fortunately, removing sites that deviate from Hardy-Weinberg equilibrium helps to alleviate these problems, because SNPs fixed between or segregating at high frequencies in one of two (or more) paralogous regions will have an excess of heterozygotes in combined short-read alignments. We applied a Hardy-Weinberg filter to all empirical data analyzed in this article. We note that deviations from Hardy-Weinberg equilibrium are expected under certain forms of balancing selection. In theory, a balancing selection signal could, therefore, be lost due to such filtering. However, we used a filtering cutoff of 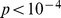 (see *Materials and Methods*). The strength of selection required to cause this type of deviation from Hardy-Weinberg equilibrium used in the filtering is extremely strong, and such selection would almost certainly have been detected using other methods. Well-established examples of balancing selection in the human genome, such as the selection affecting the HLA loci, are not lost because of filtering, and would generally not be easily detectable using deviations from Hardy-Weinberg as a test. Nonetheless, because phenomena other than balancing selection, such as bioinformatical errors or archaic admixture, could potentially lead to false signals of balancing selection, additional evidence should be obtained before definitively concluding that a site has been subjected to balancing selection.

One source of additional evidence of balancing selection is whether a signal lies within a region harboring a trans-specific polymorphism [7], [19] because it is unlikely to have a polymorphism segregating in each of a pair of closely-related species without selection maintaining the polymorphism. However, relying solely on evidence from trans-specific polymorphisms would miss many true signals of balancing selection that are not maintained as trans-specific polymorphisms. In addition, regions with bioinformatical errors (e.g., mapping errors) may give the same errors in both species, resulting in a false signal of a shared polymorphism between the pair of species. Nevertheless, the observation of a trans-specific polymorphism can provide convincing evidence of an ancient balanced polymorphism [7], [19]. Previous studies of selection have shown that combinations of statistics can be powerful tools when identifying genes under selection [15], [18], [49]. Hence, combining our methods with other summaries (e.g., linkage disequilibrium [45]–[48]) or information on trans-species polymorphisms [7], [19] will lead to increasingly effective approaches for detecting balancing selection.

The current approach taken by 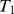 and 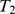 ignores higher order linkage disequilibria, in the sense that it ignores linkage disequilibrium between pairs of neutral markers and only considers correlations between neutral markers and the site under selection. However, incorporating higher order linkage information, such as employing tests based on haplotypes, could provide some advantage. For example, 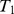 and 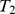 have little power to detect young balanced polymorphisms. However, the haplotype pattern around a young balanced polymorphism is likely to mimic that of an incomplete or partial selective sweep. Therefore, methods that use haplotype information (e.g., EHH [50], iHS [51], and 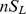 [52]), could provide a complementary and powerful approach to detecting recent balancing selection—a selective regime that the methods considered here have little power.

Another commonly-cited source of evidence for balancing selection is based on consideration of the topology and branch lengths of within-species haplotype trees. Under long-term balancing selection, the underlying genealogy (e.g., see Fig. S25) will be symmetric, with long basal branches separating a pair of allelic classes (*i.e.*, haplotypes containing one variant and haplotypes containing the other variant). However, the underlying genealogy for a linked neutral variant may differ substantially from that of the selected site. Around a balanced polymorphism, there will be a strong reduction of linkage disequilibrium, not unlike a recombination hotspot, because the long genealogy in the balanced polymorphism provides extra opportunities for recombination. Consequently, the signal of balancing selection will be narrow, and trees estimated from sites located in a window around the balanced polymorphism may fail to detect the presence of highly divergent haplotypes. The utility of within-species haplotype trees as a signature of long-term balancing selection is unclear, as the genealogy of the haplotype may not match the genealogy of the selected region. For example, Figure S26 shows that haplotype trees based on scenarios under balancing selection appear similar to those under neutrality, with the difference that external branches are slightly longer under balancing selection than under neutrality, which contrasts with the generally-held belief that basal branches should be long. These inferred long external branches are a product of estimating haplotype trees in recombining regions [53], which would likely be unavoidable in genomic regions under ancient balancing selection even if recombination events were undetected. As such, haplotype networks or trees built without explicitly accounting for recombination may not be powerful tools for identifying regions under balancing selection.

An assumption of the methods 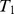 and 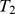 introduced in this article is that two allelic classes at a selected site are maintained for an infinitely long period of time at a constant equilibrium frequency by balancing selection. However, balancing selection is not restricted to act only on two stable allelic classes, and the equilibrium frequency can fluctuate with time and space. Examples of balancing selection that do not conform to our model assumptions are frequency-dependent selection [2], [3], fluctuating selection [2], [4], [5], selection maintained through segregation distortion [6], and selection maintaining more than two allelic classes [6]. Though these modes of balancing selection exhibit different evolutionary dynamics, they all lead to increased diversity around the site under selection, and therefore a decay in the density of polymorphisms with increasing genetic distance from the selected site. It is this information that 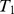 and 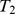 are employing to identify signatures of balancing selection, and though the dynamics of these modes of balancing selection violate the assumptions of our methods, it is likely that the statistics developed here could identify genomic signatures left behind by these selective scenarios provided selection was strong enough.

Within our scan, we identified a gene called *FANK1*, which is expressed during the transition from diploid to haploid states in meiosis [32], [33], is often identified as spermatogenesis-specific [32], [33], suppresses apoptosis [33], is imprinted [36], and exhibits evidence of segregation distortion (Fig. 8) [37]. These characteristics suggest that maintenance of polymorphism at *FANK1* results from segregation distortion, which can occur when the allele favored by distortion is associated with negative fitness effects, particularly if the negative effect is pronounced in the homozygous state (see p. 562–563 of Charlesworth and Charlesworth [6]; Úbeda and Haig [54]). The distorting allele will increase in frequency when rare because of the segregation distortion in heterozygotes. But when it becomes common, selection will act against it because it will more often occur in the homozygous state when rare. Under such a scenario, theoretical results suggest that it is possible for a distorter to spread through a population without reaching fixation, obtaining a frequency that permits the maintenance of a stable polymorphism (see p. 564 of Charlesworth and Charlesworth [6]). In addition, the inclusion of imprinting at such a locus further enchances the parameter space at which a polymorphism can be maintained [54].

The function of *FANK1* makes it a particularly good candidate for harboring alleles causing segregation distortion. It is expressed primarily during meiosis and inhibits apoptosis, which has previously been hypothesized to be associated with segregation distortion [55], [56]. A large proportion of sperm cells are eliminated by apoptosis, so allelic variants causing avoidance of apoptosis after meiosis could serve as segregation distorters. However, mutations that lead to avoidance of apoptosis may be associated with negative fitness effects, especially in the homozygous states, because they could lead to dysspermia or azoospermia. Apoptosis during spermatogenesis plays a critical role in maintaining the optimal relationship between the number of developing sperm cells and sertoli cells, which support developing sperm cells.

Though some of the sites identified in *FANK1* show marginal levels of segregation distortion, the region displaying the largest level of segregation distortion in the human genome is located 300 kb upstream of *FANK1* [37]. Further, a recent genome-wide association study for male fertility identified a significant SNP (rs9422913) located approximately 250 kb upstream of *FANK1* [57]. Even though these regions are quite distant from *FANK1*, if strong enough linkage exists with *FANK1*, then it is possible for a two-locus segregation distorter to spread within a population (p. 569 of Charlesworth and Charlesworth [6]). Hence the signals of segregation distortion [37] and fertility [57] displayed in these regions upstream of *FANK1* could be a result of an association with *FANK1*.

Thus, *FANK1* is an interesting candidate for further study of balancing selection. The association of segregation distortion and balancing selection has been empirically described in other species, e.g., *Caenorhabditis elegans* [58]. However, as it has not yet been documented in humans, *FANK1* may be the first example of a segregation distorter causing balancing selection in humans. However, further experiments would be needed to test the hypothesis of segregation distortion in *FANK1*.

In the last several years, there has been an accumulation of evidence against the pervasiveness of hard sweeps in some species, e.g., in humans [11]–[13]. Instead, other adaptive forces, such as balancing selection, could play an important role in shaping genetic variation across the genome. Interestingly, a recent theoretical study showed that a large proportion of adaptive mutations in diploids leads to heterozygote advantage [59], suggesting that much of the genome may be under balancing selection. If this intriguing prospect is true, then because our methods for detecting balancing selection are the most powerful that have been developed to date, they will be useful tools in uncovering the potentially many regions under balancing selection in humans and other species.

## Materials and Methods

### Estimating inter-species expected coalescence times

To compute the probabilities of polymorphism 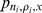 and substitution 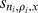 under our model, we must first obtain an estimate of the inter-species coalescent times 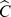. For the purposes of our simulation and empirical analyses, we introduce a basic estimate (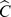) of the expected coalescence time between the ingroup and outgroup species. Consider a sample of 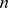 lineages (*i.e.*, 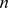 haploid individuals) from an ingroup species and one lineage from an outgroup species. For simplicity, assume that the ingroup species, outgroup species, and ancestral species from which the ingroup and outgroup diverged has an effective population size of 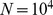 diploid individuals. Further, assume that the per-site per-generation mutation rate is 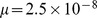 and that the total sequence length analyzed is 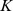. We estimate the expected coalescence time of all 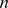 lineages in the ingroup species as 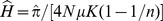, where 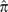 is the mean number of pairwise sequence differences and 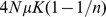 is the expected number of mutations for a sequence of length 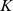 and 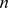 sampled lineages. Suppose that 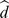 is the number of substitutions of fixed differences observed between the ingroup and outgroup species. Then we estimate the mean coalescence time between the ingroup and outgroup species by 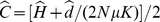.

### Application of the new test statistics to data

In the empirical analysis of human genomic data, we obtained values for the 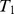 and 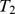 test statistics for a large number of positions spaced across the genome. From these values, we overlapped protein coding regions (or genes including exons and introns) with the positions in the genome that the test statistics were calculated at. We assigned the value of the test statistic for the gene as the maximal value of the test statistic for the positions that it overlapped. We then ranked the set of genes based on their scores to identify genes that are outliers. Note that we are not attempting to identify regions with statistical significance or a certain 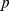-value threshold, but instead are looking for genes that may be outliers, and so the 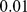, 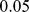, 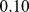, and 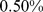 empirical cutoffs are not meant to represent a formal significance cutoff.

When applying the 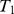 and 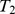 test statistics to simulated and empirical data, we do not estimate the rate of mutation 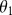 from 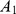 alleles to 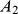 alleles or the rate of mutation 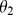 from 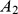 alleles to 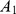 alleles at the selected site 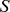, as defined in the Hudson-Darden-Kaplan model. We instead treat these rates as a constant, with 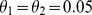 for the analyses in this article. The motivation is that, if these mutation rates did not exist, then the tree height would increase rapidly for small recombination rates. Our method assumes that a most recent common ancestor of the set of sampled alleles is reached more recently than the inter-species coalescence time 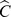 between the ingroup and outgroup species (*i.e.*, 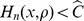 even for small 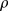). Simulation results (see *Evaluating the methods using simulations*) show that our new methods perform extremely well, even though we set the nuisance 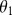 and 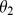 parameters to a constant value. To maximize of the equilibrium frequency 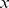 of the 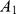 allele, we utilized the value of 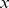, denoted by 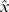, that maximized the composite likelihood under the model, by choosing 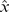 from values of 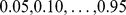.

### Simulation procedure to evaluate the performance of 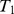 and 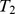


We applied 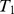 and 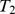 to data simulated under population divergence models, using parameters to mimic humans (ingroup) and chimpanzees (outgroup). The models that we simulated under are illustrated in Figure 2. For each of three models, we set each of the ingroup, outgroup, and ancestral population sizes to 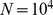 diploid individuals [60] and the divergence time between the ingroup and the outgroup species to 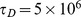 years ago [61]. We assumed a generation time of 20 years [62], a mutation rate of 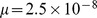 mutations per-nucleotide per-generation [62], a recombination rate of 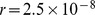 recombinations per-nucleotide per-generation, and a sequence length of 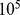 nucleotides. Assuming a per-generation selection coefficient 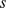, where 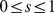, and a dominance parameter 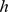, where 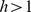, at time 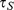, a selected allele arose and evolved under an overdominance model with 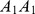 homozygotes having fitness 1, 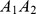 heterozygotes having fitness 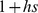, and 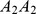 homozygotes having fitness 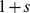. The formulation of this overdominance model is similar to that of [63] in which the fitness is 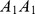 is 1, 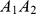 is 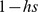, and 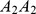 is 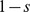. Under the Gillespie formulation, overdominance occurs when 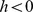, whereas it occurs when 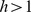 in our formulation. However, both result in an equilibrium frequency of 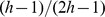. Simulations were performed using *mpop* [64], which was seeded with population-level chromosome data generated by the neutral coalescent simulator *ms* [65]. After the completion of each simulation, we sampled 18 chromosomes from the ingroup species and one chromosome from the outgroup species. For each set of parameter values, we simulated 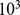 independent replicates. Ancestral alleles were called using the outgroup species, and so the called ancestral allele may not actually be the true ancestral allele. For each of the three demographic scenarios, we set 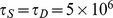 years ago. For the bottleneck model (Fig. 2*B*), we set the bottleneck population size to 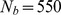 diploid individuals, the time at which the bottleneck began to 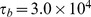 years ago, and the time at which the bottleneck ended to 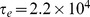 years ago [66], [67]. For the growth model (Fig. 2*C*), we set the expanded population size to 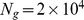 diploid individuals and the time at which the population began to grow to 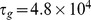 years ago [67]. Additionally, we considered a more ancient balanced polymorphism arising 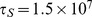 years ago and a more recent balanced polymorphism arising 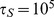 years ago. Because the forward simulations in *mpop* are computationally burdensome, we rescaled appropriate parameters by a factor of 10 such that the scaled population parameters remain the same, but the simulations are substantially sped up (by approximately a factor of 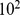). Note that scaling parameters in this way can somewhat affect the time to fixation of selected alleles. The distribution of false positive rates was generated by 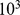 replicate neutral simulations from *mpop*, using the same parameters as the corresponding selection scenarios (including the rescaling factor) except without introducing a selected allele.

### Matching the density of polymorphic sites

In the current set of simulations, the bottleneck and growth models each produce a different density of polymorphisms (*i.e.*, number of segregating sites) than the constant size model. This section seeks to find an ancestral effective size for the growth and the bottleneck models such that the mean density of polymorphisms is close to that of the constant size model. We use eq. 1 in Marth *et al.* (2004) [68] to calculate the expected frequency spectrum under the bottleneck and growth models. The equation is 
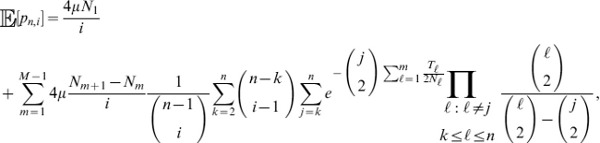
(15)where 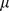 is the per-generation mutation rate, 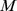 is the number of epochs, 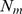 for 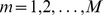, is the effective population size for epoch 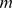, and 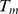 for 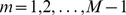, is the duration of time spent in epoch 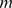. Our growth model contains two epochs, and so the appropriate version of the equation is when 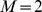. Setting the number of epochs to two, we the expected frequency spectrum under the growth model as 
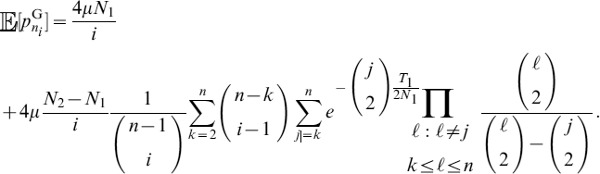
(16)Note that in our growth model, 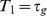, 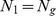, and 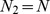. Denote the ratio of effective size during growth to the ancestral effective size as 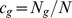. Then we can rewrite the equation as 
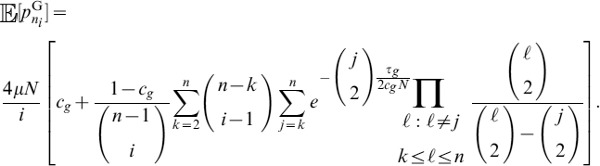
(17)


Consider an ancestral reference effective size 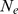 (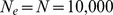 for the constant size model). Denote the expected number of segregating sites in a constant size model, conditional on effective size 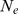 as 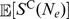. Conditional on this ancestral reference effective size 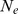, the expected site frequency spectrum under our growth model is 
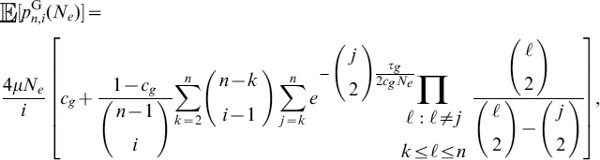
(18)where 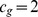 under our growth model. Therefore, the expected number of segregating sites conditional on reference effective size 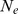 is 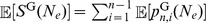. We obtain a growth model that produces the same density of polymorphic sites as our constant size model by choosing 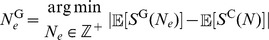


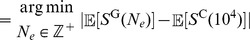


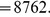
(19)


Our bottleneck model contains three epochs, and so the appropriate version of the equation is when 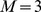. Setting the number of epochs to three, we the expected frequency spectrum under the bottleneck model as 
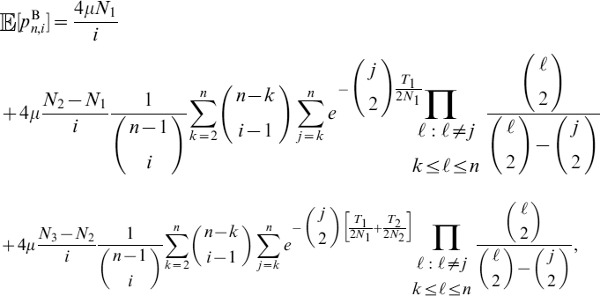
(20)


Note that in our bottleneck model, 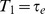, 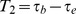, 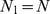, 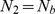, and 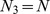. Denote the ratio of the effective size during the bottleneck to the ancestral effective size as 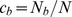. Then we can rewrite the equation as 
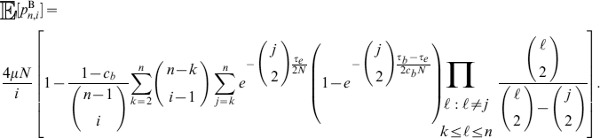
(21)


Conditional on this reference effective size, the expected site frequency spectrum under our bottleneck model is 
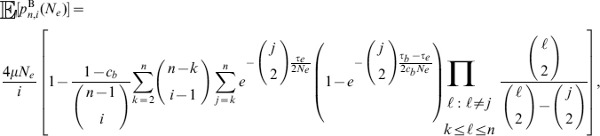
(22)where  under our bottleneck model. Therefore, the expected number of segregating sites conditional on reference effective size 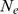 is 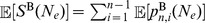. We obtain a bottleneck model that produces the same density of polymorphic sites as our constant size model by choosing 
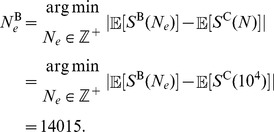
(23)


### Empirical dataset construction

We used data from nine European and nine African diploid genomes sequenced by Complete Genomics [69]. All individuals were unrelated [70], with the European individuals from the CEU population (NA06985, NA06994, NA07357, NA10851, NA12004, NA12889, NA12890, NA12891, NA12892) and the African individuals from the YRI population (NA18501, NA18502, NA18504, NA18505, NA18508, NA18517, NA19129, NA19238, NA19329). We used the genotype calls made by Complete Genomics that were found in the “masterVarBeta” files. We downloaded pairwise alignments between human reference hg18 and chimpanzee reference panTro2 from the UCSC Genome Browser at http://genome.ucsc.edu/. Sites with more than two distinct alleles across all Complete Genomics individuals as well as the hg18-panTro2 alignments, sites in the Complete Genomics data where one of the two alleles did not match the reference sequence, and sites that were within two nucleotides of structural variants called in any one of the Complete Genomics individuals were removed. In addition, combining all 54 unrelated individuals in the Complete Genomics dataset, sites that had a -value less than 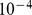 for a one-tailed Hardy-Weinberg test of excess heterozygotes [71] were excluded. We used the full set of 54 unrelated individuals, totalling 108 alleles, so that we would have sufficient power to detect Hardy-Weinberg departures due to excess heterozygotes. Sites flagged as departing from Hardy-Weinberg proportions in this set of 54 individuals were then filtered out in the smaller subsets of nine CEU and nine YRI individuals. It should be noted that under a scenario of heterozygote advantage, it is expected that we should observe an excess of heterozygous individuals at sites in the vicinity of the site under balancing selection. However, a major concern with sequencing data are mapping errors, and so the Hardy-Weinberg filter is necessary to reduce the confounding effects of regions with these bioinformatical artifacts. As a consequence, this filter may increase the chance that we miss certain regions that are under balancing selection in our scan. Finally, sites that were polymorphic in the Complete Genomics sample (*i.e.*, either CEU or YRI) and sites that contained a fixed difference between the Complete Genomics sample and the chimpanzee reference sequence were retained. As in the simulations, the ancestral allele was called using the chimpanzee outgroup, and so the called ancestral allele may not be the true ancestral allele. However, simulation results shows that our new methods perform well even when the ancestral allele is potentially misspecified. Further, it may be possible to account for ancestral allele misspecification by using multiple outgroups, or by statistically accounting for the misspecification [72].

To obtain recombination rates between pairs of sites, we used the sex-averaged pedigree-based human recombination map from deCODE Genetics [73]. We constructed recombination rates between all pairs of sites in the filtered Complete Genomics samples by linearly interpolating rates between adjacent sites within the sex-averaged maps.

## Supporting Information

Figure S1Performance of 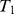, 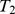, HKA, and Tajima's 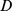 under the demographic models in Figure 2 with selection parameter 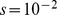 and dominance parameter 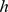. Each row represents a different 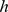 value. The first column is the divergence model in Figure 2*A*. The second column is the divergence model in Figure 2*B* with a recent bottleneck within the ingroup species. The third column is the divergence model in Figure 2*C* with recent population growth within the ingroup species.(PDF)

Figure S2Mean difference in the number of polymorphic sites for a model with 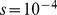 versus one with 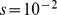 as a function of the distance from the site under balancing selection. Simulations were performed under the constant size divergence model in Figure 2*A* with selection parameter 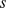, dominance parameter 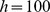, and time of selection 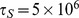 years ago. The mean difference in polymorphic sites is calculated for bins of size one kilobase and is plotted for 50 bins.(PDF)

Figure S3Performance of 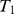, 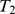, HKA, and Tajima's 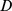 under the demographic models in Figure 2 with selection parameter 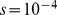 and dominance parameter 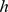. Each row represents a different 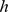 value. The first column is the divergence model in Figure 2*A*. The second column is the divergence model in Figure 2*B* with a recent bottleneck within the ingroup species. The third column is the divergence model in Figure 2*C* with recent population growth within the ingroup species.(PDF)

Figure S4Performance of 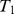, 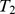, HKA, and Tajima's 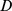 under the demographic models in Figure 2 with selection parameter 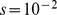 and dominance parameter 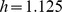. The first panel is the divergence model in Figure 2*A*. The second panel is the divergence model in Figure 2*B* with a recent bottleneck within the ingroup species. The third panel is the divergence model in Figure 2*C* with recent population growth within the ingroup species.(PDF)

Figure S5Performance of 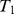 and 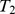 under the constant size divergence model in Figure 2*A* with no selected allele (neutrality). The first and second panels are scenarios in which we have erroneously over-estimated the recombination rate by two and one orders of magnitude, respectively (*i.e.*, we respectively assumed recombination rates of 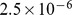 and 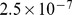 per base per generation when the simulations were performed using a rate of 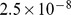 per base per generation). The third and fourth panels are scenarios in which we have erroneously under-estimated the recombination rate by one and two orders of magnitude, respectively (*i.e.*, we respectively assumed recombination rates of 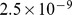 and 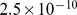 per base per generation when the simulations were performed using a rate of 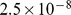 per base per generation). False positive rate is determined by neutral simulations under a model with recombination rate of 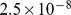 per base per generation.(PDF)

Figure S6Performance of 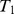 and 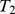 under the constant size divergence model in Figure 2*A* with selection parameter 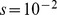, dominance parameter 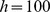 or 1.5, and time of selection 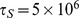 years ago. The first and second columns are scenarios in which we have erroneously over-estimated the recombination rate by two and one orders of magnitude, respectively (*i.e.*, we respectively assumed recombination rates of 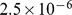 and 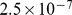 per base per generation when the simulations were performed using a rate of 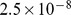 per base per generation). The third and fourth columns are scenarios in which we have erroneously under-estimated the recombination rate by one and two orders of magnitude, respectively (*i.e.*, we respectively assumed recombination rates of 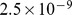 and 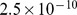 per base per generation when the simulations were performed using a rate of 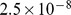 per base per generation). False positive rate is determined by neutral simulations under a model with recombination rate of 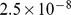 per base per generation.(PDF)

Figure S7Demographic models used in simulations in which a selected allele arises prior to the split a pair of species. (*A*) Divergence model. Model parameters are a diploid effective population size 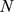, divergence time 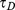 of the ingroup and outgroup species, and the time 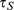 when the selected allele arises. (*B*) Divergence model with a recent bottleneck within the ingroup species. Additional model parameters are the diploid effective population size 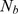 during the bottleneck, the time 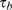 when the bottleneck began, and the time 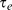 when the bottleneck ended. (*C*) Divergence model with recent population growth within the ingroup species. Additional model parameters are the current diploid effective population size 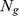 after recent growth and the time 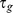 when the growth occurred.(PDF)

Figure S8Performance of 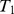, 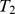, HKA, and Tajima's 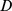 under the demographic models in Figure S7 with selection parameter 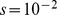 and dominance parameter 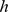. Each row represents a different 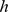 value. The first column is the divergence model in Figure S7 *A*. The second column is the divergence model in Figure S7 *B* with a recent bottleneck within the ingroup species. The third column is the divergence model in Figure S7 *C* with recent population growth within the ingroup species.(PDF)

Figure S9Performance of 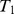, 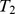, HKA, and Tajima's 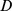 under the demographic models in Figure S7 with selection parameter 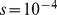 and dominance parameter 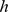. Each row represents a different 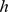 value. The first column is the divergence model in Figure S7 *A*. The second column is the divergence model in Figure S7 *B* with a recent bottleneck within the ingroup species. The third column is the divergence model in Figure S7 *C* with recent population growth within the ingroup species.(PDF)

Figure S10Performance of 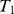, 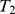, HKA, and Tajima's 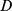 under the demographic models in Figure 2 with selection parameter 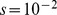, dominance parameter 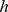, and time of selection 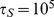. The first column is the divergence model in Figure 2*A*. The second column is the divergence model in Figure 2*B* with a recent bottleneck within the ingroup species. The third column is the divergence model in Figure 2*C* with recent population growth within the ingroup species.(PDF)

Figure S11Performance of 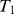, 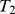, HKA, and Tajima's 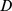 under the demographic models in Figure 2 with selection parameter 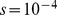, dominance parameter 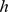, and time of selection 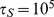. The first column is the divergence model in Figure 2*A*. The second column is the divergence model in Figure 2*B* with a recent bottleneck within the ingroup species. The third column is the divergence model in Figure 2*C* with recent population growth within the ingroup species.(PDF)

Figure S12Performance of 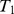, 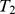, HKA, and Tajima's 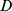 under the demographic models in Figure 2 with selection parameter 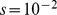 and dominance parameter 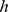. Each row represents a different 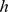 value. The population sizes for these demographic histories have been scaled so that they produce the same number of segregating sites as a constant size population with diploid effective size 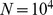 individuals. The first column is the divergence model in Figure 2*B* with a recent bottleneck within the ingroup species. The second column is the divergence model in Figure 2*C* with recent population growth within the ingroup species.(PDF)

Figure S13Performance of 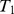, 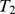, HKA, and Tajima's 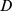 under the demographic models in Figure 2 with selection parameter 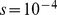 and dominance parameter 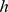. Each row represents a different 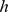 value. The population sizes for these demographic histories have been scaled so that they produce the same number of segregating sites as a constant size population with diploid effective size 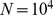 individuals. The first column is the divergence model in Figure 2*B* with a recent bottleneck within the ingroup species. The second column is the divergence model in Figure 2*C* with recent population growth within the ingroup species.(PDF)

Figure S14Performance of 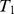, 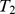, HKA, and Tajima's 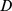 under the demographic models in Figure S7 with selection parameter 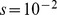 and dominance parameter 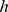. Each row represents a different 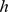 value. The population sizes for these demographic histories have been scaled so that they produce the same number of segregating sites as a constant size population with diploid effective size 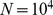 individuals. The first column is the divergence model in Figure S7 *B* with a recent bottleneck within the ingroup species. The second column is the divergence model in Figure S7 *C* with recent population growth within the ingroup species.(PDF)

Figure S15Performance of 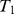, 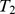, HKA, and Tajima's 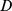 under the demographic models in Figure S7 with selection parameter 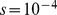 and dominance parameter 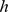. Each row represents a different 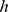 value. The population sizes for these demographic histories have been scaled so that they produce the same number of segregating sites as a constant size population with diploid effective size 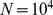 individuals. The first column is the divergence model in Figure S7 *B* with a recent bottleneck within the ingroup species. The second column is the divergence model in Figure S7 *C* with recent population growth within the ingroup species.(PDF)

Figure S16Performance of 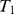, 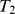, HKA, and Tajima's 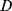 under the demographic models in Figure 2 with selection parameter 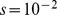, and dominance parameter 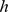, and time of selection 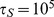. Each row represents a different 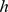 value. The population sizes for these demographic histories have been scaled so that they produce the same number of segregating sites as a constant size population with diploid effective size 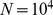 individuals. The first column is the divergence model in Figure 2*B* with a recent bottleneck within the ingroup species. The second column is the divergence model in Figure 2*C* with recent population growth within the ingroup species.(PDF)

Figure S17Performance of 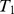, 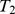, HKA, and Tajima's 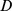 under the demographic models in Figure 2 with selection parameter 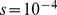, and dominance parameter 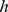, and time of selection 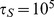. Each row represents a different 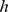 value. The population sizes for these demographic histories have been scaled so that they produce the same number of segregating sites as a constant size population with diploid effective size 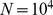 individuals. The first column is the divergence model in Figure 2*B* with a recent bottleneck within the ingroup species. The second column is the divergence model in Figure 2*C* with recent population growth within the ingroup species.(PDF)

Figure S18Manhattan plot of genome-wide scans for balancing selection within the CEU population using the 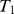 test statistic. From bottom to top, the horizontal dotted gray lines indicate the 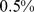, 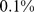, 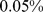, and 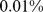 empirical cutoffs, respectively. The 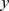-axis is truncated at log composite likelihood ratio of zero.(PDF)

Figure S19Manhattan plot of genome-wide scans for balancing selection within the YRI population using the 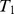 test statistic. From bottom to top, the horizontal dotted gray lines indicate the 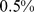, 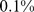, 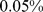, and 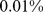 empirical cutoffs, respectively. The 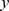-axis is truncated at log composite likelihood ratio of zero.(PDF)

Figure S20Manhattan plot of genome-wide scans for balancing selection within the CEU population using the 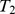 test statistic. From bottom to top, the horizontal dotted gray lines indicate the 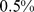, 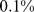, 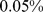, and 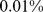 empirical cutoffs, respectively. The 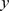-axis is truncated at log composite likelihood ratio of zero.(PDF)

Figure S21Manhattan plot of genome-wide scans for balancing selection within the YRI population using the 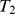 test statistic. From bottom to top, the horizontal dotted gray lines indicate the 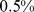, 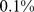, 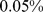, and 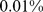 empirical cutoffs, respectively. The 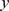-axis is truncated at log composite likelihood ratio of zero.(PDF)

Figure S22Signals of balancing selection within the HLA region for the CEU (blue) and YRI (orange) populations using the 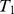 test statistic. From bottom to top, the horizontal dotted gray lines indicate the 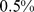, 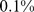, 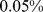, and 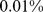 empirical cutoffs, respectively.(PDF)

Figure S23Signal of balancing selection at the *FANK1* gene for the CEU (blue) and YRI (orange) populations using the 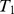 test statistic. From bottom to top, the horizontal dotted gray lines indicate the 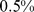, 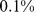, 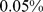, and 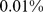 empirical cutoffs, respectively. SNPs (rsIDs) correspond to markers showing significant levels of transmission distortion within the Meyer *et al.* study.(PDF)

Figure S24Signal of balancing selection at the *FANK1* gene for the CEU (blue) and YRI (orange) populations when removing either 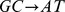 transitions or all transitions. SNPs (rsIDs) correspond to markers showing significant levels of transmission distortion within the Meyer *et al.* study.(PDF)

Figure S25 Genealogy at the site of balancing selection.
(PDF)

Figure S26Haplotype trees based on randomly sampling 18 haplotypes without replacement from a random simulation under the model in Figure S7 *A*. Trees were generated using UPGMA applied to a distance matrix of the proportion of nucleotide differences between each pair of haplotypes. The 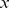-kilobase (kb) window represents a region that is 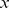 kb in length and is centered in the middle of the haplotype.(PDF)

Table S1Top 100 signals in the CEU population using the 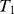 test statistic.(PDF)

Table S2Top 100 signals in the YRI population using the 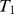 test statistic.(PDF)

Table S3Top 100 signals in the CEU population using the 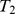 test statistic.(PDF)

Table S4Top 100 signals in the YRI population using the 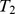 test statistic.(PDF)

Table S5GO process analysis of top 100 signals, when compared to all signals, from CEU population using the 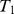 test statistic.(PDF)

Table S6GO process analysis of top 100 signals, when compared to all signals, from YRI population using the 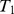 test statistic.(PDF)

Table S7GO process analysis of top 100 signals, when compared to all signals, from CEU population using the 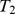 test statistic.(PDF)

Table S8GO process analysis of top 100 signals, when compared to all signals, from YRI population using the 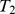 test statistic.(PDF)

Table S9GO function analysis of top 100 signals, when compared to all signals, from CEU population using the 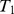 test statistic.(PDF)

Table S10GO function analysis of top 100 signals, when compared to all signals, from YRI population using the 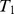 test statistic.(PDF)

Table S11GO function analysis of top 100 signals, when compared to all signals, from CEU population using the 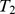 test statistic.(PDF)

Table S12GO component analysis of top 100 signals, when compared to all signals, from CEU population using the 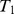 test statistic.(PDF)

Table S13GO component analysis of top 100 signals, when compared to all signals, from YRI population using the 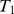 test statistic.(PDF)

Table S14GO component analysis of top 100 signals, when compared to all signals, from CEU population using the 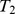 test statistic.(PDF)

Table S15GO component analysis of top 100 signals, when compared to all signals, from YRI population using the 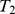 test statistic.(PDF)

Table S16GO process analysis of ranked signals from CEU population using the 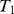 test statistic.(PDF)

Table S17GO process analysis of ranked signals from YRI population using the 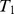 test statistic.(PDF)

Table S18GO process analysis of ranked signals from CEU population using the 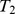 test statistic.(PDF)

Table S19GO process analysis of ranked signals from YRI population using the 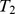 test statistic.(PDF)

Table S20GO function analysis of ranked signals from CEU population using the 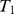 test statistic.(PDF)

Table S21GO function analysis of ranked signals from YRI population using the 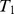 test statistic.(PDF)

Table S22GO function analysis of ranked signals from CEU population using the 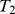 test statistic.(PDF)

Table S23GO function analysis of ranked signals from YRI population using the 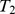 test statistic.(PDF)

Table S24GO component analysis of ranked signals from CEU population using the 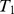 test statistic.(PDF)

Table S25GO component analysis of ranked signals from YRI population using the 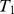 test statistic.(PDF)

Table S26GO component analysis of ranked signals from CEU population using the 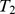 test statistic.(PDF)

Table S27GO component analysis of ranked signals from YRI population using the 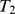 test statistic.(PDF)

## References

[1] Fisher RA (1922) On the dominance ratio. Proc Roy Soc Edin 42: 321–341.

[2] Andrés AM (2011) Balancing selection in the human genome. In: Encyclopedia of Life Sciences, Chichester: John Wiley and Sons.

[3] Wilson DS, Turelli M (1986) Stable underdominance and the evolutionary invasion of empty niches. Am Nat 127: 835–850.

[4] Levene H (1953) Genetic equilibrium when more than one ecological niche is available. Am Nat 83: 331–333.

[5] Nagylaki T (1975) Polymorphisms in cyclically varying environments. Heredity 35: 67–74. 1058845 10.1038/hdy.1975.67

[6] Charlesworth B, Charlesworth D (2010) Elements of evolutionary genetics. Greenwood Village, CO: Roberts and Company Publishers.

[7] Ségurel L, Thompson EE, Flutre T, Lovstad J, Venkat A, et al (2012) The ABO blood group is a trans-species polymorphism in primates. Proc Natl Acad Sci USA 109: 18493–18498. 23091028 10.1073/pnas.1210603109 PMC3494955

[8] Klein J, Satta Y, O'hUigín C (1993) The molecular descent of the major histocompatibility complex. Annu Rev Immunol 11: 269–95. 8476562 10.1146/annurev.iy.11.040193.001413

[9] Klein J, Sato A, Nagl S, O'hUigín C (1998) Molecular trans-species polymorphism. Annu Rev Ecol Syst 29: 1–21.

[10] Klein J, Sato A, Nikolaidis N (2007) MHC, TSP, and the origin of species: from immunogenetics to evlutionary genetics. Annu Rev Genet 41: 281–304. 18076327 10.1146/annurev.genet.41.110306.130137

[11] Hernandez RD, Kelley JL, Elyashiv E, Melton SC, Auton A, et al (2011) Classic selective sweeps were rare in recent human evolution. Science 331: 920–924. 21330547 10.1126/science.1198878 PMC3669691

[12] Lohmueller KE, Albrechtsen A, Li Y, Y KS, Korneliussen T, et al (2011) Natural selection affects multiple aspects of genetic variation at putatively neutral sites across the human genome. PLoS Genet 7: e1002326. 22022285 10.1371/journal.pgen.1002326 PMC3192825

[13] Granka JM, Henn BM, Gignoux CR, Kidd JM, Bustamante CD, et al (2012) Limited evidence for classic selective sweeps in African populations. Genetics 92: 1049–64 doi:10.1534/genetics.112.144071 22960214 10.1534/genetics.112.144071 PMC3522151

[14] Bubb KL, Bovee D, Buckley D, Haugen E, Kibukawa M, et al (2006) Scan of human genome reveals no new loci under ancient balancing selection. Genetics 173: 2165–2177. 16751668 10.1534/genetics.106.055715 PMC1569689

[15] Andrés AM, Hubisz MJ, Indap A, Torgerson DG, Degenhardt JD, et al (2009) Targets of balancing selection in the human genome. Mol Biol Evol 26: 2755–2764. 19713326 10.1093/molbev/msp190 PMC2782326

[16] Hudson RR, Kreitman M, Aguadé M (1987) A test of neutral marker evolution based on nucleotide data. Genetics 116: 153–159. 3110004 10.1093/genetics/116.1.153 PMC1203113

[17] Tajima F (1989) Statistical method for testing the neutral mutation hypothesis by DNA polymorphism. Genetics 123: 585–595. 2513255 10.1093/genetics/123.3.585 PMC1203831

[18] Innan H (2006) Modified Hudson-Kreitman-Aguadé test and two-dimensional evaluation of neutrality tests. Genetics 173: 1725–1733. 16624905 10.1534/genetics.106.056242 PMC1526661

[19] Leffler EM, Gao Z, Pfeifer S, Ségurel L, Auton A, et al (2013) Multiple instances of ancient balancing selection shared between humans and chimpanzees. Science 339: 1578–1582. 23413192 10.1126/science.1234070 PMC3612375

[20] Kaplan NL, Darden T, Hudson RR (1988) The coalescent proces in models with selection. Genetics 120: 819–829. 3066685 10.1093/genetics/120.3.819 PMC1203559

[21] Hudson RR, Kaplan NL (1988) The coalescent process in models with selection and recombination. Genetics 120: 831–840. 3147214 10.1093/genetics/120.3.831 PMC1203560

[22] Hudson RR (2001) Two-locus sampling distributions and their application. Genetics 159: 1805–1817. 11779816 10.1093/genetics/159.4.1805 PMC1461925

[23] Kim Y, Stephan W (2002) Detecting a local signature of genetic hitchhiking along a recombining chromosome. Genetics 160: 765–777. 11861577 10.1093/genetics/160.2.765 PMC1461968

[24] Kim Y, Nielsen R (2004) Linkage disequilibrium as a signature of selective sweeps. Genetics 167: 1513–1524. 15280259 10.1534/genetics.103.025387 PMC1470945

[25] Jensen JD, Kim Y, DuMont VB, Aquadro CF, Bustamante CD (2005) Distinguishing between selective sweeps and demography using DNA polymorphism data. Genetics 170: 1401–1410. 15911584 10.1534/genetics.104.038224 PMC1451184

[26] Nielsen R, Williamson S, Kim Y, Hubisz MJ, Clark AG, et al (2005) Genomic scans for selective sweeps using SNP data. Genome Res 15: 1566–1575. 16251466 10.1101/gr.4252305 PMC1310644

[27] Nielsen R, Hubsz MJ, Hellmann I, Torgerson D, Andrés AM, et al (2009) Darwinian and demographic forces affecting human protein coding genes. Genome Res 19: 838–849. 19279335 10.1101/gr.088336.108 PMC2675972

[28] Chen H, Patterson N, Reich D (2010) Population differentiation as a test for selective sweeps. Genome Res 20: 393–402. 20086244 10.1101/gr.100545.109 PMC2840981

[29] Thomas LH (1949) Elliptic problems in linear difference equations over a network. New York: Watson Sci. Comput. Lab. Rept., Columbia University.

[30] Takahata N, Nei M (1990) Allelic genealogy under overdominant and frequency-dependent selection and polymorphism of major histocompatibility loci. Genetics 124: 967–978. 2323559 10.1093/genetics/124.4.967 PMC1203987

[31] Hedrick PW (2002) Pathogen resistance and geneic variation at MHC loci. Evolution 56: 1902–1908. 12449477 10.1111/j.0014-3820.2002.tb00116.x

[32] Zheng Z, Zheng H, Yan W (2007) *Fank1* is a testis-specific gene encoding a nuclear protein exclusively expressed during the transition from meiotic to the haploid phase of spermatogenesis. Gene Expr Patterns 7: 777–783. 17604233 10.1016/j.modgep.2007.05.005

[33] Wang H, Song W, Hu T, Zhang N, Miao S, et al (2011) Fank1 interacts with Jab1 and regulates cell apoptosis via the AP-1 pathway. Cell Mol Life Sci 68: 2129–2139. 20978819 10.1007/s00018-010-0559-4 PMC11114715

[34] Hwang KC, Park SY, Park SP, Lim JH, Cui XS, et al (2005) Specific maternal transcripts in bovie oocytes and cleavaged embryos: identification with novel DDRT-PCR methods. Mol Reprod Dev 71: 275–283. 15803458 10.1002/mrd.20282

[35] Zuccotti M, Merico V, Sacchi L, Bellone M (2008) Brink R T C nd Bellazzi, (2008) et al Maternal Oct-4 is a potential key regulator of the developmental compentence of mouse oocytes. BMC Dev Biol 8: 97. 18837968 10.1186/1471-213X-8-97 PMC2576189

[36] Li Y, Zhu J, Tian G, Li N, Li Q, et al (2010) The DNA methylome of human peripheral blood mononuclear cells. PLoS Biol 8: e1000533. 21085693 10.1371/journal.pbio.1000533 PMC2976721

[37] Meyer WK, Arbeithuber B, Ober C, Ebner T, Tiemann-Boege I, et al (2012) Evaluating the evidence for transmission distortion in human pedigress. Genetics 191: 215–232. 22377632 10.1534/genetics.112.139576 PMC3338262

[38] Akey JM, Eberle MA, Rieder MJ, Carlson CS, Shriver MD, et al (2004) Population history and natural selection shape patterns of genetic variation in 132 genes. PLoS Biol 2: e286. 15361935 10.1371/journal.pbio.0020286 PMC515367

[39] Eden E, Lipson D, Yogev S, Yakhini Z (2007) Discovering motifs in ranked lists of DNA sequences. PLoS Comput Biol 3: e39. 17381235 10.1371/journal.pcbi.0030039 PMC1829477

[40] Eden E, Navon R, Steinfeld I, Lipson D, Yakhini Z (2009) GOrilla: a tool for discovery and visualization of enriched GO terms in ranked gene lists. BMC Bioinformatics 10: 48. 19192299 10.1186/1471-2105-10-48 PMC2644678

[41] Barton NH, Etheridge AM (2004) The effect of selection on genealogies. Genetics 166: 1115–1131. 15020491 10.1093/genetics/166.2.1115 PMC1470728

[42] Barton NH, Etheridge AM, Sturm AK (2004) Coalescence in a random background. Ann Appl Probab 14: 754–785.

[43] Green RE, Krause J, Briggs AW, Marici T, Stenzel U, et al (2010) A draft sequence of the Neandertal genome. Science 328: 710–722. 20448178 10.1126/science.1188021 PMC5100745

[44] Reich D, Green RE, Kircher M, Krause J, Patterson N, et al (2010) Genetic history of an archaic hominin group from Denisova Cave in Siberia. Nature 468: 1053–1060. 21179161 10.1038/nature09710 PMC4306417

[45] Jensen JD, Thornton KR, Bustamante CD, Aquadro CF (2007) On the utility of linkage disequilibrium as a statistic for identifying targets of positive selection in nonequilibrium populations. Genetics 176: 2371–2379. 17565955 10.1534/genetics.106.069450 PMC1950638

[46] Pavlidis P, Jensen JD, Stephan W (2010) Searching for footprints of positive selection in whole-genome SNP data from nonequilibrium populations. Genetics 185: 907–922. 20407129 10.1534/genetics.110.116459 PMC2907208

[47] Plagnol V, Wall JD (2006) Possible ancestral structure in human populations. PLoS Genet 2: 972–979. 10.1371/journal.pgen.0020105 PMC1523253 16895447

[48] Slatkin M (2008) Linkage disequilibrium - understanding gthe evolutionary past and mapping the medical future. Nat Rev Genet 9: 477–485. 18427557 10.1038/nrg2361 PMC5124487

[49] Grossman SR, Shylakhter I, Karlsson EK, Byrne EH, Morales S, et al (2010) A composite of multiple signals distinguishes causal variants in regions of positive selection. Science 327: 883–886. 20056855 10.1126/science.1183863

[50] Sabeti PC, Reich DE, Higgins JM, Levine HZP, Richter DJ, et al (2002) Detecting recent positive selection in the human genome from haplotype structure. Nature 419: 832–837. 12397357 10.1038/nature01140

[51] Voight BF, Kudravalli S, Wen X, Pritchard JK (2006) A map of recent positive selection in the human genome. PLoS Biol 4: e72. 16494531 10.1371/journal.pbio.0040072 PMC1382018

[52] Ferrer-Admetlla A, Liang M, Korneliussen T, Nielsen R (2014) On detecting incomplete soft or hard selective sweeps using haplotype structure. Mol Biol Evol 31: 1059–65 DOI: 10.1093/molbev/msu077 24554778 10.1093/molbev/msu077 PMC3995338

[53] Schierup MH, Hein J (2000) Consequences of recombination on traditional phylogenetic analysis. Genetics 156: 879–891. 11014833 10.1093/genetics/156.2.879 PMC1461297

[54] Úbeda F, Haig D (2004) Sex-specific meiotic drive and selection at an imprinted locus. Genetics 167: 2083–2095. 15342542 10.1534/genetics.103.021303 PMC1470996

[55] Nielsen R, Bustamante CD, Clark AG, Glanowski S, Stackton TB, et al (2005) A scan for positively selected genes in the genomes of humans and chimpanzees. PLoS Biol 3: 976–985. 10.1371/journal.pbio.0030170 PMC1088278 15869325

[56] da Fonseca RR, Kosiol C, Vinař T, Siepel A, Nielsen R (2010) Positive selection on apoptosis related genes. FEBS Lett 584: 469–476. 20026333 10.1016/j.febslet.2009.12.022

[57] Kosova G, Scott NM, Niederberger C, Prins GS, Ober C (2012) Genome-wide association study identifies candidate genes for male fertility traits in humans. Am J Hum Genet 90: 950–961. 22633400 10.1016/j.ajhg.2012.04.016 PMC3370277

[58] Seidel HS, Rockman MV, Kruglyak L (2008) Widespread gentic incompatibility in *C. elegans* maintained by balancing selection. Science 319: 589–594. 18187622 10.1126/science.1151107 PMC2421010

[59] Sellis D, Callahan BJ, Petrov DA, Messer PW (2012) Heterozygote advantage as a natural consequence of adaptation in diploids. Proc Natl Acad Sci USA 108: 20666–20671. 22143780 10.1073/pnas.1114573108 PMC3251125

[60] Takahata N, Satta Y, Klein J (1995) Divergence time and population size in the lineage leading to modern humans. Theor Popul Biol 48: 198–221. 7482371 10.1006/tpbi.1995.1026

[61] Kumar S, Filipski A, Swama V, Walker A, Hedges SB (2005) Placing confidence limits on the molecular age of the human-chimpanzee divergence. Proc Natl Acad Sci USA 102: 18842–18847. 16365310 10.1073/pnas.0509585102 PMC1316887

[62] Nachman MW, Crowell SL (2000) Estimate of the mutation rate per nucleotide in humans. Genetics 156: 297–304. 10978293 10.1093/genetics/156.1.297 PMC1461236

[63] Gillespie J (2004) Population genetics: a concise guide. Baltimore, MD: Johns Hopkins University Press, 2nd edition.

[64] Pickrell JK, Coop G, Novembre J, Kudravalli S, Li JZ, et al (2009) Signals of recent positive selection in a worldwide sample of human populations. Genome Res 19: 826–837. 19307593 10.1101/gr.087577.108 PMC2675971

[65] Hudson RR (2002) Generating samples under a Wright-Fisher neutral model. Bioinformatics 18: 337–338. 11847089 10.1093/bioinformatics/18.2.337

[66] Lohmueller KE, Bustamante CD, Clark AG (2009) Methods for human demographic inference using halptype patterns from genomewide single-nucleotide polymorphism data. Genetics 182: 217–231. 19255370 10.1534/genetics.108.099275 PMC2674818

[67] Lohmueller KE, Bustamante CD, Clark AG (2011) Detectig directional selection in the presence of recent admixture in African-Americans. Genetics 187: 823–835. 21196524 10.1534/genetics.110.122739 PMC3063676

[68] Marth GT, Czabarka E, Murvai J, Sherry ST (2004) The allele frequency spectrum in genome-wide human variation data reveals signals of differential demographic history in three large world populations. Genetics 166: 351–372. 15020430 10.1534/genetics.166.1.351 PMC1470693

[69] Drmanac R, Sparks AB, Callow MJ, Halpern AL, Burns NL, et al (2009) Human genome sequencing using unchained base reads on self-assembling DNA nanoarrays. Science 327: 78–81. 19892942 10.1126/science.1181498

[70] Pemberton TJ, Wang C, Li JZ, Rosenberg NA (2010) Inference of unexpected genetic relatedness among individuals in HapMap Phase III. Am J Hum Genet 87: 457–464. 20869033 10.1016/j.ajhg.2010.08.014 PMC2948801

[71] Wigginton JE, Cutler DJ, Abecasis GR (2005) A note on exact tests of Hardy-Weinberg equilibrium. Am J Hum Genet 76: 887–893. 15789306 10.1086/429864 PMC1199378

[72] Hernandez RD, Williamson SH, Bustamante CD (2007) Context dependence, ancestral misidentification, and spurious signatures of natural selection. Mol Biol Evol 28: 1792–1800. 17545186 10.1093/molbev/msm108

[73] Kong A, Thorleifsson G, Gudbjartsson DF, Masson G, Sigurdsson A, et al (2010) Fine-scale recombination rate differences between sexes, populations and individuals. Nature 467: 1099–1103. 20981099 10.1038/nature09525

