# Supplementary figures and images for: A Model-Based Approach for Identifying Signatures of Ancient Balancing Selection in Genetic Data
Source: PLoS Genet. 2014 Aug 21;10(8):e1004561. doi: 10.1371/journal.pgen.1004561 (PMC4140648; doi:10.1371/journal.pgen.1004561)

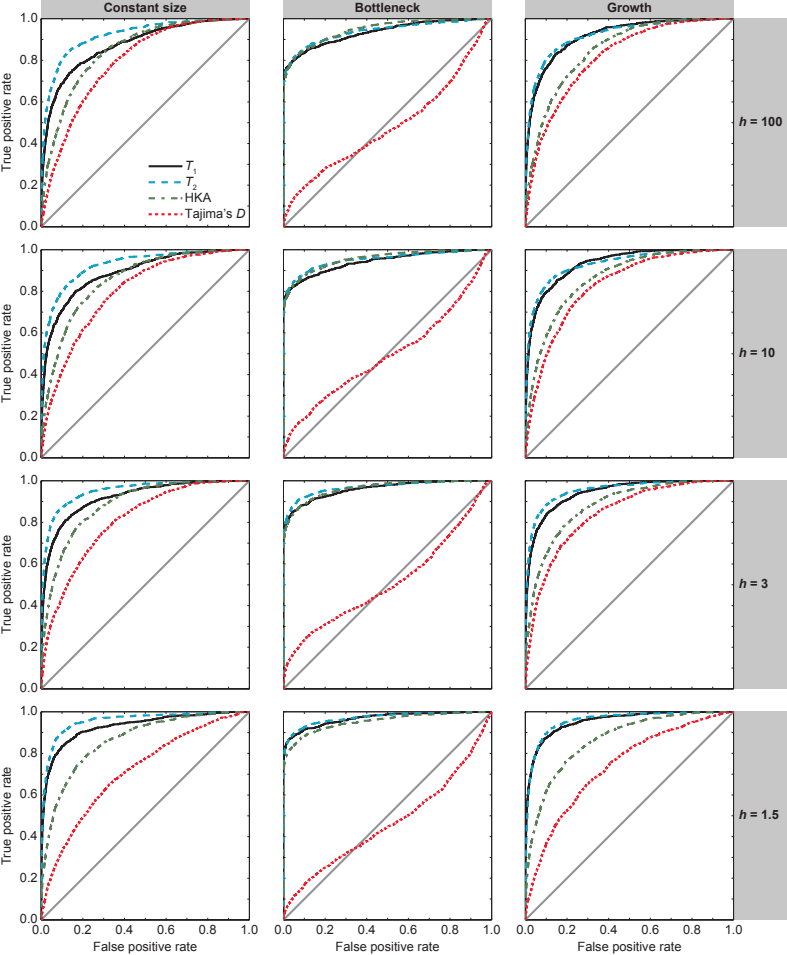

Supplement: Figure S1 — Performance of , , HKA, and Tajima's under the demographic models in Figure 2 with selection parameter and dominance parameter . Each row represents a different value. The first column is the divergence model in Figure 2A . The second column is the divergence model in Figure 2B with a recent bottleneck within the ingroup species. The third column is the divergence model in Figure 2C with recent population growth within the ingroup species. (PDF) [file pgen.1004561.s001.pdf]

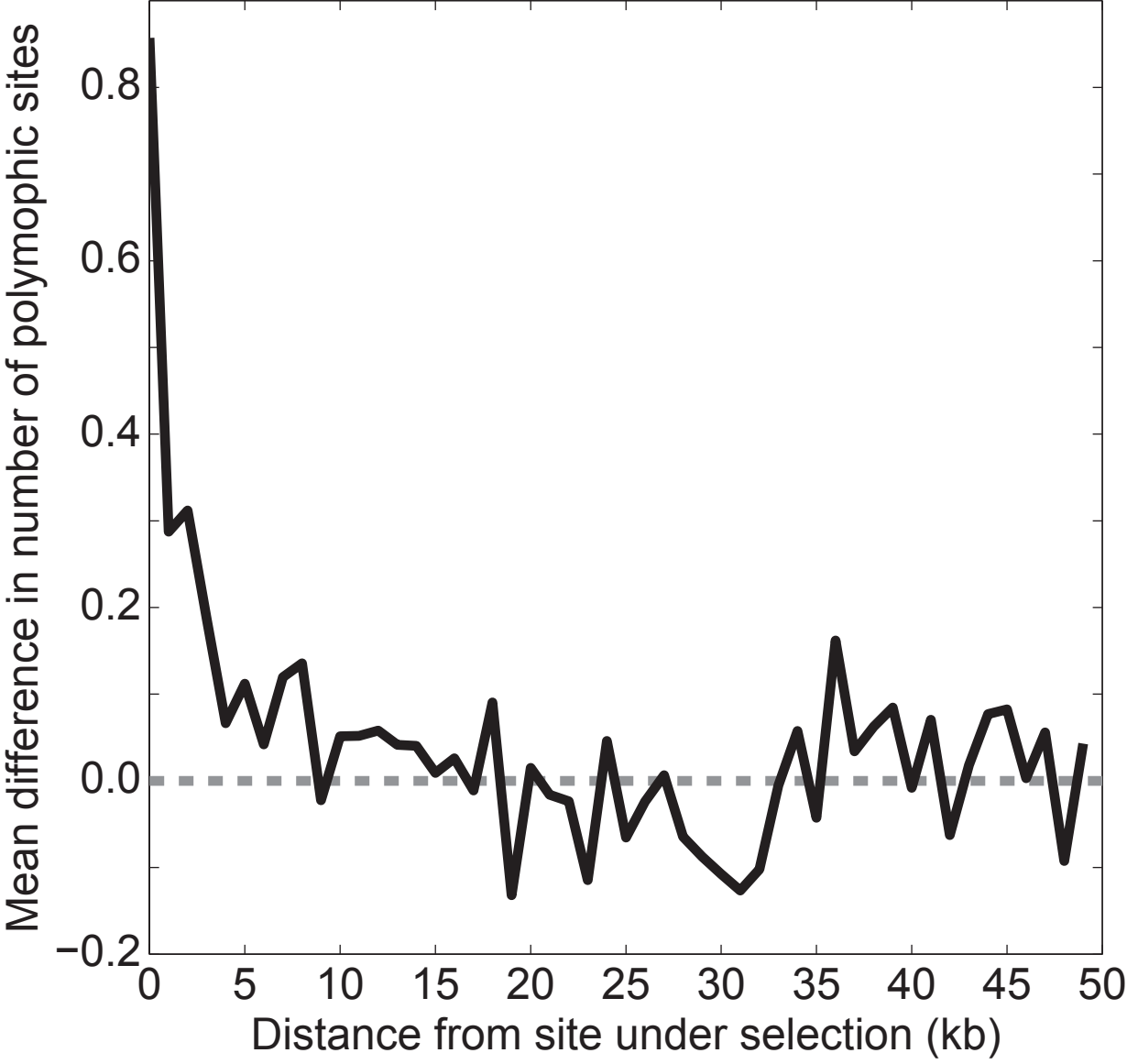

Supplement: Figure S2 — Mean difference in the number of polymorphic sites for a model with versus one with as a function of the distance from the site under balancing selection. Simulations were performed under the constant size divergence model in Figure 2A with selection parameter , dominance parameter , and time of selection years ago. The mean difference in polymorphic sites is calculated for bins of size one kilobase and is plotted for 50 bins. (PDF) [file pgen.1004561.s002.pdf]

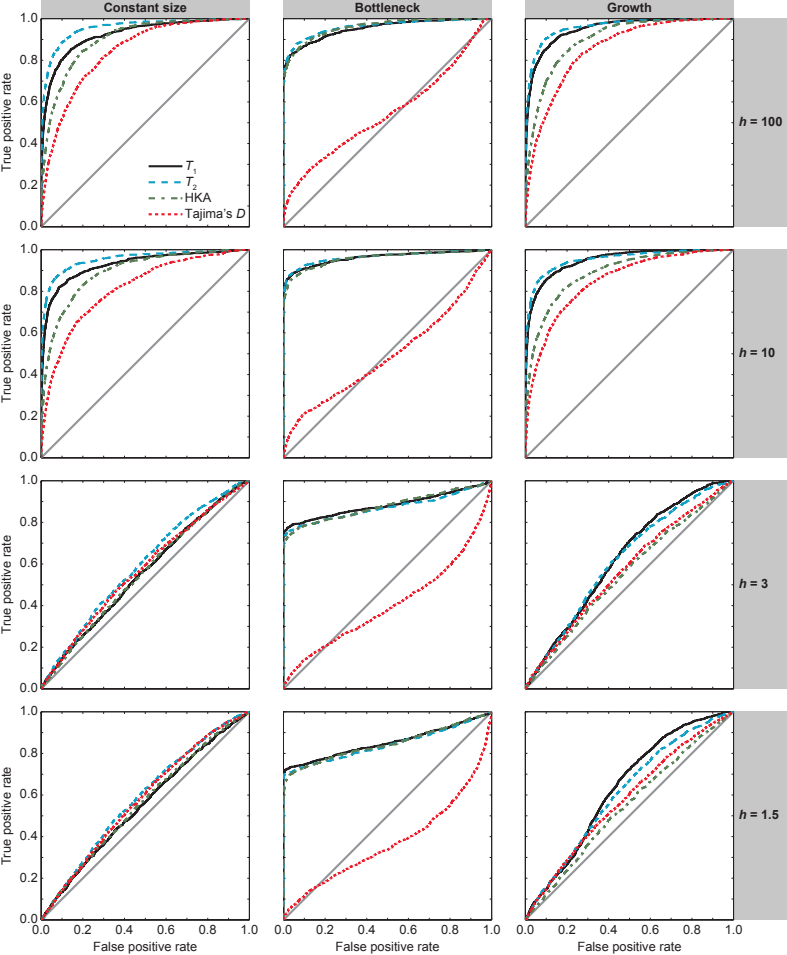

Supplement: Figure S3 — Performance of , , HKA, and Tajima's under the demographic models in Figure 2 with selection parameter and dominance parameter . Each row represents a different value. The first column is the divergence model in Figure 2A . The second column is the divergence model in Figure 2B with a recent bottleneck within the ingroup species. The third column is the divergence model in Figure 2C with recent population growth within the ingroup species. (PDF) [file pgen.1004561.s003.pdf]

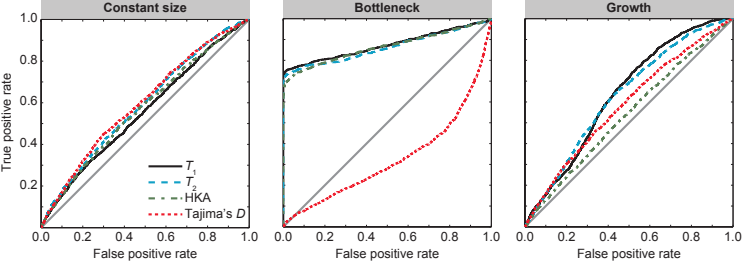

Supplement: Figure S4 — Performance of , , HKA, and Tajima's under the demographic models in Figure 2 with selection parameter and dominance parameter . The first panel is the divergence model in Figure 2A . The second panel is the divergence model in Figure 2B with a recent bottleneck within the ingroup species. The third panel is the divergence model in Figure 2C with recent population growth within the ingroup species. (PDF) [file pgen.1004561.s004.pdf]

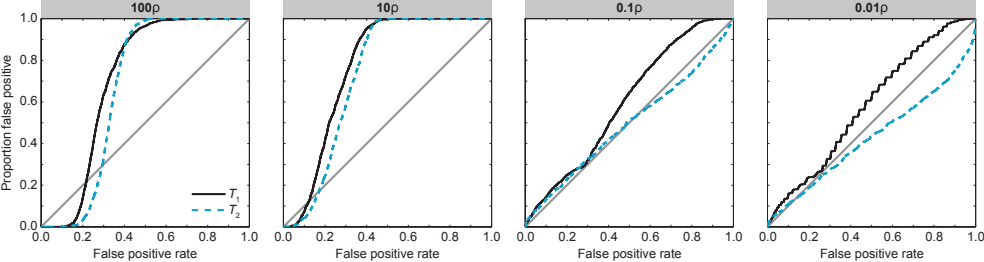

Supplement: Figure S5 — Performance of and under the constant size divergence model in Figure 2A with no selected allele (neutrality). The first and second panels are scenarios in which we have erroneously over-estimated the recombination rate by two and one orders of magnitude, respectively (i.e., we respectively assumed recombination rates of and per base per generation when the simulations were performed using a rate of per base per generation). The third and fourth panels are scenarios in which we have erroneously under-estimated the recombination rate by one and two orders of magnitude, respectively (i.e., we respectively assumed recombination rates of and per base per generation when the simulations were performed using a rate of per base per generation). False positive rate is determined by neutral simulations under a model with recombination rate of per base per generation. (PDF) [file pgen.1004561.s005.pdf]

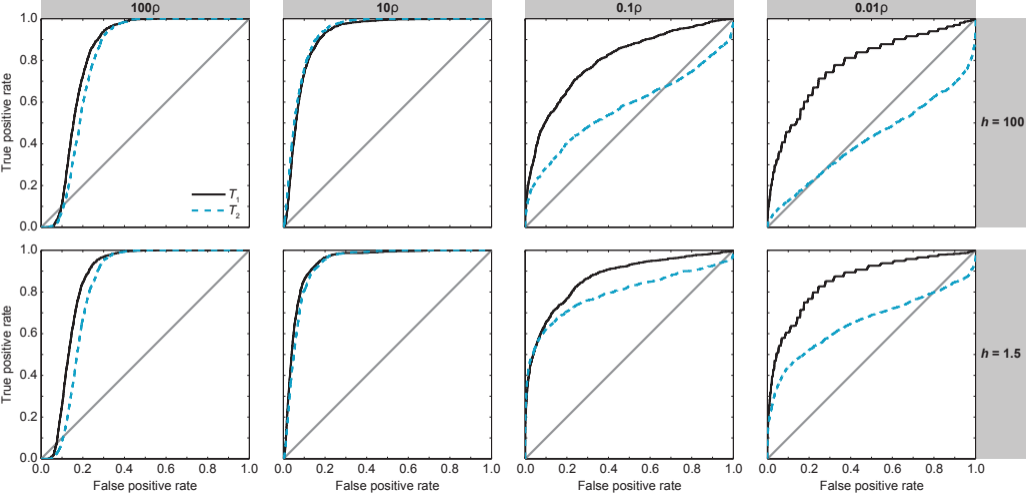

Supplement: Figure S6 — Performance of and under the constant size divergence model in Figure 2A with selection parameter , dominance parameter or 1.5, and time of selection years ago. The first and second columns are scenarios in which we have erroneously over-estimated the recombination rate by two and one orders of magnitude, respectively (i.e., we respectively assumed recombination rates of and per base per generation when the simulations were performed using a rate of per base per generation). The third and fourth columns are scenarios in which we have erroneously under-estimated the recombination rate by one and two orders of magnitude, respectively (i.e., we respectively assumed recombination rates of and per base per generation when the simulations were performed using a rate of per base per generation). False positive rate is determined by neutral simulations under a model with recombination rate of per base per generation. (PDF) [file pgen.1004561.s006.pdf]

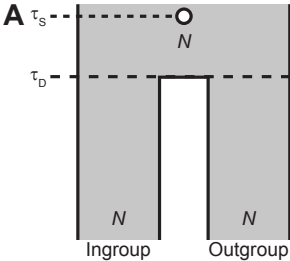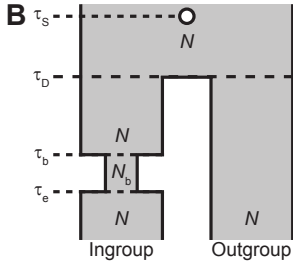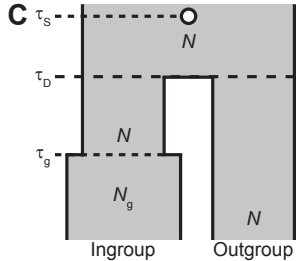

Supplement: Figure S7 — Demographic models used in simulations in which a selected allele arises prior to the split a pair of species. (A) Divergence model. Model parameters are a diploid effective population size , divergence time of the ingroup and outgroup species, and the time when the selected allele arises. (B) Divergence model with a recent bottleneck within the ingroup species. Additional model parameters are the diploid effective population size during the bottleneck, the time when the bottleneck began, and the time when the bottleneck ended. (C) Divergence model with recent population growth within the ingroup species. Additional model parameters are the current diploid effective population size after recent growth and the time when the growth occurred. (PDF) [file pgen.1004561.s007.pdf]

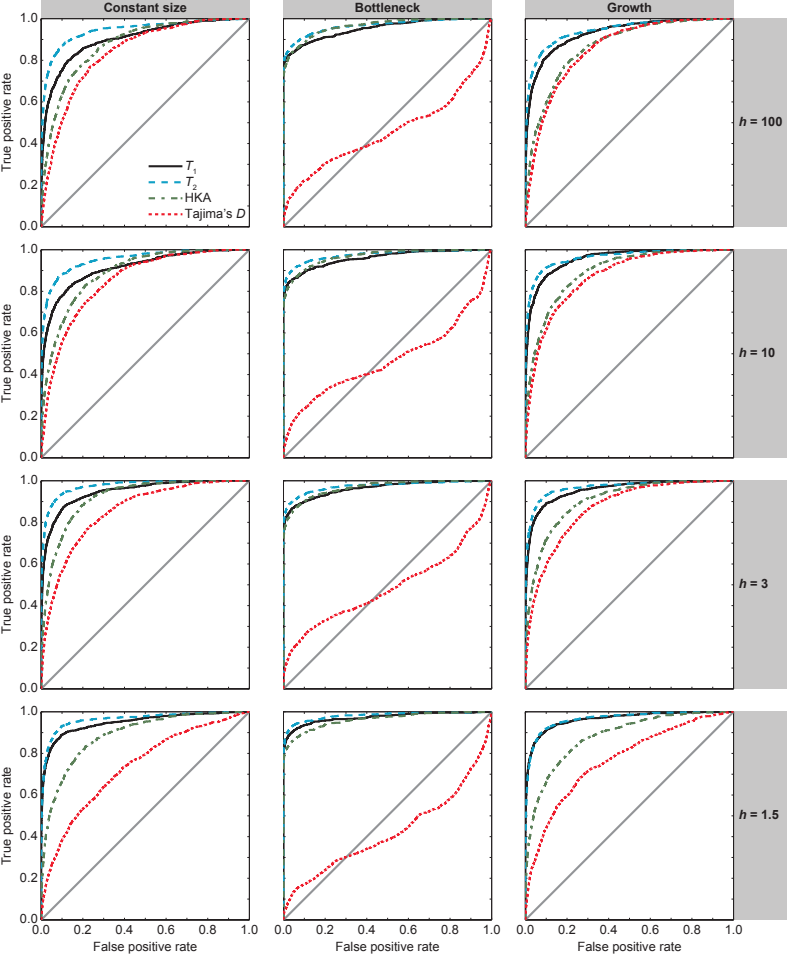

Supplement: Figure S8 — Performance of , , HKA, and Tajima's under the demographic models in Figure S7 with selection parameter and dominance parameter . Each row represents a different value. The first column is the divergence model in Figure S7 A. The second column is the divergence model in Figure S7 B with a recent bottleneck within the ingroup species. The third column is the divergence model in Figure S7 C with recent population growth within the ingroup species. (PDF) [file pgen.1004561.s008.pdf]

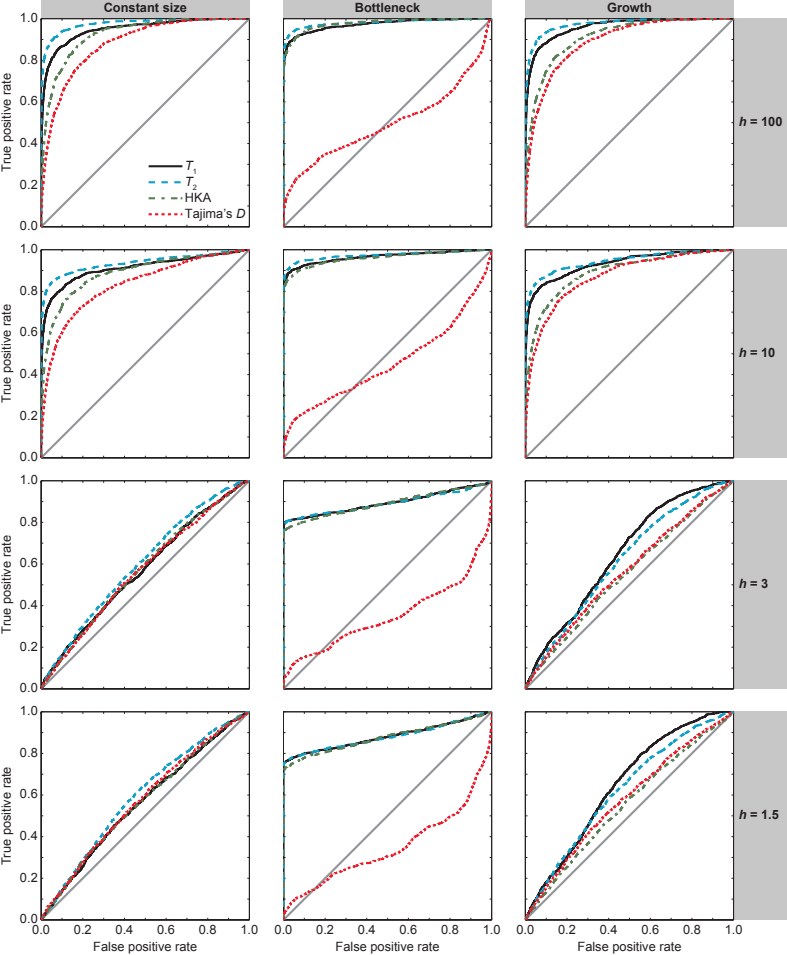

Supplement: Figure S9 — Performance of , , HKA, and Tajima's under the demographic models in Figure S7 with selection parameter and dominance parameter . Each row represents a different value. The first column is the divergence model in Figure S7 A. The second column is the divergence model in Figure S7 B with a recent bottleneck within the ingroup species. The third column is the divergence model in Figure S7 C with recent population growth within the ingroup species. (PDF) [file pgen.1004561.s009.pdf]

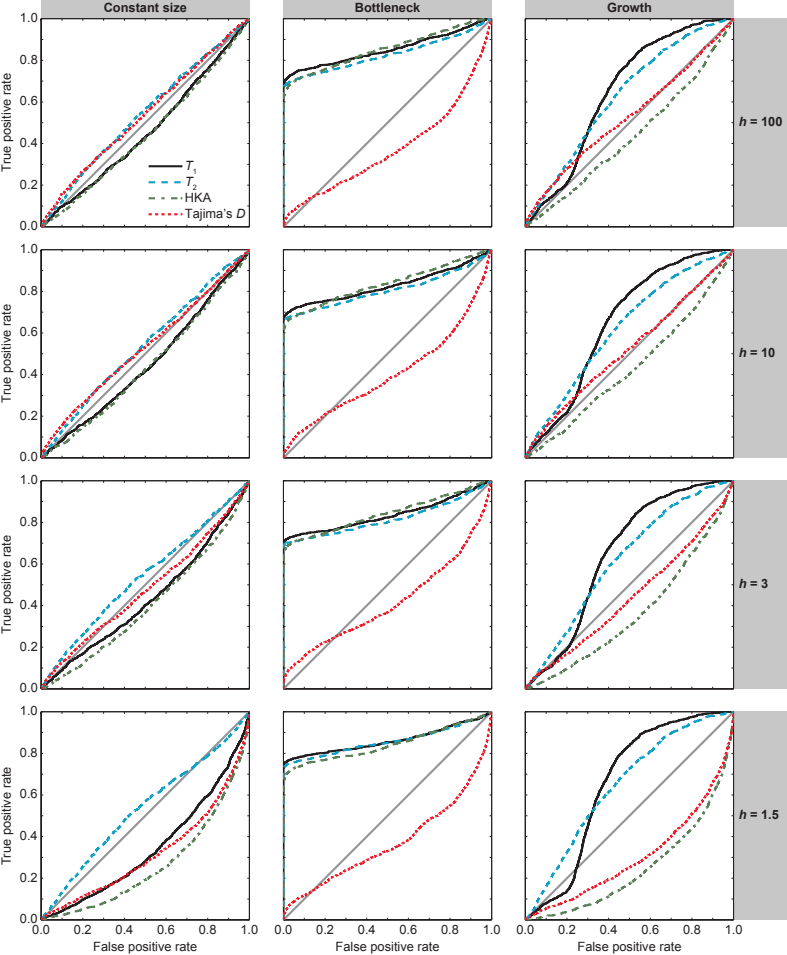

Supplement: Figure S10 — Performance of , , HKA, and Tajima's under the demographic models in Figure 2 with selection parameter , dominance parameter , and time of selection . The first column is the divergence model in Figure 2A . The second column is the divergence model in Figure 2B with a recent bottleneck within the ingroup species. The third column is the divergence model in Figure 2C with recent population growth within the ingroup species. (PDF) [file pgen.1004561.s010.pdf]

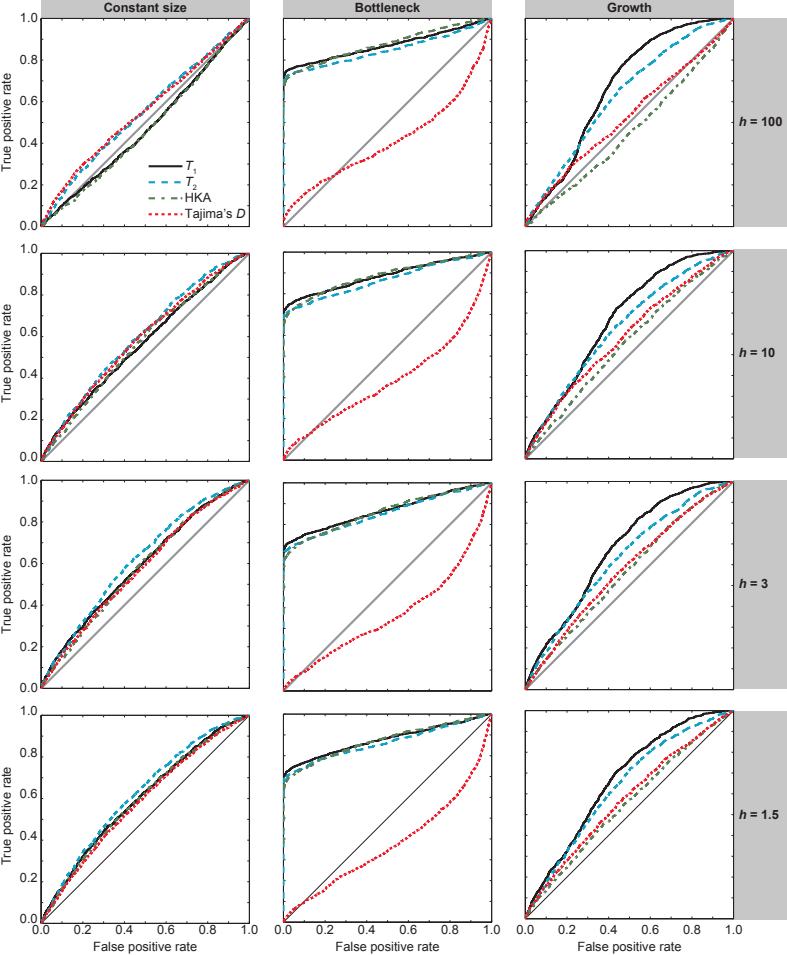

Supplement: Figure S11 — Performance of , , HKA, and Tajima's under the demographic models in Figure 2 with selection parameter , dominance parameter , and time of selection . The first column is the divergence model in Figure 2A . The second column is the divergence model in Figure 2B with a recent bottleneck within the ingroup species. The third column is the divergence model in Figure 2C with recent population growth within the ingroup species. (PDF) [file pgen.1004561.s011.pdf]

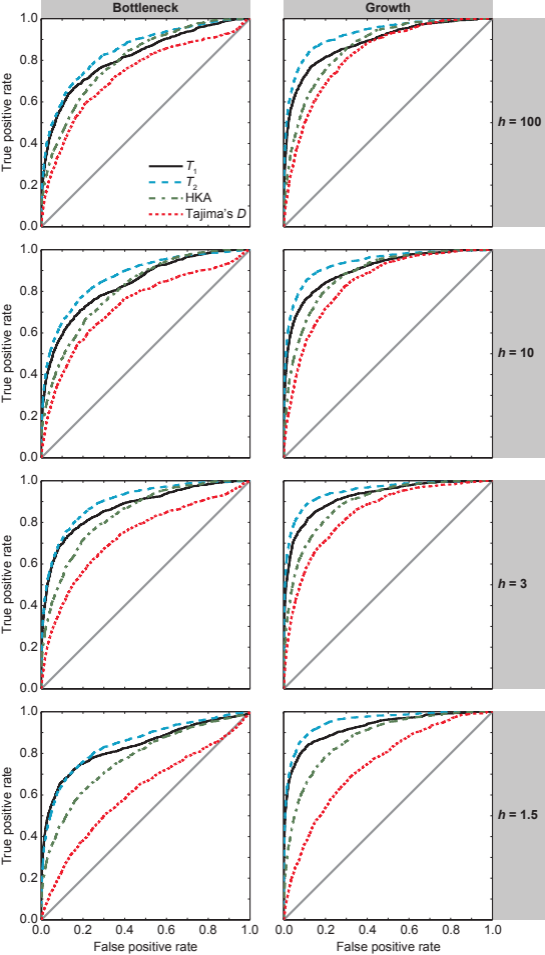

Supplement: Figure S12 — Performance of , , HKA, and Tajima's under the demographic models in Figure 2 with selection parameter and dominance parameter . Each row represents a different value. The population sizes for these demographic histories have been scaled so that they produce the same number of segregating sites as a constant size population with diploid effective size individuals. The first column is the divergence model in Figure 2B with a recent bottleneck within the ingroup species. The second column is the divergence model in Figure 2C with recent population growth within the ingroup species. (PDF) [file pgen.1004561.s012.pdf]

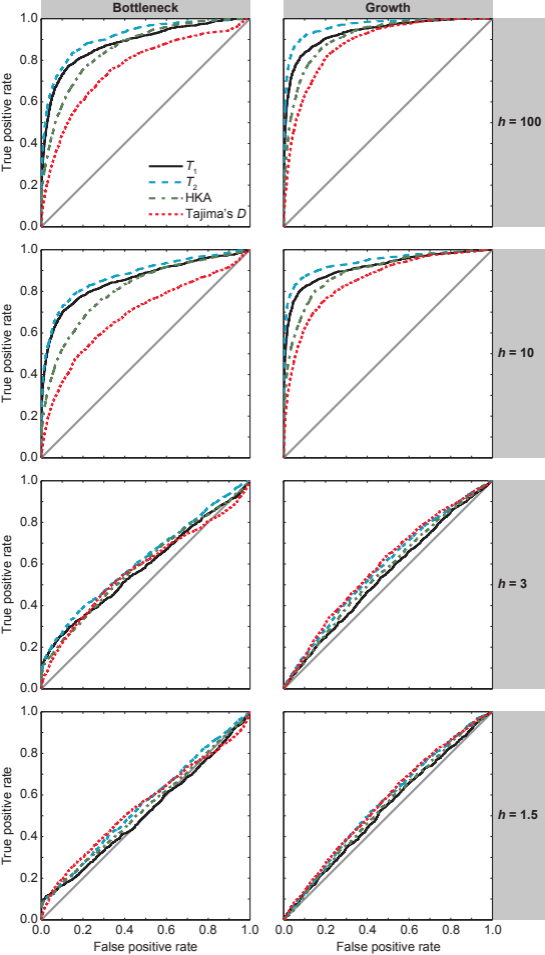

Supplement: Figure S13 — Performance of , , HKA, and Tajima's under the demographic models in Figure 2 with selection parameter and dominance parameter . Each row represents a different value. The population sizes for these demographic histories have been scaled so that they produce the same number of segregating sites as a constant size population with diploid effective size individuals. The first column is the divergence model in Figure 2B with a recent bottleneck within the ingroup species. The second column is the divergence model in Figure 2C with recent population growth within the ingroup species. (PDF) [file pgen.1004561.s013.pdf]

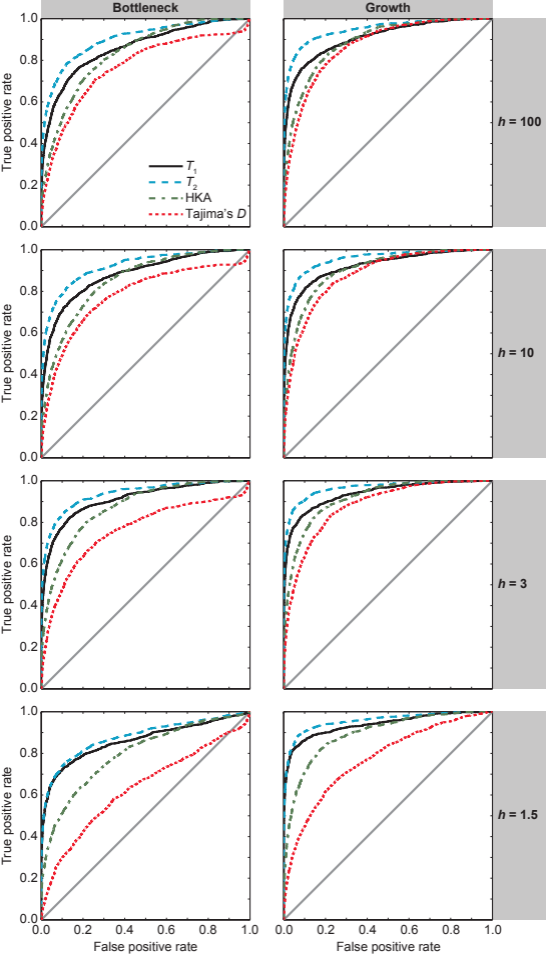

Supplement: Figure S14 — Performance of , , HKA, and Tajima's under the demographic models in Figure S7 with selection parameter and dominance parameter . Each row represents a different value. The population sizes for these demographic histories have been scaled so that they produce the same number of segregating sites as a constant size population with diploid effective size individuals. The first column is the divergence model in Figure S7 B with a recent bottleneck within the ingroup species. The second column is the divergence model in Figure S7 C with recent population growth within the ingroup species. (PDF) [file pgen.1004561.s014.pdf]

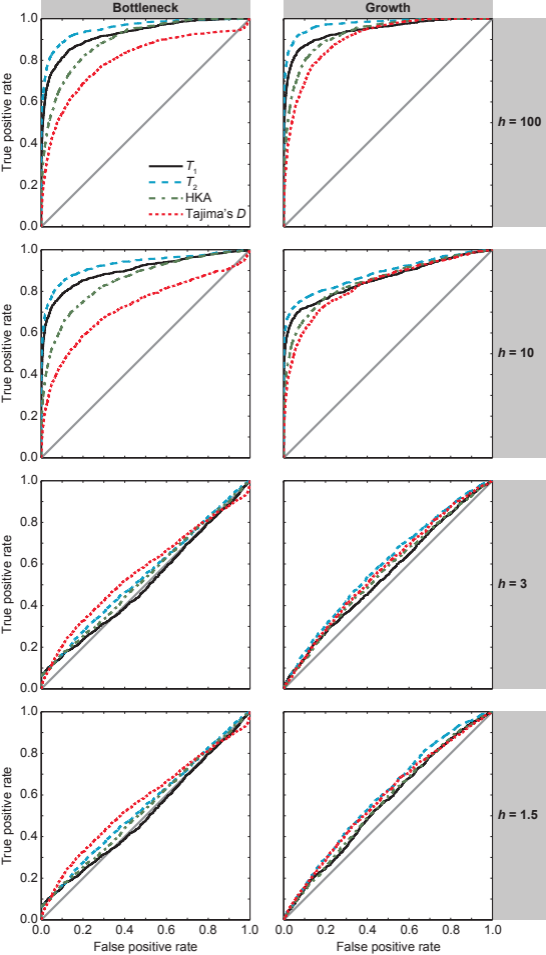

Supplement: Figure S15 — Performance of , , HKA, and Tajima's under the demographic models in Figure S7 with selection parameter and dominance parameter . Each row represents a different value. The population sizes for these demographic histories have been scaled so that they produce the same number of segregating sites as a constant size population with diploid effective size individuals. The first column is the divergence model in Figure S7 B with a recent bottleneck within the ingroup species. The second column is the divergence model in Figure S7 C with recent population growth within the ingroup species. (PDF) [file pgen.1004561.s015.pdf]

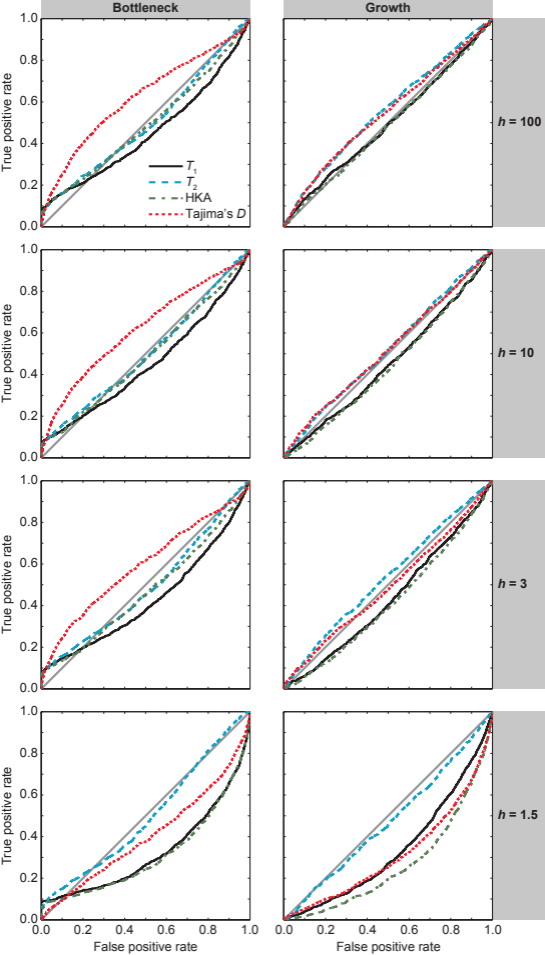

Supplement: Figure S16 — Performance of , , HKA, and Tajima's under the demographic models in Figure 2 with selection parameter , and dominance parameter , and time of selection . Each row represents a different value. The population sizes for these demographic histories have been scaled so that they produce the same number of segregating sites as a constant size population with diploid effective size individuals. The first column is the divergence model in Figure 2B with a recent bottleneck within the ingroup species. The second column is the divergence model in Figure 2C with recent population growth within the ingroup species. (PDF) [file pgen.1004561.s016.pdf]

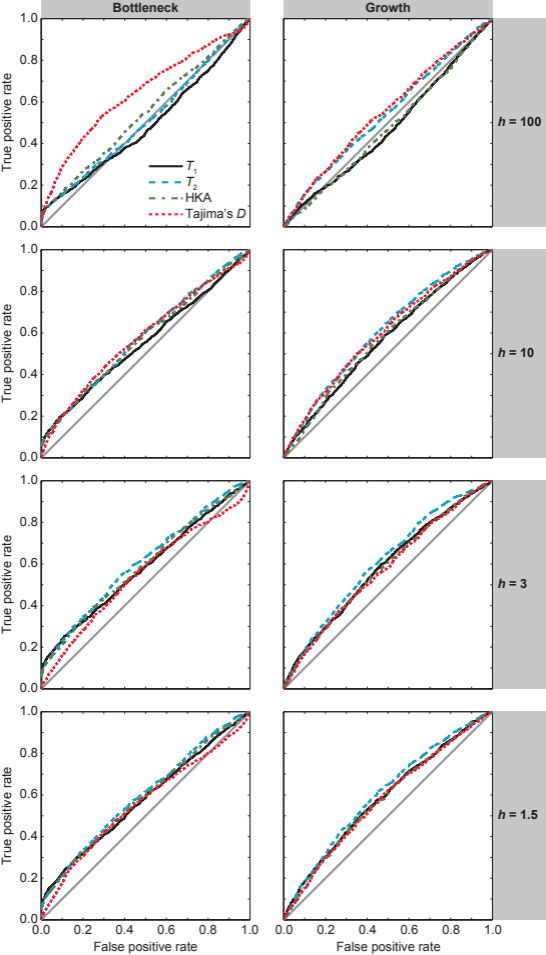

Supplement: Figure S17 — Performance of , , HKA, and Tajima's under the demographic models in Figure 2 with selection parameter , and dominance parameter , and time of selection . Each row represents a different value. The population sizes for these demographic histories have been scaled so that they produce the same number of segregating sites as a constant size population with diploid effective size individuals. The first column is the divergence model in Figure 2B with a recent bottleneck within the ingroup species. The second column is the divergence model in Figure 2C with recent population growth within the ingroup species. (PDF) [file pgen.1004561.s017.pdf]

Log composite likelihood ratio ( $T_1$ )

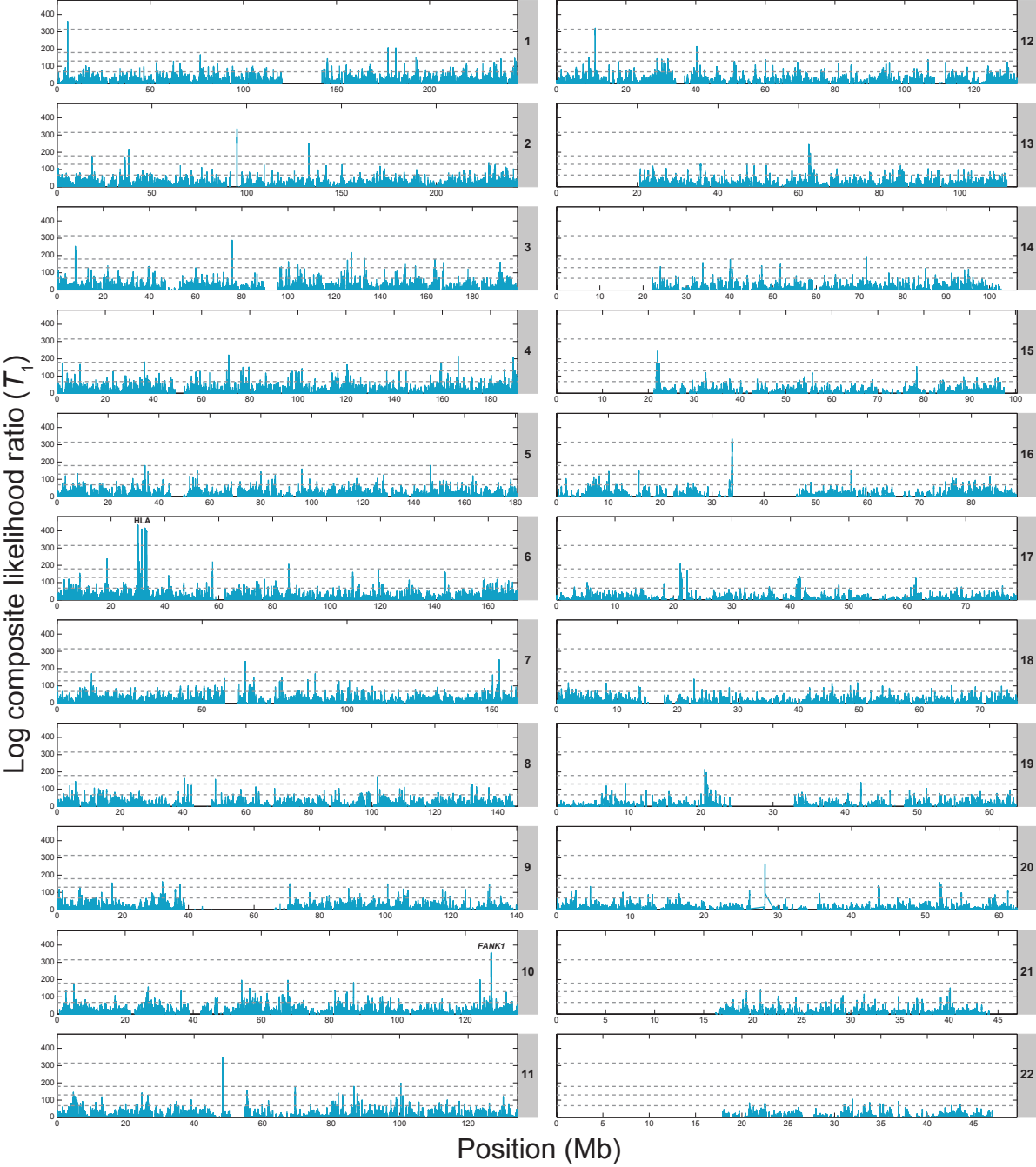

Supplement: Figure S18 — Manhattan plot of genome-wide scans for balancing selection within the CEU population using the test statistic. From bottom to top, the horizontal dotted gray lines indicate the , , , and empirical cutoffs, respectively. The -axis is truncated at log composite likelihood ratio of zero. (PDF) [file pgen.1004561.s018.pdf]

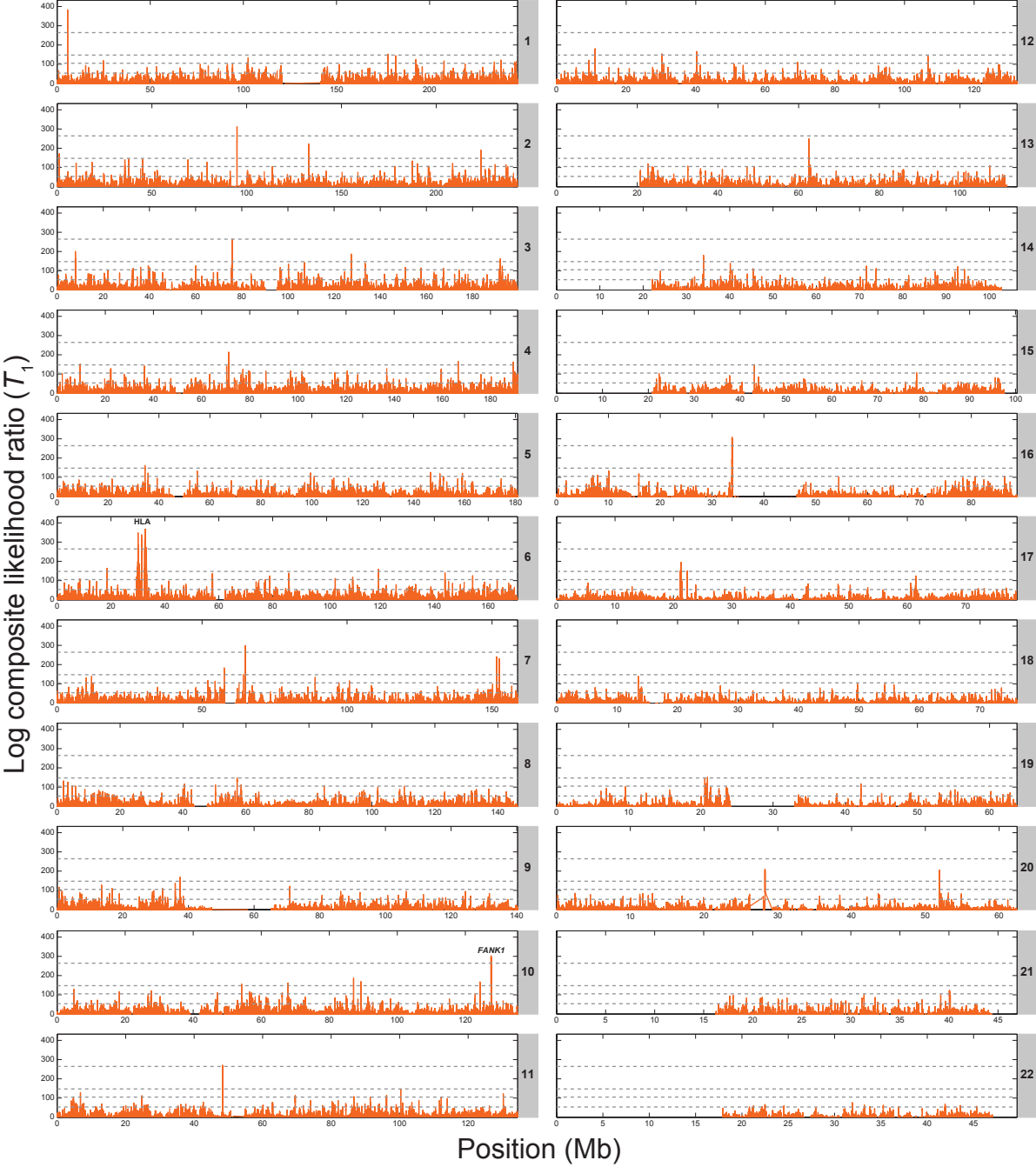

Supplement: Figure S19 — Manhattan plot of genome-wide scans for balancing selection within the YRI population using the test statistic. From bottom to top, the horizontal dotted gray lines indicate the , , , and empirical cutoffs, respectively. The -axis is truncated at log composite likelihood ratio of zero. (PDF) [file pgen.1004561.s019.pdf]

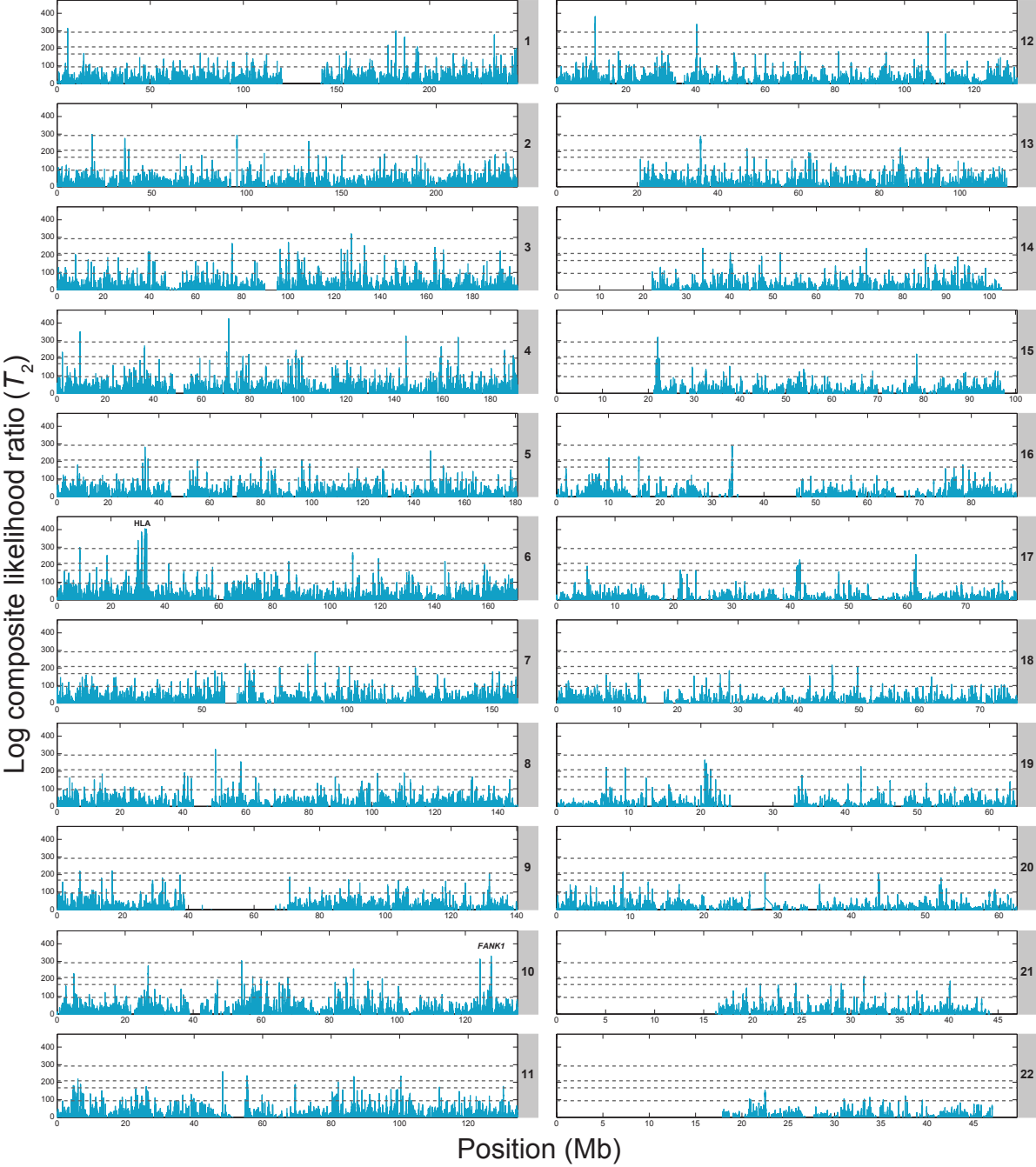

Supplement: Figure S20 — Manhattan plot of genome-wide scans for balancing selection within the CEU population using the test statistic. From bottom to top, the horizontal dotted gray lines indicate the , , , and empirical cutoffs, respectively. The -axis is truncated at log composite likelihood ratio of zero. (PDF) [file pgen.1004561.s020.pdf]

Log composite likelihood ratio ( $T_2$ )

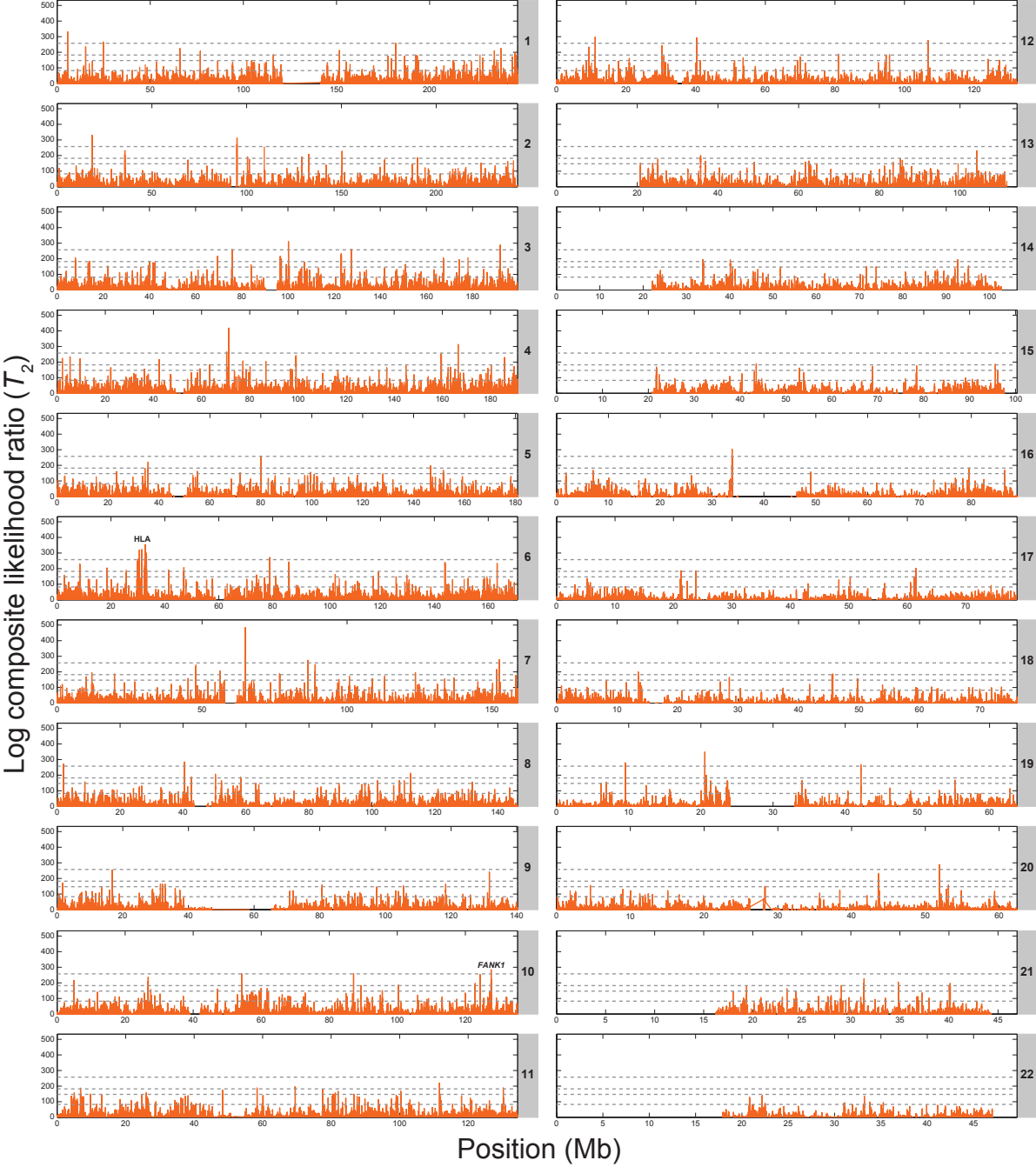

Position (Mb)

Supplement: Figure S21 — Manhattan plot of genome-wide scans for balancing selection within the YRI population using the test statistic. From bottom to top, the horizontal dotted gray lines indicate the , , , and empirical cutoffs, respectively. The -axis is truncated at log composite likelihood ratio of zero. (PDF) [file pgen.1004561.s021.pdf]

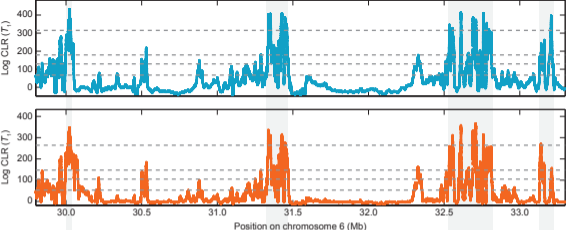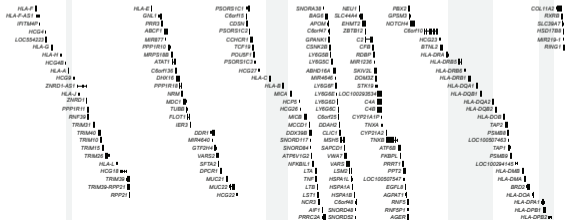

Supplement: Figure S22 — Signals of balancing selection within the HLA region for the CEU (blue) and YRI (orange) populations using the test statistic. From bottom to top, the horizontal dotted gray lines indicate the , , , and empirical cutoffs, respectively. (PDF) [file pgen.1004561.s022.pdf]

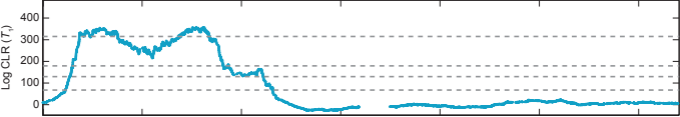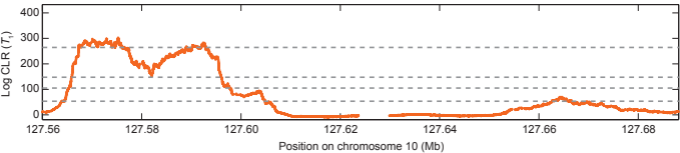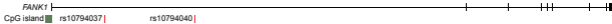

Supplement: Figure S23 — Signal of balancing selection at the FANK1 gene for the CEU (blue) and YRI (orange) populations using the test statistic. From bottom to top, the horizontal dotted gray lines indicate the , , , and empirical cutoffs, respectively. SNPs (rsIDs) correspond to markers showing significant levels of transmission distortion within the Meyer et al. study. (PDF) [file pgen.1004561.s023.pdf]

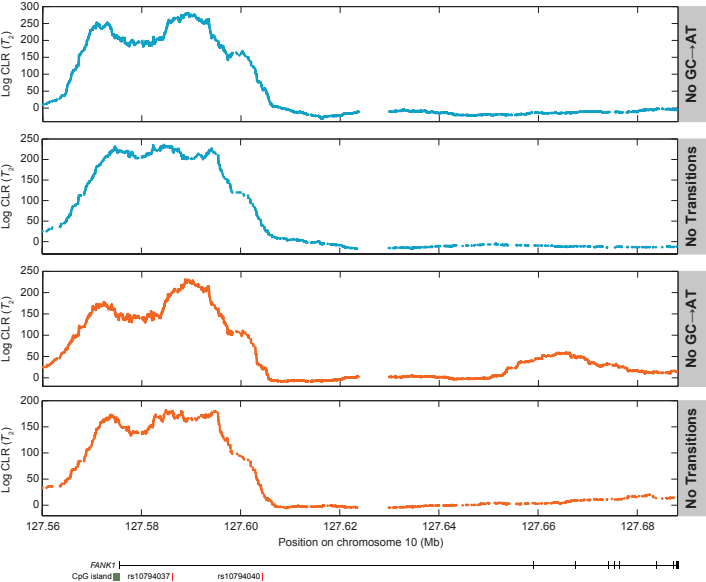

Supplement: Figure S24 — Signal of balancing selection at the FANK1 gene for the CEU (blue) and YRI (orange) populations when removing either transitions or all transitions. SNPs (rsIDs) correspond to markers showing significant levels of transmission distortion within the Meyer et al. study. (PDF) [file pgen.1004561.s024.pdf]

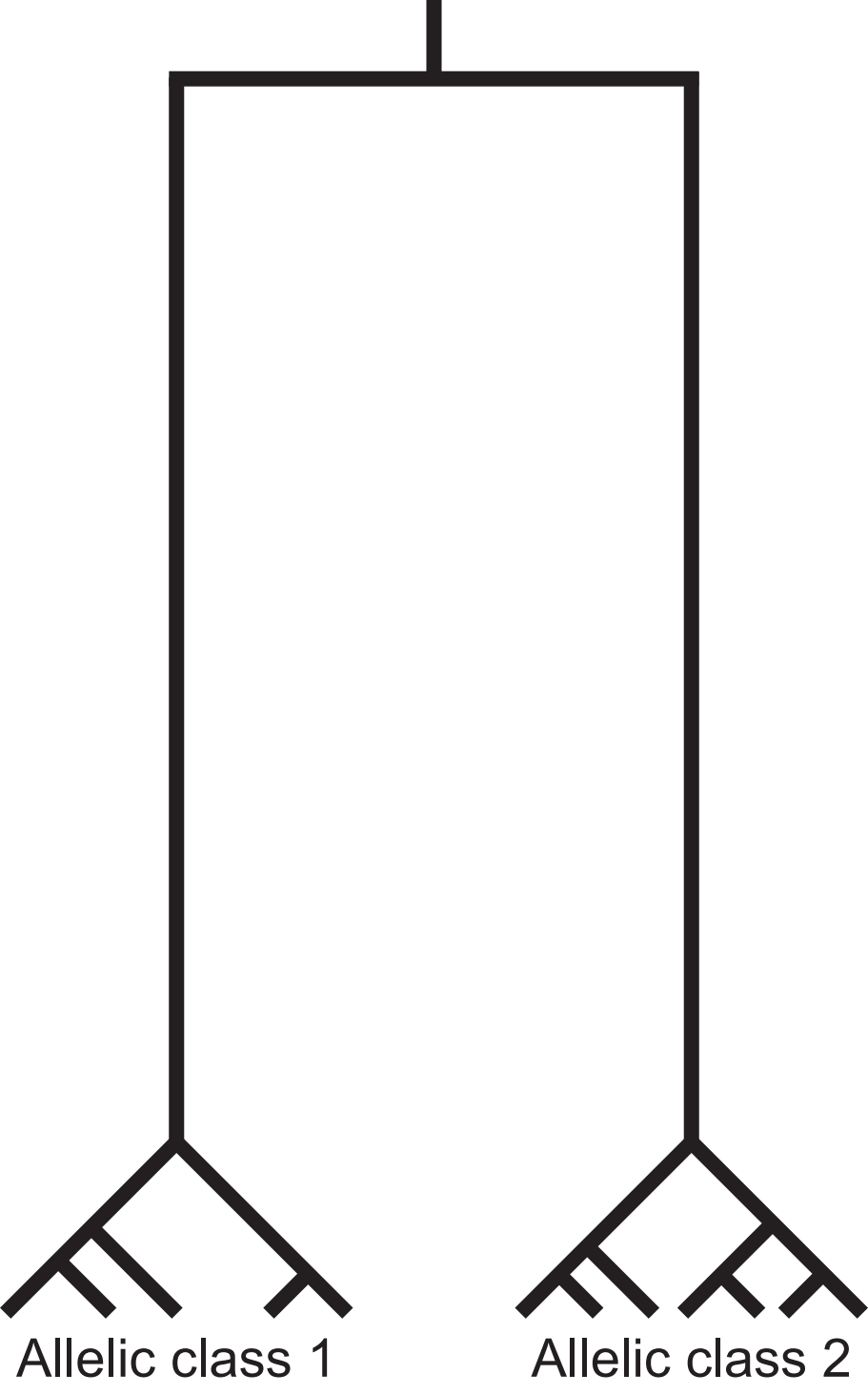

Supplement: Figure S25 — Genealogy at the site of balancing selection. (PDF) [file pgen.1004561.s025.pdf]

$s = 0.01, h = 100$

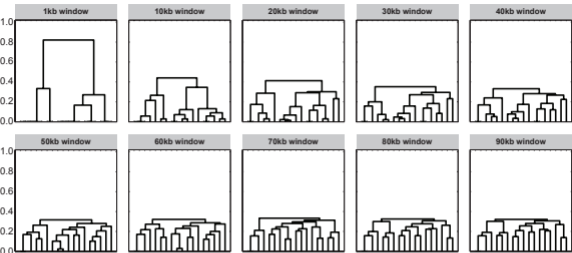

**Neutrality**

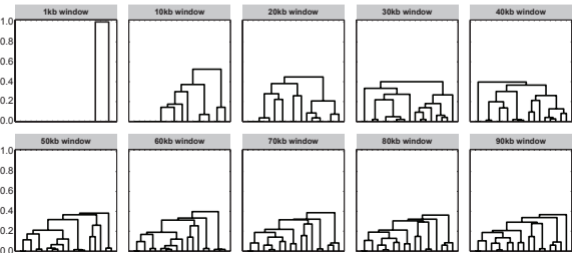

Supplement: Figure S26 — Haplotype trees based on randomly sampling 18 haplotypes without replacement from a random simulation under the model in Figure S7 A. Trees were generated using UPGMA applied to a distance matrix of the proportion of nucleotide differences between each pair of haplotypes. The -kilobase (kb) window represents a region that is kb in length and is centered in the middle of the haplotype. (PDF) [file pgen.1004561.s026.pdf]
