# Supplementary material for: A Model-Based Approach for Identifying Signatures of Ancient Balancing Selection in Genetic Data
Source: PLoS Genet. 2014 Aug 21;10(8):e1004561. doi: 10.1371/journal.pgen.1004561 (PMC4140648; doi:10.1371/journal.pgen.1004561)
Supplement: Table S1 — Top 100 signals in the CEU population using the test statistic. (PDF) [file pgen.1004561.s027.pdf]

Table S1: Top 100 signals in the CEU population using the T_1 test statistic.

Rank	Gene name	Chromosome	T_1	Rank	Gene name	Chromosome	T_1
1	***HLA-A***[74,75]	6	375.3	51	*ZNF568*	19	137.9
2	***HLA-B***[15,74,75]	6	366.3	52	*MYRIP*	3	136.8
3	*FANK1*	10	357.9	53	*IGSF5*	21	136.2
4	***HLA-C***[75]	6	320.9	54	*KIAA1267*	17	135.0
5	*TEKT4*	2	307.8	55	*CCDC169-SOHLH2*	13	134.9
6	***HLA-DPB1***[14,75]	6	262.0	56	*CCDC169*	13	134.9
7	*OR4C3*	11	246.8	57	*KIAA1324L*	7	134.5
8	***HLA-DPA1***[76]	6	244.4	58	*RNF150*	4	133.9
9	***HLA-DQA1***[14,75]	6	240.9	59	*OR51F1*	11	130.1
10	*RNF144B*	6	237.8	60	*DLG2*	11	130.0
11	*CPE*	4	216.3	61	*KDM4C*	9	129.7
12	*AXDND1*	1	206.9	62	*HLA-DRA*	6	129.0
13	*ARPC5*	1	205.5	63	***FHIT***[77]	3	128.3
14	***HLA-DQB1***[14]	6	204.0	64	*ANO3*	11	128.1
15	*DMBT1*	10	199.2	65	***CDSN***[15]	6	127.5
16	*ARHGAP42*	11	198.9	66	***PSORS1C1***[78]	6	127.5
17	*ZNF717*	3	196.6	67	*SH3RF3*	2	127.3
18	*CTNNA3*	10	195.8	68	*CCDC146*	7	127.1
19	*RGS6*	14	193.9	69	*L1TD1*	1	126.0
20	***HLA-DRB1***[14,75]	6	189.4	70	*FMO5*	1	125.9
21	*CPNE4*	3	184.1	71	*CEP112*	17	125.0
22	*RGL1*	1	181.3	72	*TRPC6*	11	124.8
23	*STK32A*	5	179.7	73	*C4orf37*	4	124.7
24	*POLN*	4	175.3	74	***PRIM2***[79]	6	124.2
25	*KALRN*	3	172.3	75	*ADH1C*	4	124.0
26	*SNX31*	8	171.7	76	***MYO3A***[80]	10	123.4
27	*MAP2K3*	17	169.0	77	*IGFBP7*	4	123.0
28	***SLC2A9***[15]	4	166.7	78	*HLA-DRB5*	6	122.7
29	*KCNJ12*	17	159.8	79	*CSMD1*	8	122.0
30	*KCNJ18*	17	159.8	80	*EMID2*	7	121.9
31	***ERAP1***[81]	5	159.2	81	*LRP1B*	2	121.7
32	*APBB1IP*	10	156.1	82	*CHRNB3*	8	121.6
33	*BNC2*	9	154.5	83	*KRT83*	12	121.4
34	*FRAS1*	4	151.1	84	*PTPRB*	12	121.3
35	*WWTR1*	3	150.9	85	*LGALS8*	1	120.9
36	*ART3*	4	150.3	86	*GCOM1*	15	120.9
37	*SLC38A9*	5	150.3	87	*SNX19*	11	120.1
38	*FXN*	9	150.0	88	*SCP2*	1	119.8
39	*FOPNL*	16	148.8	89	*ANK3*	10	119.4
40	***MAGI2***[82]	7	146.8	90	*OR51B6*	11	119.4
41	*POLR1E*	9	146.0	91	*OR5W2*	11	119.0
42	*BCAS1*	20	145.6	92	*RIMBP2*	12	118.9
43	*USP20*	9	145.3	93	*ADH4*	4	118.6
44	*GRIN2A*	16	145.2	94	*ITGA1*	5	118.5
45	*OR4C45*	11	145.0	95	*EMR1*	19	118.2
46	*SPEF2*	5	143.6	96	*PACRG*	6	118.1
47	*MSH3*	5	143.3	97	*KANK1*	9	117.9
48	*LUZP2*	11	141.1	98	*APOBEC4*	1	117.8
49	*COL4A3*	2	139.3	99	*ZDHHC7*	16	117.5
50	*MLF1IP*	4	138.1	100	*ANK2*	4	117.4

Previously-hypothesized genes are indicated in bold. Genes overlapping with Table S2 are highlighted in gray.

74 P. W. Hedrick, T. S. Whittam, and P. Parham, “Heterozygosity at individual amino acid sites: extremely high levels for *HLA-A* and *-B* genes,” *Proc. Natl. Acad. Sci. USA*, vol. 88, pp. 58975901, 1991.

75 A. Sánchez-Mazas, “An apportionment of human HLA diversity,” *Tissue Antigens*, vol. 69, pp. 198202, 2005.

76 P. G. Bronson, S. J. Mack, H. A. Erlich, and M. Slatkin, “A sequence-based approach demonstrates that balancing selection in classical human leukocyte antigen (HLA) loci is asymmetric,” *Hum. Mol. Genet.*, vol. 22, pp. 252261, 2013.

77 Y. Ding, G. Larson, G. Rivas, C. Lundberg, L. Geller, C. Ouyang, J.Weitzel, J. Archambeau, J. Slater, M. B. Daly, A. B. Benson, J. M. Kirkwood, P. J. ODwyer, R. Sutphen, J. A. Stewart, D. Johnson, M. Nordborg, and T. G. Krontiris, “Strong signature of natural selection within an *FHIT* intron implicated in prostate cancer risk,” *PLoS ONE*, vol. 3, p. e3533, 2008.

78 R. Cagliani, S. Riva, U. Pozzoli, M. Fumagalli, G. P. Comi, N. Bresolin, M. Clerici, and M. Sironi, “Balancing selection is common in the extended MHC region but most alleles with opposite risk profile for autoimmune diseases are neutraly evolving,” *BMC Evol. Biol.*, vol. 1, p. 171, 2011.

79 A. Hodgkinson and A. Eyre-Walker, “The genomic distribution and local context of coincident SNPs in human and chimpanzee,” *Genome Biol. Evol.*, vol. 2, pp. 547557, 2010.

80 S. Asthana, S. Schmidt, and S. Sunyaev, “A limited role for balancing selection,” *Trends Genet.*, vol. 21, pp. 3032, 2005.

81 R. Cagliani, S. Riva, M. Biasin, M. Fumagalli, U. Pozzoli, S. Lo Caputo, F. Mazzotta, L. Piacentini, N. Bresolin, M. Clerici, and M. Sironi, “Genetic diversity at endoplasmic reticulum aminopeptidases is maintained by balancing selection and is associated with natural resistance to HIV-1 infection,” *Hum. Mol. Genet.*, vol. 10, pp. 47054714, 2010.

82 L. Excoffier, T. Hofer, and M. Foll, “Detecting loci under selection in a hierarchically structured population,” *Heredity*, vol. 103, pp. 2852981, 2009.
