# Supplementary material for: A Model-Based Approach for Identifying Signatures of Ancient Balancing Selection in Genetic Data
Source: PLoS Genet. 2014 Aug 21;10(8):e1004561. doi: 10.1371/journal.pgen.1004561 (PMC4140648; doi:10.1371/journal.pgen.1004561)
Supplement: Table S3 — Top 100 signals in the CEU population using the test statistic. (PDF) [file pgen.1004561.s029.pdf]

Table S3: Top 100 signals in the CEU population using the T_2 test statistic.

Rank	Gene name	Chromosome	T_2	Rank	Gene name	Chromosome	T_2
1	***HLA-DPB1*** [14]	6	405.3	51	***WFDC8*** [83]	20	204.2
2	***HLA-DPA1*** [76]	6	363.5	52	*USP20*	9	203.7
3	***SLC2A9*** [15]	4	350.0	53	*CCDC146*	7	201.5
4	*FANK1*	10	330.0	54	***HLA-DRB1*** [14,75]	6	197.9
5	*CPE*	4	317.9	55	*POLR1E*	9	197.1
6	***HLA-C*** [75]	6	317.7	56	*ADH4*	4	196.5
7	*DMBT1*	10	312.2	57	*OR5W2*	11	195.8
8	***HLA-A*** [74,75]	6	309.6	58	*ASB18*	2	195.0
9	*ARPC5*	1	297.8	59	*BICC1*	10	194.3
10	*CCDC169*	13	286.0	60	*SH3RF3*	2	192.6
11	*CCDC169-SOHLH2*	13	286.0	61	*BMPR1B*	4	192.1
12	***LGALS8*** [15]	1	277.0	62	*NUP88*	17	190.9
13	*APBB1IP*	10	274.0	63	*ADAMTS12*	5	190.1
14	*RGL1*	1	268.4	64	*ADH1C*	4	188.7
15	*TEKT4*	2	261.8	65	*APOBEC4*	1	187.8
16	*STK32A*	5	259.2	66	*SNX31*	8	187.5
17	*CEP112*	17	257.7	67	*SGCZ*	8	187.1
18	*CPNE4*	3	253.0	68	*PANK1*	10	187.0
19	*RNF144B*	6	252.0	69	*ANK3*	10	186.9
20	*C4orf37*	4	245.2	70	*PKD1L1*	7	186.8
21	*MLF1IP*	4	244.1	71	*ZNF717*	3	186.5
22	***HLA-B*** [15,74,75]	6	237.0	72	*KLHL14*	18	184.2
23	*RGS6*	14	236.1	73	*FXN*	9	184.0
24	*ARHGAP42*	11	234.8	74	*MAPT*	17	182.6
25	*POLN*	4	233.4	75	*TYW1*	7	182.0
26	*SLC15A2*	3	232.8	76	*BCAS1*	20	180.4
27	*KALRN*	3	227.2	77	*NTN4*	12	179.8
28	*FOPNL*	16	227.1	78	*THSD7B*	2	177.7
29	*KIAA1267*	17	226.7	79	*COL5A2*	2	177.6
30	*ZNF568*	19	226.4	80	*LRP1B*	2	175.6
31	*HEATR1*	1	225.7	81	*SNX19*	11	175.4
32	*KIAA1324L*	7	223.4	82	*OR4C3*	11	174.7
33	*MSH3*	5	222.5	83	*KRT83*	12	173.5
34	*FRAS1*	4	221.2	84	*LRRC16A*	6	171.5
35	*EMR1*	19	221.1	85	*C18orf1*	18	170.3
36	*GRIN2A*	16	220.1	86	*HLA-DRA*	6	169.8
37	*BNC2*	9	220.1	87	*PTPN14*	1	169.5
38	***HLA-DQA1*** [14,75]	6	220.1	88	*CNBD1*	8	167.1
39	*KDM4C*	9	217.8	89	*GPC5*	13	166.6
40	*AXDND1*	1	217.1	90	*MAP2K3*	17	164.6
41	*MYRIP*	3	215.8	91	*CSMD1*	8	164.5
42	*SPEF2*	5	214.2	92	*CADM2*	3	162.8
43	*PLCB4*	20	212.3	93	*MYO1B*	2	162.6
44	*SLC38A9*	5	210.2	94	*MYLK4*	6	162.0
45	*ART3*	4	209.3	95	***OR5I1*** [55]	11	162.0
46	*CTNNA3*	10	208.3	96	***FHIT*** [77]	3	161.9
47	***ERAP1*** [81]	5	207.6	97	*CUBN*	10	161.8
48	***HLA-DQB1*** [14,75]	6	206.8	98	*ULK4*	3	161.8
49	*EMID2*	7	206.1	99	*ASTN2*	9	161.6
50	*ADCY5*	3	205.4	100	*PGLYRP4*	1	161.3

Previously-hypothesized genes are indicated in bold. Genes overlapping with Table S4 are highlighted in gray.

74 P. W. Hedrick, T. S. Whittam, and P. Parham, "Heterozygosity at individual amino acid sites: extremely high levels for *HLA-A* and *-B* genes," *Proc. Natl. Acad. Sci. USA*, vol. 88, pp. 58975901, 1991.

75 A. Sánchez-Mazas, "An apportionment of human HLA diversity," *Tissue Antigens*, vol. 69, pp. 198202, 2005.

76 P. G. Bronson, S. J. Mack, H. A. Erlich, and M. Slatkin, "A sequence-based approach demonstrates that balancing selection in classical human leukocyte antigen (HLA) loci is asymmetric," *Hum. Mol. Genet.*, vol. 22, pp. 252261, 2013.

77 Y. Ding, G. Larson, G. Rivas, C. Lundberg, L. Geller, C. Ouyang, J.Weitzel, J. Archambeau, J. Slater, M. B. Daly, A. B. Benson, J. M. Kirkwood, P. J. ODwyer, R. Sutphen, J. A. Stewart, D. Johnson, M. Nordborg, and T. G. Krontiris, "Strong signature of natural selection within an *FHIT* intron implicated in prostate cancer risk," *PLoS ONE*, vol. 3, p. e3533, 2008.

81 R. Cagliani, S. Riva, M. Biasin, M. Fumagalli, U. Pozzoli, S. Lo Caputo, F. Mazzotta, L. Piacentini, N. Bresolin, M. Clerici, and M. Sironi, "Genetic diversity at endoplasmic reticulum aminopeptidases is maintained by balancing selection and is associated with natural resistance to HIV-1 infection," *Hum. Mol. Genet.*, vol. 10, pp. 47054714, 2010.

83 Z. Ferreira, B. Hurle, J. Rocha, and S. Seixas, "Differing evolutionary histories of *WFDC8* (short-term balancing) in Europeans and *SPINT4* (incomplete selective sweep) in Africans," *Mol. Biol. Evol.*, vol. 28, pp. 28112822, 2011.
