# Supplementary material for: A Model-Based Approach for Identifying Signatures of Ancient Balancing Selection in Genetic Data
Source: PLoS Genet. 2014 Aug 21;10(8):e1004561. doi: 10.1371/journal.pgen.1004561 (PMC4140648; doi:10.1371/journal.pgen.1004561)
Supplement: Table S4 — Top 100 signals in the YRI population using the test statistic. (PDF) [file pgen.1004561.s030.pdf]

Table S4: Top 100 signals in the YRI population using the T_2 test statistic.

Rank	Gene name	Chromosome	T_2	Rank	Gene name	Chromosome	T_2
1	*CPE*	4	311.9	51	*HPSE2*	10	185.0
2	***HLA-DPA1*** [76]	6	298.7	52	*IGSF5*	21	184.3
3	***HLA-DPB1*** [14]	6	290.9	53	*CASQ2*	1	183.6
4	*FANK1*	10	283.6	54	*MYRIP*	3	183.1
5	*TEKT4*	2	277.2	55	*FRG2C*	3	182.8
6	*KIAA1324L*	7	273.7	56	*APOBEC4*	1	181.1
7	*MYOM2*	8	271.1	57	*NTN4*	12	178.9
8	*ZNF568*	19	266.8	58	*ALG8*	11	178.4
9	*C1orf130*	1	265.2	59	*ESYT2*	7	177.1
10	*ARPC5*	1	256.7	60	*ATP8A2*	13	175.5
11	*MSH3*	5	256.6	61	*RFX8*	2	175.4
12	*SH3RF3*	2	256.6	62	*ULK4*	3	174.6
13	*DMBT1*	10	253.8	63	*AXDND1*	1	174.3
14	*BNC2*	9	253.2	64	*EMID2*	7	170.6
15	*PKD1L1*	7	247.7	65	*SMYD3*	1	169.5
16	*USP20*	9	240.8	66	***HLA-B*** [15,74,75]	6	167.9
17	*C4orf37*	4	239.5	67	*VRK3*	19	167.5
18	*APBB1IP*	10	236.4	68	*ARHGAP42*	11	167.4
19	*STK32B*	4	233.7	69	*RBFOX1*	16	167.1
20	*SLC15A2*	3	233.1	70	*C15orf48*	15	166.8
21	*PACRG*	6	231.9	71	*GBA3*	4	166.8
22	***WFDC8*** [83]	20	231.4	72	*KLHL14*	18	163.7
23	*RGL1*	1	230.9	73	*BICC1*	10	163.7
24	*MLF1IP*	4	228.9	74	*SNX31*	8	163.5
25	*POLN*	4	223.2	75	*WWTR1*	3	163.5
26	***SLC2A9*** [15]	4	221.1	76	*KIAA0748*	12	163.5
27	*SPEF2*	5	220.0	77	*ASTN2*	9	162.5
28	*FRMD4B*	3	215.6	78	*ANK3*	10	162.4
29	*MLL3*	7	215.0	79	*PGBD5*	1	162.3
30	*PGLYRP4*	1	211.2	80	*SLC38A9*	5	161.0
31	***LGALS8*** [15]	1	209.4	81	*SLCO1B3*	12	160.3
32	*ART3*	4	205.9	82	*DGKI*	7	159.8
33	*RCAN1*	21	204.0	83	*RAMP3*	7	157.5
34	*ARHGAP24*	4	203.5	84	*LAMA2*	6	157.2
35	*RNF144B*	6	203.3	85	***HLA-A*** [74,75]	6	156.8
36	*CEP112*	17	201.9	86	*ACBD5*	10	155.8
37	*HLA-DRB5*	6	199.9	87	*MYLK4*	6	155.5
38	*CCDC169*	13	198.9	88	*DHX37*	12	154.6
39	*CCDC169-SOHLH2*	13	198.9	89	*EMR1*	19	154.2
40	*C18orf1*	18	198.9	90	*RYR2*	1	152.8
41	*STK32A*	5	196.7	91	*BCKDHB*	6	150.8
42	*SPATA16*	3	194.1	92	*C16orf73*	16	150.7
43	*LRRC16A*	6	192.5	93	*FAHD1*	16	150.7
44	***HLA-C*** [75]	6	192.2	94	***RCBTB1*** [15]	13	150.6
45	***HLA-DQB1*** [14,75]	6	191.9	95	*RGS6*	14	148.8
46	*SNX19*	11	187.7	96	*ACSBG2*	19	148.5
47	*CHRNB3*	8	187.5	97	*SWAP70*	11	148.0
48	*CCDC146*	7	186.5	98	*ABCD4*	14	147.7
49	*WDR75*	2	186.1	99	*PTPRB*	12	147.7
50	*MYO5B*	18	186.0	100	*PTPN14*	1	146.1

Previously-hypothesized genes are indicated in bold. Genes overlapping with Table S3 are highlighted in gray.

74 P. W. Hedrick, T. S. Whittam, and P. Parham, "Heterozygosity at individual amino acid sites: extremely high levels for *HLA-A* and *-B* genes," *Proc. Natl. Acad. Sci. USA*, vol. 88, pp. 58975901, 1991.

75 A. Sánchez-Mazas, "An apportionment of human HLA diversity," *Tissue Antigens*, vol. 69, pp. 198202, 2005.

76 P. G. Bronson, S. J. Mack, H. A. Erlich, and M. Slatkin, "A sequence-based approach demonstrates that balancing selection in classical human leukocyte antigen (HLA) loci is asymmetric," *Hum. Mol. Genet.*, vol. 22, pp. 252261, 2013.

83 Z. Ferreira, B. Hurle, J. Rocha, and S. Seixas, "Differing evolutionary histories of *WFDC8* (short-term balancing) in Europeans and *SPINT4* (incomplete selective sweep) in Africans," *Mol. Biol. Evol.*, vol. 28, pp. 28112822, 2011.
