# Supplementary material for: A Model-Based Approach for Identifying Signatures of Ancient Balancing Selection in Genetic Data
Source: PLoS Genet. 2014 Aug 21;10(8):e1004561. doi: 10.1371/journal.pgen.1004561 (PMC4140648; doi:10.1371/journal.pgen.1004561)
Supplement: Table S5 — GO process analysis of top 100 signals, when compared to all signals, from CEU population using the test statistic. (PDF) [file pgen.1004561.s031.pdf]

Table S5: GO process analysis of top 100 signals, when compared to all signals, from CEU population using the T_1 test statistic.

Description	p-value	Enrichment	Genes
Interferon-gamma-mediated signaling pathway	1.7×10^{-12}	28.6	*HLA-A*, *HLA-B*, *HLA-C*, *HLA-DPA1*, *HLA-DPB1*, *HLA-DQA1*, *HLA-DQB1*, *HLA-DRA*, *HLA-DRB1*, *HLA-DRB5*
Cellular response to interferon-gamma	1.5×10^{-11}	23.1	*HLA-A*, *HLA-B*, *HLA-C*, *HLA-DPA1*, *HLA-DPB1*, *HLA-DQA1*, *HLA-DQB1*, *HLA-DRA*, *HLA-DRB1*, *HLA-DRB5*
Response to interferon-gamma	1.1×10^{-10}	19.0	*HLA-A*, *HLA-B*, *HLA-C*, *HLA-DPA1*, *HLA-DPB1*, *HLA-DQA1*, *HLA-DQB1*, *HLA-DRA*, *HLA-DRB1*, *HLA-DRB5*
Antigen processing and presentation of peptide antigen	2.2×10^{-9}	11.8	*ERAP1*, *HLA-A*, *HLA-B*, *HLA-C*, *HLA-DPA1*, *HLA-DPB1*, *HLA-DQA1*, *HLA-DQB1*, *HLA-DRA*, *HLA-DRB1*, *HLA-DRB5*
Antigen processing and presentation	8.8×10^{-9}	10.3	*ERAP1*, *HLA-A*, *HLA-B*, *HLA-C*, *HLA-DPA1*, *HLA-DPB1*, *HLA-DQA1*, *HLA-DQB1*, *HLA-DRA*, *HLA-DRB1*, *HLA-DRB5*
Antigen processing and presentation of exogenous peptide antigen	1.6×10^{-8}	11.5	*HLA-A*, *HLA-B*, *HLA-C*, *HLA-DPA1*, *HLA-DPB1*, *HLA-DQA1*, *HLA-DQB1*, *HLA-DRA*, *HLA-DRB1*, *HLA-DRB5*
Antigen processing and presentation of exogenous antigen	1.8×10^{-8}	11.3	*HLA-A*, *HLA-B*, *HLA-C*, *HLA-DPA1*, *HLA-DPB1*, *HLA-DQA1*, *HLA-DQB1*, *HLA-DRA*, *HLA-DRB1*, *HLA-DRB5*
T cell costimulation	4.4×10^{-8}	20.7	*HLA-DPA1*, *HLA-DPB1*, *HLA-DQA1*, *HLA-DQB1*, *HLA-DRA*, *HLA-DRB1*, *HLA-DRB5*
Lymphocyte costimulation	4.9×10^{-8}	20.4	*HLA-DPA1*, *HLA-DPB1*, *HLA-DQA1*, *HLA-DQB1*, *HLA-DRA*, *HLA-DRB1*, *HLA-DRB5*
T cell receptor signaling pathway	2.0×10^{-7}	16.6	*HLA-DPA1*, *HLA-DPB1*, *HLA-DQA1*, *HLA-DQB1*, *HLA-DRA*, *HLA-DRB1*, *HLA-DRB5*
Antigen processing and presentation of exogenous peptide antigen via MHC class II	4.1×10^{-7}	15.0	*HLA-DPA1*, *HLA-DPB1*, *HLA-DQA1*, *HLA-DQB1*, *HLA-DRA*, *HLA-DRB1*, *HLA-DRB5*
Protein localization to membrane	4.6×10^{-7}	20.4	*ANK2*, *ANK3*, *CPE*, *DLG2*, *MAGI2*, *SCP2*
Antigen processing and presentation of peptide or polysaccharide antigen via MHC class II	4.8×10^{-7}	14.7	*HLA-DPA1*, *HLA-DPB1*, *HLA-DQA1*, *HLA-DQB1*, *HLA-DRA*, *HLA-DRB1*, *HLA-DRB5*
Antigen processing and presentation of peptide antigen via MHC class II	4.8×10^{-7}	14.7	*HLA-DPA1*, *HLA-DPB1*, *HLA-DQA1*, *HLA-DQB1*, *HLA-DRA*, *HLA-DRB1*, *HLA-DRB5*
Detection of bacterium	6.1×10^{-7}	55.5	*HLA-A*, *HLA-B*, *HLA-DRB1*, *HLA-DRB5*
Immune response-activating signal transduction	6.8×10^{-7}	9.0	*DMBT1*, *HLA-DPA1*, *HLA-DPB1*, *HLA-DQA1*, *HLA-DQB1*, *HLA-DRA*, *HLA-DRB1*, *HLA-DRB5*, *MAP2K3*
Immune response-regulating signaling pathway	1.2×10^{-6}	8.5	*DMBT1*, *HLA-DPA1*, *HLA-DPB1*, *HLA-DQA1*, *HLA-DQB1*, *HLA-DRA*, *HLA-DRB1*, *HLA-DRB5*, *MAP2K3*
Antigen receptor-mediated signaling pathway	1.2×10^{-6}	12.9	*HLA-DPA1*, *HLA-DPB1*, *HLA-DQA1*, *HLA-DQB1*, *HLA-DRA*, *HLA-DRB1*, *HLA-DRB5*
Immune response-activating cell surface receptor signaling pathway	1.8×10^{-6}	12.1	*HLA-DPA1*, *HLA-DPB1*, *HLA-DQA1*, *HLA-DQB1*, *HLA-DRA*, *HLA-DRB1*, *HLA-DRB5*
Regulation of immune response	2.3×10^{-6}	4.9	*DMBT1*, *ERAP1*, *HLA-A*, *HLA-B*, *HLA-C*, *HLA-DPA1*, *HLA-DPB1*, *HLA-DQA1*, *HLA-DQB1*, *HLA-DRA*, *HLA-DRB1*, *HLA-DRB5*, *MAP2K3*
Immune response-regulating cell surface receptor signaling pathway	3.2×10^{-6}	11.1	*HLA-DPA1*, *HLA-DPB1*, *HLA-DQA1*, *HLA-DQB1*, *HLA-DRA*, *HLA-DRB1*, *HLA-DRB5*
Immunoglobulin production involved in immunoglobulin mediated immune response	3.3×10^{-6}	90.1	*HLA-DQB1*, *HLA-DRB1*, *HLA-DRB5*
Humoral immune response mediated by circulating immunoglobulin	3.3×10^{-6}	90.1	*HLA-DQB1*, *HLA-DRB1*, *HLA-DRB5*
Cytokine-mediated signaling pathway	4.0×10^{-6}	6.3	*HLA-A*, *HLA-B*, *HLA-C*, *HLA-DPA1*, *HLA-DPB1*, *HLA-DQA1*, *HLA-DQB1*, *HLA-DRA*, *HLA-DRB1*, *HLA-DRB5*
Activation of immune response	4.5×10^{-6}	7.2	*DMBT1*, *HLA-DPA1*, *HLA-DPB1*, *HLA-DQA1*, *HLA-DQB1*, *HLA-DRA*, *HLA-DRB1*, *HLA-DRB5*, *MAP2K3*
Detection of biotic stimulus	7.2×10^{-6}	31.4	*HLA-A*, *HLA-B*, *HLA-DRB1*, *HLA-DRB5*
Immunoglobulin production	9.1×10^{-6}	67.6	*HLA-DQB1*, *HLA-DRB1*, *HLA-DRB5*
Antigen processing and presentation of exogenous peptide antigen via MHC class I TAP-independent	1.4×10^{-5}	60.1	*HLA-A*, *HLA-B*, *HLA-C*
Positive regulation of T cell activation	2.3×10^{-5}	8.2	*HLA-DPA1*, *HLA-DPB1*, *HLA-DQA1*, *HLA-DQB1*, *HLA-DRA*, *HLA-DRB1*, *HLA-DRB5*

GO categories in which false discovery rate is less than 0.01.
