# Supplementary material for: A Model-Based Approach for Identifying Signatures of Ancient Balancing Selection in Genetic Data
Source: PLoS Genet. 2014 Aug 21;10(8):e1004561. doi: 10.1371/journal.pgen.1004561 (PMC4140648; doi:10.1371/journal.pgen.1004561)
Supplement: Table S6 — GO process analysis of top 100 signals, when compared to all signals, from YRI population using the test statistic. (PDF) [file pgen.1004561.s032.pdf]

Table S6: GO process analysis of top 100 signals, when compared to all signals, from YRI population using the T_1 test statistic.

Description	p-value	Enrichment	Genes
Interferon-gamma-mediated signaling pathway	3.2×10^{-12}	26.8	*HLA-A*, *HLA-B*, *HLA-C*, *HLA-DPA1*, *HLA-DPB1*, *HLA-DQA1*, *HLA-DQB1*, *HLA-DRA*, *HLA-DRB1*, *HLA-DRB5*
Cellular response to interferon-gamma	2.9×10^{-11}	21.7	*HLA-A*, *HLA-B*, *HLA-C*, *HLA-DPA1*, *HLA-DPB1*, *HLA-DQA1*, *HLA-DQB1*, *HLA-DRA*, *HLA-DRB1*, *HLA-DRB5*
Response to interferon-gamma	2.2×10^{-10}	17.8	*HLA-A*, *HLA-B*, *HLA-C*, *HLA-DPA1*, *HLA-DPB1*, *HLA-DQA1*, *HLA-DQB1*, *HLA-DRA*, *HLA-DRB1*, *HLA-DRB5*
T cell receptor signaling pathway	1.5×10^{-8}	17.8	*FYB*, *HLA-DPA1*, *HLA-DPB1*, *HLA-DQA1*, *HLA-DQB1*, *HLA-DRA*, *HLA-DRB1*, *HLA-DRB5*
Antigen processing and presentation of exogenous peptide antigen	3.0×10^{-8}	10.8	*HLA-A*, *HLA-B*, *HLA-C*, *HLA-DPA1*, *HLA-DPB1*, *HLA-DQA1*, *HLA-DQB1*, *HLA-DRA*, *HLA-DRB1*, *HLA-DRB5*
Antigen processing and presentation of exogenous antigen	3.4×10^{-8}	10.6	*HLA-A*, *HLA-B*, *HLA-C*, *HLA-DPA1*, *HLA-DPB1*, *HLA-DQA1*, *HLA-DQB1*, *HLA-DRA*, *HLA-DRB1*, *HLA-DRB5*
Antigen processing and presentation of peptide antigen	5.7×10^{-8}	10.1	*HLA-A*, *HLA-B*, *HLA-C*, *HLA-DPA1*, *HLA-DPB1*, *HLA-DQA1*, *HLA-DQB1*, *HLA-DRA*, *HLA-DRB1*, *HLA-DRB5*
T cell costimulation	6.8×10^{-8}	19.4	*HLA-DPA1*, *HLA-DPB1*, *HLA-DQA1*, *HLA-DQB1*, *HLA-DRA*, *HLA-DRB1*, *HLA-DRB5*
Lymphocyte costimulation	7.7×10^{-8}	19.1	*HLA-DPA1*, *HLA-DPB1*, *HLA-DQA1*, *HLA-DQB1*, *HLA-DRA*, *HLA-DRB1*, *HLA-DRB5*
Immune response-activating signal transduction	1.1×10^{-7}	9.4	*DMBT1*, *FYB*, *HLA-DPA1*, *HLA-DPB1*, *HLA-DQA1*, *HLA-DQB1*, *HLA-DRA*, *HLA-DRB1*, *HLA-DRB5*, *MAP2K3*
Antigen receptor-mediated signaling pathway	1.1×10^{-7}	13.8	*FYB*, *HLA-DPA1*, *HLA-DPB1*, *HLA-DQA1*, *HLA-DQB1*, *HLA-DRA*, *HLA-DRB1*, *HLA-DRB5*
Immune response-activating cell surface receptor signaling pathway	1.8×10^{-7}	13.0	*FYB*, *HLA-DPA1*, *HLA-DPB1*, *HLA-DQA1*, *HLA-DQB1*, *HLA-DRA*, *HLA-DRB1*, *HLA-DRB5*
Immune response-regulating signaling pathway	2.0×10^{-7}	8.8	*DMBT1*, *FYB*, *HLA-DPA1*, *HLA-DPB1*, *HLA-DQA1*, *HLA-DQB1*, *HLA-DRA*, *HLA-DRB1*, *HLA-DRB5*, *MAP2K3*
Antigen processing and presentation	2.0×10^{-7}	8.8	*HLA-A*, *HLA-B*, *HLA-C*, *HLA-DPA1*, *HLA-DPB1*, *HLA-DQA1*, *HLA-DQB1*, *HLA-DRA*, *HLA-DRB1*, *HLA-DRB5*
Immune response-regulating cell surface receptor signaling pathway	3.7×10^{-7}	11.9	*FYB*, *HLA-DPA1*, *HLA-DPB1*, *HLA-DQA1*, *HLA-DQB1*, *HLA-DRA*, *HLA-DRB1*, *HLA-DRB5*
Antigen processing and presentation of exogenous peptide antigen via MHC class II	6.4×10^{-7}	14.1	*HLA-DPA1*, *HLA-DPB1*, *HLA-DQA1*, *HLA-DQB1*, *HLA-DRA*, *HLA-DRB1*, *HLA-DRB5*
Antigen processing and presentation of peptide or polysaccharide antigen via MHC class II	7.5×10^{-7}	13.8	*HLA-DPA1*, *HLA-DPB1*, *HLA-DQA1*, *HLA-DQB1*, *HLA-DRA*, *HLA-DRB1*, *HLA-DRB5*
Antigen processing and presentation of peptide antigen via MHC class II	7.5×10^{-7}	13.8	*HLA-DPA1*, *HLA-DPB1*, *HLA-DQA1*, *HLA-DQB1*, *HLA-DRA*, *HLA-DRB1*, *HLA-DRB5*
Detection of bacterium	7.9×10^{-7}	52.0	*HLA-A*, *HLA-B*, *HLA-DRB1*, *HLA-DRB5*
Activation of immune response	8.9×10^{-7}	7.5	*DMBT1*, *FYB*, *HLA-DPA1*, *HLA-DPB1*, *HLA-DQA1*, *HLA-DQB1*, *HLA-DRA*, *HLA-DRB1*, *HLA-DRB5*, *MAP2K3*
Positive regulation of T cell activation	3.6×10^{-6}	8.8	*HLA-DPA1*, *HLA-DPB1*, *HLA-DQA1*, *HLA-DQB1*, *HLA-DRA*, *HLA-DRB1*, *HLA-DRB5*, *TESPA1*
Immunoglobulin production involved in immunoglobulin mediated immune response	4.0×10^{-6}	84.5	*HLA-DQB1*, *HLA-DRB1*, *HLA-DRB5*
Humoral immune response mediated by circulating immunoglobulin	4.0×10^{-6}	84.5	*HLA-DQB1*, *HLA-DRB1*, *HLA-DRB5*
Regulation of immune response	4.8×10^{-6}	4.6	*DMBT1*, *FYB*, *HLA-A*, *HLA-B*, *HLA-C*, *HLA-DPA1*, *HLA-DPB1*, *HLA-DQA1*, *HLA-DQB1*, *HLA-DRA*, *HLA-DRB1*, *HLA-DRB5*, *MAP2K3*
Cytokine-mediated signaling pathway	7.1×10^{-6}	5.9	*HLA-A*, *HLA-B*, *HLA-C*, *HLA-DPA1*, *HLA-DPB1*, *HLA-DQA1*, *HLA-DQB1*, *HLA-DRA*, *HLA-DRB1*, *HLA-DRB5*
Detection of biotic stimulus	9.3×10^{-6}	29.4	*HLA-A*, *HLA-B*, *HLA-DRB1*, *HLA-DRB5*
Positive regulation of immune response	9.6×10^{-6}	5.7	*DMBT1*, *FYB*, *HLA-DPA1*, *HLA-DPB1*, *HLA-DQA1*, *HLA-DQB1*, *HLA-DRA*, *HLA-DRB1*, *HLA-DRB5*, *MAP2K3*
Immunoglobulin production	1.1×10^{-5}	63.4	*HLA-DQB1*, *HLA-DRB1*, *HLA-DRB5*
Antigen processing and presentation of exogenous peptide antigen via MHC class I TAP-independent	1.6×10^{-5}	56.3	*HLA-A*, *HLA-B*, *HLA-C*
Positive regulation of lymphocyte activation	1.9×10^{-5}	7.0	*HLA-DPA1*, *HLA-DPB1*, *HLA-DQA1*, *HLA-DQB1*, *HLA-DRA*, *HLA-DRB1*, *HLA-DRB5*, *TESPA1*

GO categories in which false discovery rate is less than 0.01.
