# Supplementary material for: A Model-Based Approach for Identifying Signatures of Ancient Balancing Selection in Genetic Data
Source: PLoS Genet. 2014 Aug 21;10(8):e1004561. doi: 10.1371/journal.pgen.1004561 (PMC4140648; doi:10.1371/journal.pgen.1004561)
Supplement: Table S7 — GO process analysis of top 100 signals, when compared to all signals, from CEU population using the test statistic. (PDF) [file pgen.1004561.s033.pdf]

Table S7: GO process analysis of top 100 signals, when compared to all signals, from CEU population using the T_2 test statistic.

Description	p-value	Enrichment	Genes
Interferon-gamma-mediated signaling pathway	8.3×10^{-11}	24.9	*HLA-A, HLA-B, HLA-C, HLA-DPA1, HLA-DPB1, HLA-DQA1, HLA-DQB1, HLA-DRA, HLA-DRB1*
Cellular response to interferon-gamma	5.9×10^{-10}	20.1	*HLA-A, HLA-B, HLA-C, HLA-DPA1, HLA-DPB1, HLA-DQA1, HLA-DQB1, HLA-DRA, HLA-DRB1*
Response to interferon-gamma	3.5×10^{-9}	16.5	*HLA-A, HLA-B, HLA-C, HLA-DPA1, HLA-DPB1, HLA-DQA1, HLA-DQB1, HLA-DRA, HLA-DRB1*
Antigen processing and presentation of peptide antigen	4.2×10^{-8}	10.4	*ERAP1, HLA-A, HLA-B, HLA-C, HLA-DPA1, HLA-DPB1, HLA-DQA1, HLA-DQB1, HLA-DRA, HLA-DRB1*
Antigen processing and presentation	1.5×10^{-7}	9.1	*ERAP1, HLA-A, HLA-B, HLA-C, HLA-DPA1, HLA-DPB1, HLA-DQA1, HLA-DQB1, HLA-DRA, HLA-DRB1*
Antigen processing and presentation of exogenous peptide antigen	2.8×10^{-7}	10.0	*HLA-A, HLA-B, HLA-C, HLA-DPA1, HLA-DPB1, HLA-DQA1, HLA-DQB1, HLA-DRA, HLA-DRB1*
Antigen processing and presentation of exogenous antigen	3.2×10^{-7}	9.9	*HLA-A, HLA-B, HLA-C, HLA-DPA1, HLA-DPB1, HLA-DQA1, HLA-DQB1, HLA-DRA, HLA-DRB1*
Detection of bacterium	7.0×10^{-7}	53.7	*HLA-A, HLA-B, HLA-DRB1, PGLYRP4*
T cell costimulation	1.3×10^{-6}	17.2	*HLA-DPA1, HLA-DPB1, HLA-DQA1, HLA-DQB1, HLA-DRA, HLA-DRB1*
Lymphocyte costimulation	1.4×10^{-6}	16.9	*HLA-DPA1, HLA-DPB1, HLA-DQA1, HLA-DQB1, HLA-DRA, HLA-DRB1*
Regulation of immune response	3.3×10^{-6}	4.7	*DMBT1, ERAP1, HLA-A, HLA-B, HLA-C, HLA-DPA1, HLA-DPB1, HLA-DQA1, HLA-DQB1, HLA-DRA, HLA-DRB1, MAP2K3, PGLYRP4*
T cell receptor signaling pathway	4.8×10^{-6}	13.8	*HLA-DPA1, HLA-DPB1, HLA-DQA1, HLA-DQB1, HLA-DRA, HLA-DRB1*
Cytokine-mediated signaling pathway	5.3×10^{-6}	6.1	*HLA-A, HLA-B, HLA-C, HLA-DPA1, HLA-DPB1, HLA-DQA1, HLA-DQB1, HLA-DRA, HLA-DRB1, NUP88*
Detection of biotic stimulus	8.2×10^{-6}	30.3	*HLA-A, HLA-B, HLA-DRB1, PGLYRP4*
Antigen processing and presentation of exogenous peptide antigen via MHC class II	8.6×10^{-6}	12.5	*HLA-DPA1, HLA-DPB1, HLA-DQA1, HLA-DQB1, HLA-DRA, HLA-DRB1*
Immune response-activating signal transduction	9.0×10^{-6}	7.8	*DMBT1, HLA-DPA1, HLA-DPB1, HLA-DQA1, HLA-DQB1, HLA-DRA, HLA-DRB1, MAP2K3*
Antigen processing and presentation of peptide or polysaccharide antigen via MHC class II	9.8×10^{-6}	12.2	*HLA-DPA1, HLA-DPB1, HLA-DQA1, HLA-DQB1, HLA-DRA, HLA-DRB1*
Antigen processing and presentation of peptide antigen via MHC class II	9.8×10^{-6}	12.2	*HLA-DPA1, HLA-DPB1, HLA-DQA1, HLA-DQB1, HLA-DRA, HLA-DRB1*
Immune response-regulating signaling pathway	1.4×10^{-5}	7.3	*DMBT1, HLA-DPA1, HLA-DPB1, HLA-DQA1, HLA-DQB1, HLA-DRA, HLA-DRB1, MAP2K3*
Antigen processing and presentation of exogenous peptide antigen via MHC class I TAP-independent	1.5×10^{-5}	58.2	*HLA-A, HLA-B, HLA-C*

GO categories in which false discovery rate is less than 0.01.
