# Supplementary material for: A Model-Based Approach for Identifying Signatures of Ancient Balancing Selection in Genetic Data
Source: PLoS Genet. 2014 Aug 21;10(8):e1004561. doi: 10.1371/journal.pgen.1004561 (PMC4140648; doi:10.1371/journal.pgen.1004561)
Supplement: Table S8 — GO process analysis of top 100 signals, when compared to all signals, from YRI population using the test statistic. (PDF) [file pgen.1004561.s034.pdf]

Table S8: GO process analysis of top 100 signals, when compared to all signals, from YRI population using the T_2 test statistic.

Description	p-value	Enrichment	Genes
Interferon-gamma-mediated signaling pathway	8.0×10^{-8}	19.0	*HLA-A*, *HLA-B*, *HLA-C*, *HLA-DPA1*, *HLA-DPB1*, *HLA-DQB1*, *HLA-DRB5*
Cellular response to interferon-gamma	3.6×10^{-7}	15.3	*HLA-A*, *HLA-B*, *HLA-C*, *HLA-DPA1*, *HLA-DPB1*, *HLA-DQB1*, *HLA-DRB5*
Detection of bacterium	7.6×10^{-7}	52.6	*HLA-A*, *HLA-B*, *HLA-DRB5*, *PGLYRP4*
Response to interferon-gamma	1.4×10^{-6}	12.6	*HLA-A*, *HLA-B*, *HLA-C*, *HLA-DPA1*, *HLA-DPB1*, *HLA-DQB1*, *HLA-DRB5*

GO categories in which false discovery rate is less than 0.01.
