# Supplementary material for: A Model-Based Approach for Identifying Signatures of Ancient Balancing Selection in Genetic Data
Source: PLoS Genet. 2014 Aug 21;10(8):e1004561. doi: 10.1371/journal.pgen.1004561 (PMC4140648; doi:10.1371/journal.pgen.1004561)
Supplement: Table S9 — GO function analysis of top 100 signals, when compared to all signals, from CEU population using the test statistic. (PDF) [file pgen.1004561.s035.pdf]

Table S9: GO function analysis of top 100 signals, when compared to all signals, from CEU population using the T_1 test statistic.

Description	p-value	Enrichment	Genes
MHC class II receptor activity	1.2×10^{-9}	90.1	*HLA-DPA1*, *HLA-DQA1*, *HLA-DQB1*, *HLA-DRA*, *HLA-DRB1*

GO categories in which false discovery rate is less than 0.01.
