# Supplementary material for: A Model-Based Approach for Identifying Signatures of Ancient Balancing Selection in Genetic Data
Source: PLoS Genet. 2014 Aug 21;10(8):e1004561. doi: 10.1371/journal.pgen.1004561 (PMC4140648; doi:10.1371/journal.pgen.1004561)
Supplement: Table S12 — GO component analysis of top 100 signals, when compared to all signals, from CEU population using the test statistic. (PDF) [file pgen.1004561.s038.pdf]

Table S12: GO component analysis of top 100 signals, when compared to all signals, from CEU population using the T_1 test statistic.

Description	p-value	Enrichment	Genes
Integral to lumenal side of endoplasmic reticulum membrane	5.0×10^{-17}	72.1	*HLA-A, HLA-B, HLA-C, HLA-DPA1, HLA-DPB1, HLA-DQA1, HLA-DQB1, HLA-DRA, HLA-DRB1, HLA-DRB5*
ER to Golgi transport vesicle membrane	4.5×10^{-16}	60.1	*HLA-A, HLA-B, HLA-C, HLA-DPA1, HLA-DPB1, HLA-DQA1, HLA-DQB1, HLA-DRA, HLA-DRB1, HLA-DRB5*
MHC protein complex	1.9×10^{-15}	53.0	*HLA-A, HLA-B, HLA-C, HLA-DPA1, HLA-DPB1, HLA-DQA1, HLA-DQB1, HLA-DRA, HLA-DRB1, HLA-DRB5*
Transport vesicle membrane	8.0×10^{-15}	36.1	*CPE, HLA-A, HLA-B, HLA-C, HLA-DPA1, HLA-DPB1, HLA-DQA1, HLA-DQB1, HLA-DRA, HLA-DRB1, HLA-DRB5*
MHC class II protein complex	4.2×10^{-13}	90.1	*HLA-DPA1, HLA-DPB1, HLA-DQA1, HLA-DQB1, HLA-DRA, HLA-DRB1, HLA-DRB5*
Endocytic vesicle membrane	6.2×10^{-13}	24.8	*DMBT1, HLA-A, HLA-B, HLA-C, HLA-DPA1, HLA-DPB1, HLA-DQA1, HLA-DQB1, HLA-DRA, HLA-DRB1, HLA-DRB5*
Intrinsic to endoplasmic reticulum membrane	8.6×10^{-12}	19.6	*FMO5, HLA-A, HLA-B, HLA-C, HLA-DPA1, HLA-DPB1, HLA-DQA1, HLA-DQB1, HLA-DRA, HLA-DRB1, HLA-DRB5*
Clathrin-coated endocytic vesicle membrane	2.0×10^{-11}	57.4	*HLA-DPA1, HLA-DPB1, HLA-DQA1, HLA-DQB1, HLA-DRA, HLA-DRB1, HLA-DRB5*
Integral to endoplasmic reticulum membrane	3.7×10^{-11}	21.2	*HLA-A, HLA-B, HLA-C, HLA-DPA1, HLA-DPB1, HLA-DQA1, HLA-DQB1, HLA-DRA, HLA-DRB1, HLA-DRB5*
Trans-Golgi network membrane	1.0×10^{-10}	46.7	*HLA-DPA1, HLA-DPB1, HLA-DQA1, HLA-DQB1, HLA-DRA, HLA-DRB1, HLA-DRB5*
Coated vesicle membrane	3.7×10^{-10}	16.9	*HLA-A, HLA-B, HLA-C, HLA-DPA1, HLA-DPB1, HLA-DQA1, HLA-DQB1, HLA-DRA, HLA-DRB1, HLA-DRB5*
Cytoplasmic vesicle membrane	6.4×10^{-9}	8.1	*CPE, DMBT1, HLA-A, HLA-B, HLA-C, HLA-DPA1, HLA-DPB1, HLA-DQA1, HLA-DQB1, HLA-DRA, HLA-DRB1, HLA-DRB5, SNX19*
Vesicle membrane	1.1×10^{-8}	7.8	*CPE, DMBT1, HLA-A, HLA-B, HLA-C, HLA-DPA1, HLA-DPB1, HLA-DQA1, HLA-DQB1, HLA-DRA, HLA-DRB1, HLA-DRB5, SNX19*
Intrinsic to organelle membrane	1.2×10^{-8}	10.1	*FMO5, HLA-A, HLA-B, HLA-C, HLA-DPA1, HLA-DPB1, HLA-DQA1, HLA-DQB1, HLA-DRA, HLA-DRB1, HLA-DRB5*
Integral to organelle membrane	4.7×10^{-8}	10.2	*HLA-A, HLA-B, HLA-C, HLA-DPA1, HLA-DPB1, HLA-DQA1, HLA-DQB1, HLA-DRA, HLA-DRB1, HLA-DRB5*
Cytoplasmic vesicle part	1.4×10^{-7}	6.3	*CPE, DMBT1, HLA-A, HLA-B, HLA-C, HLA-DPA1, HLA-DPB1, HLA-DQA1, HLA-DQB1, HLA-DRA, HLA-DRB1, HLA-DRB5, SNX19*
Clathrin-coated vesicle membrane	4.4×10^{-7}	14.9	*HLA-DPA1, HLA-DPB1, HLA-DQA1, HLA-DQB1, HLA-DRA, HLA-DRB1, HLA-DRB5*
Endosome membrane	3.7×10^{-6}	6.4	*HLA-A, HLA-B, HLA-C, HLA-DPA1, HLA-DPB1, HLA-DQA1, HLA-DQB1, HLA-DRA, HLA-DRB1, HLA-DRB5*
Endosomal part	5.7×10^{-6}	6.1	*HLA-A, HLA-B, HLA-C, HLA-DPA1, HLA-DPB1, HLA-DQA1, HLA-DQB1, HLA-DRA, HLA-DRB1, HLA-DRB5*
Lysosomal membrane	6.0×10^{-6}	10.1	*HLA-DPA1, HLA-DPB1, HLA-DQA1, HLA-DQB1, HLA-DRA, HLA-DRB1, HLA-DRB5*
Vacuolar membrane	1.8×10^{-5}	8.5	*HLA-DPA1, HLA-DPB1, HLA-DQA1, HLA-DQB1, HLA-DRA, HLA-DRB1, HLA-DRB5*
Phagocytic vesicle membrane	1.9×10^{-5}	24.9	*DMBT1, HLA-A, HLA-B, HLA-C*
Plasma membrane	3.7×10^{-5}	2.0	*ANK2, ANK3, APBB1IP, BNC2, CHRNB3, CPE, DLG2, EMID2, FHIT, FRAS1, GRIN2A, HLA-A, HLA-B, HLA-C, HLA-DPA1, HLA-DPB1, HLA-DQA1, HLA-DQB1, HLA-DRA, HLA-DRB1, HLA-DRB5, IGSF5, ITGA1, KCNJ12, MAGI2, OR4C3, OR4C45, OR51B6, OR51F1, OR5W2, RGS6, RIMBP2, SLC2A9, TRPC6*
Cell junction	9.6×10^{-5}	3.4	*ANK2, ANK3, APBB1IP, CDSN, CHRNB3, CTNNA3, DLG2, GRIN2A, IGSF5, MAGI2, POLR1E, RIMBP2, TRPC6*
MHC class I protein complex	1.8×10^{-4}	27.0	*HLA-A, HLA-B, HLA-C*

GO categories in which false discovery rate is less than 0.01.
