# Supplementary material for: A Model-Based Approach for Identifying Signatures of Ancient Balancing Selection in Genetic Data
Source: PLoS Genet. 2014 Aug 21;10(8):e1004561. doi: 10.1371/journal.pgen.1004561 (PMC4140648; doi:10.1371/journal.pgen.1004561)
Supplement: Table S13 — GO component analysis of top 100 signals, when compared to all signals, from YRI population using the test statistic. (PDF) [file pgen.1004561.s039.pdf]

Table S13: GO component analysis of top 100 signals, when compared to all signals, from YRI population using the T_1 test statistic.

Description	p-value	Enrichment	Genes
Integral to lumenal side of endoplasmic reticulum membrane	9.9×10^{-17}	67.6	*HLA-A, HLA-B, HLA-C, HLA-DPA1, HLA-DPB1, HLA-DQA1, HLA-DQB1, HLA-DRA, HLA-DRB1, HLA-DRB5*
ER to Golgi transport vesicle membrane	8.8×10^{-16}	56.3	*HLA-A, HLA-B, HLA-C, HLA-DPA1, HLA-DPB1, HLA-DQA1, HLA-DQB1, HLA-DRA, HLA-DRB1, HLA-DRB5*
MHC protein complex	3.8×10^{-15}	49.7	*HLA-A, HLA-B, HLA-C, HLA-DPA1, HLA-DPB1, HLA-DQA1, HLA-DQB1, HLA-DRA, HLA-DRB1, HLA-DRB5*
Transport vesicle membrane	1.7×10^{-14}	33.8	*CPE, HLA-A, HLA-B, HLA-C, HLA-DPA1, HLA-DPB1, HLA-DQA1, HLA-DQB1, HLA-DRA, HLA-DRB1, HLA-DRB5*
MHC class II protein complex	6.7×10^{-13}	84.5	*HLA-DPA1, HLA-DPB1, HLA-DQA1, HLA-DQB1, HLA-DRA, HLA-DRB1, HLA-DRB5*
Endocytic vesicle membrane	1.3×10^{-12}	23.2	*DMBT1, HLA-A, HLA-B, HLA-C, HLA-DPA1, HLA-DPB1, HLA-DQA1, HLA-DQB1, HLA-DRA, HLA-DRB1, HLA-DRB5*
Clathrin-coated endocytic vesicle membrane	3.2×10^{-11}	53.8	*HLA-DPA1, HLA-DPB1, HLA-DQA1, HLA-DQB1, HLA-DRA, HLA-DRB1, HLA-DRB5*
Integral to endoplasmic reticulum membrane	7.1×10^{-11}	19.9	*HLA-A, HLA-B, HLA-C, HLA-DPA1, HLA-DPB1, HLA-DQA1, HLA-DQB1, HLA-DRA, HLA-DRB1, HLA-DRB5*
Trans-Golgi network membrane	1.6×10^{-10}	43.8	*HLA-DPA1, HLA-DPB1, HLA-DQA1, HLA-DQB1, HLA-DRA, HLA-DRB1, HLA-DRB5*
Intrinsic to endoplasmic reticulum membrane	4.0×10^{-10}	16.7	*HLA-A, HLA-B, HLA-C, HLA-DPA1, HLA-DPB1, HLA-DQA1, HLA-DQB1, HLA-DRA, HLA-DRB1, HLA-DRB5*
Coated vesicle membrane	7.1×10^{-10}	15.8	*HLA-A, HLA-B, HLA-C, HLA-DPA1, HLA-DPB1, HLA-DQA1, HLA-DQB1, HLA-DRA, HLA-DRB1, HLA-DRB5*
Cytoplasmic vesicle membrane	1.4×10^{-8}	7.6	*CPE, DMBT1, HLA-A, HLA-B, HLA-C, HLA-DPA1, HLA-DPB1, HLA-DQA1, HLA-DQB1, HLA-DRA, HLA-DRB1, HLA-DRB5, SNX19*
Vesicle membrane	2.3×10^{-8}	7.3	*CPE, DMBT1, HLA-A, HLA-B, HLA-C, HLA-DPA1, HLA-DPB1, HLA-DQA1, HLA-DQB1, HLA-DRA, HLA-DRB1, HLA-DRB5, SNX19*
Integral to organelle membrane	8.8×10^{-8}	9.6	*HLA-A, HLA-B, HLA-C, HLA-DPA1, HLA-DPB1, HLA-DQA1, HLA-DQB1, HLA-DRA, HLA-DRB1, HLA-DRB5*
Intrinsic to organelle membrane	2.5×10^{-7}	8.6	*HLA-A, HLA-B, HLA-C, HLA-DPA1, HLA-DPB1, HLA-DQA1, HLA-DQB1, HLA-DRA, HLA-DRB1, HLA-DRB5*
Cytoplasmic vesicle part	2.9×10^{-7}	5.9	*CPE, DMBT1, HLA-A, HLA-B, HLA-C, HLA-DPA1, HLA-DPB1, HLA-DQA1, HLA-DQB1, HLA-DRA, HLA-DRB1, HLA-DRB5, SNX19*
Clathrin-coated vesicle membrane	6.9×10^{-7}	13.9	*HLA-DPA1, HLA-DPB1, HLA-DQA1, HLA-DQB1, HLA-DRA, HLA-DRB1, HLA-DRB5*
Endosome membrane	6.7×10^{-6}	6.0	*HLA-A, HLA-B, HLA-C, HLA-DPA1, HLA-DPB1, HLA-DQA1, HLA-DQB1, HLA-DRA, HLA-DRB1, HLA-DRB5*
Lysosomal membrane	9.2×10^{-6}	9.5	*HLA-DPA1, HLA-DPB1, HLA-DQA1, HLA-DQB1, HLA-DRA, HLA-DRB1, HLA-DRB5*
Endosomal part	1.0×10^{-5}	5.7	*HLA-A, HLA-B, HLA-C, HLA-DPA1, HLA-DPB1, HLA-DQA1, HLA-DQB1, HLA-DRA, HLA-DRB1, HLA-DRB5*
Phagocytic vesicle membrane	2.4×10^{-5}	23.3	*DMBT1, HLA-A, HLA-B, HLA-C*
Vacuolar membrane	2.8×10^{-5}	8.0	*HLA-DPA1, HLA-DPB1, HLA-DQA1, HLA-DQB1, HLA-DRA, HLA-DRB1, HLA-DRB5*
Vacuolar part	6.4×10^{-5}	5.9	*GALC, HLA-DPA1, HLA-DPB1, HLA-DQA1, HLA-DQB1, HLA-DRA, HLA-DRB1, HLA-DRB5*

GO categories in which false discovery rate is less than 0.01.
