# Supplementary material for: A Model-Based Approach for Identifying Signatures of Ancient Balancing Selection in Genetic Data
Source: PLoS Genet. 2014 Aug 21;10(8):e1004561. doi: 10.1371/journal.pgen.1004561 (PMC4140648; doi:10.1371/journal.pgen.1004561)
Supplement: Table S14 — GO component analysis of top 100 signals, when compared to all signals, from CEU population using the test statistic. (PDF) [file pgen.1004561.s040.pdf]

Table S14: GO component analysis of top 100 signals, when compared to all signals, from CEU population using the T_2 test statistic.

Description	p-value	Enrichment	Genes
Integral to lumenal side of endoplasmic reticulum membrane	8.5×10^{-15}	62.8	*HLA-A*, *HLA-B*, *HLA-C*, *HLA-DPA1*, *HLA-DPB1*, *HLA-DQA1*, *HLA-DQB1*, *HLA-DRA*, *HLA-DRB1*
ER to Golgi transport vesicle membrane	5.8×10^{-14}	52.3	*HLA-A*, *HLA-B*, *HLA-C*, *HLA-DPA1*, *HLA-DPB1*, *HLA-DQA1*, *HLA-DQB1*, *HLA-DRA*, *HLA-DRB1*
MHC protein complex	2.1×10^{-13}	46.2	*HLA-A*, *HLA-B*, *HLA-C*, *HLA-DPA1*, *HLA-DPB1*, *HLA-DQA1*, *HLA-DQB1*, *HLA-DRA*, *HLA-DRB1*
Transport vesicle membrane	5.5×10^{-13}	31.7	*CPE*, *HLA-A*, *HLA-B*, *HLA-C*, *HLA-DPA1*, *HLA-DPB1*, *HLA-DQA1*, *HLA-DQB1*, *HLA-DRA*, *HLA-DRB1*
Endocytic vesicle membrane	9.0×10^{-13}	24.0	*CUBN*, *DMBT1*, *HLA-A*, *HLA-B*, *HLA-C*, *HLA-DPA1*, *HLA-DPB1*, *HLA-DQA1*, *HLA-DQB1*, *HLA-DRA*, *HLA-DRB1*
MHC class II protein complex	8.7×10^{-11}	74.8	*HLA-DPA1*, *HLA-DPB1*, *HLA-DQA1*, *HLA-DQB1*, *HLA-DRA*, *HLA-DRB1*
Integral to endoplasmic reticulum membrane	1.3×10^{-9}	18.5	*HLA-A*, *HLA-B*, *HLA-C*, *HLA-DPA1*, *HLA-DPB1*, *HLA-DQA1*, *HLA-DQB1*, *HLA-DRA*, *HLA-DRB1*
Clathrin-coated endocytic vesicle membrane	2.1×10^{-9}	47.6	*HLA-DPA1*, *HLA-DPB1*, *HLA-DQA1*, *HLA-DQB1*, *HLA-DRA*, *HLA-DRB1*
Intrinsic to endoplasmic reticulum membrane	6.1×10^{-9}	15.6	*HLA-A*, *HLA-B*, *HLA-C*, *HLA-DPA1*, *HLA-DPB1*, *HLA-DQA1*, *HLA-DQB1*, *HLA-DRA*, *HLA-DRB1*
Trans-Golgi network membrane	8.1×10^{-9}	38.8	*HLA-DPA1*, *HLA-DPB1*, *HLA-DQA1*, *HLA-DQB1*, *HLA-DRA*, *HLA-DRB1*
Cytoplasmic vesicle membrane	9.7×10^{-9}	7.9	*CPE*, *CUBN*, *DMBT1*, *HLA-A*, *HLA-B*, *HLA-C*, *HLA-DPA1*, *HLA-DPB1*, *HLA-DQA1*, *HLA-DQB1*, *HLA-DRA*, *HLA-DRB1*, *SNX19*
Coated vesicle membrane	1.0×10^{-8}	14.7	*HLA-A*, *HLA-B*, *HLA-C*, *HLA-DPA1*, *HLA-DPB1*, *HLA-DQA1*, *HLA-DQB1*, *HLA-DRA*, *HLA-DRB1*
Vesicle membrane	1.6×10^{-8}	7.6	*CPE*, *CUBN*, *DMBT1*, *HLA-A*, *HLA-B*, *HLA-C*, *HLA-DPA1*, *HLA-DPB1*, *HLA-DQA1*, *HLA-DQB1*, *HLA-DRA*, *HLA-DRB1*, *SNX19*
Cytoplasmic vesicle part	2.0×10^{-7}	6.1	*CPE*, *CUBN*, *DMBT1*, *HLA-A*, *HLA-B*, *HLA-C*, *HLA-DPA1*, *HLA-DPB1*, *HLA-DQA1*, *HLA-DQB1*, *HLA-DRA*, *HLA-DRB1*, *SNX19*
Integral to organelle membrane	7.5×10^{-7}	8.9	*HLA-A*, *HLA-B*, *HLA-C*, *HLA-DPA1*, *HLA-DPB1*, *HLA-DQA1*, *HLA-DQB1*, *HLA-DRA*, *HLA-DRB1*
Intrinsic to organelle membrane	1.9×10^{-6}	8.0	*HLA-A*, *HLA-B*, *HLA-C*, *HLA-DPA1*, *HLA-DPB1*, *HLA-DQA1*, *HLA-DQB1*, *HLA-DRA*, *HLA-DRB1*
Endosome membrane	5.0×10^{-6}	6.2	*CUBN*, *HLA-A*, *HLA-B*, *HLA-C*, *HLA-DPA1*, *HLA-DPB1*, *HLA-DQA1*, *HLA-DQB1*, *HLA-DRA*, *HLA-DRB1*
Lysosomal membrane	7.4×10^{-6}	9.8	*CUBN*, *HLA-DPA1*, *HLA-DPB1*, *HLA-DQA1*, *HLA-DQB1*, *HLA-DRA*, *HLA-DRB1*
Endosomal part	7.7×10^{-6}	5.9	*CUBN*, *HLA-A*, *HLA-B*, *HLA-C*, *HLA-DPA1*, *HLA-DPB1*, *HLA-DQA1*, *HLA-DQB1*, *HLA-DRA*, *HLA-DRB1*
Clathrin-coated vesicle membrane	9.2×10^{-6}	12.3	*HLA-DPA1*, *HLA-DPB1*, *HLA-DQA1*, *HLA-DQB1*, *HLA-DRA*, *HLA-DRB1*
Phagocytic vesicle membrane	2.2×10^{-5}	24.1	*DMBT1*, *HLA-A*, *HLA-B*, *HLA-C*
Vacuolar membrane	2.2×10^{-5}	8.3	*CUBN*, *HLA-DPA1*, *HLA-DPB1*, *HLA-DQA1*, *HLA-DQB1*, *HLA-DRA*, *HLA-DRB1*
Vacuolar part	5.1×10^{-5}	6.1	*CUBN*, *GPC5*, *HLA-DPA1*, *HLA-DPB1*, *HLA-DQA1*, *HLA-DQB1*, *HLA-DRA*, *HLA-DRB1*

GO categories in which false discovery rate is less than 0.01.
