# Supplementary material for: A Model-Based Approach for Identifying Signatures of Ancient Balancing Selection in Genetic Data
Source: PLoS Genet. 2014 Aug 21;10(8):e1004561. doi: 10.1371/journal.pgen.1004561 (PMC4140648; doi:10.1371/journal.pgen.1004561)
Supplement: Table S15 — GO component analysis of top 100 signals, when compared to all signals, from YRI population using the test statistic. (PDF) [file pgen.1004561.s041.pdf]

Table S15: GO component analysis of top 100 signals, when compared to all signals, from YRI population using the T_2 test statistic.

Description	p-value	Enrichment	Genes
Integral to lumenal side of endoplasmic reticulum membrane	8.3×10^{-11}	47.8	*HLA-A*, *HLA-B*, *HLA-C*, *HLA-DPA1*, *HLA-DPB1*, *HLA-DQB1*, *HLA-DRB5*
ER to Golgi transport vesicle membrane	3.4×10^{-10}	39.9	*HLA-A*, *HLA-B*, *HLA-C*, *HLA-DPA1*, *HLA-DPB1*, *HLA-DQB1*, *HLA-DRB5*
MHC protein complex	8.9×10^{-10}	35.2	*HLA-A*, *HLA-B*, *HLA-C*, *HLA-DPA1*, *HLA-DPB1*, *HLA-DQB1*, *HLA-DRB5*
Transport vesicle membrane	9.9×10^{-10}	24.9	*CPE*, *HLA-A*, *HLA-B*, *HLA-C*, *HLA-DPA1*, *HLA-DPB1*, *HLA-DQB1*, *HLA-DRB5*
Endocytic vesicle membrane	2.1×10^{-8}	17.1	*DMBT1*, *HLA-A*, *HLA-B*, *HLA-C*, *HLA-DPA1*, *HLA-DPB1*, *HLA-DQB1*, *HLA-DRB5*
Cytoplasmic vesicle membrane	1.1×10^{-7}	7.1	*CPE*, *DMBT1*, *HLA-A*, *HLA-B*, *HLA-C*, *HLA-DPA1*, *HLA-DPB1*, *HLA-DQB1*, *HLA-DRB5*, *MARCH1*, *MYO5B*, *SNX19*
Vesicle membrane	1.8×10^{-7}	6.8	*CPE*, *DMBT1*, *HLA-A*, *HLA-B*, *HLA-C*, *HLA-DPA1*, *HLA-DPB1*, *HLA-DQB1*, *HLA-DRB5*, *MARCH1*, *MYO5B*, *SNX19*
Trans-Golgi network membrane	4.5×10^{-7}	31.6	*HLA-DPA1*, *HLA-DPB1*, *HLA-DQB1*, *HLA-DRB5*, *MARCH1*
Integral to endoplasmic reticulum membrane	6.4×10^{-7}	14.1	*HLA-A*, *HLA-B*, *HLA-C*, *HLA-DPA1*, *HLA-DPB1*, *HLA-DQB1*, *HLA-DRB5*
MHC class II protein complex	1.1×10^{-6}	48.8	*HLA-DPA1*, *HLA-DPB1*, *HLA-DQB1*, *HLA-DRB5*
Cytoplasmic vesicle part	1.8×10^{-6}	5.5	*CPE*, *DMBT1*, *HLA-B*, *HLA-C*, *HLA-DPA1*, *HLA-DPB1*, *HLA-DQB1*, *HLA-DRB5*, *MARCH1*, *MYO5B*, *SNX19*
Intrinsic to endoplasmic reticulum membrane	2.1×10^{-6}	11.8	*HLA-A*, *HLA-B*, *HLA-C*, *HLA-DPA1*, *HLA-DPB1*, *HLA-DQB1*, *HLA-DRB5*
Coated vesicle membrane	3.1×10^{-6}	11.2	*HLA-A*, *HLA-B*, *HLA-C*, *HLA-DPA1*, *HLA-DPB1*, *HLA-DQB1*, *HLA-DRB5*
Clathrin-coated endocytic vesicle membrane	7.4×10^{-6}	31.1	*HLA-DPA1*, *HLA-DPB1*, *HLA-DQB1*, *HLA-DRB5*
Phagocytic vesicle membrane	2.3×10^{-5}	23.6	*DMBT1*, *HLA-A*, *HLA-B*, *HLA-C*
Integral to organelle membrane	7.8×10^{-5}	6.8	*HLA-A*, *HLA-B*, *HLA-C*, *HLA-DPA1*, *HLA-DPB1*, *HLA-DQB1*, *HLA-DRB5*

GO categories in which false discovery rate is less than 0.01.
