# Supplementary material for: A Model-Based Approach for Identifying Signatures of Ancient Balancing Selection in Genetic Data
Source: PLoS Genet. 2014 Aug 21;10(8):e1004561. doi: 10.1371/journal.pgen.1004561 (PMC4140648; doi:10.1371/journal.pgen.1004561)
Supplement: Table S16 — GO process analysis of ranked signals from CEU population using the test statistic. (PDF) [file pgen.1004561.s042.pdf]

Table S16: GO process analysis of ranked signals from CEU population using the T_1 test statistic.

Description	p-value	Enrichment	Genes
Interferon-gamma-mediated signaling pathway	4.6×10^{-14}	108.4	*HLA-A, HLA-B, HLA-C, HLA-DPA1, HLA-DPB1, HLA-DQA1, HLA-DQB1, HLA-DRB1*
Cellular response to interferon-gamma	3.0×10^{-13}	87.6	*HLA-A, HLA-B, HLA-C, HLA-DPA1, HLA-DPB1, HLA-DQA1, HLA-DQB1, HLA-DRB1*
Biological adhesion	6.6×10^{-13}	1.9	*ACTN2, ADAM12, AJAP1, AMTN, ANGPT1, APP, B4GALT1, BMPR1B, CADM2, CD151, CDH10, CDH13, CDH23, CDH4, CDH9, CDHR2, CDON, CDSN, CFDP1, CHL1, CLCA2, CLDN1, CLDN10, CLDN14, CLDN20, CLSTN2, CNTN1, CNTN4, CNTN5, CNTNAP2, CNTNAP4, CNTNAP5, COL13A1, COL4A3, COL5A1, CPXM2, CTNNA3, CXADR, CXCL12, DAB1, DCHS2, DDR1, DPT, DSC1, DSCAM, DSG1, EMR1, EMR2, ENTPD1, EPHB1, F5, FAT2, FAT3, FAT4, FER, HABP2, HPSE, IGFBP7, IGSF5, ITGA1, ITGA11, ITGA2, ITGA9, ITGB3, ITGB5, ITGBL1, KIFC3, LAMA1, LAMA2, LAMC1, LAMC2, LOXL2, LPP, LSAMP, MAGI1, MEGF10, MEGF11, MTSS1, NCAM2, NELL2, NFASC, NID2, NINJ2, NRCAM, NRP1, NRXN1, NRXN3, NTM, NTN1, ODZ3, OPCML, PARVB, PCDH15, PCDH17, PCDHA1, PCDHA10, PCDHA11, PCDHA12, PCDHA13, PCDHA2, PCDHA3, PCDHA4, PCDHA5, PCDHA6, PCDHA7, PCDHA8, PCDHA9, PCDHAC1, PDZD2, PKD1L1, PKHD1, PKP1, PKP2, PLEK, PLEKHA7, PLXNC1, PPFIBP1, PRKCA, PRKCE, PRPH2, PTPRD, PT-PRF, PTPRM, PTPRT, PTPRU, RELN, ROBO2, ROR2, RPSA, SCARB1, SDK1, SEMA5A, SIRPA, SORBS1, SPON1, SSPO, SVEP1, SYK, TEK, THBS2, TMEM8A, TNN, TNR, VNN1, VWF*
Cell adhesion	6.6×10^{-13}	1.9	*ACTN2, ADAM12, AJAP1, AMTN, ANGPT1, APP, B4GALT1, BMPR1B, CADM2, CD151, CDH10, CDH13, CDH23, CDH4, CDH9, CDHR2, CDON, CDSN, CFDP1, CHL1, CLCA2, CLDN1, CLDN10, CLDN14, CLDN20, CLSTN2, CNTN1, CNTN4, CNTN5, CNTNAP2, CNTNAP4, CNTNAP5, COL13A1, COL4A3, COL5A1, CPXM2, CTNNA3, CXADR, CXCL12, DAB1, DCHS2, DDR1, DPT, DSC1, DSCAM, DSG1, EMR1, EMR2, ENTPD1, EPHB1, F5, FAT2, FAT3, FAT4, FER, HABP2, HPSE, IGFBP7, IGSF5, ITGA1, ITGA11, ITGA2, ITGA9, ITGB3, ITGB5, ITGBL1, KIFC3, LAMA1, LAMA2, LAMC1, LAMC2, LOXL2, LPP, LSAMP, MAGI1, MEGF10, MEGF11, MTSS1, NCAM2, NELL2, NFASC, NID2, NINJ2, NRCAM, NRP1, NRXN1, NRXN3, NTM, NTN1, ODZ3, OPCML, PARVB, PCDH15, PCDH17, PCDHA1, PCDHA10, PCDHA11, PCDHA12, PCDHA13, PCDHA2, PCDHA3, PCDHA4, PCDHA5, PCDHA6, PCDHA7, PCDHA8, PCDHA9, PCDHAC1, PDZD2, PKD1L1, PKHD1, PKP1, PKP2, PLEK, PLEKHA7, PLXNC1, PPFIBP1, PRKCA, PRKCE, PRPH2, PTPRD, PT-PRF, PTPRM, PTPRT, PTPRU, RELN, ROBO2, ROR2, RPSA, SCARB1, SDK1, SEMA5A, SIRPA, SORBS1, SPON1, SSPO, SVEP1, SYK, TEK, THBS2, TMEM8A, TNN, TNR, VNN1, VWF*
Response to interferon-gamma	1.6×10^{-12}	71.9	*HLA-A, HLA-B, HLA-C, HLA-DPA1, HLA-DQA1, HLA-DQB1, HLA-DRB1*
Antigen processing and presentation of exogenous peptide antigen	3.0×10^{-11}	43.5	*HLA-A, HLA-B, HLA-C, HLA-DPA1, HLA-DPB1, HLA-DQA1, HLA-DQB1, HLA-DRB1*
Antigen processing and presentation of exogenous antigen	3.2×10^{-11}	43.0	*HLA-A, HLA-B, HLA-C, HLA-DPA1, HLA-DPB1, HLA-DQA1, HLA-DQB1, HLA-DRB1*
Antigen processing and presentation of peptide antigen	4.1×10^{-11}	31.0	*ERAP1, HLA-A, HLA-B, HLA-C, HLA-DPA1, HLA-DPB1, HLA-DQA1, HLA-DQB1, HLA-DRB1*
Antigen processing and presentation	1.7×10^{-10}	27.2	*ERAP1, HLA-A, HLA-B, HLA-C, HLA-DPA1, HLA-DPB1, HLA-DQA1, HLA-DQB1, HLA-DRB1*
Cell-cell adhesion	1.6×10^{-9}	2.9	*BMPR1B, CDH10, CDH13, CDH4, CDSN, CLDN10, CLDN14, CNTN4, COL13A1, CTNNA3, DCHS2, DSC1, DSG1, FAT2, IGSF5, MEGF11, NRCAM, NRXN1, NTN1, ODZ3, PCDH15, PCDHA1, PCDHA10, PCDHA11, PCDHA12, PCDHA13, PCDHA2, PCDHA3, PCDHA4, PCDHA5, PCDHA6, PCDHA7, PCDHA8, PCDHA9, PCDHAC1, PKD1L1, PKHD1, PLEK, PTPRD, PTPRM, ROBO2, SYK, TEK, VNN1*
Regulation of immune response	2.1×10^{-9}	13.3	*DMBT1, ERAP1, HLA-A, HLA-B, HLA-C, HLA-DPA1, HLA-DPB1, HLA-DQA1, HLA-DQB1, HLA-DRB1, MAP2K3*
Antigen processing and presentation of exogenous peptide antigen via MHC class I TAP-independent	2.8×10^{-9}	1352.2	*HLA-A, HLA-B, HLA-C*
Cytokine-mediated signaling pathway	4.2×10^{-9}	24.0	*HLA-A, HLA-B, HLA-C, HLA-DPA1, HLA-DPB1, HLA-DQA1, HLA-DQB1, HLA-DRB1*
Homophilic cell adhesion	9.5×10^{-9}	4.1	*CDH10, CDH13, CDH4, DCHS2, DSC1, DSG1, FAT2, ODZ3, PCDH15, PCDHA1, PCDHA10, PCDHA11, PCDHA12, PCDHA13, PCDHA2, PCDHA3, PCDHA4, PCDHA5, PCDHA6, PCDHA7, PCDHA8, PCDHA9, PCDHAC1, PTPRM, ROBO2*
Cellular response to cytokine stimulus	3.3×10^{-8}	18.9	*HLA-A, HLA-B, HLA-C, HLA-DPA1, HLA-DPB1, HLA-DQA1, HLA-DQB1, HLA-DRB1*
Immune response-activating signal transduction	4.8×10^{-8}	25.2	*DMBT1, HLA-DPA1, HLA-DPB1, HLA-DQA1, HLA-DQB1, HLA-DRB1, MAP2K3*

GO categories in which false discovery rate is less than 0.01.

Table S16 continued.

Description	p-value	Enrichment	Genes
Immune response-regulating signaling pathway	7.4×10^{-8}	23.7	*DMBT1*, *HLA-DPA1*, *HLA-DPB1*, *HLA-DQA1*, *HLA-DQB1*, *HLA-DRB1*, *MAP2K3*
T cell costimulation	9.5×10^{-8}	27.0	*HLA-DPA1*, *HLA-DPB1*, *HLA-DQA1*, *HLA-DQB1*, *HLA-DRA*, *HLA-DRB1*, *HLA-DRB5*
Lymphocyte costimulation	1.1×10^{-7}	26.6	*HLA-DPA1*, *HLA-DPB1*, *HLA-DQA1*, *HLA-DQB1*, *HLA-DRA*, *HLA-DRB1*, *HLA-DRB5*
Response to cytokine stimulus	1.9×10^{-7}	19.5	*HLA-A*, *HLA-B*, *HLA-C*, *HLA-DPA1*, *HLA-DPB1*, *HLA-DQA1*, *HLA-DQB1*
Detection of bacterium	2.1×10^{-7}	49.9	*HLA-A*, *HLA-B*, *HLA-DRB1*, *HLA-DRB5*, *PGLYRP4*
Activation of immune response	2.4×10^{-7}	20.1	*DMBT1*, *HLA-DPA1*, *HLA-DPB1*, *HLA-DQA1*, *HLA-DQB1*, *HLA-DRB1*, *MAP2K3*
T cell receptor signaling pathway	3.2×10^{-7}	56.2	*HLA-DPA1*, *HLA-DPB1*, *HLA-DQA1*, *HLA-DQB1*, *HLA-DRB1*
Regulation of immune system process	4.5×10^{-7}	8.0	*DMBT1*, *ERAP1*, *HLA-A*, *HLA-B*, *HLA-C*, *HLA-DPA1*, *HLA-DPB1*, *HLA-DQA1*, *HLA-DQB1*, *HLA-DRB1*, *MAP2K3*
Antigen processing and presentation of exogenous peptide antigen via MHC class II	5.2×10^{-7}	50.8	*HLA-DPA1*, *HLA-DPB1*, *HLA-DQA1*, *HLA-DQB1*, *HLA-DRB1*
Antigen processing and presentation of peptide or polysaccharide antigen via MHC class II	6.0×10^{-7}	49.7	*HLA-DPA1*, *HLA-DPB1*, *HLA-DQA1*, *HLA-DQB1*, *HLA-DRB1*
Antigen processing and presentation of peptide antigen via MHC class II	6.0×10^{-7}	49.7	*HLA-DPA1*, *HLA-DPB1*, *HLA-DQA1*, *HLA-DQB1*, *HLA-DRB1*
Regulation of potassium ion transport	1.1×10^{-6}	13.1	*ANK2*, *ANK3*, *CASQ2*, *DPP6*, *NEDD4*, *NEDD4L*, *PLCB4*, *STK39*
Antigen receptor-mediated signaling pathway	1.2×10^{-6}	43.6	*HLA-DPA1*, *HLA-DPB1*, *HLA-DQA1*, *HLA-DQB1*, *HLA-DRB1*
Positive regulation of immune response	1.6×10^{-6}	15.4	*DMBT1*, *HLA-DPA1*, *HLA-DPB1*, *HLA-DQA1*, *HLA-DQB1*, *HLA-DRB1*, *MAP2K3*
Immune response-activating cell surface receptor signaling pathway	1.6×10^{-6}	41.1	*HLA-DPA1*, *HLA-DPB1*, *HLA-DQA1*, *HLA-DQB1*, *HLA-DRB1*
Protein localization to membrane	1.9×10^{-6}	13.1	*ANK2*, *ANK3*, *CPE*, *DLG2*, *MAGI2*, *RAMP3*, *RELN*, *SCP2*
Immune response-regulating cell surface receptor signaling pathway	2.6×10^{-6}	37.5	*HLA-DPA1*, *HLA-DPB1*, *HLA-DQA1*, *HLA-DQB1*, *HLA-DRB1*
Cellular response to type I interferon	2.9×10^{-6}	190.2	*HLA-A*, *HLA-B*, *HLA-C*
Type I interferon-mediated signaling pathway	2.9×10^{-6}	190.2	*HLA-A*, *HLA-B*, *HLA-C*
Response to type I interferon	3.0×10^{-6}	187.2	*HLA-A*, *HLA-B*, *HLA-C*
Immune response	3.2×10^{-6}	8.9	*DMBT1*, *HLA-A*, *HLA-B*, *HLA-C*, *HLA-DPA1*, *HLA-DQA1*, *HLA-DQB1*, *HLA-DRB1*, *MAP2K3*
Positive regulation of T cell activation	4.0×10^{-6}	27.7	*HLA-DPA1*, *HLA-DPB1*, *HLA-DQA1*, *HLA-DQB1*, *HLA-DRB1*
Antigen processing and presentation of exogenous peptide antigen via MHC class I TAP-dependent	4.4×10^{-6}	169.0	*HLA-A*, *HLA-B*, *HLA-C*
Antigen processing and presentation of exogenous peptide antigen via MHC class I	5.0×10^{-6}	160.1	*HLA-A*, *HLA-B*, *HLA-C*
Positive regulation of ion transmembrane transporter activity	5.1×10^{-6}	17.8	*ANK2*, *ANK3*, *PLCG2*, *RELN*, *RYR2*, *TRPC6*
Detection of biotic stimulus	6.0×10^{-6}	28.2	*HLA-A*, *HLA-B*, *HLA-DRB1*, *HLA-DRB5*, *PGLYRP4*
Immunoglobulin production involved in immunoglobulin mediated immune response	6.6×10^{-6}	117.6	*HLA-DQB1*, *HLA-DRB1*, *HLA-DRB5*
Humoral immune response mediated by circulating immunoglobulin	6.6×10^{-6}	117.6	*HLA-DQB1*, *HLA-DRB1*, *HLA-DRB5*
Regulation of response to stimulus	9.9×10^{-6}	3.5	*DMBT1*, *ERAP1*, *FXN*, *HLA-A*, *HLA-B*, *HLA-C*, *HLA-DPA1*, *HLA-DPB1*, *HLA-DQA1*, *HLA-DQB1*, *HLA-DRB1*, *KALRN*, *MAGI2*, *MAP2K3*, *RGL1*, *RGS6*, *USP20*, *WWTR1*
Antigen processing and presentation of peptide antigen via MHC class I	9.9×10^{-6}	132.3	*HLA-A*, *HLA-B*, *HLA-C*
Regulation of potassium ion transmembrane transporter activity	1.0×10^{-5}	35.3	*ANK2*, *CASQ2*, *NEDD4*, *NEDD4L*
Positive regulation of lymphocyte activation	1.4×10^{-5}	22.1	*HLA-DPA1*, *HLA-DPB1*, *HLA-DQA1*, *HLA-DQB1*, *HLA-DRB1*
Regulation of ion transmembrane transporter activity	1.6×10^{-5}	8.2	*ANK2*, *ANK3*, *CASQ2*, *NEDD4*, *NEDD4L*, *PLCG2*, *RELN*, *RYR2*, *TRPC6*
Immunoglobulin production	1.9×10^{-5}	88.2	*HLA-DQB1*, *HLA-DRB1*, *HLA-DRB5*
Regulation of T cell activation	1.9×10^{-5}	20.7	*HLA-DPA1*, *HLA-DPB1*, *HLA-DQA1*, *HLA-DQB1*, *HLA-DRB1*
Regulation of membrane potential	2.1×10^{-5}	2.4	*ABCB5*, *ACTN2*, *ADRA1A*, *ANK2*, *ANK3*, *CACNA1A*, *CASQ2*, *CNR2*, *DPP6*, *GRID2*, *GRIK2*, *GRIK3*, *GRIN2A*, *GRIN2B*, *KCNIP1*, *KCNMB2*, *KCNMB3*, *LRRK2*, *NEDD4*, *NEDD4L*, *NRXN1*, *PEX5L*, *PID1*, *PKP2*, *RELN*, *RYR2*, *SCN10A*, *SCN1A*, *SLC1A6*, *SNCA*
Axon guidance	2.1×10^{-5}	1.9	*ANK2*, *ANK3*, *BMPR1B*, *CAP1*, *CDH4*, *CHL1*, *CNTN1*, *CNTN4*, *COL4A1*, *COL4A2*, *COL4A3*, *COL5A2*, *COL9A1*, *CXCL12*, *DCC*, *DOCK1*, *EPHA7*, *EPHB1*, *ETV1*, *ITGA1*, *ITGA2*, *ITGA9*, *ITGB3*, *KCNQ3*, *LAMA1*, *LAMA2*, *LAMC1*, *LMX1A*, *MATN2*, *MYO10*, *NCK2*, *NFASC*, *NRCAM*, *NRP1*, *NRXN1*, *NRXN3*, *NTN1*, *NTN4*, *PLXNA4*, *PLXNC1*, *PTPRM*, *RAC2*, *RELN*, *ROBO2*, *RPS6KA2*, *SCN3B*, *SEMA3A*, *SEMA3E*, *SEMA5A*, *SEMA6D*, *SH3GL2*, *SLIT3*, *SPTB*, *TNR*, *TRPC4*, *TRPC6*, *UNC5C*, *VAV2*
Positive regulation of leukocyte activation	2.2×10^{-5}	20.2	*HLA-DPA1*, *HLA-DPB1*, *HLA-DQA1*, *HLA-DQB1*, *HLA-DRB1*
Response to bacterium	2.5×10^{-5}	19.3	*DMBT1*, *ERAP1*, *HLA-A*, *HLA-B*, *HLA-DRB1*

GO categories in which false discovery rate is less than 0.01.

Table S16 continued.

Description	p-value	Enrichment	Genes
Positive regulation of cell activation	2.6×10^{-5}	19.5	*HLA-DPA1*, *HLA-DPB1*, *HLA-DQA1*, *HLA-DQB1*, *HLA-DRB1*
Ion transport	2.7×10^{-5}	1.5	*ADD2*, *ADRA1A*, *AKR1C4*, *ANK2*, *ANO1*, *ANXA6*, *AQP8*, *AQP9*, *ASIC2*, *ATP10B*, *ATP10D*, *ATP12A*, *ATP1A4*, *ATP1B3*, *ATP2C2*, *ATP5C1*, *ATP6V0E2*, *ATP6V1C2*, *ATP6V1G2*, *ATP8A1*, *ATP8A2*, *ATP8B1*, *ATP9A*, *BSPRY*, *CACNA1A*, *CACNA2D1*, *CACNB2*, *CAMK2D*, *CASR*, *CDH23*, *CLIC5*, *CPLX1*, *EFCAB4B*, *ENPP1*, *FGF2*, *FXN*, *GABRB2*, *GABRG3*, *GABRR1*, *GLRA3*, *GRIN3A*, *ITPR1*, *KCNA6*, *KCNAB1*, *KCNB2*, *KCNJ12*, *KCNJ16*, *KCNK2*, *KCNMB1*, *KCNMB2*, *KCNMB3*, *KCNQ3*, *KCNQ4*, *KLHL3*, *LHCGR*, *LTF*, *LYN*, *NEDD4L*, *NMUR1*, *NMUR2*, *NTRK2*, *OCA2*, *PIEZO1*, *PIEZO2*, *PLA2G4A*, *PLCG2*, *PLCZ1*, *PRKAB2*, *PRKAG2*, *PRKCE*, *RAMP3*, *RIMS1*, *RYR1*, *RYR2*, *RYR3*, *SCARA5*, *SCN10A*, *SCN1A*, *SCN3A*, *SCN3B*, *SCN9A*, *SCNN1G*, *SLC10A2*, *SLC12A6*, *SLC12A8*, *SLC15A2*, *SLC17A5*, *SLC1A2*, *SLC1A6*, *SLC22A16*, *SLC22A4*, *SLC22A5*, *SLC24A4*, *SLC25A37*, *SLC27A6*, *SLC30A9*, *SLC31A2*, *SLC38A4*, *SLC38A8*, *SLC38A9*, *SLC39A11*, *SLC39A14*, *SLC39A8*, *SLC4A7*, *SLC5A12*, *SLC7A7*, *SLC8A1*, *SLC8A3*, *SLC9A4*, *SLC9A8*, *SLC9A9*, *SLC9C1*, *SLCO1B1*, *SLCO1B3*, *SLCO1C1*, *SNCA*, *SYK*, *TG*, *TRPA1*, *TRPC4*, *TRPC6*, *TRPM3*
Immune system process	2.9×10^{-5}	7.5	*DMBT1*, *HLA-A*, *HLA-B*, *HLA-C*, *HLA-DPA1*, *HLA-DPB1*, *HLA-DQA1*, *HLA-DQB1*
Regulation of transmembrane transporter activity	3.0×10^{-5}	7.6	*ANK2*, *ANK3*, *CASQ2*, *NEDD4*, *NEDD4L*, *PLCG2*, *RELN*, *RYR2*, *TRPC6*
Positive regulation of transporter activity	3.9×10^{-5}	13.2	*ANK2*, *ANK3*, *PLCG2*, *RELN*, *RYR2*, *TRPC6*
Regulation of potassium ion transmembrane transport	4.0×10^{-5}	27.4	*ANK2*, *CASQ2*, *NEDD4*, *NEDD4L*
Positive regulation of immune system process	4.4×10^{-5}	9.6	*DMBT1*, *HLA-DPA1*, *HLA-DPB1*, *HLA-DQA1*, *HLA-DQB1*, *HLA-DRB1*, *MAP2K3*
Regulation of transporter activity	4.8×10^{-5}	3.2	*ANK2*, *ANK3*, *CAMK2D*, *CASQ2*, *CNIH3*, *DAPK1*, *EFCAB4B*, *MYO5A*, *NEDD4*, *NEDD4L*, *NRXN1*, *PLCG2*, *PON1*, *RELN*, *RYR2*, *SNCA*, *TESC*, *TRDN*, *TRPC6*
Phospholipid transport	5.4×10^{-5}	12.7	*ABCA1*, *ABCA4*, *ATP10D*, *ATP8A1*, *SCARB1*, *SCP2*

GO categories in which false discovery rate is less than 0.01.
