# Supplementary material for: A Model-Based Approach for Identifying Signatures of Ancient Balancing Selection in Genetic Data
Source: PLoS Genet. 2014 Aug 21;10(8):e1004561. doi: 10.1371/journal.pgen.1004561 (PMC4140648; doi:10.1371/journal.pgen.1004561)
Supplement: Table S17 — GO process analysis of ranked signals from YRI population using the test statistic. (PDF) [file pgen.1004561.s043.pdf]

Table S17: GO process analysis of ranked signals from YRI population using the T_1 test statistic.

Description	p-value	Enrichment	Genes
Interferon-gamma-mediated signaling pathway	2.0×10^{-15}	110.4	*HLA-A*, *HLA-B*, *HLA-C*, *HLA-DPA1*, *HLA-DPB1*, *HLA-DQA1*, *HLA-DQB1*, *HLA-DRB1*, *HLA-DRB5*
Cellular response to interferon-gamma	1.9×10^{-14}	89.2	*HLA-A*, *HLA-B*, *HLA-C*, *HLA-DPA1*, *HLA-DPB1*, *HLA-DQA1*, *HLA-DQB1*, *HLA-DRB1*, *HLA-DRB5*
Response to interferon-gamma	3.0×10^{-14}	105.1	*HLA-A*, *HLA-B*, *HLA-C*, *HLA-DPA1*, *HLA-DPB1*, *HLA-DQA1*, *HLA-DQB1*, *HLA-DRB5*
Antigen processing and presentation of exogenous peptide antigen	5.6×10^{-13}	63.6	*HLA-A*, *HLA-B*, *HLA-C*, *HLA-DPA1*, *HLA-DPB1*, *HLA-DQA1*, *HLA-DQB1*, *HLA-DRB5*
Antigen processing and presentation of exogenous antigen	6.7×10^{-13}	62.8	*HLA-A*, *HLA-B*, *HLA-C*, *HLA-DPA1*, *HLA-DPB1*, *HLA-DQA1*, *HLA-DQB1*, *HLA-DRB5*
Antigen processing and presentation of peptide antigen	1.1×10^{-12}	59.4	*HLA-A*, *HLA-B*, *HLA-C*, *HLA-DPA1*, *HLA-DPB1*, *HLA-DQA1*, *HLA-DQB1*, *HLA-DRB5*
Antigen processing and presentation	3.2×10^{-12}	52.0	*HLA-A*, *HLA-B*, *HLA-C*, *HLA-DPA1*, *HLA-DPB1*, *HLA-DQA1*, *HLA-DQB1*, *HLA-DRB5*
Cytokine-mediated signaling pathway	8.8×10^{-11}	35.0	*HLA-A*, *HLA-B*, *HLA-C*, *HLA-DPA1*, *HLA-DPB1*, *HLA-DQA1*, *HLA-DQB1*, *HLA-DRB5*
Biological adhesion	4.4×10^{-10}	1.8	*ADAM12*, *AJAP1*, *AMBN*, *AMTN*, *BMPR1B*, *CADM2*, *CD84*, *CDH10*, *CDH13*, *CDH18*, *CDH23*, *CDH4*, *CDH7*, *CDH9*, *CDHR2*, *CDON*, *CDSN*, *CELSR1*, *CELSR2*, *CFDP1*, *CHL1*, *CLCA2*, *CLDN10*, *CLDN14*, *CLDN23*, *CLSTN2*, *CNTN1*, *CNTN3*, *CNTN4*, *CNTN5*, *CNTN6*, *CNTNAP2*, *CNTNAP4*, *CNTNAP5*, *COL11A1*, *COL13A1*, *COL2A1*, *COL4A3*, *COL5A1*, *CTNNA2*, *CTNNA3*, *CTNND2*, *CXADR*, *CXCL12*, *DAB1*, *DPT*, *DSC1*, *DSC3*, *DSCAM*, *DSG1*, *DSG3*, *EMR1*, *EMR2*, *EPHA3*, *EPHA4*, *EPHB1*, *F5*, *FAT2*, *FAT3*, *FREM1*, *HABP2*, *HPSE*, *HSPB11*, *IGFBP7*, *IGSF5*, *ITGA1*, *ITGA10*, *ITGA11*, *ITGA8*, *ITGA9*, *ITGB3*, *ITGB3BP*, *ITGB5*, *ITGBL1*, *LAMA2*, *LAMA4*, *LAMC1*, *LAMC2*, *LOXL2*, *LPP*, *MAEA*, *MAGI1*, *MTSS1*, *MUC16*, *NCAM2*, *NELL2*, *NFASC*, *NID2*, *NLGN1*, *NRCAM*, *NRXN1*, *NRXN3*, *NTM*, *ODZ3*, *OPCML*, *PARVA*, *PCDH15*, *PECAM1*, *PKD1L1*, *PKHD1*, *PKP2*, *PLEK*, *PLXNC1*, *PPFIBP1*, *PRKCA*, *PRKCE*, *PTPRD*, *PTPRF*, *PTPRM*, *PTPRT*, *RELN*, *ROBO2*, *ROR2*, *RPSA*, *SCARB1*, *SDK1*, *SELL*, *SEMA5A*, *SIRPA*, *SORBS1*, *SPOCK1*, *SYK*, *TEK*, *THBS2*, *TNN*, *TNR*, *VNN1*, *VWF*
Cell adhesion	4.4×10^{-10}	1.8	*ADAM12*, *AJAP1*, *AMBN*, *AMTN*, *BMPR1B*, *CADM2*, *CD84*, *CDH10*, *CDH13*, *CDH18*, *CDH23*, *CDH4*, *CDH7*, *CDH9*, *CDHR2*, *CDON*, *CDSN*, *CELSR1*, *CELSR2*, *CFDP1*, *CHL1*, *CLCA2*, *CLDN10*, *CLDN14*, *CLDN23*, *CLSTN2*, *CNTN1*, *CNTN3*, *CNTN4*, *CNTN5*, *CNTN6*, *CNTNAP2*, *CNTNAP4*, *CNTNAP5*, *COL11A1*, *COL13A1*, *COL2A1*, *COL4A3*, *COL5A1*, *CTNNA2*, *CTNNA3*, *CTNND2*, *CXADR*, *CXCL12*, *DAB1*, *DPT*, *DSC1*, *DSC3*, *DSCAM*, *DSG1*, *DSG3*, *EMR1*, *EMR2*, *EPHA3*, *EPHA4*, *EPHB1*, *F5*, *FAT2*, *FAT3*, *FREM1*, *HABP2*, *HPSE*, *HSPB11*, *IGFBP7*, *IGSF5*, *ITGA1*, *ITGA10*, *ITGA11*, *ITGA8*, *ITGA9*, *ITGB3*, *ITGB3BP*, *ITGB5*, *ITGBL1*, *LAMA2*, *LAMA4*, *LAMC1*, *LAMC2*, *LOXL2*, *LPP*, *MAEA*, *MAGI1*, *MTSS1*, *MUC16*, *NCAM2*, *NELL2*, *NFASC*, *NID2*, *NLGN1*, *NRCAM*, *NRXN1*, *NRXN3*, *NTM*, *ODZ3*, *OPCML*, *PARVA*, *PCDH15*, *PECAM1*, *PKD1L1*, *PKHD1*, *PKP2*, *PLEK*, *PLXNC1*, *PPFIBP1*, *PRKCA*, *PRKCE*, *PTPRD*, *PTPRF*, *PTPRM*, *PTPRT*, *RELN*, *ROBO2*, *ROR2*, *RPSA*, *SCARB1*, *SDK1*, *SELL*, *SEMA5A*, *SIRPA*, *SORBS1*, *SPOCK1*, *SYK*, *TEK*, *THBS2*, *TNN*, *TNR*, *VNN1*, *VWF*
Regulation of immune response	4.6×10^{-10}	14.9	*DMBT1*, *HLA-A*, *HLA-B*, *HLA-C*, *HLA-DPA1*, *HLA-DPB1*, *HLA-DQA1*, *HLA-DQB1*, *HLA-DRB1*, *HLA-DRB5*, *MAP2K3*
Cellular response to cytokine stimulus	6.6×10^{-10}	27.6	*HLA-A*, *HLA-B*, *HLA-C*, *HLA-DPA1*, *HLA-DPB1*, *HLA-DQA1*, *HLA-DQB1*, *HLA-DRB5*
Immune response-activating signal transduction	1.4×10^{-9}	28.9	*DMBT1*, *HLA-DPA1*, *HLA-DPB1*, *HLA-DQA1*, *HLA-DQB1*, *HLA-DRB1*, *HLA-DRB5*, *MAP2K3*
T cell costimulation	1.8×10^{-9}	76.0	*HLA-DPA1*, *HLA-DPB1*, *HLA-DQA1*, *HLA-DQB1*, *HLA-DRB1*, *HLA-DRB5*
Lymphocyte costimulation	2.0×10^{-9}	74.8	*HLA-DPA1*, *HLA-DPB1*, *HLA-DQA1*, *HLA-DQB1*, *HLA-DRB1*, *HLA-DRB5*
Immune response-regulating signaling pathway	2.3×10^{-9}	27.0	*DMBT1*, *HLA-DPA1*, *HLA-DPB1*, *HLA-DQA1*, *HLA-DQB1*, *HLA-DRB1*, *HLA-DRB5*, *MAP2K3*
Response to cytokine stimulus	3.8×10^{-9}	22.2	*HLA-A*, *HLA-B*, *HLA-C*, *HLA-DPA1*, *HLA-DPB1*, *HLA-DQA1*, *HLA-DQB1*, *HLA-DRB5*
T cell receptor signaling pathway	7.2×10^{-9}	61.0	*HLA-DPA1*, *HLA-DPB1*, *HLA-DQA1*, *HLA-DQB1*, *HLA-DRB1*, *HLA-DRB5*
Activation of immune response	8.0×10^{-9}	23.0	*DMBT1*, *HLA-DPA1*, *HLA-DPB1*, *HLA-DQA1*, *HLA-DQB1*, *HLA-DRB1*, *HLA-DRB5*, *MAP2K3*
Detection of bacterium	1.1×10^{-8}	237.8	*HLA-A*, *HLA-B*, *HLA-DRB1*, *HLA-DRB5*
Antigen processing and presentation of exogenous peptide antigen via MHC class II	1.4×10^{-8}	55.2	*HLA-DPA1*, *HLA-DPB1*, *HLA-DQA1*, *HLA-DQB1*, *HLA-DRB1*, *HLA-DRB5*
Antigen processing and presentation of exogenous peptide antigen via MHC class I TAP-independent	1.5×10^{-8}	901.5	*HLA-A*, *HLA-B*, *HLA-C*

GO categories in which false discovery rate is less than 0.01.

Table S17 continued.

Description	p-value	Enrichment	Genes
Antigen processing and presentation of peptide or polysaccharide antigen via MHC class II	1.6×10^{-8}	53.9	*HLA-DPA1, HLA-DPB1, HLA-DQA1, HLA-DQB1, HLA-DRB1, HLA-DRB5*
Antigen processing and presentation of peptide antigen via MHC class II	1.6×10^{-8}	53.9	*HLA-DPA1, HLA-DPB1, HLA-DQA1, HLA-DQB1, HLA-DRB1, HLA-DRB5*
Antigen receptor-mediated signaling pathway	3.7×10^{-8}	47.3	*HLA-DPA1, HLA-DPB1, HLA-DQA1, HLA-DQB1, HLA-DRB1, HLA-DRB5*
Immune response-activating cell surface receptor signaling pathway	5.3×10^{-8}	44.6	*HLA-DPA1, HLA-DPB1, HLA-DQA1, HLA-DQB1, HLA-DRB1, HLA-DRB5*
Positive regulation of immune response	7.2×10^{-8}	17.6	*DMBT1, HLA-DPA1, HLA-DPB1, HLA-DQA1, HLA-DQB1, HLA-DRB1, HLA-DRB5, MAP2K3*
Immune response-regulating cell surface receptor signaling pathway	9.4×10^{-8}	40.7	*HLA-DPA1, HLA-DPB1, HLA-DQA1, HLA-DQB1, HLA-DRB1, HLA-DRB5*
Regulation of immune system process	1.1×10^{-7}	9.0	*DMBT1, HLA-A, HLA-B, HLA-C, HLA-DPA1, HLA-DPB1, HLA-DQA1, HLA-DQB1, HLA-DRB1, HLA-DRB5, MAP2K3*
Immunoglobulin production involved in immunoglobulin mediated immune response	1.5×10^{-7}	386.4	*HLA-DQB1, HLA-DRB1, HLA-DRB5*
Humoral immune response mediated by circulating immunoglobulin	1.5×10^{-7}	386.4	*HLA-DQB1, HLA-DRB1, HLA-DRB5*
Detection of biotic stimulus	1.6×10^{-7}	134.4	*HLA-A, HLA-B, HLA-DRB1, HLA-DRB5*
Positive regulation of T cell activation	1.8×10^{-7}	30.1	*HLA-DPA1, HLA-DPB1, HLA-DQA1, HLA-DQB1, HLA-DRB1, HLA-DRB5*
Immune response	2.5×10^{-7}	9.9	*DMBT1, HLA-A, HLA-B, HLA-C, HLA-DPA1, HLA-DQA1, HLA-DQB1, HLA-DRB1, HLA-DRB5, MAP2K3*
Immunoglobulin production	5.1×10^{-7}	289.8	*HLA-DQB1, HLA-DRB1, HLA-DRB5*
Positive regulation of lymphocyte activation	7.7×10^{-7}	24.0	*HLA-DPA1, HLA-DPB1, HLA-DQA1, HLA-DQB1, HLA-DRB1, HLA-DRB5*
Regulation of T cell activation	1.1×10^{-6}	22.5	*HLA-DPA1, HLA-DPB1, HLA-DQA1, HLA-DQB1, HLA-DRB1, HLA-DRB5*
Positive regulation of leukocyte activation	1.3×10^{-6}	22.0	*HLA-DPA1, HLA-DPB1, HLA-DQA1, HLA-DQB1, HLA-DRB1, HLA-DRB5*
Regulation of action potential	1.4×10^{-6}	5.5	*ADRA1A, ANK2, ANK3, CNR2, DPP6, GRIK2, GRIN2A, KCNC2, KCNIP1, KCNMB2, KCNMB3, PKP2, RYR2, SCN9A*
Regulation of ion transmembrane transporter activity	1.6×10^{-6}	4.0	*ANK2, ANK3, CACNG8, CAMK2D, CASQ2, CNIH3, DAPK1, JPH3, MYO5A, NEDD4, NEDD4L, NLGN1, NRXN1, PLCG2, RELN, RYR2, TESC, TRPC6, WWP2*
Positive regulation of cell activation	1.6×10^{-6}	21.2	*HLA-DPA1, HLA-DPB1, HLA-DQA1, HLA-DQB1, HLA-DRB1, HLA-DRB5*
Regulation of transporter activity	2.4×10^{-6}	3.3	*ACTN4, ANK2, ANK3, CACNG8, CAMK2D, CASQ2, CNIH3, DAPK1, JPH3, MYO5A, NDFIP2, NEDD4, NEDD4L, NLGN1, NRXN1, PLCG2, PON1, RELN, RYR2, SNCA, TESC, TRPC6, WWP2*
Positive regulation of immune system process	3.0×10^{-6}	11.0	*DMBT1, HLA-DPA1, HLA-DPB1, HLA-DQA1, HLA-DQB1, HLA-DRB1, HLA-DRB5, MAP2K3*
Production of molecular mediator of immune response	3.2×10^{-6}	178.3	*HLA-DQB1, HLA-DRB1, HLA-DRB5*
Axon guidance	3.6×10^{-6}	1.9	*ABLIM1, ABLIM3, ANK1, ANK2, ANK3, BMPR1B, CACNA1C, CAP1, CDH4, CHL1, CNTN1, CNTN4, CNTN6, COL2A1, COL4A1, COL4A2, COL4A3, COL4A4, COL5A1, COL9A1, CXCL12, DCC, DOCK1, DPYSL3, EFNA5, EPHA4, EPHA7, EPHB1, ETV1, EXT1, GLI2, ITGA1, ITGA9, ITGB3, KLF7, LAMA2, LAMC1, MYO10, NFASC, NRCAM, NRXN1, NRXN3, NTN4, PAK1, PLXNA4, PLXNC1, PTPRM, RAC2, RELN, ROBO2, RPS6KA2, SEMA3A, SEMA3E, SEMA5A, SH3GL2, SLIT3, SPTB, ST8SIA2, TNR, TRPC6, UNC5D, VAV2*
Positive regulation of ion transmembrane transporter activity	3.7×10^{-6}	18.9	*ANK2, ANK3, PLCG2, RELN, RYR2, TRPC6*
Regulation of lymphocyte activation	5.2×10^{-6}	17.4	*HLA-DPA1, HLA-DPB1, HLA-DQA1, HLA-DQB1, HLA-DRB1, HLA-DRB5*
Regulation of transmembrane transporter activity	5.5×10^{-6}	3.7	*ANK2, ANK3, CACNG8, CAMK2D, CASQ2, CNIH3, DAPK1, JPH3, MYO5A, NEDD4, NEDD4L, NLGN1, NRXN1, PLCG2, RELN, RYR2, TESC, TRPC6, WWP2*
Response to bacterium	5.9×10^{-6}	25.8	*DMBT1, HLA-A, HLA-B, HLA-DRB1, HLA-DRB5*
Cellular response to organic substance	6.0×10^{-6}	8.9	*HLA-A, HLA-B, HLA-C, HLA-DPA1, HLA-DPB1, HLA-DQA1, HLA-DQB1, HLA-DRB5*
Immunoglobulin mediated immune response	7.8×10^{-6}	144.9	*HLA-DQB1, HLA-DRB1, HLA-DRB5*
Protein localization to membrane	8.4×10^{-6}	8.7	*ANK2, ANK3, CPE, DLG2, MAGI2, NLGN1, RAMP3, RELN, SCP2*
Immune system process	1.1×10^{-5}	5.8	*DMBT1, HLA-A, HLA-B, HLA-C, HLA-DPA1, HLA-DPB1, HLA-DQA1, HLA-DQB1, HLA-DRB1, HLA-DRB5, MAP2K3*
Cellular response to type I interferon	1.3×10^{-5}	126.8	*HLA-A, HLA-B, HLA-C*
Type I interferon-mediated signaling pathway	1.3×10^{-5}	126.8	*HLA-A, HLA-B, HLA-C*
Regulation of leukocyte activation	1.3×10^{-5}	15.2	*HLA-DPA1, HLA-DPB1, HLA-DQA1, HLA-DQB1, HLA-DRB1, HLA-DRB5*
Response to type I interferon	1.4×10^{-5}	124.8	*HLA-A, HLA-B, HLA-C*
Regulation of Rho protein signal transduction	1.6×10^{-5}	3.1	*ABCA1, ADRA1A, ARAP3, ARHGEF18, ARHGEF37, ARHGEF4, DLC1, ECT2L, FARP1, FLOT1, KALRN, KANK1, MCF2L2, PLEKHG1, RASGRF2, SPATA13, TIAM1, TIAM2, TNFAIP1, VAV2, VAV3*
B cell mediated immunity	1.6×10^{-5}	115.9	*HLA-DQB1, HLA-DRB1, HLA-DRB5*

GO categories in which false discovery rate is less than 0.01.

Table S17 continued.

Description	p-value	Enrichment	Genes
Antigen processing and presentation of exogenous peptide antigen via MHC class I TAP-dependent	2.0×10^{-5}	112.7	*HLA-A*, *HLA-B*, *HLA-C*
Regulation of cell activation	2.1×10^{-5}	14.1	*HLA-DPA1*, *HLA-DPB1*, *HLA-DQA1*, *HLA-DQB1*, *HLA-DRB1*, *HLA-DRB5*
Regulation of membrane potential	2.2×10^{-5}	2.7	*ABCB5*, *ADRA1A*, *ANK2*, *ANK3*, *CACNA1A*, *CASQ2*, *CNR2*, *DPP6*, *GRID2*, *GRIK2*, *GRIN2A*, *KCNC2*, *KCNIP1*, *KCNMA1*, *KCNMB2*, *KCNMB3*, *NEDD4*, *NEDD4L*, *NLGN1*, *NRXN1*, *PKP2*, *RELN*, *RYR2*, *SCN9A*, *WWP2*
Cell projection organization	2.3×10^{-5}	1.7	*ALS2*, *ANK3*, *ARHGAP26*, *ARHGAP42*, *ARHGEF4*, *ASAP1*, *CACNA1A*, *CDH13*, *CDH23*, *CELSR2*, *CHAT*, *CLIC5*, *CLMN*, *CNTN4*, *CNTNAP2*, *CSF1R*, *CTNNA2*, *DAB1*, *DCC*, *DFNB31*, *DNAH9*, *DNM3*, *DSCAM*, *DYNC2H1*, *EPHA4*, *EPHB1*, *FAS*, *FGD5*, *FLOT1*, *FOPNL*, *GAS7*, *GLI2*, *IFT122*, *IFT43*, *IQCB1*, *ITGA8*, *KALRN*, *KLF7*, *KLHL1*, *LPAR3*, *LRRC16A*, *MAP2*, *MTSS1*, *MUTED*, *MYLK*, *MYO16*, *MYO7A*, *NEDD4*, *NRCAM*, *PAK1*, *PARVA*, *PCM1*, *PKHD1*, *PLEK*, *PLXNA4*, *PRKCA*, *PRKG1*, *PTK2B*, *PTPRM*, *RAC2*, *RELN*, *RSPH9*, *SDC2*, *SEMA3A*, *SPAG16*, *SPATA13*, *TEKT4*, *TMEM17*, *TMEM237*, *TNN*, *VAV2*, *VAV3*, *WWTR1*
Antigen processing and presentation of exogenous peptide antigen via MHC class I	2.4×10^{-5}	106.8	*HLA-A*, *HLA-B*, *HLA-C*
Cellular response to chemical stimulus	2.6×10^{-5}	7.4	*HLA-A*, *HLA-B*, *HLA-C*, *HLA-DPA1*, *HLA-DPB1*, *HLA-DQA1*, *HLA-DQB1*, *HLA-DRB5*
Regulation of ion transmembrane transport	2.7×10^{-5}	3.0	*ACTN4*, *ANK2*, *ANK3*, *ANO6*, *CACNG8*, *CAMK2D*, *CASQ2*, *CNIH3*, *DAPK1*, *JPH3*, *KCNE1*, *MYO5A*, *NEDD4*, *NEDD4L*, *NLGN1*, *NRXN1*, *PLCG2*, *RELN*, *RYR2*, *TESC*, *TRPC6*, *WWP2*
Positive regulation of transporter activity	2.9×10^{-5}	14.0	*ANK2*, *ANK3*, *PLCG2*, *RELN*, *RYR2*, *TRPC6*
Regulation of potassium ion transmembrane transporter activity	3.6×10^{-5}	13.5	*ANK2*, *CASQ2*, *NEDD4*, *NEDD4L*, *WWP2*
Humoral immune response	4.1×10^{-5}	37.7	*DMBT1*, *HLA-DQB1*, *HLA-DRB1*, *HLA-DRB5*
Antigen processing and presentation of peptide antigen via MHC class I	4.4×10^{-5}	88.2	*HLA-A*, *HLA-B*, *HLA-C*
Detection of external stimulus	5.2×10^{-5}	35.5	*HLA-A*, *HLA-B*, *HLA-DRB1*, *HLA-DRB5*
Regulation of potassium ion transport	5.7×10^{-5}	4.3	*ANK2*, *ANK3*, *CASQ2*, *DPP6*, *KCNE1*, *KCNE3*, *NEDD4*, *NEDD4L*, *PTK2B*, *STK39*, *WWP2*
Regulation of interleukin-10 secretion	6.0×10^{-5}	309.1	*HLA-DRB1*, *HLA-DRB5*
Regulation of small GTPase mediated signal transduction	6.0×10^{-5}	1.9	*ABCA1*, *ADRA1A*, *ALS2*, *ARAP1*, *ARAP3*, *ARHGAP15*, *ARHGAP22*, *ARHGAP24*, *ARHGAP25*, *ARHGAP26*, *ARHGAP44*, *ARHGAP8*, *ARHGEF18*, *ARHGEF37*, *ARHGEF4*, *BCR*, *BNIP2*, *CDC42BPA*, *CDON*, *CHN2*, *DGKI*, *DLC1*, *ECT2L*, *FARP1*, *FLOT1*, *IQSEC1*, *KALRN*, *KANK1*, *MCF2L2*, *NOTCH2*, *NRG1*, *PLEKHG1*, *PSD3*, *RAC2*, *RASGEF1C*, *RASGRF2*, *RELN*, *RGL1*, *SPATA13*, *STARD13*, *TIAM1*, *TIAM2*, *TNFAIP1*, *VAV2*, *VAV3*

GO categories in which false discovery rate is less than 0.01.
