# Supplementary material for: A Model-Based Approach for Identifying Signatures of Ancient Balancing Selection in Genetic Data
Source: PLoS Genet. 2014 Aug 21;10(8):e1004561. doi: 10.1371/journal.pgen.1004561 (PMC4140648; doi:10.1371/journal.pgen.1004561)
Supplement: Table S18 — GO process analysis of ranked signals from CEU population using the test statistic. (PDF) [file pgen.1004561.s044.pdf]

Table S18: GO process analysis of ranked signals from CEU population using the T_2 test statistic.

Description	p-value	Enrichment	Genes
Biological adhesion	8.8×10^{-16}	2.3	*ADAM12, AJAP1, AMTN, ANGPT1, BMPR1B, CADM2, CDH10, CDH13, CDH4, CDHR2, CDON, CDSN, CLDN1, CLDN10, CLDN14, CLSTN2, CNTN1, CNTN4, CNTN5, CNTNAP2, CNTNAP4, CNTNAP5, COL13A1, COL4A3, COL5A1, CTNNA2, CTNNA3, CTNND2, CXADR, CXCL12, DCHS2, DDR1, DSC1, DSCAM, DSG1, DSG3, EMR1, EPHB1, F5, FAT2, FAT3, FAT4, FER, HABP2, HPSE, IGFBP7, IGSF5, ITGA1, ITGA2, ITGB5, ITGBL1, KITLG, LAMA1, LAMA2, LAMC1, LAMC2, LOXL2, LPP, LSAMP, MEGF10, MEGF11, MTSS1, MUC16, MYH9, NCAM2, NELL2, NFASC, NID2, NRCAM, NRP1, NRXN1, NTM, NTN1, ODZ3, OPCML, PCDH15, PCDH17, PCDHA1, PCDHA10, PCDHA11, PCDHA12, PCDHA13, PCDHA2, PCDHA3, PCDHA4, PCDHA5, PCDHA6, PCDHA7, PCDHA8, PCDHA9, PCDHAC1, PKD1L1, PKHD1, PKP1, PKP2, PLEK, PLEKHA7, PRKCE, PRPH2, PTPRD, PTPRF, PTPRM, PTPRT, PTPRU, RELN, ROBO2, RPSA, SCARB1, SDK1, SELE, SEMA5A, SIRPA, SPON1, SSPO, SYK, TECTA, TEK, THBS2, TNN, TNR, VNN1, VWF*
Cell adhesion	8.8×10^{-16}	2.3	*ADAM12, AJAP1, AMTN, ANGPT1, BMPR1B, CADM2, CDH10, CDH13, CDH4, CDHR2, CDON, CDSN, CLDN1, CLDN10, CLDN14, CLSTN2, CNTN1, CNTN4, CNTN5, CNTNAP2, CNTNAP4, CNTNAP5, COL13A1, COL4A3, COL5A1, CTNNA2, CTNNA3, CTNND2, CXADR, CXCL12, DCHS2, DDR1, DSC1, DSCAM, DSG1, DSG3, EMR1, EPHB1, F5, FAT2, FAT3, FAT4, FER, HABP2, HPSE, IGFBP7, IGSF5, ITGA1, ITGA2, ITGB5, ITGBL1, KITLG, LAMA1, LAMA2, LAMC1, LAMC2, LOXL2, LPP, LSAMP, MEGF10, MEGF11, MTSS1, MUC16, MYH9, NCAM2, NELL2, NFASC, NID2, NRCAM, NRP1, NRXN1, NTM, NTN1, ODZ3, OPCML, PCDH15, PCDH17, PCDHA1, PCDHA10, PCDHA11, PCDHA12, PCDHA13, PCDHA2, PCDHA3, PCDHA4, PCDHA5, PCDHA6, PCDHA7, PCDHA8, PCDHA9, PCDHAC1, PKD1L1, PKHD1, PKP1, PKP2, PLEK, PLEKHA7, PRKCE, PRPH2, PTPRD, PTPRF, PTPRM, PTPRT, PTPRU, RELN, ROBO2, RPSA, SCARB1, SDK1, SELE, SEMA5A, SIRPA, SPON1, SSPO, SYK, TECTA, TEK, THBS2, TNN, TNR, VNN1, VWF*
Cell-cell adhesion	8.4×10^{-13}	2.7	*BMPR1B, CDH10, CDH13, CDH4, CDHR2, CDSN, CLDN1, CLDN10, CLDN14, CLSTN2, CNTN4, COL13A1, CTNNA2, CTNNA3, CTNND2, CXADR, DCHS2, DSC1, DSG1, DSG3, FAT2, FAT3, FAT4, FER, IGSF5, MEGF10, MEGF11, MYH9, NCAM2, NRCAM, NRXN1, NTN1, ODZ3, PCDH15, PCDH17, PCDHA1, PCDHA10, PCDHA11, PCDHA12, PCDHA13, PCDHA2, PCDHA3, PCDHA4, PCDHA5, PCDHA6, PCDHA7, PCDHA8, PCDHA9, PCDHAC1, PKD1L1, PKHD1, PKP2, PLEK, PLEKHA7, PTPRD, PTPRF, PTPRM, PTPRT, PTPRU, ROBO2, SELE, SYK, TEK, TNR, VNN1*
Interferon-gamma-mediated signaling pathway	1.8×10^{-10}	42.9	*HLA-A, HLA-B, HLA-C, HLA-DPA1, HLA-DPB1, HLA-DQA1, HLA-DQB1, HLA-DRB1*
Cellular response to interferon-gamma	1.1×10^{-9}	34.7	*HLA-A, HLA-B, HLA-C, HLA-DPA1, HLA-DPB1, HLA-DQA1, HLA-DQB1, HLA-DRB1*
Homophilic cell adhesion	1.5×10^{-9}	4.1	*CDH10, CDH13, CDH4, CDHR2, CLSTN2, DSC1, DSG1, DSG3, FAT2, PCDH15, PCDH17, PCDHA1, PCDHA10, PCDHA11, PCDHA12, PCDHA13, PCDHA2, PCDHA3, PCDHA4, PCDHA5, PCDHA6, PCDHA7, PCDHA8, PCDHA9, PCDHAC1, PTPRM, ROBO2*
Response to interferon-gamma	5.6×10^{-9}	28.5	*HLA-A, HLA-B, HLA-C, HLA-DPA1, HLA-DPB1, HLA-DQA1, HLA-DQB1, HLA-DRB1*
Antigen processing and presentation of peptide antigen	8.3×10^{-9}	18.1	*ERAP1, HLA-A, HLA-B, HLA-C, HLA-DPA1, HLA-DPB1, HLA-DQA1, HLA-DQB1, HLA-DRB1*
Antigen processing and presentation	2.8×10^{-8}	15.9	*ERAP1, HLA-A, HLA-B, HLA-C, HLA-DPA1, HLA-DPB1, HLA-DQA1, HLA-DQB1, HLA-DRB1*
Axon guidance	5.9×10^{-8}	2.0	*ABLIM1, ABLIM2, ANK2, ANK3, BMPR1B, CACNA1C, CACNB2, CAP1, CDH4, CHL1, CNTN1, CNTN4, COL4A1, COL4A2, COL4A3, COL5A1, COL5A2, COL9A1, CXCL12, DCC, DOCK1, DPYSL2, EGFR, EPHA7, EPHB1, ETV1, EXT1, EZR, ITGA1, ITGA2, ITGA9, KCNQ3, KIF5C, LAMA1, LAMA2, LAMC1, MATN2, MYH11, MYH9, MYO10, NFASC, NRCAM, NRP1, NRXN1, NRXN3, NTN1, NTN4, PAK2, PLXNA4, PTPRM, RAC2, RELN, ROBO1, ROBO2, RPS6KA2, SCN3B, SEMA3A, SEMA3E, SEMA5A, SEMA6D, SH3GL2, SLIT3, SPTB, TNR, TRPC4, TRPC6, UNC5C, VAV2*
Antigen processing and presentation of exogenous peptide antigen	9.9×10^{-8}	17.2	*HLA-A, HLA-B, HLA-C, HLA-DPA1, HLA-DPB1, HLA-DQA1, HLA-DQB1, HLA-DRB1*
Antigen processing and presentation of exogenous antigen	1.1×10^{-7}	17.0	*HLA-A, HLA-B, HLA-C, HLA-DPA1, HLA-DPB1, HLA-DQA1, HLA-DQB1, HLA-DRB1*
Protein localization to membrane	3.1×10^{-7}	9.1	*ANK2, ANK3, CACNA1A, CPE, DLG2, GRIK2, MAGI2, NRXN1, RAMP3, RELN, SCP2*
Antigen processing and presentation of exogenous peptide antigen via MHC class I TAP-independent	5.2×10^{-7}	300.5	*HLA-A, HLA-B, HLA-C*

GO categories in which false discovery rate is less than 0.01.

Table S18 continued.

Description	p-value	Enrichment	Genes
Chemotaxis	1.3×10^{-6}	1.7	*ABLIM1*, *ABLIM2*, *ANGPT1*, *ANK2*, *ANK3*, *BMPR1B*, *CACNA1C*, *CACNB2*, *CAP1*, *CCL15*, *CCL23*, *CCL24*, *CDH4*, *CHL1*, *CMTM7*, *CMTM8*, *CNTN1*, *CNTN4*, *COL4A1*, *COL4A2*, *COL4A3*, *COL5A1*, *COL5A2*, *COL9A1*, *CXADR*, *CXCL11*, *CXCL12*, *DCC*, *DEFB1*, *DOCK1*, *DOCK2*, *DOCK4*, *EGFR*, *EMR2*, *EPHA7*, *EPHB1*, *ETV1*, *EZR*, *FER*, *FGF2*, *ITGA1*, *ITGA2*, *ITGA9*, *KCNQ3*, *KIF5C*, *LAMA1*, *LAMA2*, *LAMC1*, *MATN2*, *MYH11*, *MYH9*, *MYO10*, *NFASC*, *NRCAM*, *NRP1*, *NRXN1*, *NRXN3*, *NTN1*, *NTN4*, *PAK2*, *PDGFA*, *PIK3C2G*, *PLA2G6*, *PLAUR*, *PLD1*, *PLXNA4*, *PRKCA*, *PTPRM*, *RAC2*, *RELN*, *ROBO1*, *ROBO2*, *RPS6KA2*, *SCN3B*, *SEMA3A*, *SEMA3E*, *SEMA5A*, *SEMA6D*, *SH3GL2*, *SLIT3*, *SPTB*, *SYK*, *TNFRSF11A*, *TNR*, *TRPC4*, *TRPC6*, *UNC5C*, *VAV2*
Taxis	1.3×10^{-6}	1.7	*ABLIM1*, *ABLIM2*, *ANGPT1*, *ANK2*, *ANK3*, *BMPR1B*, *CACNA1C*, *CACNB2*, *CAP1*, *CCL15*, *CCL23*, *CCL24*, *CDH4*, *CHL1*, *CMTM7*, *CMTM8*, *CNTN1*, *CNTN4*, *COL4A1*, *COL4A2*, *COL4A3*, *COL5A1*, *COL5A2*, *COL9A1*, *CXADR*, *CXCL11*, *CXCL12*, *DCC*, *DEFB1*, *DOCK1*, *DOCK2*, *DOCK4*, *EGFR*, *EMR2*, *EPHA7*, *EPHB1*, *ETV1*, *EZR*, *FER*, *FGF2*, *ITGA1*, *ITGA2*, *ITGA9*, *KCNQ3*, *KIF5C*, *LAMA1*, *LAMA2*, *LAMC1*, *MATN2*, *MYH11*, *MYH9*, *MYO10*, *NFASC*, *NRCAM*, *NRP1*, *NRXN1*, *NRXN3*, *NTN1*, *NTN4*, *PAK2*, *PDGFA*, *PIK3C2G*, *PLA2G6*, *PLAUR*, *PLD1*, *PLXNA4*, *PRKCA*, *PTPRM*, *RAC2*, *RELN*, *ROBO1*, *ROBO2*, *RPS6KA2*, *SCN3B*, *SEMA3A*, *SEMA3E*, *SEMA5A*, *SEMA6D*, *SH3GL2*, *SLIT3*, *SPTB*, *SYK*, *TNFRSF11A*, *TNR*, *TRPC4*, *TRPC6*, *UNC5C*, *VAV2*
Locomotion	2.3×10^{-6}	1.5	*ABLIM1*, *ABLIM2*, *ANGPT1*, *ANGPT2*, *ANGPT4*, *ANK2*, *ANK3*, *ANKS1A*, *AVL9*, *B4GALT1*, *BMPR1B*, *CACNA1C*, *CACNB2*, *CAP1*, *CCDC40*, *CCL15*, *CCL23*, *CCL24*, *CD244*, *CDC42BPA*, *CDH13*, *CDH4*, *CHL1*, *CMTM7*, *CMTM8*, *CNTN1*, *CNTN4*, *COL4A1*, *COL4A2*, *COL4A3*, *COL5A1*, *COL5A2*, *COL9A1*, *CTNNA2*, *CXADR*, *CXCL11*, *CXCL12*, *DCC*, *DDR1*, *DEFB1*, *DISC1*, *DNAH5*, *DNAH8*, *DNER*, *DOCK1*, *DOCK2*, *DOCK4*, *DPYSL2*, *DYNC2H1*, *EGFR*, *EMR2*, *EPHA7*, *EPHB1*, *ERBB4*, *ETV1*, *EXT1*, *EZR*, *FAT2*, *FER*, *FGF2*, *GPC6*, *GPLD1*, *GRIN2A*, *INPP5D*, *INSL6*, *ITGA1*, *ITGA2*, *ITGA9*, *KCNQ3*, *KIAA0319*, *KIF5C*, *KIRREL3*, *KITLG*, *LAMA1*, *LAMA2*, *LAMC1*, *LDHC*, *LOXL2*, *LRRC16A*, *LRRK2*, *LYN*, *MAPT*, *MATN2*, *MYH11*, *MYH9*, *MYO10*, *MYO5A*, *NEDD4*, *NFASC*, *NKX2-3*, *NR-CAM*, *NRG1*, *NRP1*, *NRXN1*, *NRXN3*, *NTN1*, *NTN4*, *OVOL2*, *PAK2*, *PAK7*, *PARD3*, *PDGFA*, *PIK3C2G*, *PLA2G6*, *PLAUR*, *PLD1*, *PLXNA4*, *PPAP2A*, *PRKCA*, *PRKG1*, *PTPRM*, *RAC2*, *RELN*, *ROBO1*, *ROBO2*, *RPS6KA2*, *SCARB1*, *SCN3B*, *SCNN1G*, *SELE*, *SEMA3A*, *SEMA3E*, *SEMA5A*, *SEMA6D*, *SH3GL2*, *SIRPA*, *SLC22A16*, *SLC7A7*, *SLC9C1*, *SLIT3*, *SPATA13*, *SPTB*, *SYK*, *TBX20*, *TEK*, *TNFRSF11A*, *TNN*, *TNR*, *TNS1*, *TRPC4*, *TRPC6*, *UNC5C*, *VAV2*, *VAV3*, *WWC1*
Cytokine-mediated signaling pathway	3.7×10^{-6}	9.2	*HLA-A*, *HLA-B*, *HLA-C*, *HLA-DPA1*, *HLA-DPB1*, *HLA-DQA1*, *HLA-DQB1*, *HLA-DRB1*, *NUP88*
Detection of bacterium	4.4×10^{-6}	27.3	*HLA-A*, *HLA-B*, *HLA-DRB1*, *HLA-DRB5*, *PGLYRP4*
T cell costimulation	6.4×10^{-6}	20.0	*HLA-DPA1*, *HLA-DPB1*, *HLA-DQA1*, *HLA-DQB1*, *HLA-DRA*, *HLA-DRB1*
Lymphocyte costimulation	7.1×10^{-6}	19.6	*HLA-DPA1*, *HLA-DPB1*, *HLA-DQA1*, *HLA-DQB1*, *HLA-DRA*, *HLA-DRB1*
Regulation of immune response	9.8×10^{-6}	7.1	*DMBT1*, *ERAP1*, *HLA-A*, *HLA-B*, *HLA-C*, *HLA-DPA1*, *HLA-DPB1*, *HLA-DQA1*, *HLA-DQB1*, *HLA-DRB1*
Immune response-activating signal transduction	2.0×10^{-5}	8.7	*DMBT1*, *HLA-DPA1*, *HLA-DPB1*, *HLA-DQA1*, *HLA-DQB1*, *HLA-DRA*, *HLA-DRB1*, *MAP2K3*

GO categories in which false discovery rate is less than 0.01.
