# Supplementary material for: A Model-Based Approach for Identifying Signatures of Ancient Balancing Selection in Genetic Data
Source: PLoS Genet. 2014 Aug 21;10(8):e1004561. doi: 10.1371/journal.pgen.1004561 (PMC4140648; doi:10.1371/journal.pgen.1004561)
Supplement: Table S19 — GO process analysis of ranked signals from YRI population using the test statistic. (PDF) [file pgen.1004561.s045.pdf]

Table S19: GO process analysis of ranked signals from YRI population using the T_2 test statistic.

Description	p-value	Enrichment	Genes
Biological adhesion	2.9×10^{-13}	2.1	*ADAM12, AMTN, ANGPT1, BMPR1B, CADM2, CD84, CDH10, CDH13, CDH23, CDH4, CDH9, CDON, CDSN, CELSR2, CFDP1, CLCA2, CLDN10, CLDN14, CLDN23, CLSTN2, CNTN1, CNTN4, CNTN5, CNTN6, CNTNAP2, CNTNAP4, CNTNAP5, COL11A1, COL2A1, COL4A3, COL5A1, CTNNA2, CTNNA3, CTNND2, CYFIP2, DAB1, DCHS2, DDR2, DSC1, DSC3, DSCAM, DSG1, EMR1, EMR2, EPHA3, F5, FAT2, FAT3, GPNMB, HABP2, IGFBP7, IGSF5, ITGA1, ITGA11, ITGA2, ITGA8, ITGA9, ITGAE, ITGB3, ITGB5, ITGBL1, LAMA2, LAMA4, LAMC1, LAMC2, LOXL2, LPP, MAEA, MAGI1, MTSS1, MYH9, NCAM2, NELL2, NFASC, NID2, NLGN1, NRCAM, NRXN1, NTM, ODZ3, OPCML, PARVA, PCDH15, PCDHA1, PCDHA10, PCDHA11, PCDHA12, PCDHA13, PCDHA2, PCDHA3, PCDHA4, PCDHA5, PCDHA6, PCDHA7, PCDHA8, PCDHA9, PCDHAC1, PKD1L1, PKHD1, PLEKHA7, PPFIBP1, PRKCA, PRKCE, PTPRD, PTPRM, PTPRT, RELN, ROBO2, RPSA, SCARB1, SDK1, SEMA5A, SIRPA, SMAD6, SPON1, SYK, TEK, TNN, TNR, VCL, VNN1, VWF*
Cell adhesion	2.9×10^{-13}	2.1	*ADAM12, AMTN, ANGPT1, BMPR1B, CADM2, CD84, CDH10, CDH13, CDH23, CDH4, CDH9, CDON, CDSN, CELSR2, CFDP1, CLCA2, CLDN10, CLDN14, CLDN23, CLSTN2, CNTN1, CNTN4, CNTN5, CNTN6, CNTNAP2, CNTNAP4, CNTNAP5, COL11A1, COL2A1, COL4A3, COL5A1, CTNNA2, CTNNA3, CTNND2, CYFIP2, DAB1, DCHS2, DDR2, DSC1, DSC3, DSCAM, DSG1, EMR1, EMR2, EPHA3, F5, FAT2, FAT3, GPNMB, HABP2, IGFBP7, IGSF5, ITGA1, ITGA11, ITGA2, ITGA8, ITGA9, ITGAE, ITGB3, ITGB5, ITGBL1, LAMA2, LAMA4, LAMC1, LAMC2, LOXL2, LPP, MAEA, MAGI1, MTSS1, MYH9, NCAM2, NELL2, NFASC, NID2, NLGN1, NRCAM, NRXN1, NTM, ODZ3, OPCML, PARVA, PCDH15, PCDHA1, PCDHA10, PCDHA11, PCDHA12, PCDHA13, PCDHA2, PCDHA3, PCDHA4, PCDHA5, PCDHA6, PCDHA7, PCDHA8, PCDHA9, PCDHAC1, PKD1L1, PKHD1, PLEKHA7, PPFIBP1, PRKCA, PRKCE, PTPRD, PTPRM, PTPRT, RELN, ROBO2, RPSA, SCARB1, SDK1, SEMA5A, SIRPA, SMAD6, SPON1, SYK, TEK, TNN, TNR, VCL, VNN1, VWF*
Cell-cell adhesion	6.7×10^{-11}	2.6	*BMPR1B, CDH10, CDH13, CDH23, CDH4, CDH9, CDSN, CLDN14, CLDN23, CLSTN2, CNTN4, COL11A1, COL2A1, CTNNA2, CTNNA3, CTNND2, CYFIP2, DAB1, DCHS2, DSC1, DSC3, DSG1, FAT2, FAT3, IGSF5, ITGA8, MYH9, NCAM2, NLGN1, NRCAM, NRXN1, ODZ3, PARVA, PCDH15, PCDHA1, PCDHA10, PCDHA11, PCDHA12, PCDHA13, PCDHA2, PCDHA3, PCDHA4, PCDHA5, PCDHA6, PCDHA7, PCDHA8, PCDHA9, PCDHAC1, PKD1L1, PKHD1, PLEKHA7, PTPRD, PTPRM, PTPRT, ROBO2, SYK, TEK, TNR, VCL, VNN1*
Homophilic cell adhesion	1.4×10^{-8}	3.1	*CD84, CDH10, CDH13, CDH23, CDH4, CDH9, CELSR2, CLSTN2, DCHS2, DSC1, DSC3, DSG1, FAT2, FAT3, ODZ3, PCDH15, PCDHA1, PCDHA10, PCDHA11, PCDHA12, PCDHA13, PCDHA2, PCDHA3, PCDHA4, PCDHA5, PCDHA6, PCDHA7, PCDHA8, PCDHA9, PCDHAC1, PTPRM, PTPRT, ROBO2*
Interferon-gamma-mediated signaling pathway	3.4×10^{-8}	13.9	*HLA-A, HLA-B, HLA-C, HLA-DPA1, HLA-DPB1, HLA-DQA1, HLA-DQA2, HLA-DQB1, HLA-DRB1, HLA-DRB5*
Cellular response to interferon-gamma	2.9×10^{-7}	11.2	*HLA-A, HLA-B, HLA-C, HLA-DPA1, HLA-DPB1, HLA-DQA1, HLA-DQA2, HLA-DQB1, HLA-DRB1, HLA-DRB5*
Detection of bacterium	6.1×10^{-7}	40.0	*HLA-A, HLA-B, HLA-DRB1, HLA-DRB5, PGLYRP4*
Axon guidance	6.8×10^{-7}	2.0	*ABLIM1, ABLIM3, ANK2, ANK3, BMPR1B, CACNA1C, CAP1, CAP2, CDH4, CNTN1, CNTN2, CNTN4, CNTN6, COL2A1, COL4A1, COL4A2, COL4A3, COL4A4, COL5A1, DCC, DOCK1, DPYSL2, DPYSL3, EGFR, ETV1, EXT1, ITGA1, ITGA2, ITGA9, ITGB3, LAMA1, LAMA2, LAMC1, LHX4, MYH14, MYH9, MYO10, NFASC, NRCAM, NRP1, NRXN1, NRXN3, NTN1, NTN4, PLXNA4, PTPRM, PTPRO, RELN, ROBO2, RPS6KA2, SCN3B, SEMA3A, SEMA3E, SEMA5A, SEMA6D, SH3GL2, SLIT3, SPTB, TNR, TRPC4, TRPC6, UNC5C, UNC5D, VAV2, WNT3*
Regulation of small GTPase mediated signal transduction	1.5×10^{-6}	2.2	*ABCA1, ADRA1A, ALS2, ARHGAP15, ARHGAP22, ARHGAP24, ARHGAP25, ARHGAP26, ARHGAP28, ARHGAP44, ARHGAP8, ARHGEF18, BCR, BNIP2, CDC42BPA, CDON, CHN2, DEPDC7, DGKI, DLC1, FAM13A, FLOT1, IQSEC1, KALRN, KANK1, MCF2L2, MYO9B, NOTCH2, PLEKHG1, PLEKHG4B, PSD3, RAPGEF4, RASGEF1C, RELN, RGL1, SPATA13, SRGAP3, STARD13, TAGAP, TIAM1, TIAM2, VAV1, VAV2, VAV3*
Response to interferon-gamma	1.9×10^{-6}	9.2	*HLA-A, HLA-B, HLA-C, HLA-DPA1, HLA-DPB1, HLA-DQA1, HLA-DQA2, HLA-DQB1, HLA-DRB1, HLA-DRB5*
Antigen processing and presentation of peptide antigen	2.8×10^{-6}	6.2	*HLA-A, HLA-B, HLA-C, HLA-DPA1, HLA-DPB1, HLA-DQA1, HLA-DQA2, HLA-DQB1, HLA-DRB1, HLA-DRB5, MARCH1, TAP2*

GO categories in which false discovery rate is less than 0.01

Table S19 continued.

Description	p-value	Enrichment	Genes
Protein localization to membrane	6.0×10^{-6}	4.2	*ANK2*, *ANK3*, *CACNA1A*, *CAPN3*, *CPE*, *DLG2*, *FLOT1*, *GRIK2*, *MAGI2*, *MYO5A*, *NLGN1*, *NRXN1*, *RAMP3*, *RELN*, *SCN3B*, *SCP2*
Antigen processing and presentation of exogenous peptide antigen	1.0×10^{-5}	6.1	*HLA-A*, *HLA-B*, *HLA-C*, *HLA-DPA1*, *HLA-DPB1*, *HLA-DQA1*, *HLA-DQA2*, *HLA-DQB1*, *HLA-DRB1*, *HLA-DRB5*, *TAP2*
Antigen processing and presentation of exogenous antigen	1.1×10^{-5}	6.0	*HLA-A*, *HLA-B*, *HLA-C*, *HLA-DPA1*, *HLA-DPB1*, *HLA-DQA1*, *HLA-DQA2*, *HLA-DQB1*, *HLA-DRB1*, *HLA-DRB5*, *TAP2*
Regulation of cell morphogenesis	1.1×10^{-5}	1.9	*ARAP1*, *ARHGAP15*, *ARHGEF18*, *ATP10A*, *CACNA1A*, *CCL24*, *CDH4*, *CFDP1*, *CIT*, *CNTN2*, *DAB1*, *DCC*, *DLC1*, *DSCAM*, *EPHA3*, *FGD4*, *FGD5*, *FXN*, *GAS2*, *GAS7*, *KANK1*, *LIMD1*, *LRP8*, *LRRC4C*, *MBP*, *MYH14*, *MYH9*, *MYO10*, *NEDD4*, *NRCAM*, *NRP1*, *NTN1*, *NTRK2*, *NTRK3*, *PALM2*, *PALMD*, *PDLIM5*, *PDZD8*, *PLXNA4*, *PPP2CA*, *PTPRD*, *PTPRO*, *RELN*, *ROBO2*, *RUFY3*, *SEMA3A*, *SEMA3E*, *SHROOM3*, *TBCCD1*, *TIAM1*, *TNR*, *TTC3*, *WDPCP*, *WNT3*, *WWTR1*, *XYLT1*
Antigen processing and presentation	1.3×10^{-5}	5.5	*HLA-A*, *HLA-B*, *HLA-C*, *HLA-DPA1*, *HLA-DPB1*, *HLA-DQA1*, *HLA-DQA2*, *HLA-DQB1*, *HLA-DRB1*, *HLA-DRB5*, *MARCH1*, *TAP2*
Regulation of neuron projection development	1.3×10^{-5}	2.1	*CACNA1A*, *CCDC88A*, *CDH4*, *CIT*, *CNTN2*, *DAB1*, *DCC*, *DISC1*, *DPYSL3*, *DSCAM*, *EPHA3*, *FXN*, *HAP1*, *KALRN*, *KANK1*, *KIAA0319*, *LRP8*, *LRRC4C*, *MBP*, *NEDD4*, *NLGN1*, *NRCAM*, *NRP1*, *NTM*, *NTN1*, *NTRK2*, *NTRK3*, *ODZ3*, *PDLIM5*, *PLXNA4*, *PRKD1*, *PTPRD*, *PTPRO*, *RAPGEF4*, *RELN*, *ROBO2*, *RUFY3*, *SEMA3A*, *SERPINE2*, *TIAM1*, *TNR*, *WNT3*, *XYLT1*

GO categories in which false discovery rate is less than 0.01.
