# Supplementary material for: A Model-Based Approach for Identifying Signatures of Ancient Balancing Selection in Genetic Data
Source: PLoS Genet. 2014 Aug 21;10(8):e1004561. doi: 10.1371/journal.pgen.1004561 (PMC4140648; doi:10.1371/journal.pgen.1004561)
Supplement: Table S20 — GO function analysis of ranked signals from CEU population using the test statistic. (PDF) [file pgen.1004561.s046.pdf]

Table S20: GO function analysis of ranked signals from CEU population using the T_1 test statistic.

Description	p-value	Enrichment	Genes
MHC class II receptor activity	8.1×10^{-11}	49.2	*HLA-DPA1*, *HLA-DQA1*, *HLA-DQA2*, *HLA-DQB1*, *HLA-DQB2*, *HLA-DRA*, *HLA-DRB1*
Phospholipid binding	6.2×10^{-9}	1.9	*ABCA1*, *AFAP1*, *AKAP13*, *ALS2*, *ANXA6*, *APBB1IP*, *ARHGAP22*, *ARHGAP23*, *ARHGAP24*, *ARHGAP25*, *ARHGAP42*, *ARHGEF18*, *ARHGEF3*, *ASAP1*, *BCR*, *CADPS*, *CADPS2*, *CCDC88A*, *CDC42BPA*, *DAPP1*, *DGKH*, *DNM3*, *DOCK10*, *DOK6*, *DYSF*, *ELMO1*, *FARP1*, *FGD4*, *FGD5*, *GRB10*, *HIP1*, *IQSEC1*, *ITPR1*, *ITPR2*, *KALRN*, *KIF16B*, *MCF2L2*, *MYO10*, *MYO1B*, *MYOF*, *NGEF*, *NOXO1*, *OSBPL3*, *PARD3*, *PCLO*, *PHLDB2*, *PIK3C2B*, *PLA2G4A*, *PLA2G4C*, *PLCB1*, *PLCG2*, *PLCH1*, *PLCL1*, *PLD1*, *PLEK*, *PLEKHA6*, *PLEKHA7*, *PLEKHG1*, *PLEKHH2*, *PON1*, *PREX1*, *PREX2*, *PSD3*, *PXK*, *RASGRF1*, *RASGRF2*, *RTKN2*, *SBF2*, *SCARB1*, *SGIP1*, *SKAP2*, *SNTB1*, *SNTG2*, *SNX16*, *SNX19*, *SNX24*, *SNX29*, *SNX31*, *SNX5*, *SNX7*, *SPATA13*, *STAP1*, *SWAP70*, *TEC*, *TIAM1*, *TIAM2*, *TULP3*, *VAV1*, *VAV2*, *VAV3*, *VNN1*, *ZFYVE1*, *ZFYVE28*
Metal ion binding	2.5×10^{-8}	1.4	*ABO*, *ACSM5*, *ACTN1*, *ACTN2*, *ADARB2*, *ADCY3*, *ADCY5*, *ADCY8*, *ADD2*, *AGMAT*, *AKAP13*, *ALPL*, *AMDHD1*, *AMPD3*, *ANGPT2*, *ANXA6*, *APIP*, *APOA1BP*, *APP*, *ARSB*, *ARSG*, *ARSJ*, *ATP10B*, *ATP10D*, *ATP12A*, *ATP1A4*, *ATP2C2*, *ATP8A1*, *ATP8A2*, *ATP8B1*, *ATP9A*, *ATP9B*, *B4GALT1*, *BMPR1B*, *BNIP2*, *C1GALT1*, *CABP5*, *CABS1*, *CACNA1A*, *CACNA2D1*, *CACNA2D3*, *CADPS*, *CADPS2*, *CAPN14*, *CAPN8*, *CAPN9*, *CARS2*, *CASQ2*, *CCBE1*, *CDC42BPA*, *CDH10*, *CDH13*, *CDH23*, *CDH4*, *CDH9*, *CDHR2*, *CDKAL1*, *CLSTN2*, *CNOT6*, *COL27A1*, *COL5A2*, *COL9A1*, *COLEC12*, *CUBN*, *CYBRD1*, *CYP2C8*, *CYP2E1*, *CYP4F11*, *CYP4F12*, *CYP4F2*, *CYP4F3*, *CYP4F8*, *CYP4Z1*, *DBH*, *DCHS2*, *DDAH1*, *DDR1*, *DGKB*, *DGKH*, *DMRT1*, *DNER*, *DPYD*, *DSC1*, *DSG1*, *DTNA*, *EBF2*, *EFCAB11*, *EFCAB4B*, *EFEMP1*, *EGLN1*, *ELAC2*, *ELTD1*, *EMR1*, *EMR2*, *ENDOU*, *ENOSF1*, *ENPP1*, *EYS*, *F13A1*, *F5*, *FAHD1*, *FARS2*, *FAT2*, *FAT3*, *FAT4*, *FBN2*, *FBP2*, *FCER2*, *FGD4*, *FGD5*, *FHIT*, *FRAS1*, *FSCB*, *FSTL4*, *FSTL5*, *FTO*, *FXN*, *GALNT10*, *GALNT2*, *GALNTL6*, *GLRX3*, *GPR39*, *GRM7*, *GUCA1A*, *HAAO*, *HDAC7*, *HDHD2*, *HGD*, *HMGCLL1*, *HPCAL4*, *IDO2*, *IMPA2*, *INPP1*, *ITGA1*, *ITGA2*, *ITGA9*, *KALRN*, *KCNIP1*, *KCNIP4*, *KSR2*, *LEPREL1*, *LHPP*, *LOXL2*, *LRP1B*, *LTBP1*, *LTF*, *LYAR*, *MAN1A1*, *MAST4*, *MATN2*, *ME3*, *MGAT1*, *MGAT5B*, *MGMT*, *MLPH*, *MMP20*, *MOB2*, *MOB3B*, *MOXD1*, *MYO5A*, *NCALD*, *NCS1*, *NECAB2*, *NELL1*, *NELL2*, *NID2*, *NME7*, *NOTCH2*, *NOTCH4*, *NRP1*, *NRXN1*, *NRXN3*, *NUBPL*, *OAS1*, *OCM2*, *PADI2*, *PADI4*, *PADI6*, *PCDH15*, *PCDH17*, *PCDHA1*, *PCDHA10*, *PCDHA11*, *PCDHA12*, *PCDHA13*, *PCDHA2*, *PCDHA3*, *PCDHA4*, *PCDHA5*, *PCDHA6*, *PCDHA7*, *PCDHA8*, *PCDHA9*, *PCDHAC1*, *PCLO*, *PDE11A*, *PDE1A*, *PDE1C*, *PDE3A*, *PDE4D*, *PDE8B*, *PDSS1*, *PELO*, *PFKP*, *PGM2*, *PINK1*, *PKD1L2*, *PLA2G4A*, *PLA2G4E*, *PLCB1*, *PLCB4*, *PLCH1*, *PLCL1*, *PLCZ1*, *PLOD2*, *PLSCR1*, *PON1*, *PPA2*, *PRIM2*, *PRKCE*, *PRPSAP1*, *PRTFDC1*, *PRUNE2*, *PXDN*, *PXDNL*, *RELN*, *RIMS1*, *RIMS2*, *RNPEP*, *RPS6KA2*, *RYR2*, *RYR3*, *SCD5*, *SCN1A*, *SLC25A25*, *SLC8A1*, *SLIT3*, *SMOC1*, *SMOC2*, *SNCA*, *SPARCL1*, *STAC*, *STK32A*, *STK32B*, *SVEP1*, *SWAP70*, *SYT6*, *SYT9*, *TAF1B*, *TEC*, *TESC*, *THBS2*, *TIMP2*, *TKT*, *TNFRSF11A*, *TP63*, *TPD52*, *TPO*, *TYW1*, *UNC13C*, *VAV2*, *VAV3*, *ZFYVE28*
MHC class I receptor activity	2.8×10^{-8}	760.6	*HLA-A*, *HLA-B*, *HLA-C*
Transmembrane transporter activity	3.6×10^{-8}	1.6	*ABCA1*, *ABCA4*, *ABCB5*, *ABCC11*, *ABCC4*, *AKR1C4*, *ANO1*, *ANO2*, *AQP8*, *AQP9*, *ASIC2*, *ATP10B*, *ATP10D*, *ATP12A*, *ATP1A4*, *ATP1B3*, *ATP2C2*, *ATP5C1*, *ATP6V0E2*, *ATP6V1C2*, *ATP6V1G2*, *ATP8A1*, *ATP8A2*, *ATP8B1*, *ATP9A*, *ATP9B*, *BEST3*, *CACNA1A*, *CACNA2D1*, *CACNA2D3*, *CACNB2*, *CHRNA9*, *CHRNB3*, *CLCA2*, *CLCNKB*, *CLIC5*, *CNGA1*, *CNGA3*, *COX7B2*, *FGF2*, *GABRB2*, *GABRG3*, *GABRR1*, *GLRA3*, *GRIA1*, *GRIA4*, *GRID2*, *GRIK2*, *GRIK3*, *GRIK4*, *GRIN2A*, *GRIN2B*, *GRIN3A*, *GRM7*, *ITPR1*, *ITPR2*, *KCNA6*, *KCNAB1*, *KCNB2*, *KCNH7*, *KCNIP1*, *KCNIP4*, *KCNJ12*, *KCNJ16*, *KCNK10*, *KCNK2*, *KCNMB1*, *KCNMB2*, *KCNMB3*, *KCNQ3*, *KCNQ4*, *KCNQ5*, *LRRC52*, *MCOLN2*, *MRS2*, *NALCN*, *NCS1*, *NIPAL1*, *NIPAL3*, *NMUR2*, *OCA2*, *PEX5L*, *PIEZO1*, *PIEZO2*, *PKD1L2*, *RYR1*, *RYR2*, *RYR3*, *SCN10A*, *SCN1A*, *SCN3A*, *SCN3B*, *SCN9A*, *SCNN1G*, *SLC10A2*, *SLC12A6*, *SLC12A8*, *SLC14A1*, *SLC14A2*, *SLC15A2*, *SLC15A5*, *SLC16A14*, *SLC17A5*, *SLC19A3*, *SLC1A2*, *SLC1A6*, *SLC22A16*, *SLC22A4*, *SLC22A5*, *SLC24A4*, *SLC28A1*, *SLC2A1*, *SLC2A14*, *SLC2A9*, *SLC30A9*, *SLC31A2*, *SLC35D2*, *SLC38A4*, *SLC39A11*, *SLC39A14*, *SLC39A8*, *SLC41A3*, *SLC4A7*, *SLC5A12*, *SLC6A5*, *SLC7A7*, *SLC8A1*, *SLC8A3*, *SLC9A4*, *SLC9A8*, *SLC9A9*, *SLC9C1*, *SLCO1B1*, *SLCO1B3*, *SV2B*, *SV2C*, *TAP1*, *TAP2*, *TOMM7*, *TRPA1*, *TRPC4*, *TRPC6*, *TRPM3*, *ZDHHC13*

GO categories in which false discovery rate is less than 0.01.

Table S20 continued.

Description	p-value	Enrichment	Genes
Calcium ion binding	2.0×10^{-7}	1.8	*ACTN1, ACTN2, CABP5, CABS1, CADPS, CAPN14, CAPN8, CAPN9, CASQ2, CCBE1, CDH10, CDH13, CDH23, CDH4, CDH9, CDHR2, CLSTN2, CUBN, DCHS2, DGKB, DNER, DSC1, DSG1, DTNA, EFCAB11, EFCAB4B, EFEMP1, ELTD1, EMR1, EMR2, EYS, FAT2, FAT4, FSCB, FSTL4, FSTL5, GRM7, GUCA1A, HPCAL4, KCNIP1, KCNIP4, LRP1B, LTBP1, MAN1A1, MATN2, MGMT, MMP20, MYO5A, NCALD, NELL1, NID2, NOTCH4, OCM2, PADI2, PADI4, PADI6, PCDH15, PCDH17, PCDHA1, PCDHA10, PCDHA11, PCDHA12, PCDHA13, PCDHA2, PCDHA3, PCDHA4, PCDHA5, PCDHA6, PCDHA7, PCDHA8, PCDHA9, PCDHAC1, PCLO, PKD1L2, PLCB1, PLCB4, PLCH1, PLSCR1, PON1, RYR2, RYR3, SLC25A25, SLIT3, SMOC1, SMOC2, SNCA, SPARCL1, SVEP1, SWAP70, TESC, THBS2, TPD52, TPO*
Ion gated channel activity	2.5×10^{-7}	2.0	*ANO1, ANO2, ASIC2, CACNA1A, CACNA2D1, CACNA2D3, CACNB2, CHRNA9, CHRNB3, CLCA2, CLCNKB, CLIC5, CNGA3, FGF2, GABRB2, GABRG3, GABRR1, GLRA3, GRIA1, GRIA4, GRID2, GRIK2, GRIK3, GRIK4, GRIN2A, GRIN2B, GRIN3A, GRM7, ITPR1, ITPR2, KCNA6, KCNAB1, KCNB2, KCNH7, KCNIP1, KCNIP4, KCNJ12, KCNJ16, KCNK10, KCNK2, KCNMB1, KCNMB2, KCNMB3, KCNQ3, KCNQ4, KCNQ5, NALCN, NCS1, NMUR2, PEX5L, PIEZO1, RYR1, RYR2, RYR3, SCN10A, SCN1A, SCN3A, SCN3B, SCN9A, SCNN1G, TRPC4, TRPC6*
Gated channel activity	2.5×10^{-7}	2.0	*ANO1, ANO2, ASIC2, CACNA1A, CACNA2D1, CACNA2D3, CACNB2, CHRNA9, CHRNB3, CLCA2, CLCNKB, CLIC5, CNGA3, FGF2, GABRB2, GABRG3, GABRR1, GLRA3, GRIA1, GRIA4, GRID2, GRIK2, GRIK3, GRIK4, GRIN2A, GRIN2B, GRIN3A, GRM7, ITPR1, ITPR2, KCNA6, KCNAB1, KCNB2, KCNH7, KCNIP1, KCNIP4, KCNJ12, KCNJ16, KCNK10, KCNK2, KCNMB1, KCNMB2, KCNMB3, KCNQ3, KCNQ4, KCNQ5, NALCN, NCS1, NMUR2, PEX5L, PIEZO1, RYR1, RYR2, RYR3, SCN10A, SCN1A, SCN3A, SCN3B, SCN9A, SCNN1G, TRPC4, TRPC6*
Substrate-specific transmembrane transporter activity	2.7×10^{-7}	1.6	*ABCA1, AKR1C4, ANO1, ANO2, AQP8, AQP9, ASIC2, ATP10B, ATP10D, ATP12A, ATP1A4, ATP1B3, ATP2C2, ATP5C1, ATP6V0E2, ATP6V1C2, ATP8A1, ATP8A2, ATP8B1, ATP9A, ATP9B, BEST3, CACNA1A, CACNA2D1, CACNA2D3, CACNB2, CHRNA9, CHRNB3, CLCA2, CLCNKB, CLIC5, CNGA1, CNGA3, COX7B2, FGF2, GABRB2, GABRG3, GABRR1, GLRA3, GRIA1, GRIA4, GRID2, GRIK2, GRIK3, GRIK4, GRIN2A, GRIN2B, GRIN3A, GRM7, ITPR1, ITPR2, KCNA6, KCNAB1, KCNB2, KCNH7, KCNIP1, KCNIP4, KCNJ12, KCNJ16, KCNK10, KCNK2, KCNMB1, KCNMB2, KCNMB3, KCNQ3, KCNQ4, KCNQ5, LRRC52, MCOLN2, MRS2, NALCN, NCS1, NIPAL1, NIPAL3, NMUR2, OCA2, PEX5L, PIEZO1, PIEZO2, PKD1L2, RYR1, RYR2, RYR3, SCN10A, SCN1A, SCN3A, SCN3B, SCN9A, SCNN1G, SLC10A2, SLC12A6, SLC14A1, SLC14A2, SLC15A2, SLC17A5, SLC19A3, SLC1A2, SLC1A6, SLC22A16, SLC22A4, SLC22A5, SLC28A1, SLC2A1, SLC2A14, SLC2A9, SLC30A9, SLC31A2, SLC35D2, SLC38A4, SLC39A11, SLC39A14, SLC39A8, SLC41A3, SLC4A7, SLC6A5, SLC7A7, SLC8A1, SLC8A3, SLC9A4, SLC9A8, SLC9A9, SLC9C1, SLCO1B1, SLCO1B3, TAP1, TAP2, TOMM7, TRPA1, TRPC4, TRPC6, TRPM3, ZDHHC13*
Rho guanyl-nucleotide exchange factor activity	$6.4 \times 10^{-}$	3.3	*AKAP13, ALS2, ARHGEF18, ARHGEF3, ARHGEF37, BCR, DOCK10, FARP1, FGD4, FGD5, KALRN, MCF2L2, NGEF, PLEKHG1, PREX1, PREX2, RASGRF1, RASGRF2, SPATA13, TIAM1, TIAM2, VAV1, VAV2, VAV3*
Ion transmembrane transporter activity	8.3×10^{-7}	1.6	*ABCA1, AKR1C4, ANO1, ANO2, AQP9, ASIC2, ATP10B, ATP10D, ATP12A, ATP1A4, ATP1B3, ATP2C2, ATP5C1, ATP6V0E2, ATP6V1C2, ATP8A1, ATP8A2, ATP8B1, ATP9A, ATP9B, BEST3, CACNA1A, CACNA2D1, CACNA2D3, CACNB2, CHRNA9, CHRNB3, CLCA2, CLCNKB, CLIC5, CNGA1, CNGA3, COX7B2, FGF2, GABRB2, GABRG3, GABRR1, GLRA3, GRIA1, GRIA4, GRID2, GRIK2, GRIK3, GRIK4, GRIN2A, GRIN2B, GRIN3A, GRM7, ITPR1, ITPR2, KCNA6, KCNAB1, KCNB2, KCNH7, KCNIP1, KCNIP4, KCNJ12, KCNJ16, KCNK10, KCNK2, KCNMB1, KCNMB2, KCNMB3, KCNQ3, KCNQ4, KCNQ5, LRRC52, MCOLN2, MRS2, NALCN, NCS1, NIPAL1, NIPAL3, NMUR2, OCA2, PEX5L, PIEZO1, PIEZO2, PKD1L2, RYR1, RYR2, RYR3, SCN10A, SCN1A, SCN3A, SCN3B, SCN9A, SCNN1G, SLC10A2, SLC12A6, SLC15A2, SLC17A5, SLC1A2, SLC1A6, SLC22A16, SLC22A4, SLC22A5, SLC28A1, SLC2A9, SLC30A9, SLC31A2, SLC38A4, SLC39A11, SLC39A14, SLC39A8, SLC41A3, SLC4A7, SLC6A5, SLC7A7, SLC8A1, SLC8A3, SLC9A4, SLC9A8, SLC9A9, SLC9C1, SLCO1B1, SLCO1B3, TRPA1, TRPC4, TRPC6, TRPM3, ZDHHC13*
Glutamate receptor activity	2.6×10^{-6}	4.9	*GRIA1, GRIA4, GRID2, GRIK2, GRIK3, GRIK4, GRIN2A, GRIN2B, GRIN3A, GRM4, GRM5, GRM7, GRM8*

GO categories in which false discovery rate is less than 0.01.

Table S20 continued.

Description	p-value	Enrichment	Genes
Substrate-specific channel activity	3.0×10^{-6}	1.8	*ANO1, ANO2, AQP8, AQP9, ASIC2, BEST3, CACNA1A, CACNA2D1, CACNA2D3, CACNB2, CHRNA9, CHRNB3, CLCA2, CLCNKB, CLIC5, CNGA1, CNGA3, FGF2, GABRB2, GABRG3, GABRR1, GLRA3, GRIA1, GRIA4, GRID2, GRIK2, GRIK3, GRIK4, GRIN2A, GRIN2B, GRIN3A, GRM7, ITPR1, ITPR2, KCNA6, KCNAB1, KCNB2, KCNH7, KCNIP1, KCNIP4, KCNJ12, KCNJ16, KCNK10, KCNK2, KCNMB1, KCNMB2, KCNMB3, KCNQ3, KCNQ4, KCNQ5, LRRC52, MCOLN2, NALCN, NCS1, NMUR2, PEX5L, PIEZO1, PIEZO2, PKD1L2, RYR1, RYR2, RYR3, SCN10A, SCN1A, SCN3A, SCN3B, SCN9A, SCNN1G, TRPA1, TRPC4, TRPC6, TRPM3*
Ion channel activity	5.0×10^{-6}	1.8	*ANO1, ANO2, ASIC2, BEST3, CACNA1A, CACNA2D1, CACNA2D3, CACNB2, CHRNA9, CHRNB3, CLCA2, CLCNKB, CLIC5, CNGA1, CNGA3, FGF2, GABRB2, GABRG3, GABRR1, GLRA3, GRIA1, GRIA4, GRID2, GRIK2, GRIK3, GRIK4, GRIN2A, GRIN2B, GRIN3A, GRM7, ITPR1, ITPR2, KCNA6, KCNAB1, KCNB2, KCNH7, KCNIP1, KCNIP4, KCNJ12, KCNJ16, KCNK10, KCNK2, KCNMB1, KCNMB2, KCNMB3, KCNQ3, KCNQ4, KCNQ5, LRRC52, MCOLN2, NALCN, NCS1, NMUR2, PEX5L, PIEZO1, PIEZO2, PKD1L2, RYR1, RYR2, RYR3, SCN10A, SCN1A, SCN3A, SCN3B, SCN9A, SCNN1G, TRPA1, TRPC4, TRPC6, TRPM3*
Receptor activity	5.1×10^{-6}	1.5	*ABCA1, ADRA1A, ALK, ASGR2, BAI3, BMPR1B, CLEC1A, CLEC1B, CNR2, COLEC12, CUBN, DAB2, DCC, DMBT1, DNER, EFEMP1, ELTD1, EMR1, ENDOU, EPHB1, ERBB4, F2RL2, FAS, FSHR, GABRR1, GPR111, GPR139, GPR158, GPR39, GPR78, GRIA1, GRIK2, GRIK3, GRIN2A, GRIN3A, GRM5, GRM7, GRM8, HLA-A, HLA-B, HLA-C, HLA-DOA, HLA-DPA1, HLA-DQA1, HLA-DQA2, HLA-DQB1, HLA-DQB2, HLA-DRA, HLA-DRB1, HLA-F, HLA-G, IL1RL2, ITGB5, ITGBL1, KLRB1, LGR5, LHCGR, LOXL2, LRP1B, LTBP1, MCC, NOTCH4, NPSR1, NRP1, NRXN1, NRXN3, OPCML, OPRK1, OR10AG1, OR10C1, OR12D2, OR13C5, OR14A16, OR1L8, OR1N2, OR1S1, OR2AK2, OR2L13, OR2T4, OR2W3, OR4C3, OR4C45, OR51B2, OR51B6, OR51E1, OR51F1, OR51I2, OR51M1, OR51Q1, OR52E2, OR52E6, OR52J3, OR52N2, OR52N4, OR52R1, OR5AC2, OR5F1, OR5I1, OR5P2, OR5W2, OR8G1, OR8G5, OR8H3, OR8I2, OR8U8, OR9G1, OR9G9, PDGFRL, PGLYRP4, PKHD1, PLA2R1, PLXNA4, PPYR1, PROKR2, PTCHD3, PTGER3, PTPRB, PTPRD, PTPRM, RAMP3, ROBO2, ROR1, RORA, RXRG, SCARB1, SEMA3A, SEMA3E, SEMA5A, SEMA6D, SORCS1, SORCS2, SORCS3, TAS2R19, TAS2R20, TEK, TMPRSS15, TNFRSF11A, TSHR*
Passive transmembrane transporter activity	1.3×10^{-5}	1.8	*ANO1, ANO2, AQP8, AQP9, ASIC2, BEST3, CACNA1A, CACNA2D1, CACNA2D3, CACNB2, CHRNA9, CHRNB3, CLCA2, CLCNKB, CLIC5, CNGA1, CNGA3, FGF2, GABRB2, GABRG3, GABRR1, GLRA3, GRIA1, GRIA4, GRID2, GRIK2, GRIK3, GRIK4, GRIN2A, GRIN2B, GRIN3A, GRM7, ITPR1, ITPR2, KCNA6, KCNAB1, KCNB2, KCNH7, KCNIP1, KCNIP4, KCNJ12, KCNJ16, KCNK10, KCNK2, KCNMB1, KCNMB2, KCNMB3, KCNQ3, KCNQ4, KCNQ5, LRRC52, MCOLN2, NALCN, NCS1, NMUR2, PEX5L, PIEZO1, PIEZO2, PKD1L2, RYR1, RYR2, RYR3, SCN10A, SCN1A, SCN3A, SCN3B, SCN9A, SCNN1G, TRPA1, TRPC4, TRPC6, TRPM3*
Channel activity	1.3×10^{-5}	1.8	*ANO1, ANO2, AQP8, AQP9, ASIC2, BEST3, CACNA1A, CACNA2D1, CACNA2D3, CACNB2, CHRNA9, CHRNB3, CLCA2, CLCNKB, CLIC5, CNGA1, CNGA3, FGF2, GABRB2, GABRG3, GABRR1, GLRA3, GRIA1, GRIA4, GRID2, GRIK2, GRIK3, GRIK4, GRIN2A, GRIN2B, GRIN3A, GRM7, ITPR1, ITPR2, KCNA6, KCNAB1, KCNB2, KCNH7, KCNIP1, KCNIP4, KCNJ12, KCNJ16, KCNK10, KCNK2, KCNMB1, KCNMB2, KCNMB3, KCNQ3, KCNQ4, KCNQ5, LRRC52, MCOLN2, NALCN, NCS1, NMUR2, PEX5L, PIEZO1, PIEZO2, PKD1L2, RYR1, RYR2, RYR3, SCN10A, SCN1A, SCN3A, SCN3B, SCN9A, SCNN1G, TRPA1, TRPC4, TRPC6, TRPM3*
Lipid binding	1.4×10^{-5}	1.6	*ABCA1, ADH4, ADH5, AFAP1, AKAP13, AKR1C2, ALS2, ANXA6, APBB1IP, ARHGAP22, ARHGAP23, ARHGAP24, ARHGAP25, ARHGAP42, ARHGEF18, ARHGEF3, ASAP1, BCR, CADPS, CADPS2, CCDC88A, CDC42BPA, DAPP1, DGKH, DNM3, DOCK10, DOK6, DYSF, ELMO1, ESRRB, ESRRG, FABP2, FARP1, FER, FGD4, FGD5, GRB10, HIP1, HSDL2, INSR, IQSEC1, ITPR1, ITPR2, KALRN, KIF16B, KL, LAMA1, LAMC1, LYN, MCF2L2, MYO10, MYO1B, MYOF, NGEF, OPN4, OSBPL3, PARD3, PCLO, PHLDB2, PIK3C2B, PLA2G4A, PLA2G4C, PLCB1, PLCG2, PLCH1, PLCL1, PLD1, PLEK, PLEKHA6, PLEKHA7, PLEKHG1, PLEKHH2, PON1, PREX1, PREX2, PSD3, PXK, RASGRF1, RASGRF2, RTKN2, SBF2, SCARB1, SCP2, SGIP1, SH3GL2, SKAP2, SNTB1, SNTG2, SNX16, SNX19, SNX24, SNX29, SNX31, SNX5, SNX7, SOAT1, SPATA13, STAP1, SWAP70, TEC, TIAM1, TIAM2, TULP3, UGT1A7, UGT1A8, UGT1A9, UNC13A, VAV2, VAV3, VNN1, ZFYVE1, ZFYVE28*

GO categories in which false discovery rate is less than 0.01.

Table S20 continued.

Description	p-value	Enrichment	Genes
Transmembrane signaling receptor activity	1.5×10^{-5}	1.5	*ABCA1, ADRA1A, AKR1C3, ALK, BAI3, BMPR1B, CLEC1A, CLEC1B, CNR2, DCC, DNER, EFEMP1, ELTD1, EMR1, EPHB1, ERBB4, F2RL2, FAS, FLT3, FSHR, FZD6, GABRR1, GPR111, GPR139, GPR158, GPR39, GPR78, GRIA1, GRID2, GRIK2, GRIK3, GRIK4, GRIN2A, GRIN3A, GRM5, GRM7, GRM8, HLA-A, HLA-B, HLA-C, HLA-DOA, HLA-DPA1, HLA-DQA1, HLA-DQA2, HLA-DQB1, HLA-DQB2, HLA-DRA, HLA-DRB1, HLA-F, HLA-G, IL1RL2, KLRB1, LGR5, LHCGR, LTBP1, NPSR1, NRP1, OPCML, OPRK1, OR10AG1, OR10C1, OR12D2, OR13C5, OR14A16, OR1L8, OR1N2, OR1S1, OR2AK2, OR2L13, OR2T4, OR2W3, OR4C3, OR4C45, OR51B2, OR51B6, OR51E1, OR51F1, OR51I2, OR51M1, OR51Q1, OR52E2, OR52E6, OR52J3, OR52N2, OR52N4, OR52R1, OR5AC2, OR5F1, OR5I1, OR5P2, OR5W2, OR8G1, OR8G5, OR8H3, OR8I2, OR8U8, OR9G1, OR9G9, PDGFRL, PLXNA4, PPYR1, PROKR2, PTCHD3, PTGER3, PTPRB, PTPRD, PTPRM, ROBO2, ROR1, ROR2, SEMA5A, SORCS1, SORCS2, SORCS3, TAS2R19, TAS2R20, TEK, TNFRSF11A, TSHR*
Metal ion transmembrane transporter activity	1.7×10^{-5}	1.8	*ASIC2, ATP12A, ATP1A4, ATP1B3, ATP2C2, CACNA1A, CACNA2D1, CACNA2D3, CACNB2, CHRNA9, FGF2, GRIN2A, GRIN2B, GRIN3A, GRM7, ITPR1, ITPR2, KCNA6, KCNAB1, KCNB2, KCNH7, KCNIP1, KCNIP4, KCNJ12, KCNJ16, KCNK10, KCNK2, KCNMB1, KCNMB2, KCNMB3, KCNQ3, KCNQ4, KCNQ5, MRS2, NALCN, NCS1, NIPAL1, NIPAL3, RYR1, RYR2, RYR3, SCN10A, SCN1A, SCN3A, SCN3B, SCN9A, SCNN1G, SLC10A2, SLC1A2, SLC1A6, SLC28A1, SLC31A2, SLC39A11, SLC39A14, SLC39A8, SLC4A7, SLC6A5, SLC8A1, SLC8A3, SLC9A4, SLC9A8, SLC9A9, TRPA1, TRPC4, TRPC6, TRPM3, ZDHHC13*
Ras guanyl-nucleotide exchange factor activity	1.9×10^{-5}	2.6	*AKAP13, ALS2, ARHGEF18, ARHGEF3, ARHGEF37, BCR, DOCK10, FARP1, FGD4, FGD5, KALRN, MCF2L2, NGEF, PLEKHG1, PREX1, PREX2, RAPGEF4, RASGRF1, RASGRF2, RGL1, RIN2, SBF2, SPATA13, TIAM1, TIAM2, VAV1, VAV2, VAV3*
Signaling receptor activity	2.1×10^{-5}	7.8	*DMBT1, HLA-A, HLA-B, HLA-C, HLA-DPA1, HLA-DQA1, HLA-DQB1, OR4C3*
Transporter activity	2.2×10^{-5}	1.4	*ABCA1, ABCA4, ABCB5, ABCC11, ABCC4, ABCG8, AKR1C4, ANO1, ANO2, AP1S3, AQP8, AQP9, ASIC2, ATP10B, ATP10D, ATP12A, ATP1A4, ATP1B3, ATP2C2, ATP5C1, ATP6V0E2, ATP6V1C2, ATP6V1G2, ATP8A1, ATP8A2, ATP8B1, ATP9A, ATP9B, BEST3, CACNA1A, CACNA2D1, CACNA2D3, CACNB2, CHRNA9, CHRNB3, CLCA2, CLCNKB, CLIC5, CNGA1, CNGA3, COX7B2, CPLX1, CUBN, FABP2, FGF2, FLVCR1, GABRB2, GABRG3, GABRR1, GLRA3, GRIA1, GRIA4, GRID2, GRIK2, GRIK3, GRIK4, GRIN2A, GRIN2B, GRIN3A, GRM7, ITPR1, ITPR2, KCNA6, KCNAB1, KCNB2, KCNH7, KCNIP1, KCNIP4, KCNJ12, KCNJ16, KCNK10, KCNK2, KCNMB1, KCNMB2, KCNMB3, KCNQ3, KCNQ4, KCNQ5, KPNA7, LRRC52, MCOLN2, MRS2, NALCN, NCS1, NIPAL1, NIPAL3, NMUR2, NUP88, OCA2, PCLO, PEX5L, PIEZO1, PIEZO2, PKD1L2, PLSCR1, RAMP3, RYR1, RYR2, RYR3, SCARB1, SCN10A, SCN1A, SCN3A, SCN3B, SCN9A, SCNN1G, SCP2, SLC10A2, SLC12A6, SLC12A8, SLC14A1, SLC14A2, SLC15A2, SLC15A5, SLC16A14, SLC17A5, SLC19A3, SLC1A2, SLC1A6, SLC22A16, SLC22A4, SLC22A5, SLC24A4, SLC28A1, SLC2A1, SLC2A14, SLC2A9, SLC30A9, SLC31A2, SLC35D2, SLC37A1, SLC37A2, SLC38A4, SLC39A11, SLC39A14, SLC39A8, SLC41A3, SLC4A7, SLC5A12, SLC6A5, SLC7A7, SLC8A1, SLC8A3, SLC9A4, SLC9A8, SLC9A9, SLC9C1, SLCO1B1, SLCO1B3, SLCO1C1, SLCO5A1, SLCO6A1, SV2B, SV2C, SYNPR, SYT6, SYT9, TAP1, TAP2, TOMM7, TRPA1, TRPC4, TRPC6, TRPM3, ZDHHC13*
Signal transducer activity	2.3×10^{-5}	5.8	*DMBT1, HLA-A, HLA-B, HLA-C, HLA-DPA1, HLA-DQA1, HLA-DQB1, HLA-DRB1, OR4C3, RGS6*
Molecular transducer activity	2.3×10^{-5}	5.8	*DMBT1, HLA-A, HLA-B, HLA-C, HLA-DPA1, HLA-DQA1, HLA-DQB1, HLA-DRB1, OR4C3, RGS6*

GO categories in which false discovery rate is less than 0.01.

Table S20 continued.

Description	p-value	Enrichment	Genes
Substrate-specific transporter activity	2.6×10^{-5}	1.5	*ABCA1*, *ABCA4*, *ABCG8*, *AKR1C4*, *ANO1*, *ANO2*, *AP1S3*, *AQP8*, *AQP9*, *ASIC2*, *ATP10B*, *ATP10D*, *ATP12A*, *ATP1A4*, *ATP1B3*, *ATP2C2*, *ATP5C1*, *ATP6V0E2*, *ATP6V1C2*, *ATP8A1*, *ATP8A2*, *ATP8B1*, *ATP9A*, *ATP9B*, *BEST3*, *CACNA1A*, *CACNA2D1*, *CACNA2D3*, *CACNB2*, *CHRNA9*, *CHRNB3*, *CLCA2*, *CLCNKB*, *CLIC5*, *CNGA1*, *CNGA3*, *COX7B2*, *FABP2*, *FGF2*, *GABRB2*, *GABRG3*, *GABRR1*, *GLRA3*, *GRIA1*, *GRIA4*, *GRID2*, *GRIK2*, *GRIK3*, *GRIK4*, *GRIN2A*, *GRIN2B*, *GRIN3A*, *GRM7*, *ITPR1*, *ITPR2*, *KCNA6*, *KCNAB1*, *KCNB2*, *KCNH7*, *KCNIP1*, *KCNIP4*, *KCNJ12*, *KCNJ16*, *KCNK10*, *KCNK2*, *KCNMB1*, *KCNMB2*, *KCNMB3*, *KCNQ3*, *KCNQ4*, *KCNQ5*, *KPNA7*, *LRRC52*, *MCOLN2*, *MRS2*, *NALCN*, *NCS1*, *NIPAL1*, *NIPAL3*, *NMUR2*, *OCA2*, *PEX5L*, *PIEZO1*, *PIEZO2*, *PKD1L2*, *PLSCR1*, *RAMP3*, *RYR1*, *RYR2*, *RYR3*, *SCN10A*, *SCN1A*, *SCN3A*, *SCN3B*, *SCN9A*, *SCNN1G*, *SCP2*, *SLC10A2*, *SLC12A6*, *SLC14A1*, *SLC14A2*, *SLC15A2*, *SLC17A5*, *SLC19A3*, *SLC1A2*, *SLC1A6*, *SLC22A16*, *SLC22A4*, *SLC22A5*, *SLC28A1*, *SLC2A1*, *SLC2A14*, *SLC2A9*, *SLC30A9*, *SLC31A2*, *SLC35D2*, *SLC38A4*, *SLC39A11*, *SLC39A14*, *SLC39A8*, *SLC41A3*, *SLC4A7*, *SLC6A5*, *SLC7A7*, *SLC8A1*, *SLC8A3*, *SLC9A4*, *SLC9A8*, *SLC9A9*, *SLC9C1*, *SLCO1B1*, *SLCO1B3*, *TAP1*, *TAP2*, *TOMM7*, *TRPA1*, *TRPC4*, *TRPC6*, *TRPM3*, *ZDHHC13*
Phospholipid-translocating ATPase activity	4.2×10^{-5}	6.2	*ABCA4*, *ATP10B*, *ATP10D*, *ATP8A1*, *ATP8A2*, *ATP8B1*, *ATP9A*, *ATP9B*

GO categories in which false discovery rate is less than 0.01.
