# Supplementary material for: A Model-Based Approach for Identifying Signatures of Ancient Balancing Selection in Genetic Data
Source: PLoS Genet. 2014 Aug 21;10(8):e1004561. doi: 10.1371/journal.pgen.1004561 (PMC4140648; doi:10.1371/journal.pgen.1004561)
Supplement: Table S21 — GO function analysis of ranked signals from YRI population using the test statistic. (PDF) [file pgen.1004561.s047.pdf]

Table S21: GO function analysis of ranked signals from YRI population using the T_1 test statistic.

Description	p-value	Enrichment	Genes
MHC class II receptor activity	1.3×10^{-9}	130.9	*HLA-DPA1*, *HLA-DQA1*, *HLA-DQB1*, *HLA-DRA*, *HLA-DRB1*
MHC class I receptor activity	1.2×10^{-7}	507.1	*HLA-A*, *HLA-B*, *HLA-C*
Phospholipid binding	2.4×10^{-7}	2.0	*ABCA1*, *AFAP1*, *APBB1IP*, *ARAP1*, *ARAP3*, *ARHGAP22*, *ARHGAP24*, *ARHGAP25*, *ARHGAP26*, *ARHGAP42*, *ARHGEF18*, *ARHGEF4*, *ASAP1*, *CADPS*, *CDC42BPA*, *CNKSR1*, *DAPP1*, *DGKH*, *DOCK10*, *DOK5*, *DOK6*, *DOK7*, *DYSF*, *ELMO1*, *FARP1*, *FGD5*, *FRMPD2*, *GRB10*, *HS1BP3*, *IQSEC1*, *KALRN*, *LANCL2*, *MCF2L2*, *MYO10*, *MYO1B*, *MYOF*, *PCLO*, *PHLDB2*, *PIK3C2G*, *PLA2G4C*, *PLCB1*, *PLCG2*, *PLCH1*, *PLEK*, *PLEKHG1*, *PLEKHH2*, *PON1*, *PREX2*, *PSD3*, *PXK*, *SCARB1*, *SGIP1*, *SKAP2*, *SNTB1*, *SNTG1*, *SNTG2*, *SNX16*, *SNX19*, *SNX29*, *SNX31*, *SNX4*, *SNX7*, *SPATA13*, *SWAP70*, *TEC*, *TIAM1*, *TIAM2*, *TULP3*, *VAV2*, *VAV3*, *VNN1*, *VPS36*
Ion gated channel activity	9.7×10^{-7}	2.2	*ANO2*, *ANO6*, *ASIC2*, *CACNA1A*, *CACNA1C*, *CACNA2D3*, *CACNG8*, *CATSPER4*, *CHRNB3*, *CLCA2*, *CLCNKB*, *CLIC5*, *CNGA3*, *FGF2*, *GABRB3*, *GABRG3*, *GABRR1*, *GRIA1*, *GRID1*, *GRID2*, *GRIK2*, *GRIK4*, *GRIN2A*, *GRM7*, *JPH3*, *KCNAB1*, *KCNC2*, *KCND3*, *KCNE1*, *KCNE3*, *KCNIP1*, *KCNIP4*, *KCNJ12*, *KCNJ15*, *KCNJ6*, *KCNK2*, *KCNMA1*, *KCNMB1*, *KCNMB2*, *KCNMB3*, *KCNQ4*, *KCNQ5*, *PEX5L*, *RYR2*, *RYR3*, *SCN11A*, *SCN3A*, *SCN9A*, *SCNN1G*, *TPCN2*, *TRPC6*
Gated channel activity	9.7×10^{-7}	2.2	*ANO2*, *ANO6*, *ASIC2*, *CACNA1A*, *CACNA1C*, *CACNA2D3*, *CACNG8*, *CATSPER4*, *CHRNB3*, *CLCA2*, *CLCNKB*, *CLIC5*, *CNGA3*, *FGF2*, *GABRB3*, *GABRG3*, *GABRR1*, *GRIA1*, *GRID1*, *GRID2*, *GRIK2*, *GRIK4*, *GRIN2A*, *GRM7*, *JPH3*, *KCNAB1*, *KCNC2*, *KCND3*, *KCNE1*, *KCNE3*, *KCNIP1*, *KCNIP4*, *KCNJ12*, *KCNJ15*, *KCNJ6*, *KCNK2*, *KCNMA1*, *KCNMB1*, *KCNMB2*, *KCNMB3*, *KCNQ4*, *KCNQ5*, *PEX5L*, *RYR2*, *RYR3*, *SCN11A*, *SCN3A*, *SCN9A*, *SCNN1G*, *TPCN2*, *TRPC6*
Metal ion binding	2.5×10^{-6}	1.3	*ABO*, *ACACB*, *ACSM2B*, *ACSM3*, *ACTN4*, *ACY3*, *ADARB2*, *ADCY2*, *ADCY3*, *ADCY5*, *ADD2*, *AGMAT*, *ANGPT2*, *APIP*, *APOA1BP*, *ARSB*, *ARSJ*, *ATP10B*, *ATP10D*, *ATP2C2*, *ATP8A1*, *ATP8A2*, *BMPR1B*, *BNIP2*, *C1GALT1*, *CACNA1A*, *CACNA1C*, *CACNA2D1*, *CACNA2D3*, *CADPS*, *CALN1*, *CAPN14*, *CAPN8*, *CAPN9*, *CARS2*, *CASQ2*, *CCBE1*, *CCDC155*, *CDC42BPA*, *CDH10*, *CDH13*, *CDH18*, *CDH23*, *CDH4*, *CDH7*, *CDH9*, *CDHR2*, *CDKAL1*, *CELSR1*, *CELSR2*, *CHN2*, *CLSTN2*, *COL11A1*, *COL27A1*, *COL2A1*, *COL5A1*, *COL9A1*, *CPQ*, *CRTAC1*, *CTDSPL*, *CUBN*, *CYBRD1*, *CYP11B1*, *CYP19A1*, *CYP27A1*, *CYP2E1*, *CYP4F11*, *CYP4F12*, *CYP4F2*, *CYP4F3*, *CYP4Z1*, *DCHS2*, *DDAH1*, *DGKH*, *DGKI*, *DMRT1*, *DNER*, *DSC1*, *DSC3*, *DSG1*, *DSG3*, *DTNA*, *EBF2*, *EFCAB11*, *EFEMP1*, *EFHB*, *EMR1*, *EMR2*, *ENPP1*, *EPHX2*, *EXO1*, *EYA2*, *EYS*, *F13A1*, *F5*, *FAHD1*, *FARS2*, *FAT2*, *FAT3*, *FBN2*, *FBP2*, *FCER2*, *FGD5*, *FHIT*, *FRAS1*, *FREM1*, *FSCB*, *FSTL4*, *FSTL5*, *FTO*, *FXN*, *GALNT10*, *GALNT2*, *GALNTL6*, *GCLC*, *GPHN*, *GPR39*, *GRM7*, *GUCA1A*, *GUCA1B*, *HAAO*, *HBD*, *HGD*, *HMGCLL1*, *HPCAL4*, *IDO2*, *IMPA2*, *INPP1*, *IQGAP1*, *ISCU*, *ITGA1*, *ITGA10*, *ITGA11*, *ITGA8*, *ITGA9*, *KALRN*, *KCND3*, *KCNIP1*, *KCNIP4*, *KCNMA1*, *KSR2*, *LCN2*, *LEPREL1*, *LHPP*, *LOXL2*, *LRP1B*, *LTBP1*, *LYAR*, *MAN1A1*, *MARC1*, *MARC2*, *MAST4*, *MCTP2*, *ME3*, *MGAT1*, *MGAT4C*, *MGAT5B*, *MIPEP*, *MOB2*, *MOB3B*, *MOCOS*, *MOXD1*, *MRE11A*, *MYL1*, *MYLK*, *MYO5A*, *NCALD*, *NECAB2*, *NELL1*, *NELL2*, *NID2*, *NME7*, *NOS1*, *NOTCH2*, *NOTCH4*, *NQO2*, *NRXN1*, *NRXN3*, *NUBPL*, *NUDT7*, *OAS2*, *OCM2*, *OMA1*, *PADI2*, *PADI4*, *PAM*, *PCDH15*, *PCLO*, *PDE11A*, *PDE1A*, *PDE1C*, *PDE2A*, *PDE3A*, *PDE4B*, *PDE4D*, *PDSS1*, *PELO*, *PHF2*, *PINK1*, *PKD1L2*, *PKDREJ*, *PLA2G4E*, *PLCB1*, *PLCB4*, *PLCH1*, *PLEKHF2*, *PLSCR1*, *POLD1*, *PON1*, *PPA1*, *PPEF2*, *PPP3CA*, *PRIM2*, *PRKCE*, *PRKCH*, *PRTFDC1*, *PRUNE2*, *PTGS1*, *PXDN*, *PXDNL*, *RELN*, *REPS1*, *RIMS1*, *RNPEP*, *RPS6KA2*, *RYR2*, *RYR3*, *SCD5*, *SCGN*, *SCIN*, *SCN9A*, *SCUBE1*, *SDF4*, *SIAH3*, *SLIT3*, *SMOC2*, *SNCA*, *SPARCL1*, *SPOCK1*, *STAC*, *STK32A*, *STK32B*, *SULF1*, *SULF2*, *SWAP70*, *SYT5*, *SYT6*, *SYT9*, *TEC*, *TESC*, *TF*, *THBS2*, *TIMP2*, *TLL1*, *TNFRSF11A*, *TPO*, *TYW1*, *UNC13C*, *USP32*, *UTRN*, *VAV2*, *VAV3*, *ZFYVE26*
Glutamate receptor activity	3.0×10^{-6}	6.3	*GRIA1*, *GRID1*, *GRID2*, *GRIK2*, *GRIK4*, *GRIN2A*, *GRM1*, *GRM3*, *GRM5*, *GRM7*, *GRM8*

GO categories in which false discovery rate is less than 0.01.

Table S21 continued.

Description	p-value	Enrichment	Genes
Ion channel activity	4.2×10^{-6}	1.8	*ANO2, ANO6, ASIC2, CACNA1A, CACNA1C, CACNA2D1, CACNA2D3, CACNG8, CATSPER3, CATSPER4, CHRNA2, CHRNB3, CLCA2, CLCNKB, CLIC5, CNGA1, CNGA3, FGF2, GABRB3, GABRG3, GABRR1, GLRA3, GRIA1, GRID1, GRID2, GRIK2, GRIK4, GRIN2A, GRM7, JPH3, KCNAB1, KCNB2, KCNC2, KCND3, KCNE1, KCNE3, KCNIP1, KCNIP4, KCNJ12, KCNJ15, KCNJ6, KCNK18, KCNK2, KCNMA1, KCNMB1, KCNMB2, KCNMB3, KCNQ4, KCNQ5, LRRC52, MCOLN2, NALCN, PDE2A, PEX5L, PIEZO2, PKD1L2, PKDREJ, RYR2, RYR3, SCN11A, SCN3A, SCN5A, SCN9A, SCNN1G, SLC24A2, TPCN2, TRPC6, TRPM3, TRPM4*
Substrate-specific channel activity	4.8×10^{-6}	1.8	*ANO2, ANO6, AQP8, ASIC2, CACNA1A, CACNA1C, CACNA2D1, CACNA2D3, CACNG8, CATSPER3, CATSPER4, CHRNA2, CHRNB3, CLCA2, CLCNKB, CLIC5, CNGA1, CNGA3, FGF2, GABRB3, GABRG3, GABRR1, GLRA3, GRIA1, GRID1, GRID2, GRIK2, GRIK4, GRIN2A, GRM7, JPH3, KCNAB1, KCNB2, KCNC2, KCND3, KCNE1, KCNE3, KCNIP1, KCNIP4, KCNJ12, KCNJ15, KCNJ6, KCNK18, KCNK2, KCNMA1, KCNMB1, KCNMB2, KCNMB3, KCNQ4, KCNQ5, LRRC52, MCOLN2, NALCN, PDE2A, PEX5L, PIEZO2, PKD1L2, PKDREJ, RYR2, RYR3, SCN11A, SCN3A, SCN5A, SCN9A, SCNN1G, SLC24A2, TPCN2, TRPC6, TRPM3, TRPM4*
Actin binding	7.9×10^{-6}	1.9	*ABLIM1, ABLIM3, ACTN4, ACTR2, ADD2, AFAP1, ARPC5, CAP1, CLMN, COBLL1, CORO2B, DAAM2, EPB41L2, FER, FHOD3, FKBP15, FMN1, FMN2, FMNL2, GAS7, IQGAP2, KCNMA1, KLHL1, KLHL5, LIMCH1, MAEA, MICAL2, MTSS1, MYLK, MYO10, MYO16, MYO18B, MYO1B, MYO3B, MYO5A, MYO5B, MYO7A, MYOZ2, MYPN, MYRIP, NCALD, NRAP, PALLD, PARVA, PDLIM5, PHACTR1, PRKCE, PXK, RCSD1, SCIN, SHROOM3, SNTB1, SNTG1, SNTG2, SORBS1, SPTB, SVIL, SYNE1, TNS1, UTRN, WASF3, XIRP2*
Signaling receptor activity	1.6×10^{-5}	6.2	*DMBT1, HLA-A, HLA-B, HLA-C, HLA-DPA1, HLA-DQA1, HLA-DQB1, HLA-DRB1, OR4C3, OR4C45*
Phospholipid-translocating ATPase activity	1.7×10^{-5}	20.1	*ABCA4, ATP10B, ATP10D, ATP8A1, ATP8A2*
Passive transmembrane transporter activity	2.0×10^{-5}	1.7	*ANO2, ANO6, AQP8, ASIC2, CACNA1A, CACNA1C, CACNA2D1, CACNA2D3, CACNG8, CATSPER3, CATSPER4, CHRNA2, CHRNB3, CLCA2, CLCNKB, CLIC5, CNGA1, CNGA3, FGF2, GABRB3, GABRG3, GABRR1, GLRA3, GRIA1, GRID1, GRID2, GRIK2, GRIK4, GRIN2A, GRM7, JPH3, KCNAB1, KCNB2, KCNC2, KCND3, KCNE1, KCNE3, KCNIP1, KCNIP4, KCNJ12, KCNJ15, KCNJ6, KCNK18, KCNK2, KCNMA1, KCNMB1, KCNMB2, KCNMB3, KCNQ4, KCNQ5, LRRC52, MCOLN2, NALCN, PDE2A, PEX5L, PIEZO2, PKD1L2, PKDREJ, RYR2, RYR3, SCN11A, SCN3A, SCN5A, SCN9A, SCNN1G, SLC24A2, TPCN2, TRPC6, TRPM3, TRPM4*
Channel activity	2.0×10^{-5}	1.7	*ANO2, ANO6, AQP8, ASIC2, CACNA1A, CACNA1C, CACNA2D1, CACNA2D3, CACNG8, CATSPER3, CATSPER4, CHRNA2, CHRNB3, CLCA2, CLCNKB, CLIC5, CNGA1, CNGA3, FGF2, GABRB3, GABRG3, GABRR1, GLRA3, GRIA1, GRID1, GRID2, GRIK2, GRIK4, GRIN2A, GRM7, JPH3, KCNAB1, KCNB2, KCNC2, KCND3, KCNE1, KCNE3, KCNIP1, KCNIP4, KCNJ12, KCNJ15, KCNJ6, KCNK18, KCNK2, KCNMA1, KCNMB1, KCNMB2, KCNMB3, KCNQ4, KCNQ5, LRRC52, MCOLN2, NALCN, PDE2A, PEX5L, PIEZO2, PKD1L2, PKDREJ, RYR2, RYR3, SCN11A, SCN3A, SCN5A, SCN9A, SCNN1G, SLC24A2, TPCN2, TRPC6, TRPM3, TRPM4*
Transmembrane transporter activity	2.7×10^{-5}	1.5	*ABCA1, ABCA4, ABCB5, ABCC12, ABCC4, ABCD4, ABCG1, ANKH, ANO2, ANO6, AQP8, ASIC2, ATP10B, ATP10D, ATP2C2, ATP6V0A2, ATP6V0A4, ATP6V0E2, ATP8A1, ATP8A2, CACNA1A, CACNA1C, CACNA2D1, CACNA2D3, CACNG8, CATSPER3, CATSPER4, CHRNA2, CHRNB3, CLCA2, CLCNKB, CLIC5, CNGA1, CNGA3, FGF2, GABRB3, GABRG3, GABRR1, GLRA3, GRIA1, GRID1, GRID2, GRIK2, GRIK4, GRIN2A, GRM7, JPH3, KCNAB1, KCNB2, KCNC2, KCND3, KCNE1, KCNE3, KCNIP1, KCNIP4, KCNJ12, KCNJ15, KCNJ6, KCNK18, KCNK2, KCNMA1, KCNMB1, KCNMB2, KCNMB3, KCNQ4, KCNQ5, LRRC52, MCOLN2, NALCN, NIPAL3, OCA2, PDE2A, PEX5L, PIEZO2, PKD1L2, PKDREJ, RYR2, RYR3, SCN11A, SCN3A, SCN5A, SCN9A, SCNN1G, SIDT1, SLC12A6, SLC12A8, SLC15A2, SLC15A5, SLC16A14, SLC17A5, SLC19A3, SLC1A2, SLC1A6, SLC1A7, SLC22A16, SLC24A2, SLC24A3, SLC24A4, SLC26A11, SLC26A9, SLC28A3, SLC2A1, SLC2A9, SLC30A9, SLC35A1, SLC36A1, SLC36A2, SLC39A11, SLC39A12, SLC39A8, SLC4A4, SLC5A12, SLC7A1, SLC7A13, SLC7A5, SLC8A3, SLC9A2, SLC9A4, SLC9A9, SLC9C1, SLCO1B1, SLCO1B3, SV2C, TAP1, TAP2, TPCN2, TRPC6, TRPM3, TRPM4, ZDHHC13*

GO categories in which false discovery rate is less than 0.01.

Table S21 continued.

Description	p-value	Enrichment	Genes
Lipid binding	3.0×10^{-5}	1.5	*ABCA1, ABCG1, ACBD5, ADH4, ADH5, AFAP1, AKR1C2, ALS2, APBB1IP, APOC2, ARAP1, ARAP3, ARHGAP15, ARHGAP22, ARHGAP24, ARHGAP25, ARHGAP26, ARHGAP42, ARHGEF18, ARHGEF4, ASAP1, BCR, BPIFB2, BPIFB6, CADPS, CDC42BPA, CMIP, CNKSR1, DAPP1, DGKH, DNM3, DOCK10, DOK5, DOK6, DOK7, DYSF, ELMO1, ESR1, ESRRG, FARP1, FER, FGD5, FRMPD2, GRB10, HS1BP3, IQSEC1, KALRN, KL, LAMC1, LANCL2, LPAR3, MAP3K1, MCF2L2, MYO10, MYO1B, MYOF, NR3C1, NR3C2, O3FAR1, PCLO, PGR, PHLDB2, PIK3C2G, PLA2G4C, PLCB1, PLCG2, PLCH1, PLEK, PLEKHA6, PLEKHF2, PLEKHG1, PLEKHH2, PON1, PREX2, PSD3, PTGS1, PXK, RAS-GRF2, RBP4, SCARB1, SCIN, SCP2, SELL, SGIP1, SH3GL2, SKAP2, SNTB1, SNTG1, SNTG2, SNX16, SNX19, SNX29, SNX31, SNX4, SNX7, SNX9, SOAT1, SPATA13, SWAP70, TEC, TIAM1, TIAM2, TULP3, UGT1A7, UGT1A8, UGT1A9, VAV2, VAV3, VNN1, VPS36, ZFYVE26*
Cytoskeletal protein binding	3.8×10^{-5}	1.6	*ABLIM1, ABLIM3, ACTN4, ACTR2, ADD2, AFAP1, AGBL1, AGBL4, AGTPBP1, ANK1, ANK2, ANK3, ARHGAP26, ARHGAP42, ARPC5, ASAP1, BICD1, CACNA1C, CAP1, CCDC88C, CLMN, COBLL1, CORO2B, DAAM2, DLC1, DYNC1I1, EPB41L2, EPB41L4A, FARP1, FBXO25, FER, FHOD3, FKBP15, FMN1, FMN2, FMNL2, FRMD3, GAS7, IQGAP2, JAKMIP2, JAKMIP3, KCNE1, KCNMA1, KLHL1, KLHL5, LDB3, LIMCH1, LRPPRC, MAEA, MAGI1, MAP2, MICAL2, MTSS1, MTUS2, MYLK, MYO10, MYO16, MYO18B, MYO1B, MYO3B, MYO5A, MYO5B, MYO7A, MYOZ2, MYPN, MYRIP, NCALD, NRAP, NRCAM, PACSIN2, PALLD, PARVA, PDLIM5, PHACTR1, PRKCE, PTPRT, PXK, RCSD1, SCIN, SCN5A, SDC2, SGIP1, SHROOM3, SKA1, SNCA, SNTB1, SNTG1, SNTG2, SORBS1, SPTB, SVIL, SYBU, SYNE1, TBCD, TNS1, UTRN, WASF3, XIRP2*
Rho guanyl-nucleotide exchange factor activity	3.9×10^{-5}	3.1	*ALS2, ARHGEF18, ARHGEF37, ARHGEF4, BCR, DOCK10, DOCK2, ECT2L, FARP1, FGD5, KALRN, MCF2L2, PLEKHG1, PREX2, RASGRF2, SPATA13, TIAM1, TIAM2, VAV2, VAV3*
Transmembrane signaling receptor activity	4.4×10^{-5}	8.6	*HLA-A, HLA-B, HLA-C, HLA-DPA1, HLA-DQA1, HLA-DQB1, OR4C3*

GO categories in which false discovery rate is less than 0.01.
