# Supplementary material for: A Model-Based Approach for Identifying Signatures of Ancient Balancing Selection in Genetic Data
Source: PLoS Genet. 2014 Aug 21;10(8):e1004561. doi: 10.1371/journal.pgen.1004561 (PMC4140648; doi:10.1371/journal.pgen.1004561)
Supplement: Table S22 — GO function analysis of ranked signals from CEU population using the test statistic. (PDF) [file pgen.1004561.s048.pdf]

Table S22: GO function analysis of ranked signals from CEU population using the T_2 test statistic.

Description	p-value	Enrichment	Genes
Metal ion binding	5.6×10^{-11}	1.5	*ABO, ADARB2, ADCY3, ADCY5, ADD2, AGMAT, ALPL, AMDHD1, ANGPT2, APIP, APOA1BP, APP, ARSB, ARSG, ARSJ, ATP10B, ATP10D, ATP12A, ATP13A5, ATP2A3, ATP2B4, ATP2C2, ATP8A1, ATP8A2, ATP8B1, B4GALT1, BBOX1, BIRC5, BMPR1B, CABP5, CACNA1A, CACNA1C, CACNA2D1, CACNA2D3, CADPS, CADPS2, CALN1, CAPN14, CAPN8, CARS2, CASQ2, CCBE1, CDC42BPA, CDH10, CDH12, CDH13, CDH18, CDH4, CDH9, CDHR2, CDKAL1, CHN2, CLSTN2, COL27A1, COL5A1, COL5A2, COL9A1, COLEC12, CTDSPL, CUBN, CYBRD1, CYP2E1, CYP4F11, CYP4F12, CYP4F2, CYP4F3, CYP4F8, CYP4Z1, DCHS2, DDR1, DGKB, DGKH, DMRT1, DNER, DNTT, DPYD, DSC1, DSG1, DSG3, DTNA, EBF2, EFCAB11, EFCAB4B, EFEMP1, EGLN1, ELAC2, ELTD1, EMR1, EMR2, ENDOU, ENOSF1, ENPP1, EPHX2, EYA4, EYS, F13A1, F5, FAHD1, FARS2, FAT2, FAT3, FAT4, FBN2, FBP2, FCER2, FGD3, FGD4, FGD5, FHIT, FRAS1, FREM2, FRRS1, FSCB, FSTL5, FTO, FXN, GALNT10, GALNT2, GALNTL6, GNAL, GPHN, GPR39, GRM7, GUCA1A, HAAO, HDAC7, HDHD2, HGD, HMGCLL1, HPCAL4, IDO2, IMPA2, INPP1, ISCU, ITGA1, ITGA11, ITGA2, ITGA9, ITGAE, ITK, KALRN, KCNIP4, KCNMA1, KIAA0528, KSR2, LEPREL1, LHPP, LOXL2, LRP1B, LTBP1, LTF, LUC7L2, LYAR, MAN1A1, MATN2, ME3, MGAT1, MGAT5B, MGMT, MLPH, MMP20, MOB2, MYO5A, MYO9B, NAALAD2, NCALD, NCS1, NELL1, NELL2, NID2, NME7, NOTCH4, NRP1, NRXN1, NRXN3, NT5DC3, NUBPL, NUDT13, NUDT3, OAS1, OCM2, OMA1, OVCH1, PADI2, PADI4, PADI6, PAPD4, PAPL, PCDH15, PCDH17, PCDHA1, PCDHA10, PCDHA11, PCDHA12, PCDHA13, PCDHA2, PCDHA3, PCDHA4, PCDHA5, PCDHA6, PCDHA7, PCDHA8, PCDHA9, PCDHAC1, PCLO, PDE11A, PDE1A, PDE1C, PDE3A, PDE4D, PDE8B, PDSS1, PELO, PFKP, PGS1, PINK1, PKD1L2, PKDREJ, PLA2G4D, PLA2G4E, PLCB1, PLCB4, PLCH1, PLCL1, PLOD2, PLSCR1, POMT1, PON3, PPA2, PPP2CA, PPP3CA, PRIM2, PRKCE, PRTFDC1, PRUNE2, PTGS1, PXDN, PXDNL, RASAL1, RCN3, RELN, RIMS2, RNPEP, RPS6KA2, RYR1, RYR2, RYR3, SCD5, SCGN, SCN1A, SDF4, SIK2, SLC25A25, SLC8A1, SLIT3, SNCA, SNTN, SPARCL1, SPOCK3, STAC, STK32A, STK32B, STK32C, SVEP1, SWAP70, SYT1, SYT6, SYT9, TEC, TET1, THBS2, TIMP2, TNFRSF11A, TPD52, TPO, TYW1, UNC13C, VAV1, VAV2, VAV3, ZBED6, ZFYVE28*
Phospholipid binding	1.6×10^{-9}	2.0	*ABCA1, AFAP1, AFAP1L2, AGAP1, ALS2, APBB1IP, ARHGAP22, ARHGAP23, ARHGAP24, ARHGAP25, ARHGAP26, ARHGAP42, ARHGEF18, ARHGEF4, ASAP1, BCR, CADPS, CADPS2, CDC42BPA, DGKH, DOK6, DYSF, ELMO1, FGD3, FGD4, FGD5, GRB10, HIP1, IQSEC1, ITK, ITPR1, ITPR2, KALRN, KIF16B, MCF2L2, MYO10, MYO1B, MYOF, NOXO1, OSBPL11, OSBPL3, PARD3, PASK, PCLO, PHLDB2, PIK3C2B, PIK3C2G, PLA2G4C, PLCB1, PLCG2, PLCH1, PLCL1, PLD1, PLEK, PLEKHA6, PLEKHA7, PLEKHG1, PLEKHH2, PLEKHM2, PREX1, PREX2, PSD3, RASAL1, RTKN2, SBF2, SCARB1, SGIP1, SKAP2, SNTG1, SNTG2, SNX19, SNX24, SNX25, SNX29, SNX30, SNX31, SNX5, SNX7, SNX9, SPATA13, STAP1, SWAP70, SYT1, TEC, TIAM1, TIAM2, TULP3, VAV1, VAV2, VAV3, VNN1, ZFYVE1, ZFYVE28*
MHC class II receptor activity	3.8×10^{-9}	101.4	*HLA-DPA1, HLA-DQA1, HLA-DQB1, HLA-DRA, HLA-DRB1*

GO categories in which false discovery rate is less than 0.01.

Table S22 continued.

Description	p-value	Enrichment	Genes
Cation binding	6.8×10^{-7}	1.3	*ABLIM1, ABO, ACPP, ADAM12, ADAM28, ADAM29, ADAMTS12, ADAMTS16, ADAMTS18, ADAMTSL1, ADAMTSL3, ADARB2, ADCY3, ADCY5, ADD2, ADH1C, ADH4, AGAP1, AGBL1, AGBL4, AGMAT, AGTPBP1, ALPL, AMDHD1, ANGPT2, APIP, APOA1BP, APOBEC3H, APOBEC4, APTX, AQPEP, ARSB, ARSJ, ASAP1, ATP10D, ATP12A, ATP13A5, ATP2A3, ATP2B4, ATP2C2, ATP8A1, ATP8A2, ATP8B1, BARD1, BAZ1A, BAZ2B, BIRC5, BMPR1B, BNC2, BSPRY, CABP5, CACNA1A, CACNA1C, CACNA2D3, CADPS, CADPS2, CALN1, CAPN14, CAPN8, CARS2, CASQ2, CCBE1, CDC42BPA, CDH10, CDH12, CDH13, CDH18, CDH4, CDH9, CDHR2, CDKAL1, CHD5, CHIA, CHN2, CHRNB3, CLSTN2, COL27A1, COL5A1, COL5A2, COL9A1, COLEC12, CPA3, CPB2, CPE, CTDSPL, CUBN, CYBRD1, CYP2E1, CYP4F11, CYP4F12, CYP4F2, CYP4F3, CYP4F8, CYP4Z1, DCHS2, DCTD, DDR1, DGKB, DGKH, DMRT1, DNER, DNTT, DPF3, DPYD, DSC1, DSG1, DSG3, DTNA, DYTN, EBF2, EFCAB11, EFEMP1, EGLN1, ELAC2, ELTD1, EMR1, ENDOU, ENOSF1, ENPP1, EPHX2, ERAP1, ERAP2, ESR1, ESRRB, ESRRG, EYS, F13A1, F5, FAHD1, FARS2, FAT2, FAT3, FAT4, FBN2, FBP2, FCER2, FGD5, FHIT, FRAS1, FRRS1, FSCB, FSTL5, FTO, FXN, GALC, GALNT10, GALNT2, GALNTL6, GDA, GLB1, GLIS1, GLIS3, GPHN, GPR39, GRIN2A, GRM7, GUCA1A, HAAO, HDAC7, HDHD2, HGD, HMGCLL1, HPCAL4, HPSE, HPSE2, HTR1E, IDO2, IMPA2, ING5, INPP1, ITGA1, ITGA11, ITGA2, ITGA9, ITK, KALRN, KCNIP4, KDM1B, KDM4C, KIAA0528, KL, KLF10, KLF12, KNG1, KSR2, L3MBTL2, L3MBTL4, LDB3, LEPREL1, LHPP, LOXL2, LPP, LRP1B, LTBP1, LTF, LUC7L2, LYAR, MAN1A1, MAN2A1, MANBA, MARCH1, MARCH4, MATN2, ME3, MECOM, MGAT1, MGAT5B, MLL3, MLPH, MMP20, MOB2, MYO5A, MYO9B, MYRIP, NCALD, NCS1, NELL1, NELL2, NID2, NME7, NOTCH4, NPLOC4, NRAP, NRP1, NRXN1, NT5DC3, NUBPL, NUDT13, NUDT3, OAS1, OCM2, OMA1, OVCH1, OVOL2, PADI2, PADI4, PADI6, PAPD4, PAPPA2, PARK2, PCDH15, PCDH17, PCDHA1, PCDHA10, PCDHA11, PCDHA12, PCDHA13, PCDHA2, PCDHA3, PCDHA4, PCDHA5, PCDHA6, PCDHA7, PCDHA8, PCDHA9, PCDHAC1, PCLO, PDE11A, PDE1A, PDE1C, PDE3A, PDE4D, PDE8B, PDLIM1, PDSS1, PDZRN3, PDZRN4, PELO, PFKP, PGLYRP4, PHF11, PINK1, PKD1L2, PKDREJ, PLA2G4D, PLA2G4E, PLCB1, PLCB4, PLCH1, PLCL1, PLSCR1, POMT1, PPA2, PPP2CA, PPP3CA, PRDM5, PRICKLE1, PRIM2, PRKCB, PRKCE, PRUNE2, PTGS1, PXDN, PXDNL, QPCT, RAD18, RASAL1, RBM20, RCN3, RELN, RFPL3, RIMS2, RNF144B, RNF150, RNF175, RNFT2, RNPEP, RORA, RPS6KA2, RTN4IP1, RYR2, RYR3, SCD5, SCN1A, SDF4, SEC24D, SH3RF3, SIK2, SIRT3, SLC25A25, SLC8A1, SLIT3, SMYD3, SNCA, SNTN, SORBS2, SP110, SP140L, SPARCL1, SPOCK3, ST18, STAC, STK32A, STK32B, SVEP1, SWAP70, SYT9, TEC, TES, TET1, THBS2, TIMP2, TMEM163, TNFRSF11A, TPD52, TPO, TRERF1, TRHDE, TRIM10, TRIM21, TRIM22, TRIM40, TRIM5, TRIM9, TRIML1, TSHZ2, TTC3, TYW1, UNC13C, USP20, USP22, VAV1, VAV2, VAV3, VPS8, WT1, ZBED6, ZBTB16, ZBTB49, ZBTB8A, ZC3H11A, ZC3H12C, ZC3H12D, ZC3H3, ZC3H6, ZDHHC14, ZDHHC7, ZFYVE1, ZFYVE28, ZNF100, ZNF155, ZNF180, ZNF184, ZNF195, ZNF211, ZNF257, ZNF283, ZNF285, ZNF331, ZNF354B, ZNF365, ZNF366, ZNF37A, ZNF385D, ZNF391, ZNF426, ZNF440, ZNF441, ZNF443, ZNF45, ZNF460, ZNF492, ZNF543, ZNF568, ZNF578, ZNF595, ZNF670, ZNF677, ZNF714, ZNF717, ZNF718, ZNF727, ZNF761, ZNF763, ZNF766, ZNF780A, ZNF804B, ZNF813, ZNF85, ZSCAN18, ZSWIM2*
Calcium ion binding	1.1×10^{-6}	2.0	*CABP5, CADPS, CAPN14, CCBE1, CDH10, CDH13, CDH4, CDHR2, CLSTN2, CUBN, DNER, DSC1, DSG1, DSG3, DTNA, EFCAB11, ELTD1, EMR1, EYS, FAT2, FBN2, FSTL5, HPCAL4, KCNIP4, LRP1B, LTBP1, MAN1A1, MMP20, MYO5A, NCALD, NID2, NOTCH4, PADI2, PADI4, PADI6, PCDH15, PCDH17, PCDHA1, PCDHA10, PCDHA11, PCDHA12, PCDHA13, PCDHA2, PCDHA3, PCDHA4, PCDHA5, PCDHA6, PCDHA7, PCDHA8, PCDHA9, PCDHAC1, PCLO, PKD1L2, PKDREJ, PLCB1, PLCB4, PLCH1, PLSCR1, RYR2, RYR3, SLC25A25, SNCA, SPARCL1, SWAP70, THBS2, TPD52, TPO*
Rac guanyl-nucleotide exchange factor activity	1.3×10^{-6}	10.3	*ALS2, ARHGEF4, DOCK2, PREX2, SPATA13, VAV1, VAV3*

GO categories in which false discovery rate is less than 0.01.

Table S22 continued.

Description	p-value	Enrichment	Genes
Ion binding	2.4×10^{-6}	1.2	ABCA1, ABCA13, ABCA4, ABCB5, ABCC11, ABCC4, ABCG8, ABI3BP, ABLIM1, ABLIM2, ABO, ACBD5, ACPP, ACSF3, ACSL1, ACTA2, ACTR2, ADAM12, ADAM28, ADAM29, ADAMTS12, ADAMTS14, ADAMTS16, ADAMTS18, ADAMTS20, ADAMTSL1, ADAMTSL3, ADARB2, ADCY3, ADCY5, ADD2, ADH1C, ADH4, AGAP1, AGBL1, AGBL4, AGL, AGMAT, AGTPBP1, AIFM2, AK5, ALDH6A1, ALK, ALPL, AMDHD1, ANGPT2, APIP, APOA1BP, APOBEC3H, APOBEC4, APP, APTX, AQPEP, ARL15, ARSB, ARSG, ARSJ, ASAP1, ATP10B, ATP10D, ATP12A, ATP13A5, ATP2A3, ATP2B4, ATP2C2, ATP8A1, ATP8A2, ATP8B1, B4GALT1, BARD1, BAZ1A, BAZ2B, BBOX1, BCR, BIRC5, BLM, BMPR1B, BNC2, BSPRY, CABP5, CACNA1A, CACNA1C, CACNA2D1, CACNA2D3, CADPS, CADPS2, CALN1, CAMK1D, CAMK2D, CAPN14, CAPN8, CARS2, CASQ2, CBFA2T3, CCBE1, CCL15, CCL23, CCT5, CDC42BPA, CDH10, CDH12, CDH13, CDH18, CDH4, CDH9, CDHR2, CDKAL1, CHD1L, CHD5, CHIA, CHN2, CHRNB3, CLCN6, CLSTN2, CNGA3, COL13A1, COL23A1, COL27A1, COL5A1, COL5A2, COL9A1, COLEC12, CPA3, CPB2, CPE, CTDSPL, CUBN, CYBRD1, CYP2E1, CYP4F11, CYP4F12, CYP4F2, CYP4F3, CYP4F8, CYP4Z1, DAPK1, DCHS2, DCTD, DDC, DDR1, DDX20, DDX60L, DGKB, DGKH, DHX37, DMRT1, DNAH11, DNAH12, DNAH14, DNAH5, DNAH8, DNER, DNM1L, DNTT, DPF3, DPYD, DSC1, DSG1, DSG3, DTNA, DYNC2H1, DYTN, EBF2, EFCAB11, EFCAB4B, EFEMP1, EGFR, EGLN1, ELAC2, ELTD1, EMR1, EMR2, ENDOU, ENOSF1, ENPP1, EPHA6, EPHA7, EPHB1, EPHX2, EPRS, ERAP1, ERAP2, ERBB4, ERO1LB, ESR1, ESRRB, ESRRG, EYA4, EYS, F13A1, F5, FABP2, FAHD1, FARS2, FAT2, FAT3, FAT4, FBN2, FBP2, FCER2, FER, FGD3, FGD4, FGD5, FGF1, FGF14, FGF2, FHIT, FLT3, FMO2, FMO5, FPGT, FRAS1, FREM2, FRRS1, FSCB, FSTL5, FTO, FXN, GADL1, GALC, GALNT10, GALNT2, GALNTL6, GDA, GLB1, GLIS1, GLIS3, GNAL, GPC5, GPC6, GPHN, GPR39, GRIN2A, GRIN3A, GRM7, GUCA1A, HAAO, HADH, HDAC7, HDHD2, HGD, HHAT, HK1, HLCS, HMGCLL1, HPCAL4, HPSE, HPSE2, HTR1E, HUNK, IDO2, IKZF1, IMPA2, ING5, INPP1, INSR, ISCU, ITGA1, ITGA11, ITGA2, ITGA9, ITGAE, ITK, KALRN, KCNIP4, KCNMA1, KDM1B, KDM4C, KIAA0528, KIAA1804, KIF13A, KIF16B, KIF5C, KL, KLF10, KLF12, KNG1, KSR2, L3MBTL2, L3MBTL4, LAMC2, LDB3, LEPREL1, LHPP, LOXL2, LPP, LRP1B, LRRK2, LTBP1, LTF, LUC7L2, LYAR, LYN, MAGI1, MAN1A1, MAN2A1, MANBA, MAP2K3, MAP3K9, MARCH1, MARCH4, MATN2, MCM9, ME3, MECOM, MGAT1, MGAT5B, MGMT, MLL3, MLPH, MMP20, MOB2, MSH3, MTHFD1L, MYH11, MYH13, MYH9, MYLK4, MYO10, MYO16, MYO1B, MYO3A, MYO3B, MYO5A, MYO5B, MYO7A, MYO9B, MYRIP, NAALAD2, NARS2, NAV2, NCALD, NCS1, NEDD4, NELL1, NELL2, NID2, NLRP1, NLRP11, NME7, NME8, NMNAT2, NMUR2, NOTCH4, NPLOC4, NRAP, NRP1, NRXN1, NRXN3, NT5DC3, NUBPL, NUDT13, NUDT3, OAS1, OCM2, OLA1, OMA1, ORC5, OVCH1, OVOL2, PADI2, PADI4, PADI6, PAK2, PAK7, PANK1, PAPD4, PAPL, PAPPA2, PAPSS2, PARD3, PARK2, PASK, PCDH15, PCDH17, PCDHA1, PCDHA10, PCDHA11, PCDHA12, PCDHA13, PCDHA2, PCDHA3, PCDHA4, PCDHA5, PCDHA6, PCDHA7, PCDHA8, PCDHA9, PCDHAC1, PCLO, PDE11A, PDE1A, PDE1C, PDE3A, PDE4D, PDE8B, PDLIM1, PDSS1, PDZRN3, PDZRN4, PELO, PFKP, PGLYRP4, PGS1, PHF11, PIK3C2B, PIK3C2G, PINK1, PKD1L2, PKDREJ, PLA2G4D, PLA2G4E, PLCB1, PLCB4, PLCH1, PLCL1, PLEK, PLOD2, PLSCR1, POMT1, PON3, PPA2, PPP2CA, PPP3CA, PRDM5, PRICKLE1, PRICKLE2, PRIM2, PRKAG2, PRKCA, PRKCB, PRKCE, PRKG1, PRTFDC1, PRUNE2, PSMC1, PTGS1, PTPRF, PXDN, PXDNL, PYGL, QPCT, RAB12, RAB17, RAB20, RAB27A, RAB31, RAB36, RAB3C, RABL5, RAC2, RAD18, RAPGEF4, RASAL1, RBKS, RBM20, RCN3, RELN, RFPL3, RIMS2, RNF144B, RNF150, RNF175, RNFT2, RNPEP, RORA, RPS6KA2, RTCA, RTN4IP1, RXRG, RYR1, RYR2, RYR3, SCARB1, SCD5, SCGN, SCN1A, SCP2, SDF4, SDHA, SEC24D, SELE, SEPT11, SERPINE2, SETX, SH3RF3, SIK2, SIRT3, SLC19A3, SLC22A5, SLC25A25, SLC8A1, SLIT3, SMARCA2, SMCHD1, SMYD3, SNCA, SNTN, SNX9, SOAT2, SORBS2, SP110, SP140L, SPARCL1, SPATA5, SPEF2, SPOCK3, SPTLC3, ST18, STAC, STK32A, STK32B, STK32C, STK39, STYK1, SUCLG2, SVEP1, SWAP70, SYK, SYN3, SYT1, SYT6, SYT9, TAP1, TAP2, TCF19, TEC, TEK, TES, TET1, THBS2, TIMP2, TLK1, TMEM163, TNFRSF11A, TOR1A, TPD52, TPK1, TPO, TRERF1, TRHDE, TRIM10, TRIM21, TRIM22, TRIM40, TRIM5, TRIM69, TRIM9, TRIML1, TRPC4, TRPC6, TSHZ2, TTC3, TTK, TTLL10, TULP3, TYMS, TYW1, UBE2E3, UBE2F, UBE2H, UBE2QL1, UBR3, UBR5, ULK4, UNC13C, USP20, USP22, VARS2, VAV1, VAV2, VAV3, VPS8, VRK3, WNK2, WT1, ZBED6, ZBTB16, ZBTB49, ZBTB8A, ZC3H11A, ZC3H12C, ZC3H12D, ZC3H3, ZC3H6, ZDHHC14, ZDHHC7, ZFYVE1, ZFYVE28, ZNF100, ZNF155, ZNF180, ZNF184, ZNF195, ZNF211, ZNF257, ZNF273, ZNF283, ZNF285, ZNF331, ZNF354B, ZNF365, ZNF366, ZNF37A, ZNF385D, ZNF391, ZNF426, ZNF440, ZNF441, ZNF443, ZNF45, ZNF460, ZNF467, ZNF492, ZNF543, ZNF568, ZNF578, ZNF595, ZNF670, ZNF677, ZNF714, ZNF717, ZNF718, ZNF727, ZNF761, ZNF763, ZNF766, ZNF773, ZNF780A, ZNF804B, ZNF813, ZNF85, ZSCAN18, ZSWIM2
MHC class I receptor activity	4.3×10^{-6}	169.0	HLA-A, HLA-B, HLA-C
Lipid binding	4.5×10^{-6}	1.6	ABCA1, ACBD5, ADH4, ADH5, AFAP1, AFAP1L2, AGAP1, ALS2, APBB1IP, ARHGAP22, ARHGAP23, ARHGAP24, ARHGAP25, ARHGAP26, ARHGAP42, ARHGEF18, ARHGEF4, ASAP1, BCR, CADPS, CADPS2, CDC42BPA, DGKH, DOK6, DYSF, ELMO1, ESR1, ESRRB, ESRRG, FABP2, FER, FGD3, FGD4, FGD5, GRB10, HIP1, HSDL2, INSR, IQSEC1, ITK, ITPR1, ITPR2, KALRN, KIF16B, KL, LAMA1, LAMC1, LYN, MCF2L2, MYO10, MYO1B, MYOF, NOXO1, NPC2, OSBPL11, OSBPL3, PAQR8, PARD3, PASK, PCLO, PHLDB2, PIK3C2B, PIK3C2G, PLA2G4C, PLCB1, PLCG2, PLCH1, PLCL1, PLD1, PLEK, PLEKHA6, PLEKHA7, PLEKHG1, PLEKHH2, PLEKHM2, PREX1, PREX2, PSD3, PTGS1, PYGL, RASAL1, RTKN2, SBF2, SCARB1, SCP2, SGIP1, SH3GL2, SKAP2, SNTG1, SNTG2, SNX19, SNX24, SNX25, SNX29, SNX30, SNX31, SNX5, SNX7, SNX9, SOAT1, SOAT2, SPATA13, STAP1, SWAP70, SYT1, TEC, TIAM1, TIAM2, TULP3, UNC13A, VAV1, VAV2, VAV3, VNN1, ZFYVE1, ZFYVE28
Receptor activity	2.4×10^{-5}	2.3	ABCA1, BMPR1B, CUBN, DMBT1, EMR1, ERBB4, GPR158, GRIA1, GRIN2A, HLA-A, HLA-B, HLA-C, HLA-DPA1, HLA-DQA1, HLA-DQB1, HLA-DRA, HLA-DRB1, HLA-G, ITGBL1, LRP1B, NPSR1, OR10AG1, OR4C3, OR4C45, OR51B6, OR51F1, OR52E6, OR52J3, OR5I1, OR5P2, OR5W2, OR8I2, PGLYRP4, PKHD1, PTPRM, RAMP3, SORCS2

GO categories in which false discovery rate is less than 0.01.
