# Supplementary material for: A Model-Based Approach for Identifying Signatures of Ancient Balancing Selection in Genetic Data
Source: PLoS Genet. 2014 Aug 21;10(8):e1004561. doi: 10.1371/journal.pgen.1004561 (PMC4140648; doi:10.1371/journal.pgen.1004561)
Supplement: Table S23 — GO function analysis of ranked signals from YRI population using the test statistic. (PDF) [file pgen.1004561.s049.pdf]

Table S23: GO function analysis of ranked signals from YRI population using the T_2 test statistic.

Description	p-value	Enrichment	Genes
Metal ion binding	3.3×10^{-11}	1.5	*ABO, ADARB2, ADCY5, ADCY8, ADD2, AGMAT, AGMO, ALPL, ANGPT2, APIP, APOA1BP, ARSB, ARSG, ARSJ, ATP10A, ATP10B, ATP10D, ATP13A5, ATP1A2, ATP2C2, ATP8A1, ATP8A2, BMPR1B, BNIP2, C1GALT1, CACNA1A, CACNA1C, CACNA2D1, CACNA2D3, CADPS, CALN1, CAPN14, CAPN3, CAPN9, CARS2, CASQ2, CCBE1, CDC42BPA, CDH10, CDH12, CDH13, CDH23, CDH4, CDH9, CDKAL1, CELSR2, CHN2, CLSTN2, COL11A1, COL27A1, COL2A1, COL5A1, COLEC12, CPQ, CRTAC1, CUBN, CYB5A, CYBRD1, CYP2E1, CYP2U1, CYP39A1, CYP3A43, CYP4F11, CYP4F12, CYP4F3, CYP4F8, CYP4Z1, DCHS2, DGKB, DGKH, DGKI, DMRT1, DNER, DOHH, DSC1, DSC3, DSG1, DSG3, DTNA, EFCAB11, ELAC2, ELTD1, EMR1, EMR2, ENOSF1, ENPP1, EXO1, EYA2, EYA4, EYS, F13A1, F5, FAHD1, FAM188A, FARS2, FAT2, FAT3, FBLN1, FBP2, FCER2, FGD4, FGD5, FHIT, FOLH1B, FRAS1, FSTL4, FSTL5, FTO, FXN, GALNT10, GALNT2, GALNTL6, GPR39, GRM7, GUCA1A, GUCA1B, HDAC9, HMGCLL1, IDO2, IMPA2, INPP1, ITGA1, ITGA11, ITGA2, ITGA6, ITGA8, ITGA9, ITGAE, KALRN, KCND3, KCNIP1, KCNIP4, KSR2, LCN2, LEPREL1, LHPP, LOXL2, LRP1B, LRP8, LTBP1, MARC1, MARC2, MAST4, MCTP2, ME3, MGAT1, MGMT, MLPH, MMP20, MOB2, MOB3B, MRE11A, MYO5A, MYO9B, NCALD, NELL1, NELL2, NID2, NME7, NOTCH2, NOTCH4, NQO2, NRXN1, NRXN3, NUBPL, NUDT7, OC90, OMA1, P4HA1, PADI1, PADI2, PADI4, PADI6, PAPD4, PCCA, PCDH15, PCDHA1, PCDHA10, PCDHA11, PCDHA12, PCDHA13, PCDHA2, PCDHA3, PCDHA4, PCDHA5, PCDHA6, PCDHA7, PCDHA8, PCDHA9, PCDHAC1, PCLO, PDE11A, PDE1A, PDE1C, PDE3A, PDE4D, PDE6C, PDSS1, PDZD8, PFKP, PGS1, PKD1L2, PKDREJ, PLA2G4E, PLCB1, PLCB4, PLCH1, PLOD2, PLSCR1, POLD1, PON3, PPEF2, PPP2CA, PRIM2, PRKCE, PRKD1, PRLR, PRUNE2, PTGS1, PXDN, PXDNL, RASEF, RCN3, RELN, RIMS1, RIMS2, RNPEP, RPS6KA2, RYR2, RYR3, SCD5, SCN1A, SCN9A, SCUBE1, SDF4, SDHC, SLC25A25, SLIT3, SMOC2, SPARCL1, STAC, STK32A, STK32B, SWAP70, SYT1, SYT10, SYT5, SYT6, SYT9, TESC, TET1, THBS2, TKT, TLL1, TPD52, TYW1, UNC13C, USP32, UTRN, VAV1, VAV2, VAV3, ZFYVE28*
MHC class II receptor activity	8.6×10^{-9}	16.2	*HLA-DOB, HLA-DPA1, HLA-DQA1, HLA-DQA2, HLA-DQB1, HLA-DQB2, HLA-DRA, HLA-DRB1*
Calcium ion binding	7.2×10^{-8}	1.8	*BNIP2, CADPS, CALN1, CAPN14, CAPN3, CAPN9, CASQ2, CCBE1, CDH10, CDH12, CDH13, CDH23, CDH4, CDH9, CELSR2, CLSTN2, CRTAC1, CUBN, DCHS2, DGKB, DNER, DSC1, DSC3, DSG1, DTNA, EFCAB11, ELTD1, EMR1, EMR2, EYS, FAM188A, FAT2, FAT3, FBLN1, FSTL4, FSTL5, GRM7, GUCA1A, GUCA1B, KCNIP1, KCNIP4, LRP1B, LRP8, LTBP1, MCTP2, MGMT, MMP20, MYO5A, NCALD, NELL1, NELL2, NID2, NOTCH2, NOTCH4, OC90, PADI1, PADI2, PADI4, PADI6, PCDH15, PCDHA1, PCDHA10, PCDHA11, PCDHA12, PCDHA13, PCDHA2, PCDHA3, PCDHA4, PCDHA5, PCDHA6, PCDHA7, PCDHA8, PCDHA9, PCDHAC1, PCLO, PGS1, PKD1L2, PKDREJ, PLCB1, PLCB4, PLCH1, PLSCR1, RASEF, RCN3, RYR2, RYR3, SCUBE1, SDF4, SLC25A25, SLIT3, SMOC2, SPARCL1, SWAP70, SYT1, THBS2, TLL1, TPD52, USP32, UTRN*

GO categories in which false discovery rate is less than 0.01.

Table S23 continued.

Description	p-value	Enrichment	Genes
Cation binding	9.6×10^{-8}	1.3	*ABLIM1, ABLIM3, ABO, ACPP, ADAM12, ADAM28, ADAM29, ADAM32, ADAMDEC1, ADAMTS12, ADAMTS16, ADAMTS2, ADAMTSL2, ADAMTSL3, ADARB2, ADAT2, ADCY5, ADCY8, ADD2, ADH1C, ADH4, AGAP1, AGBL1, AGBL3, AGBL4, AGMAT, AGMO, AGTPBP1, ALPL, AMZ1, ANGPT2, APIP, APOA1BP, APOBEC3H, APOBEC4, ARSB, ARSG, ARSJ, ASAP1, ATP10A, ATP10B, ATP10D, ATP13A5, ATP1A2, ATP2C2, ATP8A1, ATP8A2, BARD1, BAZ2B, BMPR1B, BNC2, BNIP2, BSPRY, C1GALT1, CACNA1A, CACNA1C, CACNA2D1, CACNA2D3, CADPS, CALN1, CAPN14, CAPN3, CAPN9, CARS2, CASQ2, CBFA2T3, CCBE1, CDC42BPA, CDH10, CDH12, CDH13, CDH23, CDH4, CDH9, CDKAL1, CELSR2, CHIA, CHN2, CHRNB3, CLSTN2, COL11A1, COL27A1, COL2A1, COL5A1, COLEC12, CPA3, CPB2, CPE, CPQ, CREB5, CRTAC1, CUBN, CYB5A, CYBRD1, CYP2E1, CYP2U1, CYP39A1, CYP3A43, CYP4F11, CYP4F12, CYP4F3, CYP4F8, CYP4Z1, DCHS2, DCTD, DGKB, DGKH, DGKI, DMRT1, DNER, DOHH, DPYD, DSC1, DSC3, DSG1, DSG3, DTNA, DYTN, EFCAB11, ELAC2, ELTD1, EMR1, EMR2, ENOSF1, ENPP1, ERAP1, ERAP2, ESR1, ESRRB, ESRRG, EXO1, EYA2, EYA4, EYS, F13A1, F5, FAHD1, FAM170A, FAM188A, FARS2, FAT2, FAT3, FBLN1, FBP2, FCER2, FGD4, FGD5, FHIT, FOLH1B, FRAS1, FRRS1, FSTL4, FSTL5, FTO, FXN, GALC, GALNT10, GALNT2, GALNTL6, GBA3, GCM1, GDA, GLB1, GLIS1, GLIS3, GLRA3, GPR39, GRIN2A, GRM7, GUCA1A, GUCA1B, HAGH, HDAC9, HMGCLL1, HPSE2, IDO2, IKZF1, IKZF3, IMPA2, INPP1, ITGA1, ITGA11, ITGA2, ITGA6, ITGA8, ITGA9, ITGAE, KALRN, KCND3, KCNIP1, KCNIP4, KDM1B, KDM4C, KL, KLF12, KNG1, KSR2, L3MBTL2, L3MBTL4, LCN2, LCT, LDB3, LEPREL1, LHPP, LIMD1, LMCD1, LOXL2, LPP, LRP1B, LRP8, LTBP1, MANBA, MARC1, MARC2, MARCH1, MARCH4, MAST4, MCTP2, ME3, MECOM, MGAT1, MGAT5B, MGMT, MLL3, MLPH, MMP20, MOB2, MOB3B, MRE11A, MYO5A, MYO9B, MYRIP, MYT1L, NCALD, NELL1, NELL2, NEURL1B, NID2, NME7, NOTCH2, NOTCH4, NPEPPS, NQO2, NR1H2, NR3C1, NRAP, NRXN1, NRXN3, NUBPL, NUDT7, OC90, OMA1, P4HA1, PADI1, PADI2, PADI4, PADI6, PAPD4, PARK2, PCCA, PCDH15, PCDHA1, PCDHA10, PCDHA11, PCDHA12, PCDHA13, PCDHA2, PCDHA3, PCDHA4, PCDHA5, PCDHA6, PCDHA7, PCDHA8, PCDHA9, PCDHAC1, PCLO, PDE11A, PDE1A, PDE1C, PDE3A, PDE4D, PDE6C, PDLIM1, PDLIM5, PDSS1, PDZD8, PDZRN4, PFKP, PGLYRP4, PGS1, PHF11, PHF17, PIKFYVE, PKD1L2, PKDREJ, PLA2G4E, PLCB1, PLCB4, PLCH1, PLOD2, PLSCR1, POLD1, PON3, PPEF2, PPP2CA, PRICKLE1, PRIM2, PRKCA, PRKCB, PRKCE, PRKD1, PRLR, PRUNE2, PTGS1, PXDN, PXDNL, RASEF, RCN3, RELN, RIMS1, RIMS2, RNF144B, RNF150, RNF212, RNFT2, RNPEP, RORA, RPS6KA2, RTN4IP1, RYR2, RYR3, SCD5, SCN1A, SCN9A, SCUBE1, SDF4, SDHC, SH3RF3, SLC25A25, SLIT3, SMOC2, SMYD1, SMYD3, SORBS2, SORD, SP110, SP140L, SPARCL1, SREK1IP1, ST18, STAC, STK32A, STK32B, SWAP70, SYT1, SYT10, SYT5, SYT6, SYT9, TCF19, TESC, TET1, THBS2, TKT, TLL1, TMEM163, TPD52, TRHDE, TRIM10, TRIM40, TRIM5, TRIM9, TRIML1, TSHZ2, TTC3, TYW1, UBR2, UNC13C, USP20, USP32, UTRN, VAV1, VAV2, VAV3, VPS8, YAF2, ZBTB16, ZBTB49, ZBTB7C, ZBTB8A, ZC3H12C, ZC3H6, ZCCHC4, ZDHHC14, ZDHHC7, ZFP57, ZFP64, ZFYVE28, ZNF107, ZNF155, ZNF160, ZNF18, ZNF195, ZNF211, ZNF257, ZNF280A, ZNF283, ZNF323, ZNF331, ZNF33B, ZNF354B, ZNF365, ZNF366, ZNF37A, ZNF385D, ZNF391, ZNF423, ZNF425, ZNF441, ZNF443, ZNF45, ZNF473, ZNF492, ZNF510, ZNF543, ZNF568, ZNF578, ZNF675, ZNF677, ZNF716, ZNF717, ZNF727, ZNF729, ZNF761, ZNF766, ZNF773, ZNF808, ZNF83, ZNF835, ZNF85, ZNF879, ZNF90, ZSCAN18, ZSWIM2, ZSWIM4*

GO categories in which false discovery rate is less than 0.01.

Table S23 continued.

Description	p-value	Enrichment	Genes
Ion binding	2.8×10^{-7}	1.2	*ABCA1, ABCA12, ABCA13, ABCA4, ABCB11, ABCB5, ABCC4, ABCC8, ABCD4, ABI3BP, ABLIM1, ABLIM3, ABO, ACBD5, ACPP, ACSBG2, ACSF3, ADAM12, ADAM28, ADAM29, ADAM32, ADAMDEC1, ADAMTS12, ADAMTS16, ADAMTS2, ADAMTSL2, ADAMTSL3, ADARB2, ADAT2, ADCY5, ADCY8, ADD2, ADH1C, ADH4, AGAP1, AGBL1, AGBL3, AGBL4, AGMAT, AGMO, AGTPBP1, AK3, AK5, ALK, ALPL, AMZ1, ANGPT2, APIP, APOA1BP, APOBEC3H, APOBEC4, ARL15, ARSB, ARSG, ARSJ, ASAP1, ASCC3, ATP10A, ATP10B, ATP10D, ATP13A5, ATP1A2, ATP2C2, ATP8A1, ATP8A2, BARD1, BAZ2B, BCR, BLK, BLM, BMP2K, BMPR1B, BNC2, BNIP2, BSPRY, C1GALT1, CACNA1A, CACNA1C, CACNA2D3, CADPS, CALN1, CAMK2D, CAPN14, CAPN3, CAPN9, CARS2, CASQ2, CBFA2T3, CCBE1, CCBL2, CCT5, CDC42BPA, CDH10, CDH12, CDH13, CDH23, CDH4, CDH9, CDKAL1, CELSR2, CHIA, CHN2, CHRNB3, CLSTN2, CNGA3, COL11A1, COL23A1, COL25A1, COL27A1, COL2A1, COL5A1, COLEC12, CPA3, CPB2, CPE, CPQ, CREB5, CRTAC1, CUBN, CYB5A, CYBRD1, CYP2E1, CYP2U1, CYP39A1, CYP3A43, CYP4F11, CYP4F12, CYP4F3, CYP4F8, CYP4Z1, DCHS2, DCTD, DDC, DDR2, DGKB, DGKH, DGKI, DHX37, DMGDH, DMRT1, DNAH11, DNAH17, DNAH5, DNAH8, DNAH9, DNER, DNM3, DOHH, DPYSL3, DSC1, DSC3, DSG1, DSG3, DTNA, DYNC2H1, DYTN, EFCAB11, EGFR, EIF2AK4, ELAC2, ELTD1, EMR1, EMR2, ENOSF1, ENPP1, EPHA3, EPHA6, EPRS, ERAP1, ERAP2, ERBB4, ERO1LB, ESR1, ESRRB, ESRRG, EXO1, EYA2, EYA4, EYS, F13A1, F5, FABP2, FAHD1, FAM170A, FAM188A, FARS2, FAT2, FAT3, FBLN1, FBP2, FCER2, FER, FGD4, FGD5, FGF1, FGF12, FGF14, FGF2, FHIT, FLT3, FMO2, FMO4, FMO5, FOLH1B, FRAS1, FSTL4, FSTL5, FTO, FXN, GADL1, GALC, GALNT10, GALNT2, GALNTL6, GBA3, GCM1, GDA, GLB1, GLIS1, GLIS3, GLRA3, GMDS, GPC5, GPC6, GPNMB, GPR39, GRIK1, GRIN2A, GRM7, GUCA1A, GUCA1B, HADHB, HAGH, HDAC9, HHAT, HK1, HLCS, HMGCLL1, HPSE2, HSPA12A, IDO2, IKZF3, IMPA2, INPP1, IQCA1, ITGA1, ITGA11, ITGA2, ITGA6, ITGA8, ITGA9, ITGAE, KALRN, KCND3, KCNIP1, KCNIP4, KDM1B, KDM4C, KIAA0564, KIAA1804, KIF13A, KIF16B, KIF21B, KL, KLF12, KNG1, KSR2, L3MBTL2, L3MBTL4, LAMC2, LCN2, LCT, LDB3, LEPREL1, LHPP, LIMD1, LMCD1, LOXL2, LPP, LRGUK, LRP1B, LRP8, LTBP1, MAGI1, MANBA, MAP2K3, MARC1, MARC2, MARCH1, MARCH4, MAST4, MCM9, MCTP2, ME3, MECOM, MGAT1, MGMT, MLL3, MLPH, MMP20, MOB2, MOB3B, MRE11A, MSH3, MTHFD1, MTHFD1L, MX1, MYH13, MYH9, MYLK4, MYO10, MYO16, MYO18B, MYO1B, MYO3A, MYO5A, MYO5B, MYO9B, MYRIP, MYT1L, NADSYN1, NAV2, NCALD, NEDD4, NELL1, NELL2, NEURL1B, NID2, NLRP1, NLRP11, NLRP2, NLRP5, NME7, NMNAT2, NOTCH2, NOTCH4, NPEPPS, NR1H2, NR3C1, NRAP, NRXN1, NRXN3, NTRK2, NTRK3, NUBPL, NUDT7, OC90, OMA1, ORC5, P4HA1, PADI1, PADI2, PADI4, PADI6, PANK1, PAPD4, PAPSS2, PARD3, PARK2, PCCA, PCDH15, PCDHA1, PCDHA10, PCDHA11, PCDHA12, PCDHA13, PCDHA2, PCDHA3, PCDHA4, PCDHA5, PCDHA6, PCDHA7, PCDHA8, PCDHA9, PCDHAC1, PCLO, PDE11A, PDE1A, PDE1C, PDE3A, PDE4D, PDE6C, PDIK1L, PDLIM1, PDSS1, PDZD8, PDZRN4, PFKFB2, PFKP, PGLYRP4, PGS1, PHF11, PHF17, PIK3C2G, PIKFYVE, PKD1L2, PKDREJ, PLA2G4E, PLCB1, PLCB4, PLCH1, PLOD2, PLSCR1, POLD1, PON3, PPEF2, PPP2CA, PRICKLE1, PRIM2, PRKAG2, PRKCA, PRKCB, PRKCE, PRKD1, PRKG1, PRKG2, PRLR, PRUNE2, PSMC1, PTCH1, PTGS1, PXDNL, RAB27A, RAB36, RAD51B, RAD54L, RAPGEF4, RASEF, RCN3, RELN, RIMS1, RIMS2, RNF144B, RNF150, RNF212, RNFT2, RNGTT, RNPEP, ROR1, RORA, RPS6KA2, RTN4IP1, RYR2, RYR3, SCARB1, SCD5, SCLY, SCN1A, SCN9A, SCP2, SCUBE1, SDF4, SDHA, SDHC, SEPT11, SERPINE2, SH3RF3, SLC19A3, SLC25A25, SLC27A2, SLIT3, SMC2, SMOC2, SMYD1, SMYD3, SORBS2, SORD, SP110, SP140L, SPARCL1, SPEF2, SPTLC3, SRD5A1, SREK1IP1, ST18, STAC, STK32A, STK32B, STK39, STYK1, SUCLG2, SWAP70, SYK, SYN3, SYT1, SYT10, SYT5, SYT6, SYT9, TAP2, TEK, TET1, THBS2, TKT, TLK1, TLL1, TMEM163, TPD52, TPK1, TRHDE, TRIM10, TRIM40, TRIM5, TRIM9, TRIML1, TRPC4, TRPC6, TSHZ2, TTC3, TTLL2, TUBB, TULP3, TXNRD2, TYMS, TYW1, UBE2E3, UBR2, UGT1A7, UGT1A8, UGT1A9, ULK4, UNC13C, USP20, USP32, UTRN, VAV1, VAV2, VAV3, VPS8, VRK3, XYLB, YAF2, ZBTB16, ZBTB49, ZBTB7C, ZBTB8A, ZC3H12C, ZC3H6, ZCCHC4, ZDHHC14, ZDHHC7, ZFP57, ZFP64, ZFYVE28, ZNF107, ZNF155, ZNF160, ZNF18, ZNF195, ZNF211, ZNF257, ZNF280A, ZNF283, ZNF323, ZNF331, ZNF33B, ZNF354B, ZNF365, ZNF366, ZNF37A, ZNF385D, ZNF391, ZNF423, ZNF425, ZNF441, ZNF443, ZNF45, ZNF473, ZNF492, ZNF510, ZNF543, ZNF568, ZNF578, ZNF675, ZNF677, ZNF717, ZNF727, ZNF729, ZNF761, ZNF766, ZNF773, ZNF83, ZNF835, ZNF85, ZNF879, ZNF90, ZSCAN18, ZSWIM2, ZSWIM4*
Phospholipid binding	5.1×10^{-7}	1.8	*ABCA1, AFAP1, AGAP1, ALS2, ANXA4, APBB1IP, ARAP1, ARHGAP15, ARHGAP22, ARHGAP24, ARHGAP25, ARHGAP26, ARHGAP42, ARHGEF18, ASAP1, BCR, CADPS, CADPS2, CCDC88A, CDC42BPA, CIT, DGKH, DNM3, DOCK10, DOK5, DOK6, DYSF, ELMO1, FGD4, FGD5, GRB10, GRK5, HS1BP3, IQSEC1, KALRN, KIF16B, MCF2L2, MYO10, MYO1B, MYOF, OSBPL5, PARD3, PCLO, PHLDB2, PIK3C2G, PLA2G4C, PLCB1, PLCG2, PLCH1, PLD1, PLEKHA6, PLEKHA7, PLEKHG1, PLEKHG4B, PLEKHH2, PLEKHM3, PREX2, PRKD1, PSD3, PXK, RGNEF, SBF2, SCARB1, SGIP1, SKAP2, SNTB1, SNTG1, SNTG2, SNX19, SNX29, SNX31, SNX7, SPATA13, SWAP70, SYT1, TEX2, TIAM1, TIAM2, TULP3, VAV1, VAV2, VAV3, VEPH1, VNN1, VPS36, ZFYVE28*
Glutamate receptor activity	5.4×10^{-7}	10.8	*GRIA1, GRID1, GRID2, GRIK2, GRIN2A, GRM1, GRM5, GRM7, GRM8*
Actin binding	2.0×10^{-6}	1.9	*ABLIM1, ABLIM3, ADD2, AFAP1, ARPC5, CAP1, CAP2, CCDC88A, CLMN, COBLL1, CORO2B, COTL1, DAAM2, EGFR, FBXO25, FER, FGD4, FHOD3, FLNB, FMN1, FMN2, GAS7, IQGAP2, KLHL1, KLHL3, KLHL5, MAEA, MLPH, MTSS1, MYH13, MYH14, MYH9, MYO10, MYO16, MYO18B, MYO1B, MYO1F, MYO1H, MYO3A, MYO5A, MYO5B, MYO9B, MYPN, MYRIP, NCALD, NRAP, PALLD, PARVA, PDLIM5, PHACTR1, PHACTR3, PKNOX2, PRKCE, PXK, RCSD1, SHROOM3, SNTB1, SNTG1, SNTG2, SPTB, SVIL, SYNE1, TNS1, UTRN, XIRP2*
Ion gated channel activity	2.3×10^{-6}	2.2	*ANO2, ANO6, ASIC2, CACNA1A, CACNA1C, CACNA2D3, CHRNB3, CLCA2, CLCNKB, CNGA3, FGF2, GABRB3, GABRG3, GABRR1, GLRA3, GRIA1, GRID1, GRID2, GRIK1, GRIK2, GRIK4, GRIN2A, GRM7, KCNA6, KCNAB1, KCNC2, KCND3, KCNH8, KCNIP1, KCNIP4, KCNJ12, KCNJ6, KCNK10, KCNK18, KCNK2, KCNMB3, KCNQ5, NALCN, PEX5L, RYR2, RYR3, SCN11A, SCN3A, SCN3B, SCN9A, SCNN1G, TRPC4, TRPC6*

GO categories in which false discovery rate is less than 0.01.

Table S23 continued.

Description	p-value	Enrichment	Genes
Gated channel activity	2.3×10^{-6}	2.2	*ANO2, ANO6, ASIC2, CACNA1A, CACNA1C, CACNA2D3, CHRNB3, CLCA2, CLCNKB, CNGA3, FGF2, GABRB3, GABRG3, GABRR1, GLRA3, GRIA1, GRID1, GRID2, GRIK1, GRIK2, GRIK4, GRIN2A, GRM7, KCNA6, KCNAB1, KCNC2, KCND3, KCNH8, KCNIP1, KCNIP4, KCNJ12, KCNJ6, KCNK10, KCNK18, KCNK2, KCNMB3, KCNQ5, NALCN, PEX5L, RYR2, RYR3, SCN11A, SCN3A, SCN3B, SCN9A, SCNN1G, TRPC4, TRPC6*
Ion channel activity	2.7×10^{-6}	1.9	*ABCC8, ANO2, ANO6, ASIC2, BEST3, CACNA1A, CACNA1C, CACNA2D1, CACNA2D3, CHRNB3, CLCA2, CLCNKB, CNGA3, FGF2, FXYD6, GABRB3, GABRG3, GABRR1, GLRA3, GPM6A, GRIA1, GRID1, GRID2, GRIK1, GRIK2, GRIK4, GRIN2A, GRM7, KCNA6, KCNAB1, KCNB2, KCNC2, KCND3, KCNE1, KCNH5, KCNH7, KCNH8, KCNIP1, KCNIP4, KCNJ12, KCNJ6, KCNK10, KCNK18, KCNK2, KCNMB2, KCNMB3, KCNQ5, KCNV2, LRRC52, MCOLN2, NALCN, PDE2A, PEX5L, PIEZO2, PKD1L2, PKDREJ, RYR2, RYR3, SCN11A, SCN1A, SCN3A, SCN3B, SCN9A, SCNN1G, SLC26A7, TRPA1, TRPC4, TRPC6, TRPM3*
Substrate-specific channel activity	6.1×10^{-6}	1.8	*ABCC8, ANO2, ANO6, ASIC2, BEST3, CACNA1A, CACNA1C, CACNA2D1, CACNA2D3, CHRNB3, CLCA2, CLCNKB, CNGA3, FGF2, FXYD6, GABRB3, GABRG3, GABRR1, GLRA3, GPM6A, GRIA1, GRID1, GRID2, GRIK1, GRIK2, GRIK4, GRIN2A, GRM7, KCNA6, KCNAB1, KCNB2, KCNC2, KCND3, KCNE1, KCNH5, KCNH7, KCNH8, KCNIP1, KCNIP4, KCNJ12, KCNJ6, KCNK10, KCNK18, KCNK2, KCNMB2, KCNMB3, KCNQ5, KCNV2, LRRC52, MCOLN2, NALCN, PDE2A, PEX5L, PIEZO2, PKD1L2, PKDREJ, RYR2, RYR3, SCN11A, SCN1A, SCN3A, SCN3B, SCN9A, SCNN1G, SLC26A7, TRPA1, TRPC4, TRPC6, TRPM3*
Passive transmembrane transporter activity	2.3×10^{-5}	1.7	*ABCC8, ANO2, ANO6, ASIC2, BEST3, CACNA1A, CACNA1C, CACNA2D1, CACNA2D3, CHRNB3, CLCA2, CLCNKB, CNGA3, FGF2, FXYD6, GABRB3, GABRG3, GABRR1, GLRA3, GPM6A, GRIA1, GRID1, GRID2, GRIK1, GRIK2, GRIK4, GRIN2A, GRM7, KCNA6, KCNAB1, KCNB2, KCNC2, KCND3, KCNE1, KCNH5, KCNH7, KCNH8, KCNIP1, KCNIP4, KCNJ12, KCNJ6, KCNK10, KCNK18, KCNK2, KCNMB2, KCNMB3, KCNQ5, KCNV2, LRRC52, MCOLN2, NALCN, PDE2A, PEX5L, PIEZO2, PKD1L2, PKDREJ, RYR2, RYR3, SCN11A, SCN1A, SCN3A, SCN3B, SCN9A, SCNN1G, SLC26A7, TRPA1, TRPC4, TRPC6, TRPM3*
Channel activity	2.3×10^{-5}	1.7	*ABCC8, ANO2, ANO6, ASIC2, BEST3, CACNA1A, CACNA1C, CACNA2D1, CACNA2D3, CHRNB3, CLCA2, CLCNKB, CNGA3, FGF2, FXYD6, GABRB3, GABRG3, GABRR1, GLRA3, GPM6A, GRIA1, GRID1, GRID2, GRIK1, GRIK2, GRIK4, GRIN2A, GRM7, KCNA6, KCNAB1, KCNB2, KCNC2, KCND3, KCNE1, KCNH5, KCNH7, KCNH8, KCNIP1, KCNIP4, KCNJ12, KCNJ6, KCNK10, KCNK18, KCNK2, KCNMB2, KCNMB3, KCNQ5, KCNV2, LRRC52, MCOLN2, NALCN, PDE2A, PEX5L, PIEZO2, PKD1L2, PKDREJ, RYR2, RYR3, SCN11A, SCN1A, SCN3A, SCN3B, SCN9A, SCNN1G, SLC26A7, TRPA1, TRPC4, TRPC6, TRPM3*
Transmembrane transporter activity	3.2×10^{-5}	1.5	*ABCA1, ABCA4, ABCB11, ABCB5, ABCC12, ABCC4, ABCC6, ABCC8, ABCD4, ANKH, ANO2, ANO6, ASIC2, ATP10A, ATP10B, ATP10D, ATP12A, ATP13A5, ATP1A2, ATP2C2, ATP6V0A4, ATP6V0E2, ATP8A1, ATP8A2, BEST3, CACNA1A, CACNA1C, CACNA2D1, CACNA2D3, CHRNB3, CLCA2, CLCNKB, CNGA3, CTNS, CYB5A, FGF2, FXYD6, GABRB3, GABRG3, GABRR1, GLRA3, GPM6A, GRIA1, GRID1, GRID2, GRIK1, GRIK2, GRIK4, GRIN2A, GRM7, KCNA6, KCNAB1, KCNB2, KCNC2, KCND3, KCNE1, KCNH5, KCNH7, KCNH8, KCNIP1, KCNIP4, KCNJ12, KCNJ6, KCNK10, KCNK18, KCNK2, KCNMB2, KCNMB3, KCNQ5, KCNV2, LRRC52, MCOLN2, NALCN, OCA2, PDE2A, PEX5L, PIEZO2, PKD1L2, PKDREJ, PQLC2, RYR2, RYR3, SCN11A, SCN1A, SCN3A, SCN3B, SCN9A, SCNN1G, SLC12A3, SLC12A6, SLC12A8, SLC14A2, SLC15A2, SLC15A5, SLC16A14, SLC16A9, SLC17A5, SLC19A3, SLC1A2, SLC1A6, SLC1A7, SLC22A16, SLC22A23, SLC24A3, SLC24A4, SLC26A7, SLC28A3, SLC2A9, SLC36A2, SLC39A11, SLC39A12, SLC39A14, SLC5A12, SLC6A15, SLC7A1, SLC7A13, SLC9A4, SLC9A9, SLC9C1, SLCO1B3, SV2B, SV2C, TAP2, TRPA1, TRPC4, TRPC6, TRPM3*

GO categories in which false discovery rate is less than 0.01.
