# Supplementary material for: A Model-Based Approach for Identifying Signatures of Ancient Balancing Selection in Genetic Data
Source: PLoS Genet. 2014 Aug 21;10(8):e1004561. doi: 10.1371/journal.pgen.1004561 (PMC4140648; doi:10.1371/journal.pgen.1004561)
Supplement: Table S24 — GO component analysis of ranked signals from CEU population using the test statistic. (PDF) [file pgen.1004561.s050.pdf]

Table S24: GO component analysis of ranked signals from CEU population using the T_1 test statistic.

Description	p-value	Enrichment	Genes
Integral to lumenal side of endoplasmic reticulum membrane	1.7×10^{-17}	273.3	*HLA-A*, *HLA-B*, *HLA-C*, *HLA-DPA1*, *HLA-DPB1*, *HLA-DQA1*, *HLA-DQB1*, *HLA-DRB1*
ER to Golgi transport vesicle membrane	1.1×10^{-16}	227.7	*HLA-A*, *HLA-B*, *HLA-C*, *HLA-DPA1*, *HLA-DPB1*, *HLA-DQA1*, *HLA-DQB1*, *HLA-DRB1*
Transport vesicle membrane	1.5×10^{-16}	139.8	*CPE*, *HLA-A*, *HLA-B*, *HLA-C*, *HLA-DPA1*, *HLA-DPB1*, *HLA-DQA1*, *HLA-DQB1*, *HLA-DRB1*
MHC protein complex	3.9×10^{-16}	200.9	*HLA-A*, *HLA-B*, *HLA-C*, *HLA-DPA1*, *HLA-DPB1*, *HLA-DQA1*, *HLA-DQB1*, *HLA-DRB1*
Endocytic vesicle membrane	7.7×10^{-15}	96.1	*DMBT1*, *HLA-A*, *HLA-B*, *HLA-C*, *HLA-DPA1*, *HLA-DPB1*, *HLA-DQA1*, *HLA-DQB1*, *HLA-DRB1*
MHC class II protein complex	3.3×10^{-13}	71.9	*HLA-DPA1*, *HLA-DPB1*, *HLA-DQA1*, *HLA-DQB1*, *HLA-DQB2*, *HLA-DRA*, *HLA-DRB1*, *HLA-DRB5*
Integral to endoplasmic reticulum membrane	6.2×10^{-13}	80.4	*HLA-A*, *HLA-B*, *HLA-C*, *HLA-DPA1*, *HLA-DPB1*, *HLA-DQA1*, *HLA-DQB1*, *HLA-DRB1*
Plasma membrane	9.0×10^{-13}	1.5	*ABCA1*, *ABCA4*, *ABCB5*, *ABCC4*, *ABCG8*, *ACPP*, *ADAM12*, *ADAM28*, *ADCY3*, *ADCY5*, *ADD2*, *ADRA1A*, *ALPL*, *ANGPT1*, *ANGPT2*, *ANK2*, *ANK3*, *ANKS1B*, *ANO2*, *APBB1IP*, *ARHGAP24*, *ARHGEF18*, *ATP10D*, *ATP12A*, *ATP1A4*, *ATP8A1*, *ATP8B1*, *BAI3*, *BBS9*, *BLNK*, *BMPR1B*, *BNC2*, *BTN3A2*, *CACNA1A*, *CADM2*, *CAMK2D*, *CAP1*, *CDH10*, *CDH13*, *CDH4*, *CHD1L*, *CHL1*, *CHRNB3*, *CLDN10*, *CLDN14*, *CLSTN2*, *CNGA3*, *CNR2*, *CNTN1*, *CNTN4*, *CNTN5*, *COL13A1*, *CPE*, *CSMD2*, *CSMD3*, *CUBN*, *CXADR*, *CYBRD1*, *DAAM1*, *DAPP1*, *DCC*, *DCHS2*, *DGKH*, *DIO1*, *DISC1*, *DLG2*, *DLGAP1*, *DNAAF1*, *DNER*, *DSC1*, *DSCAM*, *DSG1*, *DTNA*, *DYM*, *DYNC2H1*, *DYSF*, *DYTN*, *ELTD1*, *EMID2*, *ENDOU*, *ERBB4*, *ESYT2*, *EXOC2*, *F2RL2*, *F5*, *FABP2*, *FAS*, *FAT2*, *FAT4*, *FHIT*, *FLVCR1*, *FMN1*, *FNDC1*, *FRAS1*, *FRMPD1*, *FSHR*, *GABRR1*, *GPC5*, *GPC6*, *GPR111*, *GPR139*, *GPR158*, *GPR39*, *GPR78*, *GRB10*, *GRIA1*, *GRIK2*, *GRIK3*, *GRIN2A*, *GRIP1*, *GRM5*, *GRM7*, *GRM8*, *HLA-A*, *HLA-B*, *HLA-C*, *HLA-DOA*, *HLA-DPA1*, *HLA-DPB1*, *HLA-DQA1*, *HLA-DQA2*, *HLA-DQB1*, *HLA-DQB2*, *HLA-DRA*, *HLA-DRB1*, *HLA-DRB5*, *HLA-F*, *HLA-G*, *HPSE2*, *IGSF5*, *INADL*, *INPP5D*, *ITGA1*, *ITGA2*, *ITGA9*, *ITGB5*, *IYD*, *KCNA6*, *KCNAB1*, *KCNB2*, *KCNIP4*, *KCNJ12*, *KCNJ16*, *KCNK2*, *KCNMB2*, *KCNMB3*, *KCNQ4*, *KCNQ5*, *KIRREL3*, *KL*, *KLRB1*, *KNG1*, *LAMA2*, *LDLRAD3*, *LHCGR*, *LRRC52*, *LRRC7*, *LRRTM4*, *LYPD6B*, *MAGI2*, *MAPT*, *MCC*, *MDGA2*, *MTUS1*, *MYO10*, *MYO16*, *MYO1B*, *MYOF*, *NEDD4*, *NETO1*, *NFASC*, *NGEF*, *NKAIN3*, *NOTCH4*, *NPSR1*, *NRCAM*, *NRP1*, *NRXN1*, *NSL1*, *NTM*, *NTN4*, *OPCML*, *OPRK1*, *OR10AG1*, *OR10C1*, *OR12D2*, *OR13C5*, *OR14A16*, *OR1L8*, *OR1N2*, *OR1S1*, *OR2AK2*, *OR2L13*, *OR2T4*, *OR2W3*, *OR4C3*, *OR4C45*, *OR51B2*, *OR51B6*, *OR51E1*, *OR51F1*, *OR51I2*, *OR51M1*, *OR51Q1*, *OR52E2*, *OR52E6*, *OR52J3*, *OR52N2*, *OR52N4*, *OR52R1*, *OR5AC2*, *OR5F1*, *OR5I1*, *OR5P2*, *OR5W2*, *OR8G1*, *OR8G5*, *OR8H3*, *OR8I2*, *OR8U8*, *OR9G1*, *OR9G9*, *PALM2*, *PCDH15*, *PCDHA12*, *PCDHA13*, *PCDHA9*, *PDIA6*, *PHLDB2*, *PKP1*, *PLA2R1*, *PLCG2*, *PLSCR1*, *PLXNA4*, *PPFIBP1*, *PPYR1*, *PRKCE*, *PRKG1*, *PROKR2*, *PSD3*, *PTGER3*, *PTPN13*, *PXK*, *RAB31*, *RAB3C*, *RAMP3*, *RAPGEF4*, *RFTN1*, *RGS6*, *RIMBP2*, *RIMS1*, *RIMS2*, *RNPEP*, *ROR1*, *RPSA*, *RYR2*, *SCARB1*, *SEMA5A*, *SEMA6D*, *SGCG*, *SGCZ*, *SGIP1*, *SH3GL2*, *SIRPA*, *SKAP2*, *SLC12A6*, *SLC15A2*, *SLC16A14*, *SLC19A3*, *SLC1A2*, *SLC1A6*, *SLC22A16*, *SLC22A5*, *SLC24A4*, *SLC27A6*, *SLC28A1*, *SLC2A9*, *SLC9A4*, *SLCO1B1*, *SLCO1B3*, *SLCO5A1*, *SNCA*, *SNTB1*, *SNTG2*, *SPRED1*, *SVIL*, *SWAP70*, *SYK*, *TANC1*, *TAP1*, *TAP2*, *TEC*, *TEK*, *TES*, *TESC*, *THSD7A*, *TIAM1*, *TJP2*, *TPO*, *TRPC4*, *TRPC6*, *TSHR*, *TULP3*, *UNC13C*, *VAV3*, *VNN1*, *WWOX*
Cytoplasmic vesicle membrane	1.9×10^{-12}	29.7	*CPE*, *DMBT1*, *HLA-A*, *HLA-B*, *HLA-C*, *HLA-DPA1*, *HLA-DPB1*, *HLA-DQA1*, *HLA-DQB1*, *HLA-DRB1*
Intrinsic to endoplasmic reticulum membrane	2.7×10^{-12}	67.6	*HLA-A*, *HLA-B*, *HLA-C*, *HLA-DPA1*, *HLA-DPB1*, *HLA-DQA1*, *HLA-DQB1*, *HLA-DRB1*
Vesicle membrane	3.1×10^{-12}	28.5	*CPE*, *DMBT1*, *HLA-A*, *HLA-B*, *HLA-C*, *HLA-DPA1*, *HLA-DPB1*, *HLA-DQA1*, *HLA-DQB1*, *HLA-DRB1*

GO categories in which false discovery rate is less than 0.01.

Table S24 continued.

Description	p-value	Enrichment	Genes
Membrane part	3.6×10^{-12}	1.2	*AADAC, AADACL2, ABCA1, ABCA13, ABCA4, ABCB5, ABCC11, ABCC4, ABCG8, ABO, ACER1, ACPP, ADAM12, ADAM28, ADAM29, ADCY3, ADCY5, ADCY8, ADRA1A, AIFM2, AJAP1, AKAP6, ALK, ALPL, ANGPT1, ANK2, ANK3, ANO1, ANO2, ANO3, ANXA6, AP1S3, AP3B1, APP, AQP8, AQP9, AQPEP, ART3, ASAH2, ASGR2, ASIC2, ASTN2, ATP10B, ATP10D, ATP12A, ATP1A4, ATP1B3, ATP2C2, ATP5C1, ATP6V0E2, ATP6V1C2, ATP6V1G2, ATP8A1, ATP8A2, ATP8B1, ATP9A, ATP9B, ATRNL1, AVL9, B4GALT1, BAI3, BBS9, BEST3, BMPR1B, BRI3BP, BTBD11, BTN3A1, BTN3A2, BTNL2, C1GALT1, C7, C8A, CACNA1A, CACNA2D1, CACNA2D3, CADM2, CAMK2D, CASQ2, CASR, CATSPERB, CD151, CD207, CDH10, CDH13, CDH23, CDH4, CDH9, CDHR2, CDKAL1, CDON, CHL1, CHRM2, CHRNA9, CHRNB3, CHST11, CHST9, CLCA2, CLCNKB, CLDN1, CLDN10, CLDN14, CLDN20, CLEC12A, CLEC1A, CLEC1B, CLEC6A, CLECL1, CLIC5, CLSTN2, CMTM7, CMTM8, CNGA1, CNGA3, CNIH3, CNR2, CNTN1, CNTN4, CNTN5, CNTNAP2, CNTNAP4, CNTNAP5, COL13A1, COL25A1, COLEC12, CORIN, COX16, COX7B2, CPLX1, CSMD1, CSMD2, CSMD3, CUBN, CUX1, CXADR, CXCL12, CYBRD1, CYP2C8, CYP2E1, CYP4F11, CYP4F12, CYP4F2, CYP4F3, CYP4F8, CYP4Z1, DAB2, DAD1, DBH, DCC, DCHS2, DDOST, DDR1, DERL3, DIO1, DLC1, DMBT1, DNER, DPP6, DPY19L4, DRAM1, DSC1, DSCAM, DSG1, DYSF, ELFN2, ELOVL2, ELOVL6, ELTD1, EMCN, EMR1, EMR2, ENPP1, ENTPD1, EPB41L4A, EPHA7, EPHB1, ERAP1, ERAP2, ERBB4, ERO1LB, ERVFRD-1, ESYT2, EVC, EVC2, EXOC4, F2RL2, FAM155A, FAM163A, FAM173B, FAM189A1, FAM189A2, FARP1, FAS, FAT2, FAT3, FAT4, FCER2, FER, FER1L6, FGD5, FLT3, FLVCR1, FMO2, FMO5, FRAS1, FRMD3, FSHR, FUT9, FZD6, GABBR2, GABRB2, GABRG3, GABRR1, GALNT10, GALNT13, GALNT2, GALNTL4, GALNTL5, GALNTL6, GLDN, GLIPR1L2, GLRA3, GNB5, GOLM1, GPC5, GPC6, GPR111, GPR139, GPR158, GPR39, GPR65, GPR78, GRAMD1A, GRIA1, GRIA4, GRID2, GRIK2, GRIK3, GRIK4, GRIN2A, GRIN2B, GRIN3A, GRIP1, GRM4, GRM5, GRM7, GRM8, HHAT, HHLA2, HK1, HLA-A, HLA-B, HLA-C, HLA-DOA, HLA-DOB, HLA-DPA1, HLA-DPB1, HLA-DQA1, HLA-DQA2, HLA-DQB1, HLA-DQB2, HLA-DRA, HLA-DRB1, HLA-DRB5, HLA-F, HLA-G, HMGCLL1, HPSE, HS3ST4, HS6ST3, IFI27L1, IGSF5, IL12RB2, IL1RL1, IL1RL2, INADL, INSR, ITGA1, ITGA11, ITGA2, ITGA9, ITGB3, ITGB5, ITGBL1, ITPR1, ITPR2, IYD, JKAMP, KANK1, KCNA6, KCNAB1, KCNB2, KCNH7, KCNJ12, KCNJ16, KCNK10, KCNK2, KCNMB1, KCNMB2, KCNMB3, KCNQ3, KCNQ4, KCNQ5, KIAA1024, KIAA1324L, KIAA1549, KIRREL3, KL, KLHL14, KLRB1, LAMP3, LAPTM4B, LARGE, LDLRAD3, LEMD2, LEPR, LGR5, LHCGR, LHFP, LHFPL2, LHFPL3, LINGO2, LOC100507421, LPGAT1, LPPR5, LRFN2, LRFN5, LRP1B, LRRC52, LRRK2, LRRTM4, LSAMP, LYN, LYPD6B, MAN1A1, MAN2A1, MARCH4, MCOLN2, MDGA2, MEGF10, MEGF11, MEP1B, MGAT1, MGAT5B, MGST3, MOXD1, MRS2, MS4A6E, MTCH1, MUC12, MUC22, MYO10, MYO1D, MYOF, NAALADL2, NALCN, NAV3, NCALD, NCAM2, NDST3, NDST4, NDUFA12, NDUFB1, NDUFB10, NDUFB4, NEDD4, NETO1, NFASC, NINJ2, NIPAL1, NIPAL3, NKAIN1, NKAIN3, NMUR1, NMUR2, NOTCH2, NOTCH4, NPHS2, NPSR1, NRCAM, NRG1, NRG3, NRP1, NRXN1, NRXN3, NTM, NTRK2, NTSR1, NUP210, NUP35, NUP88, OCA2, ODZ3, ODZ4, OMA1, OPCML, OPN4, OPRK1, OR10AG1, OR10C1, OR12D2, OR13C2, OR13C5, OR14A16, OR1L8, OR1N2, OR1S1, OR2AK2, OR2B11, OR2J2, OR2L13, OR2T4, OR2W3, OR4C3, OR4C45, OR51B2, OR51B6, OR51D1, OR51E1, OR51F1, OR51I2, OR51M1, OR51Q1, OR51V1, OR52B6, OR52E2, OR52E4, OR52E6, OR52E8, OR52J3, OR52N2, OR52N4, OR52R1, OR5AC2, OR5F1, OR5I1, OR5P2, OR5W2, OR6N1, OR8G1, OR8G5, OR8H3, OR8I2, OR8U8, OR9G1, OR9G9, OTOP1, PCDH15, PCDH17, PCDHA1, PCDHA10, PCDHA11, PCDHA12, PCDHA13, PCDHA2, PCDHA3, PCDHA4, PCDHA5, PCDHA6, PCDHA7, PCDHA8, PCDHA9, PCDHAC1, PCNXL2, PCSK5, PCSK9, PDE3A, PDIA6, PEX5L, PGBD5, PI16, PIEZO1, PIEZO2, PIGG, PIGN, PIGP, PINK1, PKD1L1, PKD1L2, PKHD1, PKP2, PLA2G4A, PLA2G4C, PLA2R1, PLAUR, PLD1, PLD5, PLEK, PLEKHH2, PLOD2, PLSCR1, PLXDC2, PLXNA4, PLXNC1, PNPLA3, PNPLA6, PPYR1, PRICKLE2, PRKAB2, PRKCA, PROKR2, PRPH2, PTCHD3, PTGER3, PTPRB, PTPRD, PTPRF, PTPRG, PTPRM, PTPRQ, PTPRT, PTPRU, QRFPR, RAB27A, RAMP3, RAPGEF4, RASGRF2, RECK, RFTN1, RGS6, RGS7, RNF144B, RNF150, RNPEP, ROBO2, ROR1, ROR2, RYR1, RYR2, RYR3, SCARA5, SCARB1, SCD5, SCN10A, SCN1A, SCN3A, SCN3B, SCN9A, SCNN1G, SDC2, SDHA, SDK1, SEC11C, SEC24D, SEMA5A, SEMA6D, SERINC5, SGCG, SGCZ, SGIP1, SGMS1, SHISA2, SHISA6, SHISA9, SHROOM3, SIRPA, SLC10A2, SLC12A6, SLC12A8, SLC14A1, SLC14A2, SLC15A2, SLC15A5, SLC16A14, SLC17A5, SLC19A3, SLC1A2, SLC1A6, SLC22A16, SLC22A4, SLC22A5, SLC22A9, SLC24A4, SLC25A21, SLC25A25, SLC25A37, SLC25A38, SLC27A6, SLC28A1, SLC2A1, SLC2A14, SLC2A9, SLC30A9, SLC31A2, SLC35B3, SLC35D2, SLC35F3, SLC35F4, SLC37A1, SLC37A2, SLC38A4, SLC38A8, SLC38A9, SLC39A11, SLC39A14, SLC39A8, SLC41A3, SLC4A7, SLC5A12, SLC6A5, SLC7A7, SLC8A1, SLC8A3, SLC9A4, SLC9A8, SLC9A9, SLC9C1, SLCO1B1, SLCO1B3, SLCO1C1, SLCO5A1, SLCO6A1, SNTB1, SNTG2, SNX16, SNX5, SOAT1, SORBS1, SORCS1, SORCS2, SORCS3, SPATA13, SPCS3, SPINK5, SPNS3, SPPL2C, SPRED1, SPTB, SPTLC3, SSR1, ST6GALNAC3, ST8SIA1, ST8SIA6, STEAP1B, STK39, STON2, STT3A, STX2, STXBP6, STYK1, SV2B, SV2C, SYK, SYNE1, SYNJ2, SYNJ2BP, SYNPR, SYT6, SYT9, TACSTD2, TAP1, TAP2, TAS1R2, TAS2R19, TAS2R20, TAS2R30, TEK, TESC, TGFBR3, THSD7A, THSD7B, TKT, TM4SF4, TMC2, TMCC3, TMCO4, TMEM106B, TMEM108, TMEM117, TMEM128, TMEM132B, TMEM132C, TMEM132D, TMEM156, TMEM163, TMEM17, TMEM176A, TMEM176B, TMEM2, TMEM220, TMEM229B, TMEM244, TMEM44, TMEM51, TMEM63C, TMEM8A, TMPRSS15, TMPRSS9, TMTC1, TMTC2, TNFRSF11A, TNR, TOMM7, TPO, TRAM2, TRDN, TRPA1, TRPC4, TRPC6, TRPM3, TSHR, TSPAN15, TSPAN18, TSPAN2, TSPAN8, TSPAN9, UBR3, UGT1A10, UGT1A7, UGT1A8, UGT1A9, UGT2B7, UGT3A2, UNC5C, UNC93A, USH2A, UST, VNN1, VOPP1, VSTM4, VWF, WBSCR17, WDR11, WLS, WWC1, XKR6, XXYLT1, XYLT1, XYLT2, YIPF1, ZDHHC13, ZDHHC14, ZDHHC7*
Coated vesicle membrane	4.3×10^{-12}	63.9	*HLA-A, HLA-B, HLA-C, HLA-DPA1, HLA-DPB1, HLA-DQA1, HLA-DQB1, HLA-DRB1*
Trans-Golgi network membrane	2.2×10^{-11}	26.0	*HLA-DPA1, HLA-DPB1, HLA-DQA1, HLA-DQA2, HLA-DQB1, HLA-DQB2, HLA-DRA, HLA-DRB1, HLA-DRB5, KIF13A*

GO categories in which false discovery rate is less than 0.01.

Table S24 continued.

Description	p-value	Enrichment	Genes
Integral to membrane	2.9×10^{-11}	1.3	*AADAC*, *AADACL2*, *ABCA1*, *ABCA13*, *ABCA4*, *ABCB5*, *ABCC11*, *ABCC4*, *ABCG8*, *ABO*, *ACER1*, *ACPP*, *ADAM12*, *ADAM28*, *ADAM29*, *ADCY3*, *ADCY5*, *ADCY8*, *ADRA1A*, *AIFM2*, *AJAP1*, *ALK*, *ALPL*, *ANK2*, *APP*, *AQP8*, *AQP9*, *AQPEP*, *ART3*, *ASAH2*, *ASGR2*, *ASIC2*, *ASTN2*, *ATP10B*, *ATP10D*, *ATP1A4*, *ATP2C2*, *ATP6V0E2*, *ATP8A1*, *ATP8A2*, *ATP8B1*, *ATP9A*, *ATP9B*, *ATRNL1*, *AVL9*, *B4GALT1*, *BAI3*, *BMPR1B*, *BRI3BP*, *BTBD11*, *BTN3A1*, *BTN3A2*, *BTNL2*, *C1GALT1*, *CACNA1A*, *CACNA2D3*, *CADM2*, *CASR*, *CD151*, *CD207*, *CDH10*, *CDH23*, *CDH4*, *CDH9*, *CDHR2*, *CDKAL1*, *CDON*, *CHL1*, *CHRM2*, *CHRNB3*, *CHST11*, *CHST9*, *CLCA2*, *CLCNKB*, *CLDN1*, *CLDN10*, *CLDN14*, *CLDN20*, *CLEC12A*, *CLEC1A*, *CLEC1B*, *CLEC6A*, *CLECL1*, *CLSTN2*, *CMTM7*, *CMTM8*, *CNGA1*, *CNGA3*, *CNR2*, *CNTNAP2*, *CNTNAP4*, *CNTNAP5*, *COL13A1*, *COL25A1*, *COLEC12*, *CORIN*, *COX16*, *COX7B2*, *CSMD1*, *CSMD2*, *CSMD3*, *CUX1*, *CXADR*, *CYBRD1*, *CYP4F11*, *CYP4F12*, *CYP4F3*, *CYP4F8*, *CYP4Z1*, *DAD1*, *DBH*, *DCC*, *DCHS2*, *DDOST*, *DDR1*, *DERL3*, *DIO1*, *DNER*, *DPP6*, *DPY19L4*, *DRAM1*, *DSC1*, *DSCAM*, *DSG1*, *DYSF*, *ELFN2*, *ELOVL2*, *ELOVL6*, *ELTD1*, *EMCN*, *EMR1*, *EMR2*, *ENPP1*, *ENTPD1*, *EPHA7*, *EPHB1*, *ERAP1*, *ERAP2*, *ERBB4*, *ERVFRD-1*, *ESYT2*, *EVC*, *EVC2*, *F2RL2*, *FAM155A*, *FAM163A*, *FAM173B*, *FAM189A1*, *FAM189A2*, *FAS*, *FAT2*, *FAT3*, *FAT4*, *FCER2*, *FER1L6*, *FLT3*, *FLVCR1*, *FMO2*, *FMO5*, *FRAS1*, *FRMD3*, *FSHR*, *FUT9*, *FZD6*, *GABBR2*, *GABRB2*, *GABRG3*, *GABRR1*, *GALNT10*, *GALNT13*, *GALNT2*, *GALNTL4*, *GALNTL5*, *GALNTL6*, *GLDN*, *GLIPR1L2*, *GLRA3*, *GOLM1*, *GPC5*, *GPC6*, *GPR111*, *GPR139*, *GPR158*, *GPR39*, *GPR65*, *GPR78*, *GRAMD1A*, *GRID2*, *GRIK2*, *GRIK3*, *GRIK4*, *GRIN2A*, *GRIN2B*, *GRIN3A*, *GRM4*, *GRM5*, *GRM7*, *GRM8*, *HHAT*, *HHLA2*, *HLA-A*, *HLA-B*, *HLA-C*, *HLA-DOA*, *HLA-DOB*, *HLA-DPA1*, *HLA-DPB1*, *HLA-DQA1*, *HLA-DQA2*, *HLA-DQB1*, *HLA-DQB2*, *HLA-DRA*, *HLA-DRB1*, *HLA-DRB5*, *HLA-F*, *HLA-G*, *HS3ST4*, *HS6ST3*, *IFI27L1*, *IGSF5*, *IL12RB2*, *IL1RL1*, *IL1RL2*, *INSR*, *ITGB3*, *ITPR1*, *ITPR2*, *IYD*, *JKAMP*, *KCNAB1*, *KCNH7*, *KCNJ12*, *KCNK10*, *KCNMB2*, *KCNMB3*, *KIAA1024*, *KIAA1324L*, *KIAA1549*, *KIRREL3*, *KL*, *KLRB1*, *LAMP3*, *LAPTM4B*, *LARGE*, *LDLRAD3*, *LEMD2*, *LEPR*, *LGR5*, *LHCGR*, *LHFP*, *LHFPL2*, *LHFPL3*, *LINGO2*, *LOC100507421*, *LPGAT1*, *LPPR5*, *LRFN2*, *LRFN5*, *LRP1B*, *LRRC52*, *LRRTM4*, *MAN1A1*, *MAN2A1*, *MARCH4*, *MCOLN2*, *MEGF10*, *MEGF11*, *MEP1B*, *MGAT1*, *MGAT5B*, *MGST3*, *MOXD1*, *MRS2*, *MS4A6E*, *MTCH1*, *MUC12*, *MUC22*, *MYOF*, *NAALADL2*, *NALCN*, *NCAM2*, *NDST3*, *NDST4*, *NDUFB1*, *NDUFB4*, *NETO1*, *NFASC*, *NINJ2*, *NIPAL1*, *NIPAL3*, *NKAIN1*, *NKAIN3*, *NMUR1*, *NMUR2*, *NOTCH2*, *NOTCH4*, *NPHS2*, *NPSR1*, *NRCAM*, *NRG1*, *NRG3*, *NRP1*, *NRXN1*, *NRXN3*, *NTRK2*, *NTSR1*, *OCA2*, *ODZ3*, *ODZ4*, *OMA1*, *OPCML*, *OPN4*, *OPRK1*, *OR10AG1*, *OR10C1*, *OR12D2*, *OR13C2*, *OR13C5*, *OR14A16*, *OR1L8*, *OR1N2*, *OR1S1*, *OR2AK2*, *OR2B11*, *OR2J2*, *OR2L13*, *OR2T4*, *OR2W3*, *OR4C3*, *OR4C45*, *OR51B2*, *OR51B6*, *OR51D1*, *OR51E1*, *OR51F1*, *OR51I2*, *OR51M1*, *OR51Q1*, *OR51V1*, *OR52B6*, *OR52E2*, *OR52E4*, *OR52E6*, *OR52E8*, *OR52J3*, *OR52N2*, *OR52N4*, *OR52R1*, *OR5AC2*, *OR5F1*, *OR5I1*, *OR5P2*, *OR5W2*, *OR6N1*, *OR8G1*, *OR8G5*, *OR8H3*, *OR8I2*, *OR8U8*, *OR9G1*, *OR9G9*, *OTOP1*, *PCDH15*, *PCDH17*, *PCDHA1*, *PCDHA10*, *PCDHA11*, *PCDHA12*, *PCDHA13*, *PCDHA2*, *PCDHA3*, *PCDHA4*, *PCDHA5*, *PCDHA6*, *PCDHA7*, *PCDHA8*, *PCDHA9*, *PCDHAC1*, *PCNXL2*, *PCSK5*, *PDE3A*, *PGBD5*, *PI16*, *PIEZO1*, *PIEZO2*, *PIGG*, *PIGN*, *PIGP*, *PINK1*, *PKD1L1*, *PKD1L2*, *PKHD1*, *PKP2*, *PLA2R1*, *PLAUR*, *PLD5*, *PLEKHH2*, *PLSCR1*, *PLXDC2*, *PLXNA4*, *PLXNC1*, *PNPLA3*, *PNPLA6*, *PPYR1*, *PROKR2*, *PRPH2*, *PTCHD3*, *PTGER3*, *PTPRB*, *PTPRD*, *PTPRF*, *PTPRG*, *PTPRM*, *PTPRQ*, *PTPRT*, *PTPRU*, *QRFPR*, *RAMP3*, *RNF144B*, *RNF150*, *ROBO2*, *ROR1*, *ROR2*, *RYR1*, *RYR3*, *SCARA5*, *SCARB1*, *SCD5*, *SCN1A*, *SCN3B*, *SCNN1G*, *SDC2*, *SDK1*, *SEC11C*, *SEMA5A*, *SEMA6D*, *SERINC5*, *SGCG*, *SGCZ*, *SGMS1*, *SHISA2*, *SHISA6*, *SIRPA*, *SLC10A2*, *SLC12A6*, *SLC12A8*, *SLC14A1*, *SLC14A2*, *SLC15A2*, *SLC15A5*, *SLC16A14*, *SLC17A5*, *SLC19A3*, *SLC1A2*, *SLC1A6*, *SLC22A16*, *SLC22A4*, *SLC22A5*, *SLC22A9*, *SLC24A4*, *SLC25A21*, *SLC25A25*, *SLC25A37*, *SLC25A38*, *SLC27A6*, *SLC28A1*, *SLC2A1*, *SLC2A14*, *SLC2A9*, *SLC30A9*, *SLC31A2*, *SLC35B3*, *SLC35D2*, *SLC35F3*, *SLC35F4*, *SLC37A1*, *SLC37A2*, *SLC38A4*, *SLC38A8*, *SLC38A9*, *SLC39A11*, *SLC39A14*, *SLC39A8*, *SLC41A3*, *SLC4A7*, *SLC5A12*, *SLC6A5*, *SLC7A7*, *SLC8A1*, *SLC8A3*, *SLC9A4*, *SLC9A8*, *SLC9A9*, *SLC9C1*, *SLCO1B1*, *SLCO1B3*, *SLCO1C1*, *SLCO5A1*, *SLCO6A1*, *SOAT1*, *SORCS1*, *SORCS2*, *SORCS3*, *SPCS3*, *SPNS3*, *SPPL2C*, *SPTLC3*, *SSR1*, *ST6GALNAC3*, *ST8SIA1*, *ST8SIA6*, *STEAP1B*, *STT3A*, *STX2*, *STXBP6*, *STYK1*, *SV2B*, *SV2C*, *SYNE1*, *SYNJ2BP*, *SYNPR*, *SYT6*, *SYT9*, *TACSTD2*, *TAP1*, *TAP2*, *TAS1R2*, *TAS2R19*, *TAS2R20*, *TAS2R30*, *TEK*, *TGFBR3*, *THSD7A*, *THSD7B*, *TM4SF4*, *TMC2*, *TMCC3*, *TMCO4*, *TMEM106B*, *TMEM108*, *TMEM117*, *TMEM128*, *TMEM132B*, *TMEM132C*, *TMEM132D*, *TMEM156*, *TMEM163*, *TMEM17*, *TMEM176A*, *TMEM176B*, *TMEM2*, *TMEM220*, *TMEM229B*, *TMEM244*, *TMEM44*, *TMEM51*, *TMEM63C*, *TMEM8A*, *TMPRSS15*, *TMPRSS9*, *TMTC1*, *TMTC2*, *TNFRSF11A*, *TOMM7*, *TPO*, *TRAM2*, *TRDN*, *TRPA1*, *TRPC4*, *TRPC6*, *TRPM3*, *TSHR*, *TSPAN15*, *TSPAN18*, *TSPAN2*, *TSPAN8*, *TSPAN9*, *UBR3*, *UGT1A10*, *UGT1A7*, *UGT1A8*, *UGT1A9*, *UGT2B7*, *UGT3A2*, *UNC5C*, *UNC93A*, *USH2A*, *UST*, *VNN1*, *VOPP1*, *VSTM4*, *WBSCR17*, *WDR11*, *WLS*, *XKR6*, *XXYLT1*, *XYLT1*, *XYLT2*, *YIPF1*, *ZDHHC13*, *ZDHHC14*, *ZDHHC7*
Cytoplasmic vesicle part	3.0×10^{-11}	22.9	*CPE*, *DMBT1*, *HLA-A*, *HLA-B*, *HLA-C*, *HLA-DPA1*, *HLA-DPB1*, *HLA-DQA1*, *HLA-DQB1*, *HLA-DRB1*

GO categories in which false discovery rate is less than 0.01.

Table S24 continued.

Description	p-value	Enrichment	Genes
Membrane	3.3×10^{-11}	1.3	*AADAC, ABCA1, ABCA4, ABCB5, ABCC4, ABCG8, ABO, ACPP, ACTN1, ADAM12, ADAM28, ADCY3, ADCY5, ADCY8, ADD2, ADRA1A, AIFM2, AKAP12, AKAP13, AKAP6, ALDH18A1, ALPL, ALS2, AMPH, ANGPT1, ANGPT2, ANK2, ANK3, ANKS1B, ANO2, AP1S3, AP3B1, APBB1IP, APBB2, AQP8, ARHGAP24, ARHGEF18, ASAH2, ASAP1, ATP10D, ATP12A, ATP1A4, ATP2C2, ATP5C1, ATP6V0E2, ATP8A1, ATP8B1, ATP9A, AVEN, B4GALT1, BAI3, BBS9, BCL2L14, BCR, BEST3, BICD1, BLNK, BMPR1B, BNC2, BSPRY, BTN3A1, BTN3A2, C1GALT1, C3, C8A, CABS1, CACNA1A, CADM2, CADPS, CADPS2, CAMK2D, CAP1, CASQ2, CBFA2T3, CCDC88A, CCDC91, CD207, CDH10, CDH13, CDH23, CDH4, CDH9, CDKAL1, CHD1L, CHL1, CHRM2, CHRNA9, CHRNB3, CHST11, CHST9, CLDN10, CLDN14, CLEC12A, CLECL1, CLSTN2, CNGA3, CNIH3, CNR2, CNTN1, CNTN4, CNTN5, CNTNAP2, COG6, COL13A1, CORIN, CORO2B, CPE, CSMD2, CSMD3, CUBN, CUX1, CXADR, CYB5R2, CYBRD1, CYP2C8, CYP2E1, CYP4F11, CYP4F12, CYP4F2, CYP4F3, CYP4F8, CYP4Z1, DAAM1, DAB2, DAD1, DAPP1, DCC, DCHS2, DGKB, DGKH, DIO1, DISC1, DLG2, DLGAP1, DMBT1, DNAAF1, DNER, DNM1L, DOCK1, DOCK4, DOPEY2, DRAM1, DSC1, DSCAM, DSG1, DTNA, DYM, DYNC2H1, DYSF, DYTN, ELMO1, ELOVL2, ELOVL6, ELTD1, EMID2, EMR2, ENDOU, ENPP1, EPHA7, EPHB1, ERAP1, ERAP2, ERBB4, ERC2, ERO1LB, ERVFRD-1, ESYT2, EXOC2, EXOC4, F2RL2, F5, FABP2, FAS, FAT2, FAT3, FAT4, FHIT, FLVCR1, FMN1, FMO2, FNDC1, FRAS1, FRMPD1, FSHR, FUT9, FZD6, GABBR2, GABRB2, GABRR1, GALNT10, GALNT13, GALNT2, GALNTL4, GALNTL5, GAS2, GLDN, GPC5, GPC6, GPR111, GPR139, GPR158, GPR39, GPR65, GPR78, GRB10, GRIA1, GRID2, GRIK2, GRIK3, GRIK4, GRIN2A, GRIN2B, GRIN3A, GRIP1, GRIP2, GRM5, GRM7, GRM8, GUCA1A, HAAO, HHAT, HIP1, HK1, HLA-A, HLA-B, HLA-C, HLA-DOA, HLA-DOB, HLA-DPA1, HLA-DPB1, HLA-DQA1, HLA-DQA2, HLA-DQB1, HLA-DQB2, HLA-DRA, HLA-DRB1, HLA-DRB5, HLA-F, HLA-G, HMGCLL1, HPSE2, HS3ST4, IGSF5, INADL, INPP5D, ITGA1, ITGA2, ITGA9, ITGB3, ITGB5, ITPR2, IYD, JKAMP, KAZN, KCNA6, KCNAB1, KCNB2, KCNH7, KCNIP1, KCNIP4, KCNJ12, KCNJ16, KCNK10, KCNK2, KCNMB1, KCNMB2, KCNMB3, KCNQ3, KCNQ4, KCNQ5, KIF13A, KIRREL3, KL, KLHL14, KLRB1, KNG1, KSR2, LAMA2, LDLRAD3, LHCGR, LOXL2, LRRC52, LRRC7, LRRK2, LRRTM4, LYPD6B, MAGI1, MAGI2, MAN1A1, MAN2A1, MAPT, MARCH4, MBP, MCC, MDGA2, MGAT1, MGAT5B, MGST3, MLXIP, MRS2, MRTO4, MTCH1, MTHFS, MTUS1, MYO10, MYO16, MYO1B, MYO5B, MYOF, NAV3, NCAM2, NCK2, NDST3, NDST4, NDUFA12, NDUFB1, NDUFB10, NDUFB4, NEDD4, NETO1, NFASC, NGEF, NKAIN3, NMUR1, NMUR2, NOTCH4, NPHS2, NPSR1, NRCAM, NRG1, NRP1, NRXN1, NSL1, NTM, NTN4, NUP210, NUP35, OCA2, ODZ3, OPCML, OPRK1, OR10AG1, OR10C1, OR12D2, OR13C2, OR13C5, OR14A16, OR1L8, OR1N2, OR1S1, OR2AK2, OR2B11, OR2J2, OR2L13, OR2T4, OR2W3, OR4C3, OR4C45, OR51B2, OR51B6, OR51D1, OR51E1, OR51F1, OR51I2, OR51M1, OR51Q1, OR52B6, OR52E2, OR52E6, OR52E8, OR52J3, OR52N2, OR52N4, OR52R1, OR5AC2, OR5F1, OR5I1, OR5P2, OR5W2, OR8G1, OR8G5, OR8H3, OR8I2, OR8U8, OR9G1, OR9G9, PALM2, PARD3B, PCDH15, PCDH17, PCDHA12, PCDHA13, PCDHA9, PCLO, PCM1, PDE4D, PDGFD, PDIA6, PEX5L, PGLYRP4, PHLDB2, PIGG, PIGN, PIGP, PINK1, PKD1L1, PKP1, PKP2, PLA2G4A, PLA2G4C, PLA2G4E, PLA2G6, PLA2R1, PLAUR, PLCB1, PLCG2, PLCH1, PLD1, PLOD2, PLSCR1, PLXNA4, PNPLA3, PNPLA6, PPFIBP1, PPP1R14C, PPYR1, PRICKLE1, PRICKLE2, PRKCA, PRKCE, PRKG1, PROKR2, PSD3, PSTPIP2, PTGER3, PTPN13, PXK, QRFPR, RAB27A, RAB31, RAB3C, RAC2, RAMP3, RAPGEF4, RASGRF1, RFTN1, RGS6, RGS7, RIMBP2, RIMS1, RIMS2, RNF144B, RNPEP, ROR1, RPSA, RYR2, RYR3, SBF2, SCARB1, SCD5, SDHA, SEC24D, SEMA3A, SEMA3E, SEMA5A, SEMA6D, SERINC5, SGCG, SGCZ, SGIP1, SGMS1, SH3GL2, SIRPA, SKAP2, SLC10A2, SLC12A6, SLC14A2, SLC15A2, SLC16A14, SLC17A5, SLC19A3, SLC1A2, SLC1A6, SLC22A16, SLC22A5, SLC24A4, SLC25A21, SLC25A25, SLC25A37, SLC25A38, SLC27A6, SLC28A1, SLC2A1, SLC2A9, SLC35B3, SLC35D2, SLC39A14, SLC39A8, SLC6A5, SLC7A7, SLC9A4, SLC9A8, SLC9A9, SLC9C1, SLCO1B1, SLCO1B3, SLCO5A1, SLCO6A1, SNCA, SNTB1, SNTG2, SNX16, SNX19, SNX24, SNX7, SOAT1, SORBS1, SORCS1, SORCS2, SORCS3, SPCS3, SPINK5, SPPL2C, SPRED1, SPTLC3, SQRDL, SSC5D, SSR1, ST8SIA6, STARD13, STT3A, STYK1, SV2C, SVEP1, SVIL, SWAP70, SYK, SYN3, SYNE1, SYNPR, SYT6, SYT9, TANC1, TAP1, TAP2, TAS1R2, TEC, TEK, TES, TESC, THSD7A, TIAM1, TJP2, TKT, TMEM106B, TMEM163, TMEM176B, TPO, TRDN, TRPC4, TRPC6, TSHR, TSPAN15, TSPAN9, TULP3, UGT1A10, UGT1A7, UGT1A8, UGT1A9, UGT2B7, UNC13C, UNC5C, UNC93A, UST, VAV3, VNN1, VOPP1, VPS37C, WBSCR17, WLS, WWOX, XXYLT1, XYLT1, XYLT2, ZDHHC13, ZFYVE28*
Clathrin-coated endocytic vesicle membrane	3.3×10^{-11}	74.8	*HLA-DPA1, HLA-DPB1, HLA-DQA1, HLA-DQB1, HLA-DRA, HLA-DRB1, HLA-DRB5*

GO categories in which false discovery rate is less than 0.01.

Table S24 continued.

Description	p-value	Enrichment	Genes
Intrinsic to membrane	5.7×10^{-11}	1.3	*AADAC, AADACL2, ABCA1, ABCA13, ABCA4, ABCB5, ABCC11, ABCC4, ABCG8, ABO, ACER1, ACPP, ADAM12, ADAM28, ADAM29, ADCY3, ADCY5, ADCY8, ADRA1A, AIFM2, AJAP1, ALK, ALPL, ANK2, APP, AQP8, AQP9, AQPEP, ART3, ASAH2, ASGR2, ASIC2, ASTN2, ATP10B, ATP10D, ATP1A4, ATP2C2, ATP6V0E2, ATP8A1, ATP8A2, ATP8B1, ATP9A, ATP9B, ATRNL1, AVL9, B4GALT1, BAI3, BMPR1B, BRI3BP, BTBD11, BTN3A1, BTN3A2, BTNL2, C1GALT1, CACNA1A, CACNA2D3, CADM2, CASR, CD151, CD207, CDH10, CDH13, CDH23, CDH4, CDH9, CDHR2, CDKAL1, CDON, CHL1, CHRM2, CHRNB3, CHST11, CHST9, CLCA2, CLCNKB, CLDN1, CLDN10, CLDN14, CLDN20, CLEC12A, CLEC1A, CLEC1B, CLEC6A, CLECL1, CLSTN2, CMTM7, CMTM8, CNGA1, CNGA3, CNR2, CNTN1, CNTN4, CNTN5, CNTNAP2, CNTNAP4, CNTNAP5, COL13A1, COL25A1, COLEC12, CORIN, COX16, COX7B2, CSMD1, CSMD2, CSMD3, CUX1, CXADR, CY-BRD1, CYP2E1, CYP4F11, CYP4F12, CYP4F3, CYP4F8, CYP4Z1, DAD1, DBH, DCC, DCHS2, DDOST, DDR1, DERL3, DIO1, DNER, DPP6, DPY19L4, DRAM1, DSC1, DSCAM, DSG1, DYSF, ELFN2, ELOVL2, ELOVL6, ELTD1, EMCN, EMR1, EMR2, ENPP1, ENTPD1, EPHA7, EPHB1, ERAP1, ERAP2, ERBB4, ERVFRD-1, ESYT2, EVC, EVC2, F2RL2, FAM155A, FAM163A, FAM173B, FAM189A1, FAM189A2, FAS, FAT2, FAT3, FAT4, FCER2, FER1L6, FLT3, FLVCR1, FMO2, FMO5, FRAS1, FRMD3, FSHR, FUT9, FZD6, GABBR2, GABRB2, GABRG3, GABRR1, GALNT10, GALNT13, GALNT2, GALNTL4, GALNTL5, GAL-NTL6, GLDN, GLIPR1L2, GLRA3, GOLM1, GPC5, GPC6, GPR111, GPR139, GPR158, GPR39, GPR65, GPR78, GRAMD1A, GRID2, GRIK2, GRIK3, GRIK4, GRIN2A, GRIN2B, GRIN3A, GRM4, GRM5, GRM7, GRM8, HHAT, HHLA2, HLA-A, HLA-B, HLA-C, HLA-DOA, HLA-DOB, HLA-DPA1, HLA-DPB1, HLA-DQA1, HLA-DQA2, HLA-DQB1, HLA-DQB2, HLA-DRA, HLA-DRB1, HLA-DRB5, HLA-F, HLA-G, HS3ST4, HS6ST3, IFI27L1, IGSF5, IL12RB2, IL1RL1, IL1RL2, INSR, ITGB3, ITPR1, ITPR2, IYD, JKAMP, KCNAB1, KCNH7, KCNJ12, KCNK10, KCNMB2, KCNMB3, KIAA1024, KIAA1324L, KIAA1549, KIRREL3, KL, KLRB1, LAMP3, LAPTM4B, LARGE, LDLRAD3, LEMD2, LEPR, LGR5, LHCGR, LHFP, LHFPL2, LHFPL3, LINGO2, LOC100507421, LPGAT1, LPPR5, LRFN2, LRFN5, LRP1B, LRRC52, LRRTM4, LSAMP, LYPD6B, MAN1A1, MAN2A1, MARCH4, MCOLN2, MDGA2, MEGF10, MEGF11, MEP1B, MGAT1, MGAT5B, MGST3, MOXD1, MRS2, MS4A6E, MTCH1, MUC12, MUC22, MYOF, NAALADL2, NALCN, NCAM2, NDST3, NDST4, NDUFB1, NDUFB4, NETO1, NFASC, NINJ2, NIPAL1, NIPAL3, NKAIN1, NKAIN3, NMUR1, NMUR2, NOTCH2, NOTCH4, NPHS2, NPSR1, NRCAM, NRG1, NRG3, NRP1, NRXN1, NRXN3, NTM, NTRK2, NTSR1, OCA2, ODZ3, ODZ4, OMA1, OPCML, OPN4, OPRK1, OR10AG1, OR10C1, OR12D2, OR13C2, OR13C5, OR14A16, OR1L8, OR1N2, OR1S1, OR2AK2, OR2B11, OR2J2, OR2L13, OR2T4, OR2W3, OR4C3, OR4C45, OR51B2, OR51B6, OR51D1, OR51E1, OR51F1, OR51I2, OR51M1, OR51Q1, OR51V1, OR52B6, OR52E2, OR52E4, OR52E6, OR52E8, OR52J3, OR52N2, OR52N4, OR52R1, OR5AC2, OR5F1, OR5I1, OR5P2, OR5W2, OR6N1, OR8G1, OR8G5, OR8H3, OR8I2, OR8U8, OR9G1, OR9G9, OTOP1, PCDH15, PCDH17, PCDHA1, PCDHA10, PCDHA11, PCDHA12, PCDHA13, PCDHA2, PCDHA3, PCDHA4, PCDHA5, PCDHA6, PCDHA7, PCDHA8, PCDHA9, PCDHAC1, PCNXL2, PCSK5, PDE3A, PGBD5, PI16, PIEZO1, PIEZO2, PIGG, PIGN, PIGP, PINK1, PKD1L1, PKD1L2, PKHD1, PKP2, PLA2R1, PLAUR, PLD5, PLEKHH2, PLSCR1, PLXDC2, PLXNA4, PLXNC1, PN-PLA3, PNPLA6, PPYR1, PROKR2, PRPH2, PTCHD3, PTGER3, PTPRB, PTPRD, PTPRF, PTPRG, PTPRM, PTPRQ, PTPRT, PTPRU, QRFPR, RAMP3, RECK, RNF144B, RNF150, ROBO2, ROR1, ROR2, RYR1, RYR3, SCARA5, SCARB1, SCD5, SCN1A, SCN3B, SCNN1G, SDC2, SDK1, SEC11C, SEMA5A, SEMA6D, SERINC5, SGCG, SGCZ, SGMS1, SHISA2, SHISA6, SIRPA, SLC10A2, SLC12A6, SLC12A8, SLC14A1, SLC14A2, SLC15A2, SLC15A5, SLC16A14, SLC17A5, SLC19A3, SLC1A2, SLC1A6, SLC22A16, SLC22A4, SLC22A5, SLC22A9, SLC24A4, SLC25A21, SLC25A25, SLC25A37, SLC25A38, SLC27A6, SLC28A1, SLC2A1, SLC2A14, SLC2A9, SLC30A9, SLC31A2, SLC35B3, SLC35D2, SLC35F3, SLC35F4, SLC37A1, SLC37A2, SLC38A4, SLC38A8, SLC38A9, SLC39A11, SLC39A14, SLC39A8, SLC41A3, SLC4A7, SLC5A12, SLC6A5, SLC7A7, SLC8A1, SLC8A3, SLC9A4, SLC9A8, SLC9A9, SLC9C1, SLCO1B1, SLCO1B3, SLCO1C1, SLCO5A1, SLCO6A1, SOAT1, SORCS1, SORCS2, SORCS3, SPCS3, SPNS3, SPPL2C, SPTB, SPTLC3, SSR1, ST6GALNAC3, ST8SIA1, ST8SIA6, STEAP1B, STT3A, STX2, STXBP6, STYK1, SV2B, SV2C, SYNE1, SYNJ2BP, SYNPR, SYT6, SYT9, TACSTD2, TAP1, TAP2, TAS1R2, TAS2R19, TAS2R20, TAS2R30, TEK, TGFBR3, THSD7A, THSD7B, TM4SF4, TMC2, TMCC3, TMCO4, TMEM106B, TMEM108, TMEM117, TMEM128, TMEM132B, TMEM132C, TMEM132D, TMEM156, TMEM163, TMEM17, TMEM176A, TMEM176B, TMEM2, TMEM220, TMEM229B, TMEM244, TMEM44, TMEM51, TMEM63C, TMEM8A, TMPRSS15, TMPRSS9, TMTC1, TMTC2, TNFRSF11A, TOMM7, TPO, TRAM2, TRDN, TRPA1, TRPC4, TRPC6, TRPM3, TSHR, TSPAN15, TSPAN18, TSPAN2, TSPAN8, TSPAN9, UBR3, UGT1A10, UGT1A7, UGT1A8, UGT1A9, UGT2B7, UGT3A2, UNC5C, UNC93A, USH2A, UST, VNN1, VOPP1, VSTM4, WBSCR17, WDR11, WLS, XKR6, XXYLT1, XYLT1, XYLT2, YIPF1, ZDHHC13, ZDHHC14, ZDHHC7*
Integral to organelle membrane	7.9×10^{-11}	38.8	*HLA-A, HLA-B, HLA-C, HLA-DPA1, HLA-DPB1, HLA-DQA1, HLA-DQB1, HLA-DRB1*
Intrinsic to organelle membrane	2.3×10^{-10}	34.7	*HLA-A, HLA-B, HLA-C, HLA-DPA1, HLA-DPB1, HLA-DQA1, HLA-DQB1, HLA-DRB1*
Cell junction	5.6×10^{-10}	1.8	*ABCB5, ABI1, ACTN1, ACTN2, AFAP1, AJAP1, AMPH, AMTN, ANK2, ANK3, ANKS1B, APBB1IP, ARHGAP24, ARHGEF18, B4GALT1, BSPRY, CADM2, CADPS, CADPS2, CCDC85C, CDC42BPA, CDHR2, CDSN, CGNL1, CHRM2, CHRNA9, CHRNB3, CLCA2, CLDN1, CLDN10, CLDN14, CLDN20, CNIH3, COL13A1, CTNNA3, CXADR, DISC1, DLC1, DLG2, DLGAP1, DSC1, DSG1, DTNA, EGFLAM, ERC2, FAT2, FER, FMN1, FNDC1, FRMD4A, GABBR2, GABRB2, GABRG3, GABRR1, GLRA3, GRIA1, GRIA4, GRID2, GRIK2, GRIK3, GRIK4, GRIN2A, GRIN2B, GRIN3A, GRIP1, IGSF5, INADL, IQSEC1, ITGB5, KAZN, KIFC3, LAMA1, LPP, LRFN2, LRRC7, LRRTM4, MAGI1, MAGI2, NCS1, NETO1, NFASC, NFIA, NPHS2, NRXN1, PALLD, PARD3, PARD3B, PARVB, PCLO, PDZD2, PHACTR1, PKP1, PKP2, PLEKHA7, POLR1E, PSD3, PTPRM, PTPRU, RIMBP2, RIMS1, RIMS2, SCN1A, SEPT11, SHISA9, SHROOM3, SLC2A1, SLC8A1, SNCA, SNTB1, SORBS1, STX2, SV2B, SV2C, SVIL, SYN3, SYNPR, SYT6, SYT9, TANC1, TEK, TES, TIAM1, TJP2, TMEM163, TNS1, TRIM9, TRPC4, TRPC6, UNC13A, UNC13C*

GO categories in which false discovery rate is less than 0.01.

Table S24 continued.

Description	p-value	Enrichment	Genes
Plasma membrane part	2.5×10^{-9}	1.7	ABCA1, ABCA4, ABCB5, ABCG8, ADCY3, ADRA1A, ALK, ANK2, ANK3, ART3, ASIC2, BMPR1B, CACNA1A, CASQ2, CATSPERB, CDH13, CDH4, CHRNB3, CLCNKB, CLEC1A, CLEC1B, CNR2, CNTNAP2, CUBN, CYBRD1, CYP4F12, DAB2, DCC, DLC1, DSCAM, DSG1, DYSF, EMR1, ERBB4, EVC, EVC2, FAS, FCER2, FGD5, FLVCR1, GABRR1, GPC5, GPC6, GRIA1, GRIK2, GRIK3, GRIN2A, GRM5, HLA-A, HLA-B, HLA-C, HLA-DOA, HLA-DPA1, HLA-DPB1, HLA-DQA1, HLA-DQA2, HLA-DQB1, HLA-DQB2, HLA-DRA, HLA-DRB1, HLA-DRB5, HLA-F, HLA-G, IL1RL2, INADL, ITGA1, ITGBL1, KANK1, KCNK2, KCNMB3, KCNQ5, KL, LHCGR, MEGF11, MUC12, MYO10, MYOF, NEDD4, NOTCH4, NRCAM, NRG1, NRG3, NRXN1, NRXN3, OPCML, OPRK1, PCDHA1, PCDHA10, PCDHA11, PCDHA2, PCDHA3, PCDHA4, PCDHA5, PCDHA6, PCDHA7, PCDHA8, PCDHAC1, PCSK9, PKHD1, PLEK, PLSCR1, PPYR1, PRKAB2, PTGER3, PTPRB, PTPRD, PTPRM, RAMP3, RAPGEF4, RGS6, ROBO2, ROR1, SCARB1, SCN1A, SCNN1G, SGCG, SGCZ, SLC12A6, SLC15A2, SLC17A5, SLC1A2, SLC2A9, SLC5A12, SLC9C1, SLCO1B1, SLCO1B3, SNTB1, SNTG2, SPATA13, SPRED1, SPTB, STK39, SYK, TEK, TMEM17, TNFRSF11A, TPO, TRPC4, TSHR, TSPAN9, VWF
Cell projection part	3.2×10^{-9}	1.9	ABI1, ACTN2, ALS2, AMPH, ANK3, ANKS1B, APBB2, ATP8B1, BBS9, CDH23, CHRM2, CNIH3, CNTNAP2, CUBN, CXADR, CYBRD1, DCC, DISC1, DLG2, DLGAP1, DNAAF1, DNAH11, DNAH12, DNAH17, DNAH3, DNAH5, DNAH8, DSCAM, DYNC2H1, EMR2, ERBB4, ERC2, EVC, EVC2, EXOC4, FGD5, FOPNL, FSCB, FZD6, GRIA1, GRID2, GRIK2, GRIK3, GRIK4, GRIN2A, GRIN2B, GRIN3A, GRM5, GRM7, IFT57, KANK1, KCNQ3, KIRREL3, LRRC7, LRRK2, MAGI2, MAP2, MAPT, MBP, MYO10, MYO5A, MYO5B, NETO1, NFASC, NGEF, NRCAM, NRP1, NRXN1, ODF1, PACRG, PKD1L1, PKHD1, PLCB4, PLEK, PSD3, RAPGEF4, ROBO2, SCARB1, SCN1A, SLC1A2, SLC22A5, SLC9C1, SNCA, SPAG16, SPATA13, SYNJ2, TANC1, TEKT4, TESC, TIAM2, TMEM17, TTLL11, USH2A, WWC1
Endosome membrane	4.2×10^{-9}	24.1	HLA-A, HLA-B, HLA-C, HLA-DPA1, HLA-DPB1, HLA-DQA1, HLA-DQB1, HLA-DRB1
Endosomal part	5.9×10^{-9}	23.0	HLA-A, HLA-B, HLA-C, HLA-DPA1, HLA-DPB1, HLA-DQA1, HLA-DQB1, HLA-DRB1
Cell projection	1.7×10^{-8}	1.7	ABI1, ACPP, ACTN1, ACTN2, ACTR2, ADCY5, ALS2, ANGPT1, ANGPT2, APBB1IP, APBB2, APOA1BP, APP, ARHGAP24, ARPC5, BMPR1B, CABS1, CACNA1A, CADM2, CATSPERB, CCDC40, CCDC88A, CDH13, CDH23, CHL1, CHRM2, CLIC5, CNGA3, CNR2, CNTN4, CNTNAP2, CPLX1, CTNNA3, CXADR, DBH, DCC, DDC, DNAH8, DNER, DNM3, DOCK4, DSCAM, DYNC2H1, EPHA7, EPHB1, EVC, EVC2, FABP2, FAM65B, FER, FGD4, FGD5, FOPNL, FRMD4B, FSCB, GABBR2, GAS7, GRIA1, GRID2, GRIK3, GRIK4, GRIN2B, GRIN3A, GRIP1, GRM7, GRM8, IFT57, IQCB1, IQGAP2, ITGA1, KALRN, KCNIP1, KCNIP4, KCNQ4, KIRREL3, KLHL1, KLHL14, KLHL24, LDB3, LDHC, LRRC16A, LRRK2, MAGI1, MAPT, MEGF10, MTSS1, MYO10, MYO1B, MYO3A, MYO5A, MYO5B, MYRIP, NCAM2, NCS1, NOV, NRCAM, NTM, ODZ4, PACRG, PALLD, PCDH15, PEX5L, PKHD1, PLD1, PRKCA, PTPN13, PTPRM, RAB27A, RAPGEF4, RASGRF1, RELN, RPTOR, SCN10A, SEMA3A, SEPT11, SLC1A2, SLC39A14, SLC8A1, SLC9C1, SNCA, SPATA13, SVIL, SWAP70, SYNPR, TEK, TESC, TIAM2, TMPRSS15, TP63, TRIM9, TTLL11, TULP3, WWOX
MHC class I protein complex	6.0×10^{-8}	608.5	HLA-A, HLA-B, HLA-C
Phagocytic vesicle membrane	8.8×10^{-8}	159.9	DMBT1, HLA-A, HLA-B, HLA-C
Golgi membrane	2.4×10^{-7}	18.6	HLA-A, HLA-B, HLA-C, HLA-DPA1, HLA-DPB1, HLA-DQA1, HLA-DQB1
Clathrin-coated vesicle membrane	5.6×10^{-7}	50.2	HLA-DPA1, HLA-DPB1, HLA-DQA1, HLA-DQB1, HLA-DRB1
Integral to plasma membrane	6.8×10^{-7}	2.0	ABCA1, ABCA4, ABCB5, ALK, ANK2, ART3, BMPR1B, CDH4, CLCNKB, CNR2, DSCAM, EMR1, FCER2, FLVCR1, GABRR1, GPC5, GPC6, GRIK2, GRIN2A, GRM5, HLA-A, HLA-B, HLA-C, HLA-DPA1, HLA-DQA1, HLA-DQA2, HLA-DRA, HLA-DRB1, IL1RL2, KCNMB3, KL, LHCGR, MUC12, NOTCH4, NRG3, NRXN1, NRXN3, OPCML, PCDHA1, PCDHA10, PCDHA11, PCDHA2, PCDHA3, PCDHA4, PCDHA5, PCDHA6, PCDHA7, PCDHA8, PCDHAC1, PLSCR1, PPYR1, PTPRB, PTPRD, PTPRM, RAMP3, SCARB1, SCNN1G, SLC15A2, SLC17A5, SLC2A9, SLCO1B1, SLCO1B3, TEK
Intrinsic to plasma membrane	7.4×10^{-7}	1.9	ABCA1, ABCA4, ABCB5, ALK, ANK2, ART3, ASIC2, BMPR1B, CDH4, CLCNKB, CLEC1A, CNR2, DSCAM, EMR1, FCER2, FLVCR1, GABRR1, GPC5, GPC6, GRIK2, GRIN2A, GRM5, HLA-A, HLA-B, HLA-C, HLA-DPA1, HLA-DQA1, HLA-DQA2, HLA-DRA, HLA-DRB1, IL1RL2, KCNMB3, KL, LHCGR, MUC12, NOTCH4, NRG3, NRXN1, NRXN3, OPCML, OPRK1, PCDHA1, PCDHA10, PCDHA11, PCDHA2, PCDHA3, PCDHA4, PCDHA5, PCDHA6, PCDHA7, PCDHA8, PCDHAC1, PKHD1, PLSCR1, PPYR1, PTGER3, PTPRB, PTPRD, PTPRM, RAMP3, ROR1, SCARB1, SCNN1G, SLC15A2, SLC17A5, SLC2A9, SLCO1B1, SLCO1B3, SPTB, TEK, TSPAN9
Lysosomal membrane	9.8×10^{-7}	10.1	CUBN, HLA-DPA1, HLA-DPB1, HLA-DQA1, HLA-DQB1, HLA-DQB2, HLA-DRA, HLA-DRB1, HLA-DRB5, SLC17A5
Synapse part	1.2×10^{-6}	1.9	ALS2, AMPH, ANK2, ANK3, ANKS1B, CADPS2, CHRM2, CHRNA9, CHRNB3, CLSTN2, CNIH3, DBH, DDC, DISC1, DLG2, DLGAP1, DNM3, DPYSL2, EPHA7, ERBB4, ERC2, EXOC4, GABBR2, GABRB2, GABRG3, GABRR1, GLRA3, GRIA1, GRIA4, GRID2, GRIK2, GRIK3, GRIK4, GRIN2A, GRIN2B, GRIN3A, GRIP1, GRM5, GRM7, GRM8, LRFN2, LRRC7, LRRK2, LRRTM4, LYN, MAGI2, NCS1, NETO1, NRXN1, NTRK2, PCLO, PLCB4, PSD3, RAB3C, RIMS1, RIMS2, SLC1A2, STON2, SV2B, SV2C, SYN3, SYNE1, SYNPR, SYT6, SYT9, TANC1, TMEM163, TRIM9, UNC13A, UNC13C
Vacuolar membrane	1.5×10^{-6}	8.5	CUBN, HLA-DPA1, HLA-DPB1, HLA-DQA1, HLA-DQB1, HLA-DQB2, HLA-DRA, HLA-DRB1, HLA-DRB5, SLC17A5
Golgi apparatus part	1.6×10^{-6}	14.3	HLA-A, HLA-B, HLA-C, HLA-DPA1, HLA-DPB1, HLA-DQA1, HLA-DQB1
Extracellular matrix part	2.8×10^{-6}	2.3	ADAMTS1, AMTN, CCBE1, COL13A1, COL21A1, COL24A1, COL27A1, COL4A1, COL4A2, COL4A3, COL5A2, COL9A1, COLEC12, CTHRC1, EGFLAM, EMID1, EMID2, FBN2, GLDN, GRIN2B, LAMA1, LAMA2, LAMC1, LAMC2, LEPREL1, LOXL2, MATN2, NID2, NTN1, NTN4, SMOC1, SMOC2, SNCA, THBS2, THSD4, TIMP2, TNR, USH2A

GO categories in which false discovery rate is less than 0.01.

Table S24 continued.

Description	p-value	Enrichment	Genes
Early endosome membrane	5.0×10^{-6}	160.1	*HLA-A*, *HLA-B*, *HLA-C*
Organelle membrane	9.7×10^{-6}	5.7	*CPE*, *DMBT1*, *HLA-A*, *HLA-B*, *HLA-C*, *HLA-DPA1*, *HLA-DPB1*, *HLA-DQA1*, *HLA-DQB1*, *RNF144B*
Synaptic membrane	1.0×10^{-5}	2.1	*ANK2*, *ANK3*, *ANKS1B*, *CADPS2*, *CHRM2*, *CHRNA9*, *CHRNB3*, *CLSTN2*, *CNIH3*, *DISC1*, *DLG2*, *DLGAP1*, *EPHA7*, *ERC2*, *GABBR2*, *GABRB2*, *GABRG3*, *GABRR1*, *GLRA3*, *GRIA1*, *GRIA4*, *GRID2*, *GRIK2*, *GRIK3*, *GRIK4*, *GRIN2A*, *GRIN2B*, *GRIN3A*, *GRIP1*, *GRM7*, *GRM8*, *LRFN2*, *LRRC7*, *LRRTM4*, *NCS1*, *NETO1*, *NRXN1*, *PSD3*, *RIMS1*, *RIMS2*, *SLC1A2*, *SYNE1*, *TANC1*, *UNC13A*, *UNC13C*
Vacuolar part	1.3×10^{-5}	6.0	*CUBN*, *GPC5*, *HLA-DPA1*, *HLA-DPB1*, *HLA-DQA1*, *HLA-DQB1*, *HLA-DQB2*, *HLA-DRA*, *HLA-DRB1*, *HLA-DRB5*, *SLC17A5*
Endoplasmic reticulum part	1.5×10^{-5}	10.3	*HLA-A*, *HLA-B*, *HLA-C*, *HLA-DPA1*, *HLA-DPB1*, *HLA-DQA1*, *HLA-DQB1*
Neuron projection	3.3×10^{-5}	1.7	*ACTN2*, *ALS2*, *APP*, *BMPR1B*, *CACNA1A*, *CADM2*, *CDH13*, *CHL1*, *CHRM2*, *CNR2*, *CNTN4*, *CNTNAP2*, *CPLX1*, *CXADR*, *DBH*, *DCC*, *DDC*, *DNER*, *DNM3*, *DSCAM*, *EPHA7*, *EPHB1*, *GABBR2*, *GRIA1*, *GRID2*, *GRIK3*, *GRIK4*, *GRIN2B*, *GRIN3A*, *GRIP1*, *GRM7*, *GRM8*, *ITGA1*, *KALRN*, *KCNIP1*, *KCNIP4*, *KCNQ4*, *KIRREL3*, *KLHL1*, *KLHL14*, *KLHL24*, *LRRK2*, *MAPT*, *MYO10*, *MYO5A*, *MYO5B*, *NCAM2*, *NCS1*, *NOV*, *NRCAM*, *NTM*, *ODZ4*, *PEX5L*, *PRKCA*, *PTPN13*, *RAB27A*, *RAPGEF4*, *RASGRF1*, *RELN*, *RPTOR*, *SCN10A*, *SEMA3A*, *SEPT11*, *SLC1A2*, *SLC8A1*, *SNCA*, *SYNPR*, *TP63*, *TRIM9*
Synapse	7.2×10^{-5}	2.1	*ABI1*, *APBB2*, *APP*, *CADM2*, *CADPS*, *CAMK2D*, *CDH23*, *CHRM2*, *CPLX1*, *CXADR*, *EGFLAM*, *EPHA7*, *GRID2*, *GRIN3A*, *GRM7*, *MAGI2*, *MYRIP*, *NETO1*, *NRCAM*, *NRG1*, *NRXN1*, *NTM*, *NTRK2*, *PCDH15*, *PCLO*, *PHACTR1*, *PTPRF*, *RAPGEF4*, *RIMBP2*, *SDC2*, *SEPT11*, *SHISA9*, *SNCA*, *SNTB1*
Extracellular matrix	1.1×10^{-4}	1.8	*ABI3BP*, *ADAMTS12*, *ADAMTS14*, *ADAMTS16*, *ADAMTS18*, *ADAMTS2*, *ADAMTS20*, *ADAMTSL1*, *ADAMTSL2*, *AMTN*, *CDON*, *CHL1*, *COL21A1*, *COL4A1*, *COL4A2*, *COL5A2*, *CPXM2*, *DMBT1*, *DPT*, *EFEMP1*, *EGFLAM*, *FBN2*, *FGF1*, *FRAS1*, *GPC5*, *GPC6*, *GPLD1*, *HPSE2*, *IGFBP7*, *LAMA1*, *LAMA2*, *LAMC1*, *LMCD1*, *LTBP1*, *MATN2*, *MEPE*, *MMP20*, *NAV2*, *NID2*, *NOV*, *PLSCR1*, *PXDN*, *RELN*, *SMOC2*, *SPARCL1*, *TNN*, *TNR*, *VIT*, *VWF*
Protein complex	1.4×10^{-4}	4.1	*ARPC5*, *HLA-A*, *HLA-B*, *HLA-C*, *HLA-DPA1*, *HLA-DPB1*, *HLA-DQA1*, *HLA-DQB1*, *RNF144B*, *TEKT4*
Postsynaptic membrane	2.0×10^{-4}	2.0	*ANK2*, *ANK3*, *ANKS1B*, *CADPS2*, *CHRM2*, *CHRNA9*, *CHRNB3*, *CLSTN2*, *CNIH3*, *DISC1*, *DLG2*, *DLGAP1*, *EPHA7*, *GABBR2*, *GABRB2*, *GABRG3*, *GABRR1*, *GLRA3*, *GRIA1*, *GRIA4*, *GRID2*, *GRIK2*, *GRIK3*, *GRIK4*, *GRIN2A*, *GRIN2B*, *GRIN3A*, *GRIP1*, *GRM7*, *LRFN2*, *LRRC7*, *LRRTM4*, *NCS1*, *NETO1*, *PSD3*, *SYNE1*, *TANC1*
Cell-cell junction	2.1×10^{-4}	1.8	*ABCB5*, *ACTN1*, *AMTN*, *ANK2*, *ANK3*, *B4GALT1*, *CCDC85C*, *CDC42BPA*, *CDSN*, *CGNL1*, *CLDN1*, *CLDN10*, *CLDN14*, *CLDN20*, *COL13A1*, *CTNNA3*, *CXADR*, *DSC1*, *DSG1*, *FAT2*, *FNDC1*, *FRMD4A*, *IGSF5*, *INADL*, *KAZN*, *KIFC3*, *LAMA1*, *MAGI1*, *MAGI2*, *NFASC*, *NPHS2*, *PARD3*, *PARD3B*, *PDZD2*, *PKP1*, *PKP2*, *PLEKHA7*, *PTPRM*, *PTPRU*, *SCN1A*, *SHROOM3*, *SLC2A1*, *SLC8A1*, *SORBS1*, *STX2*, *TEK*, *TIAM1*, *TJP2*, *TRPC4*, *TRPC6*
Ion channel complex	2.5×10^{-4}	2.1	*AKAP6*, *ANO2*, *ANO3*, *BEST3*, *CACNA1A*, *CACNA2D1*, *CASQ2*, *CATSPERB*, *CHRNA9*, *CHRNB3*, *CLCNKB*, *CNTNAP2*, *GABRB2*, *GABRR1*, *KCNA6*, *KCNB2*, *KCNJ16*, *KCNK2*, *KCNMB1*, *KCNMB2*, *KCNMB3*, *KCNQ4*, *KCNQ5*, *PEX5L*, *RYR2*, *SCN10A*, *SCN1A*, *SCN3A*, *SCNN1G*, *TRDN*, *TRPC4*
Flagellum part	2.7×10^{-4}	19.5	*PACRG*, *SLC9C1*, *SPAG16*, *TEKT4*
Microtubule-based flagellum part	2.7×10^{-4}	19.5	*PACRG*, *SLC9C1*, *SPAG16*, *TEKT4*
Collagen type IV	3.3×10^{-4}	24.1	*COL4A1*, *COL4A2*, *COL4A3*
Proteinaceous extracellular matrix	3.9×10^{-4}	1.9	*ABI3BP*, *ADAMTS12*, *ADAMTS14*, *ADAMTS16*, *ADAMTS18*, *ADAMTS2*, *ADAMTS20*, *ADAMTSL1*, *ADAMTSL2*, *AMTN*, *CHL1*, *DMBT1*, *DPT*, *EFEMP1*, *EGFLAM*, *FBN2*, *FGF1*, *GPC5*, *GPC6*, *GPLD1*, *HPSE2*, *LAMA1*, *LTBP1*, *MATN2*, *MEPE*, *MMP20*, *NAV2*, *PXDN*, *RELN*, *SMOC2*, *SPARCL1*, *TNN*, *TNR*, *VIT*, *VWF*

GO categories in which false discovery rate is less than 0.01.
