# Supplementary material for: A Model-Based Approach for Identifying Signatures of Ancient Balancing Selection in Genetic Data
Source: PLoS Genet. 2014 Aug 21;10(8):e1004561. doi: 10.1371/journal.pgen.1004561 (PMC4140648; doi:10.1371/journal.pgen.1004561)
Supplement: Table S25 — GO component analysis of ranked signals from YRI population using the test statistic. (PDF) [file pgen.1004561.s051.pdf]

Table S25: GO component analysis of ranked signals from YRI population using the T_1 test statistic.

Description	p-value	Enrichment	Genes
Integral to lumenal side of endoplasmic reticulum membrane	6.9×10^{-20}	278.2	*HLA-A, HLA-B, HLA-C, HLA-DPA1, HLA-DPB1, HLA-DQA1, HLA-DQB1, HLA-DRB1, HLA-DRB5*
ER to Golgi transport vesicle membrane	5.8×10^{-19}	231.8	*HLA-A, HLA-B, HLA-C, HLA-DPA1, HLA-DPB1, HLA-DQA1, HLA-DQB1, HLA-DRB1, HLA-DRB5*
Transport vesicle membrane	1.6×10^{-18}	140.5	*CPE, HLA-A, HLA-B, HLA-C, HLA-DPA1, HLA-DPB1, HLA-DQA1, HLA-DQB1, HLA-DRB1, HLA-DRB5*
MHC protein complex	2.4×10^{-18}	204.5	*HLA-A, HLA-B, HLA-C, HLA-DPA1, HLA-DPB1, HLA-DQA1, HLA-DQB1, HLA-DRB1, HLA-DRB5*
Endocytic vesicle membrane	1.3×10^{-16}	96.6	*DMBT1, HLA-A, HLA-B, HLA-C, HLA-DPA1, HLA-DPB1, HLA-DQA1, HLA-DQB1, HLA-DRB1, HLA-DRB5*
Integral to endoplasmic reticulum membrane	4.3×10^{-14}	117.5	*HLA-A, HLA-B, HLA-C, HLA-DPA1, HLA-DPB1, HLA-DQA1, HLA-DQB1, HLA-DRB5*
Intrinsic to endoplasmic reticulum membrane	5.0×10^{-14}	98.9	*HLA-A, HLA-B, HLA-C, HLA-DPA1, HLA-DPB1, HLA-DQA1, HLA-DQB1, HLA-DRB5*
MHC class II protein complex	6.0×10^{-14}	331.2	*HLA-DPA1, HLA-DPB1, HLA-DQA1, HLA-DQB1, HLA-DRB1, HLA-DRB5*
Coated vesicle membrane	8.1×10^{-14}	93.3	*HLA-A, HLA-B, HLA-C, HLA-DPA1, HLA-DPB1, HLA-DQA1, HLA-DQB1, HLA-DRB5*
Cytoplasmic vesicle membrane	1.5×10^{-13}	29.5	*CPE, DMBT1, HLA-A, HLA-B, HLA-C, HLA-DPA1, HLA-DPB1, HLA-DQA1, HLA-DQB1, HLA-DRB1, HLA-DRB5*
Vesicle membrane	2.2×10^{-13}	28.3	*CPE, DMBT1, HLA-A, HLA-B, HLA-C, HLA-DPA1, HLA-DPB1, HLA-DQA1, HLA-DQB1, HLA-DRB1, HLA-DRB5*
Integral to organelle membrane	1.7×10^{-12}	56.7	*HLA-A, HLA-B, HLA-C, HLA-DPA1, HLA-DPB1, HLA-DQA1, HLA-DQB1, HLA-DRB5*
Clathrin-coated endocytic vesicle membrane	1.8×10^{-12}	210.7	*HLA-DPA1, HLA-DPB1, HLA-DQA1, HLA-DQB1, HLA-DRB1, HLA-DRB5*
Cytoplasmic vesicle part	2.5×10^{-12}	22.8	*CPE, DMBT1, HLA-A, HLA-B, HLA-C, HLA-DPA1, HLA-DPB1, HLA-DQA1, HLA-DQB1, HLA-DRB1, HLA-DRB5*
Intrinsic to organelle membrane	3.9×10^{-12}	50.7	*HLA-A, HLA-B, HLA-C, HLA-DPA1, HLA-DPB1, HLA-DQA1, HLA-DQB1, HLA-DRB5*
Trans-Golgi network membrane	7.7×10^{-12}	171.7	*HLA-DPA1, HLA-DPB1, HLA-DQA1, HLA-DQB1, HLA-DRB1, HLA-DRB5*
Plasma membrane	4.9×10^{-11}	1.4	*ABCA1, ABCA4, ABCB5, ABCC4, ABCG1, ACPP, ADAM12, ADAM28, ADCY3, ADCY5, ADD2, ADORA3, ADRA1A, AGTR1, ALDH9A1, ANGPT2, ANK1, ANK2, ANK3, ANKH, ANKS1B, ANO2, ANO6, APBB1IP, AQP8, ARAP1, ARAP3, ARHGAP24, ARHGEF18, ATP10D, ATP6V0A2, ATP6V0A4, ATP8A1, BAI3, BCR, BLK, BLNK, BMPR1B, BNC2, BST1, CACNA1A, CACNA1C, CACNG8, CADM2, CALN1, CAMK1G, CAMK2D, CAP1, CD109, CD244, CD247, CD84, CDH10, CDH13, CDH18, CDH23, CDH4, CDH7, CDH9, CDON, CELSR2, CHD1L, CHL1, CHRNB3, CLDN10, CLDN14, CLDN23, CLECL1, CLSTN2, CNGA3, CNKSR1, CNR2, CNTN1, CNTN3, CNTN4, CNTN5, CNTN6, COL13A1, COL25A1, CPE, CRTC1, CSF1R, CSMD2, CSMD3, CUBN, CXADR, CYBRD1, DAPP1, DCC, DGKH, DGKI, DIO1, DLG2, DNER, DOK7, DSC1, DSC3, DSCAM, DSG1, DSG3, DTNA, DYNC2H1, DYSF, DYTN, ELMO1, EMID2, EMR2, ENOX1, ENPP1, EPB41L2, ERBB4, ESR1, ESYT2, F5, FAM176A, FANCI, FAS, FAT2, FAT3, FHIT, FLOT1, FLVCR1, FMN1, FNDC1, FRAS1, FRMPD1, FSHR, GABBR2, GABRB3, GABRG3, GABRR1, GGT1, GLDN, GLP1R, GLRA3, GNG12, GNG2, GNG4, GPC5, GPC6, GPR111, GPR115, GPR158, GPR39, GRB10, GRIA1, GRID1, GRID2, GRIK2, GRIK4, GRIN2A, GRIP1, GRM1, GRM3, GRM5, GRM7, GRM8, GUCA1B, HLA-A, HLA-B, HLA-C, HLA-DPA1, HLA-DPB1, HLA-DQA1, HLA-DQA2, HLA-DQB1, HLA-DRA, HLA-DRB1, HLA-DRB5, HLA-F, HLA-G, HPSE2, HTR5A, IGSF5, IL1R2, INADL, INPP5D, IQGAP1, IRAK2, ITGA1, ITGA10, ITGA11, ITGA8, ITGA9, ITGB3, ITGB5, IYD, JPH3, KCNAB1, KCNC2, KCND3, KCNE1, KCNIP1, KCNIP4, KCNJ12, KCNJ15, KCNJ6, KCNK2, KCNMA1, KCNMB1, KCNMB2, KCNMB3, KCNQ4, KCNQ5, KIAA1009, KIRREL3, KL, KLRB1, KNG1, KRT5, LAMA2, LANCL2, LDLRAD3, LIM2, LPAR3, LPP, LRRC52, LRRC7, LYPD6B, MAGI1, MAGI2, MARCH1, MBP, MCC, MDGA2, MEP1B, MGAM, MPP6, MRAP2, MRPL42, MTUS1, MUC16, MYLK, MYO10, MYO16, MYO1B, MYOF, NBEA, NCAM2, NEDD4, NETO1, NFAM1, NFASC, NKAIN1, NKAIN2, NKAIN3, NOS1, NOTCH2, NOTCH4, NPSR1, NRCAM, NRXN1, NTM, NTN4, O3FAR1, OPCML, OPRK1, OR10A5, OR10C1, OR12D2, OR13C2, OR13C5, OR14A16, OR1L8, OR1N2, OR1S1, OR2AG1, OR2AK2, OR2B11, OR2L13, OR2T27, OR2T8, OR2W3, OR4C3, OR4C45, OR4S1, OR51B2, OR51B6, OR51E1, OR51F1, OR51I2, OR51V1, OR52E2, OR52E4, OR52E6, OR52J3, OR52N2, OR52N4, OR52R1, OR56B1, OR5P2, OR6C74, OR6K6, OR8B2, OR9Q1, PAK1, PALM2, PARVA, PCDH15, PDE2A, PDIA6, PDLIM5, PECAM1, PHLDB2, PIK3C2G, PIP4K2A, PKP2, PLA2R1, PLAUR, PLB1, PLCG2, PLSCR1, PLXDC1, PLXNA4, PLXNC1, PPFIBP1, PPYR1, PRKCA, PRKCB, PRKCE, PRKCH, PRKG1, PROKR2, PSD3, PTK2B, PTPN13, PTPRT, PXK, RAB17, RAC2, RAMP3, RAP1A, RASGRF2, RECK, REPS1, RFTN1, RGS6, RGS7, RGS9, RIMBP2, RIMS1, RNPEP, ROR1, RPSA, RYR2, SCARB1, SCTR, SDC2, SELL, SEMA5A, SGCG, SGCZ, SGIP1, SH3GL2, SIRPA, SKAP2, SLC12A6, SLC15A2, SLC16A14, SLC1A2, SLC1A6, SLC22A16, SLC24A2, SLC24A3, SLC24A4, SLC26A11, SLC26A9, SLC27A6, SLC28A3, SLC2A1, SLC2A9, SLC36A1, SLC36A2, SLC39A8, SLC7A1, SLC7A5, SLC8A3, SLC9A2, SLCO1B1, SLCO1B3, SNCA, SNTB1, SNTG2, SORBS1, SPRED1, SPRED2, SRP72, SULF1, SULF2, SWAP70, SYK, SYT6, TANC1, TAP1, TAP2, TEC, TEK, TES, TESC, TGFA, THSD7A, TIAM1, TJP2, TMPRSS2, TPO, TREM1, TRPC6, TRPM4, TSHR, TUBB, TULP3, UNC13C, UNC93A, UTRN, VAV2, VAV3, VNN1, VSTM4, WWOX*

GO categories in which false discovery rate is less than 0.01.

Table S25 continued.

Description	p-value	Enrichment	Genes
Endosome membrane	8.0×10^{-11}	35.3	*HLA-A, HLA-B, HLA-C, HLA-DPA1, HLA-DPB1, HLA-DQA1, HLA-DQB1, HLA-DRB5*
Endosomal part	1.2×10^{-10}	33.6	*HLA-A, HLA-B, HLA-C, HLA-DPA1, HLA-DPB1, HLA-DQA1, HLA-DQB1, HLA-DRB5*
Membrane part	1.6×10^{-10}	1.2	*AAK1, ABCA1, ABCA12, ABCA13, ABCA4, ABCB5, ABCC12, ABCC4, ABCD4, ABCG1, ABCG5, ABHD2, ABO, ACBD5, ACPP, ACY3, ADAM12, ADAM19, ADAM28, ADAM32, ADCY2, ADCY3, ADCY5, ADORA3, ADRA1A, AGPAT5, AGTR1, AJAP1, AKAP6, AKAP7, ALG8, ALK, ANK1, ANK2, ANK3, ANKH, ANO2, ANO3, ANO5, ANO6, AP1S3, AQP8, AQPEP, ARHGEF4, ART3, ASGR2, ASIC2, ASTN2, ATP10B, ATP10D, ATP2C2, ATP6V0A2, ATP6V0A4, ATP6V0E2, ATP8A1, ATP8A2, ATRNL1, AVL9, BAI3, BMPR1B, BRI3BP, BST1, BTNL2, C1GALT1, C6, CACHD1, CACNA1A, CACNA1C, CACNA2D1, CACNA2D3, CACNG8, CADM2, CALN1, CAMK2D, CASQ2, CATSPER3, CATSPER4, CATSPERB, CCDC155, CD109, CD244, CD247, CD84, CDH10, CDH13, CDH18, CDH23, CDH4, CDH7, CDH9, CDHR2, CDKAL1, CDON, CELSR1, CELSR2, CHL1, CHODL, CHRNA2, CHRNB3, CHST11, CHST8, CHST9, CLCA2, CLCNKB, CLDN10, CLDN14, CLDN23, CLEC1A, CLEC6A, CLECL1, CLIC5, CLMN, CLSTN2, CMTM7, CMTM8, CNGA1, CNGA3, CNIH3, CNR2, CNTN1, CNTN3, CNTN4, CNTN5, CNTN6, CNTNAP2, CNTNAP4, CNTNAP5, COL13A1, COL25A1, CSF1R, CSMD1, CSMD2, CSMD3, CTNNA2, CUBN, CUX1, CX-ADR, CXCL12, CYBRD1, CYP19A1, CYP2C8, CYP2E1, CYP4F11, CYP4F12, CYP4F2, CYP4F3, CYP4Z1, DAD1, DCC, DCHS2, DIO1, DLC1, DMBT1, DNAJC16, DNER, DPP6, DPY19L1, DSC1, DSC3, DSCAM, DSG1, DSG3, DYSF, EFNA5, ELFN2, EMR1, EMR2, ENPP1, ENTPD3, ENTPD5, EPB41L2, EPB41L4A, EPHA3, EPHA4, EPHA6, EPHA7, EPHB1, ERAP2, ERBB4, ERO1LB, ESR1, ESYT2, EVC, EVC2, EXT1, FAM176A, FAM176C, FAM189A1, FARP1, FAS, FAT2, FAT3, FCER2, FER, FER1L6, FGD5, FLOT1, FLT3, FLVCR1, FMO2, FMO5, FRAS1, FREM1, FRMD3, FRMPD2, FSHR, FUT9, GABBR2, GABRB3, GABRG3, GABRR1, GALNT10, GALNT2, GALNTL4, GALNTL5, GALNTL6, GCNT1, GGT1, GLDN, GLP1R, GLRA3, GNB2L1, GNG12, GNG2, GNG4, GNGT1, GPC5, GPC6, GPNMB, GPR111, GPR115, GPR137B, GPR158, GPR39, GPR78, GRIA1, GRID1, GRID2, GRIK2, GRIK4, GRIN2A, GRIP1, GRM1, GRM3, GRM5, GRM7, GRM8, HHAT, HHLA2, HK1, HLA-A, HLA-B, HLA-C, HLA-DOB, HLA-DPA1, HLA-DPB1, HLA-DQA1, HLA-DQA2, HLA-DQB1, HLA-DQB2, HLA-DRA, HLA-DRB1, HLA-DRB5, HLA-F, HLA-G, HMGCLL1, HPSE, HS3ST4, HTR5A, IFI27L1, IGDCC3, IGSF5, IL12RB2, IL1R2, IL1RL1, IL1RL2, INADL, IQGAP1, IQGAP3, ITGA1, ITGA10, ITGA11, ITGA8, ITGA9, ITGB3, ITGB5, ITGBL1, ITLN1, IYD, JKAMP, JPH3, KANK1, KCNAB1, KCNB2, KCNC2, KCND3, KCNE1, KCNE3, KCNJ12, KCNJ15, KCNJ6, KCNK18, KCNK2, KC-NMA1, KCNMB1, KCNMB2, KCNMB3, KCNQ4, KCNQ5, KIAA0922, KIAA1324L, KIAA1549, KIRREL3, KL, KLHL14, KLRB1, KREMEN1, LAMP3, LAPTM4B, LARGE, LDLRAD3, LEMD2, LGR5, LHFP, LHFPL2, LHFPL3, LIM2, LINGO2, LPAR3, LPPR1, LRFN5, LRMP, LRP1B, LRPPRC, LRRC15, LRRC52, LYPD6B, MAD1L1, MAEA, MAN1A1, MARCH1, MARCH4, MCOLN2, MCTP2, MDGA2, MEP1B, MFSD6L, MGAM, MGAT1, MGAT4C, MGAT5, MGAT5B, MGST3, MOXD1, MPP7, MSR1, MTCH1, MUC12, MUC16, MUC22, MX1, MYO10, MYO7A, MYOF, NAALADL2, NALCN, NAV3, NCALD, NCAM2, ND-FIP2, NDST4, NDUFB1, NEDD4, NETO1, NFAM1, NFASC, NIPAL3, NKAIN1, NKAIN2, NKAIN3, NLGN1, NOTCH2, NOTCH4, NOX4, NPR3, NPSR1, NR3C2, NRCAM, NRG1, NRG3, NRXN1, NRXN3, NTM, NUP210, NUP88, O3FAR1, OCA2, ODZ3, OMA1, OPCML, OPRK1, OR10A5, OR10C1, OR12D2, OR13C2, OR13C5, OR14A16, OR1L8, OR1N2, OR1S1, OR2AG1, OR2AK2, OR2B11, OR2L13, OR2T27, OR2T8, OR2W3, OR4C3, OR4C45, OR4S1, OR51B2, OR51B6, OR51E1, OR51F1, OR51I2, OR51M1, OR51V1, OR52E2, OR52E4, OR52E6, OR52J3, OR52N2, OR52N4, OR52R1, OR56B1, OR5P2, OR6C74, OR6K6, OR8B2, OR9Q1, OSTM1, OTOP1, PAM, PCDH15, PCNXL2, PCSK5, PCSK9, PDE2A, PDE3A, PDIA6, PECAM1, PEX5L, PGBD5, PI16, PIEZO2, PIGG, PIGH, PIKFYVE, PINK1, PKD1L1, PKD1L2, PKDREJ, PKHD1, PKP2, PLA2G4C, PLA2R1, PLAUR, PLB1, PLD5, PLEK, PLEKHH2, PLSCR1, PLXDC1, PLXDC2, PLXNA4, PLXNC1, PPYR1, PRKAB2, PRKCA, PROKR2, PTCHD3, PTGFRN, PTGS1, PTK2B, PTPRB, PTPRD, PTPRF, PT-PRG, PTPRH, PTPRM, PTPRN2, PTPRT, QSOX1, RAMP1, RAMP3, RASGRF2, RECK, REPS1, RFTN1, RGS6, RGS7, RGS9, RGSL1, RNF144B, RNF150, RNPEP, ROBO2, ROR1, ROR2, RYR2, RYR3, SCARB1, SCD5, SCN11A, SCN3A, SCN5A, SCN9A, SCNN1G, SCTR, SCUBE1, SDC2, SDHA, SDK1, SEC24D, SELL, SEMA5A, SEMA5B, SERAC1, SEZ6L, SGCG, SGCZ, SGIP1, SHISA2, SHISA6, SHROOM3, SIDT1, SIRPA, SLC12A6, SLC12A8, SLC15A2, SLC15A5, SLC16A14, SLC17A5, SLC19A3, SLC1A2, SLC1A6, SLC1A7, SLC22A16, SLC22A9, SLC24A2, SLC24A3, SLC24A4, SLC25A21, SLC25A37, SLC25A48, SLC26A11, SLC26A9, SLC27A6, SLC28A3, SLC2A1, SLC2A9, SLC30A9, SLC35A1, SLC35B3, SLC35F1, SLC35F3, SLC35F4, SLC36A1, SLC36A2, SLC37A1, SLC37A2, SLC38A9, SLC39A11, SLC39A12, SLC39A8, SLC4A4, SLC5A12, SLC7A1, SLC7A13, SLC7A5, SLC8A3, SLC9A2, SLC9A4, SLC9A9, SLC9C1, SLCO1B1, SLCO1B3, SNTB1, SNTG1, SNTG2, SNX16, SNX9, SOAT1, SORBS1, SORCS1, SORCS2, SPATA13, SPINK5, SPRED1, SPTB, SPTLC3, SPTSSA, SSR1, ST6GAL1, ST6GALNAC3, ST8SIA1, ST8SIA2, ST8SIA6, STEAP1B, STK39, STON2, STX7, STXBP6, SULF1, SV2C, SYBU, SYK, SYNE1, SYNGR1, SYNJ2, SYNPR, SYT5, SYT6, SYT9, TACSTD2, TAP1, TAP2, TBC1D5, TBCD, TECRL, TEK, TESC, TF, TGFA, TGFBR3, THSD7A, THSD7B, TIMM21, TLR10, TMC2, TMC5, TMCC3, TMCO1, TMCO5A, TMEM105, TMEM106B, TMEM108, TMEM110, TMEM117, TMEM128, TMEM132B, TMEM132C, TMEM132D, TMEM156, TMEM163, TMEM17, TMEM176B, TMEM199, TMEM220, TMEM229B, TMEM237, TMEM39A, TMEM44, TMEM63C, TMEM80, TMEM8A, TMPRSS11A, TMPRSS15, TMPRSS2, TMPRSS4, TMPRSS9, TMTC1, TMTC2, TNFRSF11A, TNR, TPCN2, TPO, TRAM2, TREM1, TRPC6, TRPM3, TRPM4, TSHR, TSPAN15, TSPAN18, TSPAN8, TSPAN9, UBR3, UCP2, UGT1A10, UGT1A7, UGT1A8, UGT1A9, UGT2A1, UNC5C, UNC5D, UNC93A, UST, UTRN, VIPR2, VNN1, VPS45, VSTM4, VWF, WBSCR17, WDFY4, WDR11, XKR4, XKR6, XXYLT1, XYLT1, ZDHHC13, ZDHHC14, ZDHHC7*

GO categories in which false discovery rate is less than 0.01.

Table S25 continued.

Description	p-value	Enrichment	Genes
Membrane	2.3×10^{-10}	1.2	*ABCA1, ABCA12, ABCA4, ABCB5, ABCC4, ABCD4, ABCG5, ABO, ACACB, ACBD5, ACPP, ACSBG2, ADAM12, ADAM28, ADCY2, ADCY3, ADCY5, ADD2, ADORA3, ADRA1A, AGPAT5, AGTR1, AKAP6, AKAP7, ALDH9A1, ALG8, ALS2, AMPH, ANGPT2, ANK1, ANK2, ANK3, ANKS1B, ANO2, ANO5, ANO6, AP1S3, APPBP2, AQP8, ARAP1, ARAP3, ARHGAP15, ARHGAP24, ARHGEF18, ASAP1, ATP10B, ATP10D, ATP2C2, ATP6V0A2, ATP8A1, BAI3, BCR, BICD1, BLK, BLNK, BMPR1B, BNC2, BRI3BP, BSPRY, BST1, C1GALT1, CACNA1A, CACNA1C, CACNG8, CADM2, CADPS, CALN1, CAMK1G, CAMK2D, CAP1, CASQ2, CATSPER3, CATSPER4, CBFA2T3, CCDC155, CCDC91, CCZ1B, CD109, CD244, CD247, CD84, CDH10, CDH13, CDH18, CDH4, CDH7, CDH9, CDKAL1, CDON, CELSR2, CFI, CHD1L, CHL1, CHN2, CHRNA2, CHRNB3, CHST8, CHST9, CLDN10, CLDN14, CLDN23, CLECL1, CNGA1, CNGA3, CNIH3, CNKSR1, CNR2, CNTN1, CNTN3, CNTN4, CNTN5, CNTN6, CNTNAP2, COG6, COL13A1, COL25A1, CORO2B, CPE, CRTC1, CSF1R, CSMD2, CSMD3, CUBN, CUX1, CXADR, CYB5R2, CYBRD1, CYP11B1, CYP19A1, CYP27A1, CYP2C8, CYP2E1, CYP4F11, CYP4F12, CYP4F2, CYP4F3, CYP4Z1, DAD1, DAPP1, DCC, DCHS2, DGKH, DGKI, DHRS4, DIO1, DIRAS3, DLG2, DMBT1, DNER, DOCK1, DOCK2, DOK7, DOPEY2, DSC1, DSC3, DSCAM, DSG1, DSG3, DTNA, DYNC2H1, DYSF, DYTN, EFNA5, ELMO1, EMID2, ENOX1, ENPP1, ENTPD5, EPB41L2, EPHA4, EPHA7, EPHB1, ERAP2, ERBB4, ERC2, ERO1LB, ESR1, ESYT2, EXT1, F5, FAM176A, FANCI, FAS, FAT2, FAT3, FHIT, FLOT1, FLVCR1, FMN1, FMO2, FNDC1, FRAS1, FRMPD1, FSHR, GABBR2, GABRB3, GABRG3, GABRR1, GALNT10, GALNT2, GALNTL4, GALNTL5, GALNTL6, GAS2, GCNT1, GGT1, GLDN, GLIPR2, GLP1R, GLRA3, GNG12, GNG2, GNG4, GNGT1, GPC5, GPC6, GPHN, GPR111, GPR115, GPR137B, GPR158, GPR39, GPR78, GRB10, GRIA1, GRID1, GRID2, GRIK2, GRIK4, GRIN2A, GRM1, GRM3, GRM5, GRM7, GRM8, GUCA1A, GUCA1B, HHAT, HIPK2, HK1, HLA-A, HLA-B, HLA-C, HLA-DOB, HLA-DPA1, HLA-DPB1, HLA-DQA1, HLA-DQA2, HLA-DQB1, HLA-DQB2, HLA-DRA, HLA-DRB1, HLA-DRB5, HLA-F, HLA-G, HMMR, HPSE, HPSE2, HS3ST4, HTR5A, IGSF5, IL1R2, INADL, INPP5D, IQGAP1, IRAK2, ITGA1, ITGA10, ITGA11, ITGA8, ITGA9, ITGB3, ITGB5, IYD, JKAMP, JPH3, KCNAB1, KCNB2, KCNC2, KCND3, KCNE1, KCNIP4, KCNJ12, KCNJ15, KCNJ6, KCNK18, KCNK2, KCNMA1, KCNMB1, KCNMB2, KCNMB3, KCNQ4, KCNQ5, KIAA1009, KIRREL3, KL, KLHL14, KLRB1, KNG1, KREMEN1, KRT5, KSR2, LAMA2, LAMP3, LANCL2, LDLRAD3, LIM2, LOXL2, LPAR3, LPP, LRMP, LRPPRC, LRRC52, LRRC7, LYPD6B, MAGI1, MAGI2, MAMDC2, MAN1A1, MARC2, MARCH1, MARCH4, MBP, MCC, MDGA2, MEP1B, MGAM, MGAT1, MGAT4C, MGAT5, MGAT5B, MGST3, MOXD1, MPP6, MRAP2, MRPL42, MTCH1, MTUS1, MX1, MYLK, MYO10, MYO16, MYO1B, MYO5B, MYO7A, MYOF, NAV3, NCAM2, NDFIP2, NDST4, NDUFAF6, NETO1, NFAM1, NFASC, NKAIN1, NKAIN3, NLGN1, NOS1, NOTCH2, NOTCH4, NR3C1, NR3C2, NRCAM, NRG1, NRXN1, NTM, NTN4, NUP210, O3FAR1, OAS2, OCA2, ODZ3, OMA1, OPCML, OPRK1, OR10A5, OR10C1, OR12D2, OR13C2, OR13C5, OR14A16, OR1L8, OR1N2, OR1S1, OR2AG1, OR2AK2, OR2B11, OR2L13, OR2T27, OR2T8, OR2W3, OR4C3, OR4C45, OR4S1, OR51B2, OR51B6, OR51E1, OR51F1, OR51I2, OR51M1, OR52E2, OR52E4, OR52E6, OR52J3, OR52N2, OR52N4, OR52R1, OR56B1, OR5P2, OR6C74, OR6K6, OR8B2, OR9Q1, OSTM1, PAK1, PALM2, PALMD, PARVA, PCDH15, PCLO, PCM1, PCSK2, PDE2A, PDE4D, PDGFD, PDIA6, PDLIM5, PECAM1, PEX5L, PGLYRP4, PGS1, PHLDB2, PIGG, PIGH, PIK3C2G, PIK3R6, PIKFYVE, PINK1, PIP4K2A, PKD1L1, PKDREJ, PKP2, PLA2G4C, PLA2G4E, PLA2R1, PLAUR, PLB1, PLCB1, PLCG2, PLCH1, PLSCR1, PLXDC1, PLXNA4, PLXNC1, POMP, PPFIBP1, PPP1R14C, PPP3CA, PPYR1, PRICKLE1, PRKCA, PRKCB, PRKCE, PRKCH, PRKG1, PSD3, PSTPIP2, PTGFRN, PTGS1, PTK2B, PTPN13, PTPRN2, PTPRT, PXK, RAB17, RAMP1, RAMP3, REPS1, RFTN1, RGS7, RGS9, RIMS1, RNF144B, RNPEP, ROR1, RPSA, RYR2, RYR3, SCARB1, SCD5, SCG3, SCGN, SCN5A, SCTR, SDC2, SDF4, SDHA, SEC24D, SELL, SEMA3A, SEMA5A, SEZ6L, SGCG, SGCZ, SGIP1, SH3GL2, SHISA2, SIRPA, SKAP2, SLC12A6, SLC15A2, SLC16A14, SLC17A5, SLC19A3, SLC1A2, SLC1A6, SLC1A7, SLC22A16, SLC24A2, SLC24A3, SLC24A4, SLC25A21, SLC25A37, SLC25A48, SLC26A11, SLC26A9, SLC27A6, SLC28A3, SLC2A1, SLC2A9, SLC35A1, SLC35B3, SLC36A1, SLC39A8, SLC4A4, SLC7A1, SLC7A5, SLC8A3, SLC9A2, SLC9A4, SLC9A9, SLC9C1, SLCO1B1, SLCO1B3, SNCA, SNTB1, SNTG2, SNX19, SNX4, SNX7, SNX9, SOAT1, SORBS1, SORCS1, SORCS2, SORD, SPINK5, SPRED1, SPRED2, SPTLC3, SQRDL, SRP72, SSR1, ST6GAL1, ST8SIA2, ST8SIA6, STARD13, SULF1, SULF2, SV2C, SVIL, SWAP70, SYBU, SYK, SYNE1, SYNGR1, SYNPR, SYT5, SYT6, SYT9, TACSTD2, TANC1, TAP1, TAP2, TES, TESC, THSD7A, TIAM1, TIMM21, TJP2, TLR10, TMEM106B, TMEM163, TMEM176B, TPCN2, TPO, TREM1, TRIP11, TRPC6, TRPM4, TSHR, TSPAN15, TSPAN9, TUBB, TULP3, UCP2, UGT1A7, UGT1A8, UGT1A9, UNC13C, UNC5C, UNC93A, USP32, UST, UTRN, VAV2, VAV3, VIPR2, VNN1, VPS36, VPS45, VSTM4, WBSCR17, WWOX, XXYLT1, XYLT1*
Cell projection	6.0×10^{-10}	1.7	*ACPP, ACTN4, ACTR2, ADCY2, ADCY5, ALS2, ANGPT2, APBB1IP, APOA1BP, ARAP3, ARHGAP24, ARPC5, BMPR1B, CACNA1A, CADM2, CAMK1G, CATSPER3, CATSPER4, CATSPERB, CCDC40, CDH13, CDH23, CHAT, CHL1, CLIC5, CNGA3, CNR2, CNTN4, CNTNAP2, CTNNA2, CTNNA3, CXADR, CYP19A1, DCC, DDC, DFNB31, DLD, DNAH8, DNER, DNM3, DPYSL3, DSCAM, DYNC2H1, EPHA4, EPHA7, EPHB1, EVC, EVC2, FAM65B, FER, FGD5, FKBP15, FOPNL, FRMD4B, FSCB, GABBR2, GAS7, GLI2, GNB2L1, GRIA1, GRID2, GRIK4, GRIP1, GRM1, GRM3, GRM7, GRM8, GRXCR1, HAP1, HSPB11, HTR5A, IFT122, IFT57, IQCB1, IQGAP1, IQGAP2, ITGA1, KALRN, KCND3, KCNIP1, KCNIP4, KCNJ6, KCNQ4, KIRREL3, KLHL1, KLHL14, KLHL24, LDB3, LRRC16A, MAGI1, MRI1, MTSS1, MYLK, MYO10, MYO1B, MYO5A, MYO5B, MYO7A, MYRIP, NCAM2, NLGN1, NOS1, NOV, NRCAM, NTM, PACRG, PAK1, PALLD, PALMD, PARVA, PCDH15, PCSK1, PEX5L, PKHD1, PLXDC1, PPEF2, PRKCA, PTGS1, PTK2B, PTPN13, PTPRM, PYGB, RELN, RPTOR, RSPH9, RUFY3, SCN11A, SDF4, SEMA3A, SEPT11, SLC1A2, SLC8A3, SLC9C1, SNCA, SNX9, SORD, SPATA13, SVIL, SWAP70, SYNPR, SYT5, TEK, TESC, TIAM2, TMPRSS15, TRAPPC4, TRIM9, TTLL11, TULP3, WDR69, WWOX*

GO categories in which false discovery rate is less than 0.01.

Table S25 continued.

Description	p-value	Enrichment	Genes
Cell junction	2.0×10^{-9}	1.8	ABCB5, AFAP1, AJAP1, AMPH, AMTN, ANK2, ANK3, ANKS1B, APBB1IP, ARHGAP24, ARHGAP26, ARHGEF18, BSPRY, CACNG8, CADM2, CADPS, CCDC85C, CDC42BPA, CDHR2, CDSN, CGNL1, CHRNA2, CHRNB3, CLCA2, CLDN10, CLDN14, CLDN23, CNIH3, CNKSR1, COL13A1, CTNNA2, CTNNA3, CTNND2, CXADR, DLC1, DLG2, DOK7, DSC1, DSC3, DSG1, DSG3, DTNA, EPB41L2, EPHA4, EPHX2, ERC2, FAT2, FER, FMN1, FNDC1, FRMD4A, FRMPD2, GABBR2, GABRB3, GABRG3, GABRR1, GLRA3, GPHN, GRIA1, GRID1, GRID2, GRIK2, GRIK4, GRIN2A, GRIP1, IGSF5, INADL, IQGAP1, IQGAP3, IQSEC1, ITGB5, LIM2, LIMD1, LPP, LRRC7, MAGI1, MAGI2, MPP7, NETO1, NFASC, NLGN1, NOX4, NRAP, NRXN1, PAK1, PALLD, PARVA, PCLO, PDLIM5, PECAM1, PHACTR1, PKP2, PLXDC1, POLR1E, PSD3, PTK2B, PTPRM, RIMBP2, RIMS1, SCN5A, SEPT11, SHROOM3, SLC2A1, SNCA, SNTB1, SORBS1, SV2C, SVIL, SYNGR1, SYNPR, SYT5, SYT6, SYT9, TANC1, TBCD, TEK, TES, TIAM1, TJP2, TMEM163, TNS1, TRIM9, TRPC6, UNC13C, UTRN, XIRP2
Plasma membrane part	2.2×10^{-9}	1.4	AAK1, ABCA1, ABCA4, ABCB5, ABCG1, ABCG5, ACY3, ADCY3, ADORA3, ADRA1A, AGTR1, AJAP1, AKAP7, ALK, ANK1, ANK2, ANK3, ANKH, AQP8, ARHGEF4, ART3, ASIC2, ATP6V0A4, BMPR1B, C6, CACNA1A, CACNA1C, CACNA2D1, CACNG8, CAMK2D, CASQ2, CATSPER3, CATSPER4, CATSPERB, CD244, CD247, CD84, CDH13, CDH4, CDHR2, CELSR1, CHRNA2, CHRNB3, CLCA2, CLCNKB, CLEC1A, CNGA1, CNIH3, CNR2, CNTNAP2, COL25A1, CSF1R, CTNNA2, CUBN, CXADR, CXCL12, CYBRD1, CYP4F12, CYP4F2, DCC, DLC1, DSCAM, DSG1, DYSF, EFNA5, EMR1, EMR2, ENPP1, EPHA3, EPHA4, EPHA6, EPHA7, EPHB1, ERBB4, ESR1, EVC, EVC2, FAS, FCER2, FER, FGD5, FLOT1, FLT3, FLVCR1, FRMPD2, GABBR2, GABRB3, GABRR1, GGT1, GLP1R, GLRA3, GNB2L1, GNG12, GNG2, GNG4, GNGT1, GPC5, GPC6, GPNMB, GPR137B, GPR39, GRIA1, GRID2, GRIK2, GRIK4, GRIN2A, GRM1, GRM3, GRM5, GRM7, GRM8, HLA-A, HLA-B, HLA-C, HLA-DOB, HLA-DPA1, HLA-DPB1, HLA-DQA1, HLA-DQA2, HLA-DQB1, HLA-DQB2, HLA-DRA, HLA-DRB1, HLA-DRB5, HLA-F, HLA-G, HTR5A, IGDCC3, IL12RB2, IL1RL1, IL1RL2, INADL, IQGAP1, IQGAP3, ITGA1, ITGA10, ITGA11, ITGA8, ITGA9, ITGB3, ITGB5, ITGBL1, ITLN1, KANK1, KCNB2, KCNC2, KCND3, KCNE1, KCNJ15, KCNJ6, KCNK18, KCNK2, KCNMA1, KCNMB1, KCNMB2, KCNMB3, KCNQ4, KCNQ5, KL, LGR5, LPAR3, LRMP, MAEA, MEP1B, MGAM, MPP7, MSR1, MUC12, MYO10, MYO7A, MYOF, NEDD4, NLGN1, NOTCH2, NOTCH4, NPR3, NRCAM, NRG1, NRG3, NRXN1, NRXN3, OPCML, OPRK1, PCSK9, PKHD1, PLA2R1, PLAUR, PLB1, PLEK, PLSCR1, PPYR1, PRKAB2, PTPRB, PTPRD, PTPRF, PTPRG, PTPRH, PTPRM, PTPRN2, RAMP1, RAMP3, RGS6, RGS7, RGS9, RNPEP, ROBO2, ROR1, ROR2, SCARB1, SCN11A, SCN3A, SCN5A, SCN9A, SCNN1G, SCTR, SCUBE1, SDC2, SELL, SGCG, SGCZ, SGIP1, SHROOM3, SLC12A6, SLC15A2, SLC17A5, SLC1A2, SLC1A6, SLC26A9, SLC2A1, SLC2A9, SLC35A1, SLC4A4, SLC5A12, SLC7A1, SLC7A5, SLC9A4, SLC9C1, SLCO1B1, SLCO1B3, SNTB1, SNTG1, SNTG2, SNX9, SORBS1, SPATA13, SPRED1, SPTB, STK39, SYK, SYNJ2, TACSTD2, TBCD, TEK, TESC, TF, TGFA, TGFBR3, TMEM17, TMEM8A, TMPRSS11A, TMPRSS11F, TMPRSS2, TMPRSS9, TNFRSF11A, TPO, TSHR, TSPAN15, TSPAN9, UTRN, VIPR2, VWF
Cell projection part	2.8×10^{-9}	1.8	AAK1, ALS2, AMPH, ANK3, ANKS1B, ARHGEF4, ATP6V0A4, CACNA1C, CACNG8, CATSPER3, CATSPER4, CDH23, CNGA1, CNIH3, CNTNAP2, CTNND2, CUBN, CXADR, CYBRD1, CYP19A1, DCC, DFNB31, DLG2, DNAH11, DNAH14, DNAH5, DNAH8, DNAH9, DNM3, DPYSL3, DSCAM, DYNC2H1, EMR2, EPHA4, ERBB4, ERC2, ESR1, EVC, FGD5, FKBP15, FOPNL, FSCB, GRIA1, GRID2, GRIK2, GRIK4, GRIN2A, GRM1, GRM3, GRM5, GRM7, HAP1, HSPB11, IFT122, IFT57, ITLN1, KANK1, KIRREL3, LRRC7, MAGI2, MAP2, MBP, MYO10, MYO5A, MYO5B, NETO1, NFASC, NLGN1, NOS1, NR3C1, NRCAM, NRXN1, ODF1, PACRG, PAK1, PALMD, PCSK1, PDLIM5, PKD1L1, PKHD1, PLCB4, PLEK, PSD3, PTK2B, PTPRF, ROBO2, RSPH9, RUFY3, SCARB1, SEPT11, SLC1A2, SLC8A3, SLC9C1, SNCA, SNTG1, SPAG16, SPATA13, SPOCK1, SYNJ2, TANC1, TEKT1, TEKT4, TEKT5, TESC, TIAM2, TMEM17, TMEM237, TTLL11
Golgi membrane	5.8×10^{-9}	21.3	HLA-A, HLA-B, HLA-C, HLA-DPA1, HLA-DPB1, HLA-DQA1, HLA-DQB1, HLA-DRB5
Clathrin-coated vesicle membrane	1.5×10^{-8}	54.5	HLA-DPA1, HLA-DPB1, HLA-DQA1, HLA-DQB1, HLA-DRB1, HLA-DRB5
Golgi apparatus part	5.2×10^{-8}	16.3	HLA-A, HLA-B, HLA-C, HLA-DPA1, HLA-DPB1, HLA-DQA1, HLA-DQB1, HLA-DRB5

GO categories in which false discovery rate is less than 0.01.

Table S25 continued.

Description	p-value	Enrichment	Genes
Intrinsic to membrane	9.2×10^{-8}	1.2	*ABCA1, ABCA12, ABCA13, ABCA4, ABCB5, ABCC12, ABCC4, ABCD4, ABCG1, ABCG5, ABHD2, ABO, ACBD5, ACPP, ADAM12, ADAM19, ADAM28, ADAM32, ADCY2, ADCY3, ADCY5, ADORA3, ADRA1A, AGPAT5, AGTR1, AJAP1, ALG8, ALK, ANK2, ANKH, AQP8, AQPEP, ART3, ASGR2, ASIC2, ASTN2, ATP10B, ATP10D, ATP2C2, ATP6V0A2, ATP6V0A4, ATP6V0E2, ATP8A1, ATP8A2, ATRNL1, AVL9, BAI3, BMPR1B, BRI3BP, BST1, BTNL2, C1GALT1, CACHD1, CACNA1A, CACNA1C, CACNA2D3, CADM2, CALN1, CCDC155, CD109, CD244, CD247, CD84, CDH10, CDH13, CDH18, CDH23, CDH4, CDH7, CDH9, CDHR2, CDKAL1, CDON, CELSR1, CELSR2, CHL1, CHODL, CHRNA2, CHRNB3, CHST11, CHST8, CHST9, CLCA2, CLCNKB, CLDN10, CLDN14, CLDN23, CLEC1A, CLEC6A, CLECL1, CLMN, CLSTN2, CMTM7, CMTM8, CNGA1, CNGA3, CNR2, CNTN1, CNTN3, CNTN4, CNTN5, CNTN6, CNTNAP2, CNTNAP4, CNTNAP5, COL13A1, COL25A1, CSF1R, CSMD1, CSMD2, CSMD3, CUX1, CXADR, CYBRD1, CYP2E1, CYP4F11, CYP4F12, CYP4F3, CYP4Z1, DAD1, DCC, DCHS2, DIO1, DNAJC16, DNER, DPP6, DPY19L1, DPY19L4, DSC1, DSC3, DSCAM, DSG1, DSG3, DYSF, EFNA5, ELFN2, EMR1, EMR2, ENPP1, ENTPD3, ENTPD5, EPHA3, EPHA4, EPHA6, EPHA7, EPHB1, ERAP2, ERBB4, ESR1, ESYT2, EVC, EVC2, EXT1, FAM176A, FAM176C, FAM189A1, FAS, FAT2, FAT3, FCER2, FER1L6, FLOT1, FLT3, FLVCR1, FMO2, FMO5, FRAS1, FREM1, FRMD3, FSHR, FUT9, GABBR2, GABRB3, GABRG3, GABRR1, GALNT10, GALNT2, GALNTL4, GALNTL5, GALNTL6, GCNT1, GGT1, GLDN, GLP1R, GLRA3, GPC5, GPC6, GPNMB, GPR111, GPR115, GPR137B, GPR158, GPR39, GPR78, GRID1, GRID2, GRIK2, GRIK4, GRIN2A, GRM1, GRM3, GRM5, GRM7, GRM8, HHAT, HHLA2, HLA-A, HLA-B, HLA-C, HLA-DOB, HLA-DPA1, HLA-DPB1, HLA-DQA1, HLA-DQA2, HLA-DQB1, HLA-DQB2, HLA-DRA, HLA-DRB1, HLA-DRB5, HLA-F, HLA-G, HS3ST4, HTR5A, IFI27L1, IGDCC3, IGSF5, IL12RB2, IL1R2, IL1RL1, IL1RL2, ITGB3, ITLN1, IYD, JKAMP, JPH3, KCNAB1, KCNE3, KCNJ12, KCNJ15, KCNK18, KCNMA1, KCNMB2, KCNMB3, KIAA0922, KIAA1324L, KIAA1549, KIRREL3, KL, KLRB1, KREMEN1, LAMP3, LAPTM4B, LARGE, LDLRAD3, LEMD2, LGR5, LHFP, LHFPL2, LHFPL3, LIM2, LINGO2, LPAR3, LPPR1, LRFN5, LRMP, LRP1B, LRRC15, LRRC52, LYPD6B, MAEA, MAN1A1, MARCH1, MARCH4, MCOLN2, MCTP2, MDGA2, MEP1B, MFSD6L, MGAM, MGAT1, MGAT4C, MGAT5, MGAT5B, MGST3, MOXD1, MSR1, MTCH1, MUC12, MUC16, MUC22, MYOF, NAALADL2, NALCN, NCAM2, NDFIP2, NDST4, NDUFB1, NETO1, NFAM1, NFASC, NIPAL3, NKAIN1, NKAIN2, NKAIN3, NLGN1, NOTCH2, NOTCH4, NOX4, NPR3, NPSR1, NRCAM, NRG1, NRG3, NRXN1, NRXN3, NTM, O3FAR1, OCA2, ODZ3, OMA1, OPCML, OPRK1, OR10A5, OR10C1, OR12D2, OR13C2, OR13C5, OR14A16, OR1L8, OR1N2, OR1S1, OR2AG1, OR2AK2, OR2B11, OR2L13, OR2T27, OR2T8, OR2W3, OR4C3, OR4C45, OR4S1, OR51B2, OR51B6, OR51E1, OR51F1, OR51I2, OR51M1, OR51V1, OR52E2, OR52E4, OR52E6, OR52J3, OR52N2, OR52N4, OR52R1, OR56B1, OR5P2, OR6C74, OR6K6, OR8B2, OR9Q1, OSTM1, OTOP1, PAM, PCDH15, PCNXL2, PCSK5, PDE3A, PECAM1, PGBD5, PI16, PIEZO2, PIGG, PINK1, PKD1L1, PKD1L2, PKDREJ, PKHD1, PKP2, PLA2R1, PLAUR, PLB1, PLD5, PLEKHH2, PLSCR1, PLXDC1, PLXDC2, PLXNA4, PLXNC1, PPYR1, PROKR2, PTCHD3, PTGFRN, PTPRB, PTPRD, PTPRF, PTPRG, PTPRH, PTPRM, PTPRN2, PTPRT, QSOX1, RAMP1, RAMP3, RECK, RGSL1, RNF144B, RNF150, ROBO2, ROR1, ROR2, RYR3, SCARB1, SCD5, SCN5A, SCNN1G, SCTR, SDC2, SDK1, SELL, SEMA5A, SEMA5B, SERAC1, SEZ6L, SGCG, SGCZ, SHISA2, SHISA6, SIDT1, SIRPA, SLC12A6, SLC12A8, SLC15A2, SLC15A5, SLC16A14, SLC17A5, SLC19A3, SLC1A2, SLC1A6, SLC1A7, SLC22A16, SLC22A9, SLC24A2, SLC24A3, SLC24A4, SLC25A21, SLC25A37, SLC25A48, SLC26A11, SLC26A9, SLC27A6, SLC28A3, SLC2A1, SLC2A9, SLC30A9, SLC35A1, SLC35B3, SLC35F1, SLC35F3, SLC35F4, SLC36A1, SLC36A2, SLC37A1, SLC37A2, SLC38A9, SLC39A11, SLC39A12, SLC39A8, SLC4A4, SLC5A12, SLC7A1, SLC7A13, SLC7A5, SLC8A3, SLC9A2, SLC9A4, SLC9A9, SLC9C1, SLCO1B1, SLCO1B3, SOAT1, SORCS1, SORCS2, SPTB, SPTLC3, SPTSSA, SSR1, ST6GAL1, ST6GALNAC3, ST8SIA1, ST8SIA2, ST8SIA6, STEAP1B, STX7, STXBP6, SV2C, SYBU, SYNE1, SYNGR1, SYNPR, SYT5, SYT6, SYT9, TACSTD2, TAP1, TAP2, TECRL, TEK, TGFA, TGFBR3, THSD7A, THSD7B, TIMM21, TLR10, TMC2, TMC5, TMCC3, TMCO1, TMCO5A, TMEM105, TMEM106B, TMEM108, TMEM110, TMEM117, TMEM128, TMEM132B, TMEM132C, TMEM132D, TMEM156, TMEM163, TMEM17, TMEM176B, TMEM199, TMEM220, TMEM229B, TMEM237, TMEM39A, TMEM44, TMEM63C, TMEM80, TMEM8A, TMPRSS11A, TMPRSS11F, TMPRSS15, TMPRSS2, TMPRSS4, TMPRSS9, TMTC1, TMTC2, TNFRSF11A, TPCN2, TPO, TRAM2, TREM1, TRPC6, TRPM3, TRPM4, TSHR, TSPAN15, TSPAN18, TSPAN8, TSPAN9, UBR3, UCP2, UGT1A10, UGT1A7, UGT1A8, UGT1A9, UGT2A1, UNC5C, UNC5D, UNC93A, UST, VIPR2, VNN1, VPS45, VSTM4, WBSCR17, WDFY4, WDR11, XKR4, XKR6, XXYLT1, XYLT1, ZDHHC13, ZDHHC14, ZDHHC7*
Vacuolar membrane	1.4×10^{-7}	31.3	*HLA-DPA1, HLA-DPB1, HLA-DQA1, HLA-DQB1, HLA-DRB1, HLA-DRB5*
Lysosomal membrane	1.6×10^{-7}	37.1	*HLA-DPA1, HLA-DPB1, HLA-DQA1, HLA-DQB1, HLA-DRB1, HLA-DRB5*
Phagocytic vesicle membrane	1.9×10^{-7}	131.7	*DMBT1, HLA-A, HLA-B, HLA-C*
MHC class I protein complex	2.6×10^{-7}	405.7	*HLA-A, HLA-B, HLA-C*

GO categories in which false discovery rate is less than 0.01.

Table S25 continued.

Description	p-value	Enrichment	Genes
Integral to membrane	2.7×10^{-7}	1.2	*ABCA1, ABCA12, ABCA13, ABCA4, ABCB5, ABCC12, ABCC4, ABCD4, ABCG1, ABCG5, ABHD2, ABO, ACBD5, ACPP, ADAM12, ADAM19, ADAM28, ADAM32, ADCY2, ADCY3, ADCY5, ADORA3, ADRA1A, AGPAT5, AGTR1, AJAP1, ALG8, ALK, ANK2, ANKH, AQP8, AQPEP, ART3, ASGR2, ASIC2, ASTN2, ATP10B, ATP10D, ATP2C2, ATP6V0A2, ATP6V0A4, ATP6V0E2, ATP8A1, ATP8A2, ATRNL1, AVL9, BAI3, BMPR1B, BRI3BP, BTNL2, C1GALT1, CACHD1, CACNA1A, CACNA1C, CACNA2D3, CADM2, CALN1, CCDC155, CD244, CD247, CD84, CDH10, CDH18, CDH23, CDH4, CDH7, CDH9, CDHR2, CDKAL1, CDON, CELSR1, CELSR2, CHL1, CHODL, CHRNA2, CHRNB3, CHST11, CHST8, CHST9, CLCA2, CLCNKB, CLDN10, CLDN14, CLDN23, CLEC1A, CLEC6A, CLECL1, CLMN, CLSTN2, CMTM7, CMTM8, CNGA1, CNGA3, CNR2, CNTNAP2, CNTNAP4, CNTNAP5, COL13A1, COL25A1, CSF1R, CSMD1, CSMD2, CSMD3, CUX1, CXADR, CYBRD1, CYP4F11, CYP4F12, CYP4F3, CYP4Z1, DAD1, DCC, DCHS2, DIO1, DNAJC16, DNER, DPP6, DPY19L1, DPY19L4, DSC1, DSC3, DSCAM, DSG1, DSG3, DYSF, ELFN2, EMR1, EMR2, ENPP1, ENTPD3, ENTPD5, EPHA3, EPHA4, EPHA6, EPHA7, EPHB1, ERAP2, ERBB4, ESR1, ESYT2, EVC, EVC2, EXT1, FAM176A, FAM176C, FAM189A1, FAS, FAT2, FAT3, FCER2, FER1L6, FLOT1, FLT3, FLVCR1, FMO2, FMO5, FRAS1, FREM1, FRMD3, FSHR, FUT9, GABBR2, GABRB3, GABRG3, GABRR1, GALNT10, GALNT2, GALNTL4, GALNTL5, GALNTL6, GCNT1, GGT1, GLDN, GLP1R, GLRA3, GPC5, GPC6, GPNMB, GPR111, GPR115, GPR137B, GPR158, GPR39, GPR78, GRID1, GRID2, GRIK2, GRIK4, GRIN2A, GRM1, GRM3, GRM5, GRM7, GRM8, HHAT, HHLA2, HLA-A, HLA-B, HLA-C, HLA-DOB, HLA-DPA1, HLA-DPB1, HLA-DQA1, HLA-DQA2, HLA-DQB1, HLA-DQB2, HLA-DRA, HLA-DRB1, HLA-DRB5, HLA-F, HLA-G, HS3ST4, HTR5A, IFI27L1, IGDCC3, IGSF5, IL12RB2, IL1R2, IL1RL1, IL1RL2, ITGB3, IYD, JKAMP, JPH3, KCNAB1, KCNE3, KCNJ12, KCNJ15, KCNK18, KCNMA1, KCNMB2, KCNMB3, KIAA0922, KIAA1324L, KIAA1549, KIRREL3, KL, KLRB1, KREMEN1, LAMP3, LAPTM4B, LARGE, LDLRAD3, LEMD2, LGR5, LHFP, LHFPL2, LHFPL3, LIM2, LINGO2, LPAR3, LPPR1, LRFN5, LRMP, LRP1B, LRRC15, LRRC52, MAEA, MAN1A1, MARCH1, MARCH4, MCOLN2, MCTP2, MEP1B, MFSD6L, MGAM, MGAT1, MGAT4C, MGAT5, MGAT5B, MGST3, MOXD1, MSR1, MTCH1, MUC12, MUC16, MUC22, MYOF, NAALADL2, NALCN, NCAM2, NDFIP2, NDST4, NDUFB1, NETO1, NFAM1, NFASC, NIPAL3, NKAIN1, NKAIN2, NKAIN3, NLGN1, NOTCH2, NOTCH4, NOX4, NPR3, NPSR1, NRCAM, NRG1, NRG3, NRXN1, NRXN3, O3FAR1, OCA2, ODZ3, OMA1, OPCML, OPRK1, OR10A5, OR10C1, OR12D2, OR13C2, OR13C5, OR14A16, OR1L8, OR1N2, OR1S1, OR2AG1, OR2AK2, OR2B11, OR2L13, OR2T27, OR2T8, OR2W3, OR4C3, OR4C45, OR4S1, OR51B2, OR51B6, OR51E1, OR51F1, OR51I2, OR51M1, OR51V1, OR52E2, OR52E4, OR52E6, OR52J3, OR52N2, OR52N4, OR52R1, OR56B1, OR5P2, OR6C74, OR6K6, OR8B2, OR9Q1, OSTM1, OTOP1, PAM, PCDH15, PCNXL2, PCSK5, PDE3A, PECAM1, PGBD5, PI16, PIEZO2, PIGG, PINK1, PKD1L1, PKD1L2, PKDREJ, PKHD1, PKP2, PLA2R1, PLAUR, PLB1, PLD5, PLEKHH2, PLSCR1, PLXDC1, PLXDC2, PLXNA4, PLXNC1, PPYR1, PROKR2, PTCHD3, PTGFRN, PTPRB, PTPRD, PTPRF, PTPRG, PTPRH, PTPRM, PTPRN2, PTPRT, QSOX1, RAMP1, RAMP3, RGSL1, RNF144B, RNF150, ROBO2, ROR1, ROR2, RYR3, SCARB1, SCD5, SCN5A, SCNN1G, SCTR, SDC2, SDK1, SELL, SEMA5A, SEMA5B, SERAC1, SEZ6L, SGCG, SGCZ, SHISA2, SHISA6, SIDT1, SIRPA, SLC12A6, SLC12A8, SLC15A2, SLC15A5, SLC16A14, SLC17A5, SLC19A3, SLC1A6, SLC1A7, SLC22A16, SLC22A9, SLC24A2, SLC24A3, SLC24A4, SLC25A21, SLC25A37, SLC25A48, SLC26A11, SLC26A9, SLC27A6, SLC28A3, SLC2A1, SLC2A9, SLC30A9, SLC35A1, SLC35B3, SLC35F1, SLC35F3, SLC35F4, SLC36A1, SLC36A2, SLC37A1, SLC37A2, SLC38A9, SLC39A11, SLC39A12, SLC39A8, SLC4A4, SLC5A12, SLC7A1, SLC7A13, SLC7A5, SLC8A3, SLC9A2, SLC9A4, SLC9A9, SLC9C1, SLCO1B1, SLCO1B3, SOAT1, SORCS1, SORCS2, SPTLC3, SPTSSA, SSR1, ST6GAL1, ST6GALNAC3, ST8SIA1, ST8SIA2, ST8SIA6, STEAP1B, STX7, STXBP6, SV2C, SYBU, SYNE1, SYNGR1, SYNPR, SYT5, SYT6, SYT9, TACSTD2, TAP1, TAP2, TECRL, TEK, TGFA, TGFBR3, THSD7A, THSD7B, TIMM21, TLR10, TMC2, TMC5, TMCC3, TMCO1, TMCO5A, TMEM105, TMEM106B, TMEM108, TMEM110, TMEM117, TMEM128, TMEM132B, TMEM132C, TMEM132D, TMEM156, TMEM163, TMEM17, TMEM176B, TMEM199, TMEM220, TMEM229B, TMEM237, TMEM39A, TMEM44, TMEM63C, TMEM80, TMEM8A, TMPRSS11A, TMPRSS11F, TMPRSS15, TMPRSS2, TMPRSS4, TMPRSS9, TMTC1, TMTC2, TNFRSF11A, TPCN2, TPO, TRAM2, TREM1, TRPC6, TRPM3, TRPM4, TSHR, TSPAN15, TSPAN18, TSPAN8, TSPAN9, UBR3, UCP2, UGT1A10, UGT1A7, UGT1A8, UGT1A9, UGT2A1, UNC5C, UNC5D, UNC93A, UST, VIPR2, VNN1, VPS45, VSTM4, WBSCR17, WDFY4, WDR11, XKR4, XKR6, XXYLT1, XYLT1, ZDHHC13, ZDHHC14, ZDHHC7*
Endoplasmic reticulum part	7.2×10^{-7}	11.7	*HLA-A, HLA-B, HLA-C, HLA-DPA1, HLA-DPB1, HLA-DQA1, HLA-DQB1, HLA-DRB5*
Synapse part	8.8×10^{-7}	1.9	*AAK1, ALS2, AMPH, ANK2, ANK3, ANKS1B, CACNA1C, CACNG8, CHRNA2, CHRNB3, CLSTN2, CNIH3, CTNND2, CYP19A1, DDC, DLG2, DNM3, EPHA4, EPHA7, ERBB4, ERC2, ESR1, GABBR2, GABRB3, GABRG3, GABRR1, GLRA3, GPHN, GRIA1, GRID1, GRID2, GRIK2, GRIK4, GRIN2A, GRIP1, GRM1, GRM3, GRM5, GRM7, GRM8, LRRC7, MAGI2, NETO1, NLGN1, NR3C1, NRXN1, PCLO, PDE2A, PDLIM5, PLCB4, PSD3, PTK2B, PTPRN2, RIMS1, SLC1A2, SPOCK1, STON2, SV2C, SYNE1, SYNGR1, SYNPR, SYT5, SYT6, SYT9, TANC1, TMEM163, TRAPPC4, TRIM9, UNC13C, UTRN*
Neuron projection	1.4×10^{-6}	1.8	*ALS2, BMPR1B, CACNA1A, CADM2, CAMK1G, CDH13, CHAT, CHL1, CNR2, CNTN4, CNTNAP2, CTNNA2, CXADR, CYP19A1, DCC, DFNB31, DNER, DNM3, DSCAM, EPHA4, EPHA7, EPHB1, FKBP15, GABBR2, GNB2L1, GRIA1, GRID2, GRIK4, GRIP1, GRM1, GRM3, GRM7, GRM8, HAP1, HTR5A, IQGAP1, ITGA1, KALRN, KCND3, KCNIP1, KCNIP4, KCNJ6, KCNQ4, KIRREL3, KLHL1, KLHL14, KLHL24, MYO10, MYO5A, MYO5B, NCAM2, NLGN1, NOS1, NOV, NRCAM, NTM, PAK1, PALMD, PCSK1, PEX5L, PLXDC1, PRKCA, PTK2B, PTPN13, RELN, RPTOR, SCN11A, SEMA3A, SEPT11, SLC1A2, SLC8A3, SNCA, SYNPR, SYT5, TRIM9*
Vacuolar part	2.2×10^{-6}	20.3	*HLA-DPA1, HLA-DPB1, HLA-DQA1, HLA-DQB1, HLA-DRB1, HLA-DRB5*

GO categories in which false discovery rate is less than 0.01.

Table S25 continued.

Description	p-value	Enrichment	Genes
Synaptic membrane	1.0×10^{-5}	2.2	*ANK2, ANK3, ANKS1B, CACNG8, CHRNB3, CLSTN2, CNIH3, DLG2, EPHA4, EPHA7, ERC2, GABBR2, GABRB3, GABRG3, GABRR1, GRIA1, GRID1, GRID2, GRIK2, GRIK4, GRIN2A, GRIP1, GRM1, GRM3, GRM7, GRM8, LRRC7, NETO1, NLGN1, NRXN1, PDE2A, PDLIM5, PSD3, RIMS1, SLC1A2, SYNE1, TANC1, UNC13C, UTRN*
Organelle membrane	2.2×10^{-5}	4.6	*CPE, DMBT1, HLA-A, HLA-B, HLA-C, HLA-DPA1, HLA-DPB1, HLA-DQA1, HLA-DQB1, HLA-DRB1, HLA-DRB5, RNF144B*
Early endosome membrane	2.4×10^{-5}	106.8	*HLA-A, HLA-B, HLA-C*
Cytoskeleton	3.2×10^{-5}	1.6	*ABLIM1, ACTG2, ACTL7B, ACTN4, ACTR2, ADH4, AFAP1, AMPH, ANK1, ANK2, ANK3, APBB1IP, ARAP3, ARHGAP24, ARHGAP26, ARPC5, BICD1, CAP1, CCNB2, CDSN, CLIC5, CORO2B, CTNNA2, CTNNA3, CTTNBP2NL, DAPK1, DENND2A, DNAH9, DOCK2, ELMO1, EPB41L4A, ERC2, FAM65B, FARP1, FER, FGD5, FHOD3, FMN1, FRMD3, FRMD4A, FRMD4B, FRMPD1, FRMPD2, FYB, GNB2L1, GPHN, HAP1, IFT43, INPP5D, IQGAP2, KALRN, KLHL1, KLHL5, LANCL2, LDB3, LRPPRC, LRRFIP1, MAD1L1, MAEA, MAP3K1, MICAL2, MTSS1, MYOZ2, MYPN, MYRIP, NOS1, PARVA, PCLO, PDLIM5, PHLDB2, PLEKHH2, PRKCE, PSTPIP2, PTPN13, PTPN14, PTPN21, RSPH9, SCIN, SGCG, SGCZ, SHROOM3, SKA1, SLC30A9, SNCA, SNTB1, SNTG1, SNTG2, SPRR2B, SPRR2E, SPRR2G, SPTB, STK39, SVIL, SYBU, SYNE1, TEC, TNS1, TRIM9, TRIP11, TUBB, UTRN, WASF3*
Protein complex	3.5×10^{-5}	4.2	*HLA-A, HLA-B, HLA-C, HLA-DPA1, HLA-DPB1, HLA-DQA1, HLA-DQB1, HLA-DRB5, MLL3, SNTG2, TEKT4*
Ion channel complex	4.6×10^{-5}	2.0	*AKAP6, ANO2, ANO3, ANO5, ANO6, CACNA1A, CACNA1C, CACNA2D1, CACNG8, CASQ2, CATSPER3, CATSPER4, CATSPERB, CHRNA2, CHRNB3, CLCNKB, CLIC5, CNTNAP2, GABRB3, GABRG3, GABRR1, GLRA3, KCNB2, KCNC2, KCND3, KCNE1, KCNJ6, KCNK2, KCNMA1, KCNMB1, KCNMB2, KCNMB3, KCNQ4, KCNQ5, PEX5L, RYR2, SCN11A, SCN3A, SCN5A, SCN9A, SCNN1G*
Collagen type IV	7.7×10^{-5}	13.9	*COL4A1, COL4A2, COL4A3, COL4A4*
Cilium	8.2×10^{-5}	2.1	*ADCY5, CATSPER3, CATSPER4, CCDC40, CDH23, CNGA3, DFNB31, DYNC2H1, EVC, EVC2, FOPNL, FSCB, GLI2, GRXCR1, HSPB11, IFT122, IFT57, IQCB1, MYO5A, MYO7A, MYRIP, PACRG, PCDH15, PKHD1, PPEF2, PRKCA, PTGS1, RSPH9, SLC9C1, SORD, TTLL11, TULP3, WDR69*
Synapse	9.9×10^{-5}	2.3	*CADM2, CADPS, CAMK2D, CDH23, CXADR, DOK7, EPHA4, EPHA7, GRID2, GRM7, MAGI2, MYO7A, MYRIP, NETO1, NLGN1, NRCAM, NRG1, NRXN1, NTM, PCDH15, PCLO, PHACTR1, PTPRF, RIMBP2, SDC2, SEPT11, SNCA, SNTB1, SPOCK1, UTRN*
Flagellum part	1.5×10^{-4}	5.0	*CATSPER3, CATSPER4, FSCB, PACRG, SLC9C1, SPAG16, TEKT1, TEKT4, TEKT5*
Microtubule-based flagellum part	1.5×10^{-4}	5.0	*CATSPER3, CATSPER4, FSCB, PACRG, SLC9C1, SPAG16, TEKT1, TEKT4, TEKT5*
Extracellular matrix part	1.7×10^{-4}	2.3	*AMTN, COL11A1, COL13A1, COL24A1, COL25A1, COL2A1, COL4A1, COL4A2, COL4A3, COL4A4, COL5A1, COL9A1, COLEC11, EMID1, EMID2, FBN2, GLDN, LAMA2, LAMA4, LAMC2, LEPREL1, LOXL2, MSR1, NID2, NTN4, SNCA, THBS2, TNR*
Macromolecular complex	1.8×10^{-4}	3.6	*HLA-A, HLA-B, HLA-C, HLA-DPA1, HLA-DPB1, HLA-DQA1, HLA-DQB1, HLA-DRB5, MLL3, SNTG2, TEKT4*
Postsynaptic density	1.9×10^{-4}	2.4	*ALS2, ANKS1B, CACNA1C, CACNG8, CTNND2, DLG2, DNM3, EPHA4, ERBB4, GRIA1, GRID2, GRIN2A, GRM1, GRM3, GRM5, LRRC7, MAGI2, NETO1, NLGN1, NR3C1, PDLIM5, PLCB4, PSD3, PTK2B, SPOCK1, TANC1*
Postsynaptic membrane	2.6×10^{-4}	2.1	*ANK2, ANK3, ANKS1B, CACNG8, CHRNB3, CLSTN2, CNIH3, DLG2, EPHA4, EPHA7, GABBR2, GABRB3, GABRG3, GABRR1, GRIA1, GRID1, GRID2, GRIK2, GRIK4, GRIN2A, GRIP1, GRM3, GRM7, LRRC7, NETO1, NLGN1, PSD3, SYNE1, TANC1, UTRN*
Sheet-forming collagen	3.6×10^{-4}	11.1	*COL4A1, COL4A2, COL4A3, COL4A4*

GO categories in which false discovery rate is less than 0.01.
