# Supplementary material for: A Model-Based Approach for Identifying Signatures of Ancient Balancing Selection in Genetic Data
Source: PLoS Genet. 2014 Aug 21;10(8):e1004561. doi: 10.1371/journal.pgen.1004561 (PMC4140648; doi:10.1371/journal.pgen.1004561)
Supplement: Table S26 — GO component analysis of ranked signals from CEU population using the test statistic. (PDF) [file pgen.1004561.s052.pdf]

Table S26: GO component analysis of ranked signals from CEU population using the T_2 test statistic.

Description	p-value	Enrichment	Genes
Integral to lumenal side of endoplasmic reticulum membrane	2.8×10^{-14}	73.0	*HLA-A*, *HLA-B*, *HLA-C*, *HLA-DPA1*, *HLA-DPB1*, *HLA-DQA1*, *HLA-DQB1*, *HLA-DRA*, *HLA-DRB1*
ER to Golgi transport vesicle membrane	1.9×10^{-13}	60.9	*HLA-A*, *HLA-B*, *HLA-C*, *HLA-DPA1*, *HLA-DPB1*, *HLA-DQA1*, *HLA-DQB1*, *HLA-DRA*, *HLA-DRB1*
Plasma membrane	5.7×10^{-13}	1.5	*ABCA1*, *ABCA4*, *ABCB5*, *ABCG8*, *ACPP*, *ADAM12*, *ADAM28*, *ADCY3*, *ADCY5*, *ADD2*, *ALDH9A1*, *ALPL*, *ANGPT1*, *ANGPT2*, *ANK2*, *ANK3*, *ANKS1B*, *ANO2*, *APBB1IP*, *ARHGAP24*, *ARHGEF18*, *ASAH2*, *ATP10D*, *ATP12A*, *ATP2B4*, *ATP6V0A4*, *ATP8A1*, *BAI3*, *BLNK*, *BMPR1B*, *BNC2*, *BTN3A1*, *BTN3A2*, *CACNA1A*, *CACNA1C*, *CADM2*, *CALN1*, *CAP1*, *CASR*, *CDH10*, *CDH13*, *CDH4*, *CDON*, *CHD1L*, *CHRM2*, *CHRNB3*, *CLDN10*, *CLDN14*, *CLEC12A*, *CLSTN2*, *CNGA3*, *CNR2*, *CNTN1*, *CNTN4*, *CNTN5*, *COL13A1*, *CPE*, *CSMD2*, *CSMD3*, *CUBN*, *CYBRD1*, *DCC*, *DCHS2*, *DGKH*, *DIO1*, *DISC1*, *DLG2*, *DLGAP1*, *DNER*, *DSC1*, *DSCAM*, *DSG1*, *DSG3*, *DTNA*, *DYM*, *DYNC2H1*, *DYSF*, *DYTN*, *ELMO1*, *ELTD1*, *EMID2*, *ENPP1*, *ENPP6*, *ERBB4*, *ESYT2*, *F5*, *FABP2*, *FAS*, *FAT2*, *FHIT*, *FLVCR1*, *FNDC1*, *FRAS1*, *FRMPD1*, *FSHR*, *GABBR1*, *GABRB2*, *GABRG3*, *GABRR1*, *GFRAL*, *GPC5*, *GPC6*, *GPHN*, *GPR111*, *GPR139*, *GPR146*, *GPR158*, *GPR39*, *GRB10*, *GRIA1*, *GRID2*, *GRIK2*, *GRIN2A*, *GRIP1*, *GRIP2*, *GRM5*, *GRM8*, *HLA-A*, *HLA-B*, *HLA-C*, *HLA-DOA*, *HLA-DPA1*, *HLA-DPB1*, *HLA-DQA1*, *HLA-DQA2*, *HLA-DQB1*, *HLA-DQB2*, *HLA-DRA*, *HLA-DRB1*, *HLA-DRB5*, *HLA-G*, *HPSE2*, *IGSF5*, *INADL*, *INPP5D*, *ITGA1*, *IYD*, *KCNAB1*, *KCNH7*, *KCNIP4*, *KCNJ12*, *KCNK2*, *KCNMB2*, *KCNMB3*, *KCNQ3*, *KCNQ5*, *KIRREL3*, *KITLG*, *KL*, *KLRB1*, *KNG1*, *LAMA2*, *LHCGR*, *LPP*, *LRFN2*, *LRRC52*, *LSAMP*, *LYPD6B*, *MAGI2*, *MAPT*, *MARCH1*, *MDGA2*, *MPP5*, *MRAP2*, *MTUS1*, *MUC16*, *MYH9*, *MYO10*, *MYO16*, *MYO1B*, *MYOF*, *NBEA*, *NCAM2*, *NCS1*, *NEDD4*, *NETO1*, *NKAIN1*, *NKAIN3*, *NMUR2*, *NOTCH4*, *NPSR1*, *NRCAM*, *NRP1*, *NRXN1*, *NSL1*, *NTM*, *NTN4*, *NUP35*, *OPCML*, *OPRK1*, *OR10AG1*, *OR10C1*, *OR12D2*, *OR13C5*, *OR14A16*, *OR1N2*, *OR2AK2*, *OR2L13*, *OR2W3*, *OR4C3*, *OR4C45*, *OR51B2*, *OR51B6*, *OR51E1*, *OR51F1*, *OR51I2*, *OR51M1*, *OR52E2*, *OR52E6*, *OR52J3*, *OR52N4*, *OR5AC2*, *OR5F1*, *OR5I1*, *OR5P2*, *OR5W2*, *OR8H3*, *OR8I2*, *OR9K2*, *PALM2*, *PCDH15*, *PCDH17*, *PCDHA12*, *PCDHA13*, *PCDHA9*, *PDIA6*, *PHLDB2*, *PIK3C2B*, *PKDREJ*, *PKP1*, *PKP2*, *PLA2R1*, *PLCG2*, *PLSCR1*, *PLXNA4*, *PPYR1*, *PRKCE*, *PRKG1*, *PSD3*, *PTPN13*, *PTPRT*, *PTPRU*, *RAB31*, *RAMP3*, *RGS3*, *RGS6*, *RGS7*, *RIMBP2*, *RIMS2*, *RNPEP*, *RPSA*, *RTKN2*, *RYR2*, *SCARB1*, *SCN3B*, *SDF4*, *SELE*, *SEMA5A*, *SEMA6D*, *SGCG*, *SGCZ*, *SIRPA*, *SKAP2*, *SLC10A2*, *SLC12A6*, *SLC14A1*, *SLC14A2*, *SLC15A2*, *SLC16A14*, *SLC19A3*, *SLC1A2*, *SLC24A4*, *SLC27A6*, *SLC28A1*, *SLC28A3*, *SLC2A9*, *SLC7A7*, *SLC8A1*, *SLC9A4*, *SLCO1B1*, *SLCO1B3*, *SLCO5A1*, *SLCO6A1*, *SNCA*, *SNTG2*, *SPRED1*, *STYK1*, *SWAP70*, *SYK*, *TANC1*, *TAP1*, *TAP2*, *TEC*, *TECTA*, *TEK*, *TES*, *THSD7A*, *TIAM1*, *TPO*, *TRPC4*, *TRPC6*, *TSHR*, *TULP3*, *UNC13C*, *UNC93A*, *VAV2*, *VAV3*, *VNN1*, *WWOX*
MHC protein complex	7.5×10^{-13}	53.7	*HLA-A*, *HLA-B*, *HLA-C*, *HLA-DPA1*, *HLA-DPB1*, *HLA-DQA1*, *HLA-DQB1*, *HLA-DRA*, *HLA-DRB1*
Transport vesicle membrane	7.9×10^{-13}	55.3	*CPE*, *HLA-A*, *HLA-B*, *HLA-C*, *HLA-DPA1*, *HLA-DPB1*, *HLA-DQA1*, *HLA-DQB1*, *HLA-DRB1*

GO categories in which false discovery rate is less than 0.01.

Table S26 continued.

Description	p-value	Enrichment	Genes
Intrinsic to membrane	1.0×10^{-12}	1.3	*ABCA1, ABCA13, ABCA4, ABCB5, ABCG8, ABO, ACBD5, ACPP, ADAM12, ADAM28, ADAM29, ADCY3, ADCY5, AIFM2, AJAP1, ALK, ALPL, ANK2, ART3, ASAH2, ASIC2, ASTN2, ATP10D, ATP2B4, ATP2C2, ATP6V0A4, ATP8A1, ATRNL1, BAI3, BMPR1B, BRI3BP, BTBD11, BTN3A1, BTN3A2, CACNA1A, CACNA1C, CACNA2D3, CADM2, CALN1, CASR, CDH10, CDH13, CDH4, CDHR2, CDKAL1, CDON, CHODL, CHRM2, CHRNB3, CHST11, CLCNKB, CLDN1, CLDN10, CLDN14, CLEC12A, CLEC1A, CLEC1B, CLEC6A, CLSTN2, CMTM7, CMTM8, CNGA3, CNR2, CNTN1, CNTN4, CNTN5, CNTNAP2, CNTNAP4, CNTNAP5, COL13A1, COL23A1, COLEC12, CSMD1, CSMD2, CSMD3, CUX1, CXADR, CYBRD1, CYP2E1, CYP4F12, CYP4F3, CYP4Z1, DAD1, DCC, DCHS2, DDR1, DERL3, DIO1, DISP1, DNER, DPP6, DPY19L4, DSC1, DSCAM, DSG1, DSG3, DYSF, ELOVL2, ELTD1, EMR1, ENPP1, ENPP6, EPHA6, EPHA7, EPHB1, ERAP1, ERAP2, ERBB4, ESYT2, EVC, EVC2, FAM189A1, FAS, FAT2, FAT3, FAT4, FCER2, FER1L6, FLT3, FLVCR1, FMO2, FMO5, FRAS1, FRRS1, FSHR, FUT9, GABBR1, GABRB2, GABRG3, GABRR1, GALNT10, GALNT2, GALNTL4, GALNTL6, GFRAL, GPC5, GPC6, GPR111, GPR139, GPR146, GPR158, GPR39, GRID2, GRIK2, GRIK3, GRIN2A, GRM5, GRM7, GRM8, HHAT, HHLA2, HLA-A, HLA-B, HLA-C, HLA-DOA, HLA-DPA1, HLA-DPB1, HLA-DQA1, HLA-DQA2, HLA-DQB1, HLA-DQB2, HLA-DRA, HLA-DRB1, HLA-DRB5, HLA-G, HS3ST4, HS6ST3, IFI27L1, IGSF5, IL12RB2, IL1RL2, IMMP2L, ITPR2, IYD, KCNAB1, KCNH7, KCNJ12, KCNMB2, KCNMB3, KIAA0922, KIAA1324L, KIRREL3, KITLG, KL, KLRB1, LAMP3, LAPTM4B, LGR5, LHCGR, LHFPL2, LRFN2, LRFN5, LRP1B, LRRC52, LSAMP, LYPD6B, MAN1A1, MARCH1, MARCH4, MDGA2, MEGF10, MEGF11, MGAT1, MGAT5, MGST3, MRS2, MS4A12, MUC12, MUC16, MUC22, MYOF, NAALADL2, NALCN, NCAM2, NDST3, NDUFB1, NDUFB4, NETO1, NFASC, NKAIN1, NKAIN3, NMUR1, NMUR2, NOTCH4, NPSR1, NRCAM, NRG1, NRG3, NRP1, NRXN1, NTM, OCA2, ODZ3, ODZ4, OMA1, OPCML, OPRK1, OR10AG1, OR10C1, OR12D2, OR13C5, OR14A16, OR1L8, OR1N2, OR2AK2, OR2L13, OR2W3, OR4C3, OR4C45, OR51B2, OR51B6, OR51E1, OR51F1, OR51I2, OR51M1, OR52E2, OR52E6, OR52J3, OR52N4, OR5AC2, OR5F1, OR5I1, OR5P2, OR5W2, OR8H3, OR8I2, OR9K2, OTOP1, PCDH15, PCDH17, PCDHA1, PCDHA10, PCDHA11, PCDHA12, PCDHA13, PCDHA2, PCDHA3, PCDHA4, PCDHA5, PCDHA6, PCDHA7, PCDHA8, PCDHA9, PCDHAC1, PCNXL2, PDE3A, PGBD5, PI16, PIEZO2, PIGG, PINK1, PKD1L1, PKD1L2, PKDREJ, PKHD1, PKP2, PLA2R1, PLAUR, PLD5, PLEKHH2, PLSCR1, PLXDC2, PLXNA4, PNPLA3, PPYR1, PRPH2, PTCHD3, PTCHD4, PTPRB, PTPRD, PTPRF, PTPRM, PTPRT, PTPRU, QSOX1, RAMP3, RASAL1, RNF144B, RNF150, ROBO2, RRP12, RYR3, SCARB1, SCD5, SCN3B, SCNN1G, SDK1, SEC22A, SEC61B, SELE, SEMA5A, SEMA6D, SGCG, SGCZ, SIRPA, SLC10A2, SLC12A6, SLC12A8, SLC14A1, SLC14A2, SLC15A2, SLC15A5, SLC16A14, SLC17A5, SLC19A3, SLC1A2, SLC22A9, SLC24A4, SLC25A21, SLC25A25, SLC25A37, SLC27A6, SLC28A1, SLC28A3, SLC2A9, SLC30A9, SLC35B3, SLC35F3, SLC37A1, SLC38A9, SLC39A11, SLC5A12, SLC7A7, SLC8A1, SLC9A4, SLC9A9, SLC9C1, SLCO1B1, SLCO1B3, SLCO5A1, SLCO6A1, SORCS1, SORCS2, SORCS3, SPCS3, SPPL2C, SPTB, SPTLC3, SSR1, ST6GALNAC3, ST8SIA1, ST8SIA6, STEAP1B, STXBP6, STYK1, SYBU, SYNE1, SYNJ2BP, SYNPR, SYT9, TAP1, TAP2, TAS2R19, TAS2R20, TECTA, TEK, THSD7A, THSD7B, TM4SF4, TMC2, TMCC3, TMCO4, TMEM106B, TMEM117, TMEM128, TMEM132B, TMEM132C, TMEM132D, TMEM156, TMEM163, TMEM17, TMEM2, TMEM220, TMEM229B, TMEM244, TMEM51, TMEM63C, TMPRSS15, TMTC1, TMTC2, TNFRSF11A, TPO, TRAM2, TRHDE, TRPA1, TRPC4, TRPC6, TRPM3, TSHR, TSPAN9, UGT2A1, UGT2B7, UNC5C, UNC80, UNC93A, UST, VNN1, VOPP1, VSTM2B, WBSCR17, WDR11, XKR6, XXYLT1, XYLT1, YIPF1, ZDHHC14, ZDHHC7*

GO categories in which false discovery rate is less than 0.01.

Table S26 continued.

Description	p-value	Enrichment	Genes
Membrane part	1.9×10^{-12}	1.3	*ABCA1, ABCA13, ABCA4, ABCB5, ABCG8, ABO, ACBD5, ACPP, ADAM12, ADAM28, ADAM29, ADCY3, ADCY5, AIFM2, AJAP1, AKAP6, ALK, ALPL, ANGPT1, ANK2, ANK3, ANO2, ANO3, ARHGEF4, ART3, ASAH2, ASIC2, ASTN2, ATP10D, ATP12A, ATP2B4, ATP2C2, ATP6V0A4, ATP8A1, ATRNL1, BAI3, BBS9, BMPR1B, BRI3BP, BTBD11, BTN3A1, BTN3A2, C8A, CACNA1A, CACNA1C, CACNA2D3, CADM2, CALN1, CAMK2D, CASQ2, CASR, CATSPERB, CDH10, CDH13, CDH4, CDHR2, CDKAL1, CDON, CHODL, CHRM2, CHRNB3, CHST11, CLCNKB, CLDN1, CLDN10, CLDN14, CLEC12A, CLEC1A, CLEC1B, CLEC6A, CLSTN2, CMTM7, CMTM8, CNGA3, CNR2, CNTN1, CNTN4, CNTN5, CNTNAP2, CNTNAP4, CNTNAP5, COL13A1, COL23A1, COLEC12, CSMD1, CSMD2, CSMD3, CTNNA2, CUBN, CUX1, CXADR, CXCL12, CYBRD1, CYP2E1, CYP4F11, CYP4F12, CYP4F2, CYP4F3, CYP4Z1, DAB2, DAD1, DCC, DCHS2, DDR1, DERL3, DIO1, DISP1, DLC1, DMBT1, DNER, DPP6, DPY19L4, DSC1, DSCAM, DSG1, DSG3, DYSF, ELOVL2, ELTD1, EMR1, ENPP1, ENPP6, EPHA6, EPHA7, EPHB1, ERAP1, ERAP2, ERBB4, ERO1LB, ESYT2, EVC, EVC2, FAM163A, FAM189A1, FAS, FAT2, FAT3, FAT4, FCER2, FER, FER1L6, FGD5, FLT3, FLVCR1, FMO2, FMO5, FRAS1, FRRS1, FSHR, FUT9, GABBR1, GABRB2, GABRG3, GABRR1, GALNT10, GALNT2, GALNTL4, GALNTL6, GFRAL, GOLM1, GPC5, GPC6, GPR111, GPR139, GPR146, GPR158, GPR39, GRIA1, GRID2, GRIK2, GRIK3, GRIN2A, GRIP1, GRM5, GRM7, GRM8, HHAT, HHLA2, HK1, HLA-A, HLA-B, HLA-C, HLA-DOA, HLA-DPA1, HLA-DPB1, HLA-DQA1, HLA-DQA2, HLA-DQB1, HLA-DQB2, HLA-DRA, HLA-DRB1, HLA-DRB5, HLA-G, HMGCLL1, HPSE, HS3ST4, HS6ST3, IFI27L1, IGSF5, IL12RB2, IL1RL2, IMMP2L, INADL, ITGA1, ITGA2, ITGB5, ITGBL1, ITPR2, IYD, KANK1, KCNA6, KCNAB1, KCNH7, KCNJ12, KCNK2, KCNMB2, KCNMB3, KCNQ3, KCNQ5, KIAA0922, KIAA1324L, KIRREL3, KITLG, KL, KLHL14, KLRB1, LAMP3, LAPTM4B, LGR5, LHCGR, LHFPL2, LRFN2, LRFN5, LRP1B, LRRC52, LSAMP, LYN, LYPD6B, MAN1A1, MARCH1, MARCH4, MDGA2, MEGF10, MEGF11, MGAT1, MGAT5, MGST3, MRS2, MS4A12, MUC12, MUC16, MUC22, MYH9, MYO10, MYOF, NAALADL2, NALCN, NAV3, NCALD, NCAM2, NDST3, NDUFA12, NDUFB1, NDUFB4, NEDD4, NETO1, NFASC, NKAIN1, NKAIN3, NMUR1, NMUR2, NOTCH4, NOXO1, NPSR1, NRCAM, NRG1, NRG3, NRP1, NRXN1, NTM, NUP210, NUP35, NUP88, OCA2, ODZ3, ODZ4, OMA1, OPCML, OPRK1, OR10AG1, OR10C1, OR12D2, OR13C5, OR14A16, OR1L8, OR1N2, OR2AK2, OR2L13, OR2W3, OR4C3, OR4C45, OR51B2, OR51B6, OR51E1, OR51F1, OR51I2, OR51M1, OR52E2, OR52E6, OR52J3, OR52N4, OR5AC2, OR5F1, OR5I1, OR5P2, OR5W2, OR8H3, OR8I2, OR9K2, OTOP1, PCDH15, PCDH17, PCDHA1, PCDHA10, PCDHA11, PCDHA12, PCDHA13, PCDHA2, PCDHA3, PCDHA4, PCDHA5, PCDHA6, PCDHA7, PCDHA8, PCDHA9, PCDHAC1, PCNXL2, PCSK9, PDE3A, PDIA6, PGBD5, PI16, PIEZO2, PIGG, PINK1, PKD1L1, PKD1L2, PKDREJ, PKHD1, PKP2, PLA2G4C, PLA2R1, PLAUR, PLD1, PLD5, PLEK, PLEKHH2, PLSCR1, PLXDC2, PLXNA4, PNPLA3, PPYR1, PRKAB2, PRPH2, PTCHD3, PTCHD4, PTGS1, PTPRB, PTPRD, PTPRF, PTPRM, PTPRT, PTPRU, QSOX1, RAB27A, RAMP3, RASAL1, RGS6, RGS7, RNF144B, RNF150, RNPEP, ROBO2, RRP12, RYR2, RYR3, SCARB1, SCD5, SCN3B, SCNN1G, SDHA, SDK1, SEC22A, SEC24D, SEC61B, SELE, SEMA5A, SEMA6D, SGCG, SGCZ, SHISA9, SIRPA, SLC10A2, SLC12A6, SLC12A8, SLC14A1, SLC14A2, SLC15A2, SLC15A5, SLC16A14, SLC17A5, SLC19A3, SLC1A2, SLC22A9, SLC24A4, SLC25A21, SLC25A25, SLC25A37, SLC27A6, SLC28A1, SLC28A3, SLC2A9, SLC30A9, SLC35B3, SLC35F3, SLC37A1, SLC38A9, SLC39A11, SLC5A12, SLC7A7, SLC8A1, SLC9A4, SLC9A9, SLC9C1, SLCO1B1, SLCO1B3, SLCO5A1, SLCO6A1, SNTG1, SNTG2, SNX5, SNX9, SORCS1, SORCS2, SORCS3, SPATA13, SPCS3, SPINK5, SPPL2C, SPRED1, SPTB, SPTLC3, SSR1, ST6GALNAC3, ST8SIA1, ST8SIA6, STEAP1B, STK39, STON2, STXBP6, STYK1, SYBU, SYK, SYNE1, SYNJ2BP, SYNPR, SYT9, TAP1, TAP2, TAS2R19, TAS2R20, TECTA, TEK, THSD7A, THSD7B, TM4SF4, TMC2, TMCC3, TMCO4, TMEM106B, TMEM117, TMEM128, TMEM132B, TMEM132C, TMEM132D, TMEM156, TMEM163, TMEM17, TMEM2, TMEM220, TMEM229B, TMEM244, TMEM51, TMEM63C, TMPRSS15, TMTC1, TMTC2, TNFRSF11A, TNR, TPO, TRAM2, TRHDE, TRPA1, TRPC4, TRPC6, TRPM3, TSHR, TSPAN9, UGT2A1, UGT2B7, UNC5C, UNC80, UNC93A, UST, VNN1, VOPP1, VSTM2B, VWF, WBSCR17, WDR11, WWC1, XKR6, XXYLT1, XYLT1, YIPF1, ZDHHC14, ZDHHC7*

GO categories in which false discovery rate is less than 0.01.

Table S26 continued.

Description	p-value	Enrichment	Genes
Integral to membrane	4.6×10^{-12}	1.3	*ABCA1, ABCA13, ABCA4, ABCB5, ABCG8, ABO, ACBD5, ACPP, ADAM12, ADAM28, ADAM29, ADCY3, ADCY5, AIFM2, AJAP1, ALK, ALPL, ANK2, ART3, ASAH2, ASIC2, ASTN2, ATP10D, ATP2B4, ATP2C2, ATP6V0A4, ATP8A1, ATRNL1, BAI3, BMPR1B, BRI3BP, BTBD11, BTN3A1, BTN3A2, CACNA1A, CACNA1C, CACNA2D3, CADM2, CALN1, CASR, CDH10, CDH4, CDHR2, CDKAL1, CDON, CHODL, CHRM2, CHRNB3, CHST11, CLCNKB, CLDN1, CLDN10, CLDN14, CLEC12A, CLEC1A, CLEC1B, CLEC6A, CLSTN2, CMTM7, CMTM8, CNGA3, CNR2, CNTNAP2, CNTNAP4, CNTNAP5, COL13A1, COL23A1, COLEC12, CSMD1, CSMD2, CSMD3, CUX1, CXADR, CYBRD1, CYP4F12, CYP4F3, CYP4Z1, DAD1, DCC, DCHS2, DDR1, DERL3, DIO1, DISP1, DNER, DPP6, DPY19L4, DSC1, DSCAM, DSG1, DSG3, DYSF, ELOVL2, ELTD1, EMR1, ENPP1, ENPP6, EPHA6, EPHA7, EPHB1, ERAP1, ERAP2, ERBB4, ESYT2, EVC, EVC2, FAM189A1, FAS, FAT2, FAT3, FAT4, FCER2, FER1L6, FLT3, FLVCR1, FMO2, FMO5, FRAS1, FRRS1, FSHR, FUT9, GABBR1, GABRB2, GABRG3, GABRR1, GALNT10, GALNT2, GALNTL4, GALNTL6, GFRAL, GPC5, GPC6, GPR111, GPR139, GPR146, GPR158, GPR39, GRID2, GRIK2, GRIK3, GRIN2A, GRM5, GRM7, GRM8, HHAT, HHLA2, HLA-A, HLA-B, HLA-C, HLA-DOA, HLA-DPA1, HLA-DPB1, HLA-DQA1, HLA-DQA2, HLA-DQB1, HLA-DQB2, HLA-DRA, HLA-DRB1, HLA-DRB5, HLA-G, HS3ST4, HS6ST3, IFI27L1, IGSF5, IL12RB2, IL1RL2, IMMP2L, ITPR2, IYD, KCNAB1, KCNH7, KCNJ12, KCNMB2, KCNMB3, KIAA0922, KIAA1324L, KIRREL3, KITLG, KL, KLRB1, LAMP3, LAPTM4B, LGR5, LHCGR, LHFPL2, LRFN2, LRFN5, LRP1B, LRRC52, MAN1A1, MARCH1, MARCH4, MEGF10, MEGF11, MGAT1, MGAT5, MGST3, MRS2, MS4A12, MUC12, MUC16, MUC22, MYOF, NAALADL2, NALCN, NCAM2, NDST3, NDUFB1, NDUFB4, NETO1, NFASC, NKAIN1, NKAIN3, NMUR1, NMUR2, NOTCH4, NPSR1, NRCAM, NRG1, NRG3, NRP1, NRXN1, OCA2, ODZ3, ODZ4, OMA1, OPCML, OPRK1, OR10AG1, OR10C1, OR12D2, OR13C5, OR14A16, OR1L8, OR1N2, OR2AK2, OR2L13, OR2W3, OR4C3, OR4C45, OR51B2, OR51B6, OR51E1, OR51F1, OR51I2, OR51M1, OR52E2, OR52E6, OR52J3, OR52N4, OR5AC2, OR5F1, OR5I1, OR5P2, OR5W2, OR8H3, OR8I2, OR9K2, OTOP1, PCDH15, PCDH17, PCDHA1, PCDHA10, PCDHA11, PCDHA12, PCDHA13, PCDHA2, PCDHA3, PCDHA4, PCDHA5, PCDHA6, PCDHA7, PCDHA8, PCDHA9, PCDHAC1, PCNXL2, PDE3A, PGBD5, PI16, PIEZO2, PIGG, PINK1, PKD1L1, PKD1L2, PKDREJ, PKHD1, PKP2, PLA2R1, PLAUR, PLD5, PLEKHH2, PLSCR1, PLXDC2, PLXNA4, PNPLA3, PPYR1, PRPH2, PTCHD3, PTCHD4, PTPRB, PTPRD, PTPRF, PTPRM, PTPRT, PTPRU, QSOX1, RAMP3, RNF144B, RNF150, ROBO2, RRP12, RYR3, SCARB1, SCD5, SCN3B, SCNN1G, SDK1, SEC22A, SEC61B, SELE, SEMA5A, SEMA6D, SGCG, SGCZ, SIRPA, SLC10A2, SLC12A6, SLC12A8, SLC14A1, SLC14A2, SLC15A2, SLC15A5, SLC16A14, SLC17A5, SLC19A3, SLC1A2, SLC22A9, SLC24A4, SLC25A21, SLC25A25, SLC25A37, SLC27A6, SLC28A1, SLC28A3, SLC2A9, SLC30A9, SLC35B3, SLC35F3, SLC37A1, SLC38A9, SLC39A11, SLC5A12, SLC7A7, SLC8A1, SLC9A4, SLC9A9, SLC9C1, SLCO1B1, SLCO1B3, SLCO5A1, SLCO6A1, SORCS1, SORCS2, SORCS3, SPCS3, SPPL2C, SPTLC3, SSR1, ST6GALNAC3, ST8SIA1, ST8SIA6, STEAP1B, STXBP6, STYK1, SYBU, SYNE1, SYNJ2BP, SYNPR, SYT9, TAP1, TAP2, TAS2R19, TAS2R20, TEK, THSD7A, THSD7B, TM4SF4, TMC2, TMCC3, TMCO4, TMEM106B, TMEM117, TMEM128, TMEM132B, TMEM132C, TMEM132D, TMEM156, TMEM163, TMEM17, TMEM2, TMEM220, TMEM229B, TMEM244, TMEM51, TMEM63C, TMPRSS15, TMTC1, TMTC2, TNFRSF11A, TPO, TRAM2, TRHDE, TRPA1, TRPC4, TRPC6, TRPM3, TSHR, TSPAN9, UGT2A1, UGT2B7, UNC5C, UNC80, UNC93A, UST, VNN1, VOPP1, VSTM2B, WBSCR17, WDR11, XKR6, XXYLT1, XYLT1, YIPF1, ZDHHC14, ZDHHC7*
Endocytic vesicle membrane	1.2×10^{-11}	24.8	*CUBN, DMBT1, HLA-A, HLA-B, HLA-C, HLA-DPA1, HLA-DPB1, HLA-DQA1, HLA-DQB1, HLA-DRA, HLA-DRB1*

GO categories in which false discovery rate is less than 0.01.

Table S26 continued.

Description	p-value	Enrichment	Genes
Membrane	3.0×10^{-11}	1.2	*ABCA1, ABCA4, ABCB5, ABCC11, ABCC4, ABCG8, ABO, ACACB, ACBD5, ACER1, ACPP, ACSBG2, ACSL1, ADAM12, ADAM28, ADCY3, ADCY5, ADD2, ADORA3, AFAP1L2, AIFM2, AKAP12, AKAP6, ALDH9A1, ALPL, ALS2, AMPH, ANGPT1, ANGPT2, ANK2, ANK3, ANKS1B, ANO2, ANTXR2, AP1S3, AP3B1, APBA2, APBB1IP, APBB2, APP, AQP8, AQP9, ARHGAP24, ARHGEF18, ASAH2, ASAP1, ATP10B, ATP10D, ATP12A, ATP2A3, ATP2B4, ATP2C2, ATP6V0A4, ATP6V1D, ATP8A1, ATP8B1, AVEN, B4GALT1, BAI3, BBS9, BCL2L14, BCR, BICD1, BLNK, BMPR1B, BNC2, BRI3BP, BSPRY, BTN3A1, BTN3A2, C3, C8A, CACNA1A, CACNA1C, CACNB2, CADM2, CADPS, CADPS2, CALN1, CAMK2D, CAP1, CASQ2, CASR, CBFA2T3, CD244, CD52, CDH10, CDH12, CDH13, CDH18, CDH4, CDH9, CDKAL1, CDON, CHD1L, CHL1, CHN2, CHRM2, CHRNB3, CHST11, CLCN6, CLDN10, CLDN14, CLDN16, CLDN23, CLEC12A, CLSTN2, CNGA3, CNR2, CNTN1, CNTN2, CNTN4, CNTN5, CNTNAP2, COG5, COG6, COL13A1, COL23A1, CORO2B, COX16, CPAMD8, CPE, CRYM, CSMD2, CSMD3, CUBN, CUX1, CXADR, CYB5R2, CYBRD1, CYP2E1, CYP4F11, CYP4F12, CYP4F2, CYP4F3, CYP4F8, CYP4Z1, DAAM1, DAB2, DAD1, DCC, DCHS2, DGKB, DGKH, DIO1, DISC1, DLG2, DLGAP1, DMBT1, DNAAF1, DNER, DNM1L, DOCK1, DOCK2, DOCK4, DOPEY2, DRAM1, DSC1, DSCAM, DSG1, DSG3, DTNA, DYM, DYNC2H1, DYSF, DYTN, EGFR, ELMO1, ELOVL2, ELOVL6, ELOVL7, ELTD1, EMCN, EMID2, EMR2, ENDOU, ENOX1, ENPP1, ENPP6, EPHA7, EPHB1, ERAP1, ERAP2, ERBB4, ERC2, ERO1LB, ERVFRD-1, ESR1, ESYT2, EXOC2, EXT1, EZR, F2RL2, F5, FABP2, FAS, FAT2, FAT3, FAT4, FCGR2C, FHIT, FLVCR1, FMN1, FMO2, FNDC1, FRAS1, FREM2, FRMPD1, FSHR, FUT9, FZD6, GAA, GABBR1, GABBR2, GABRB2, GABRG3, GABRR1, GALNT10, GALNT13, GALNT2, GALNTL4, GALNTL6, GAS2, GFRAL, GNAL, GPC5, GPC6, GPHN, GPR111, GPR116, GPR139, GPR146, GPR158, GPR39, GPR65, GPR78, GRB10, GRIA1, GRID2, GRIK2, GRIK3, GRIK4, GRIN2A, GRIN3A, GRIP1, GRIP2, GRM5, GRM7, GRM8, GUCA1A, HAAO, HADH, HCRTR2, HHAT, HIP1, HK1, HLA-A, HLA-B, HLA-C, HLA-DOA, HLA-DPA1, HLA-DPB1, HLA-DQA1, HLA-DQA2, HLA-DQB1, HLA-DQB2, HLA-DRA, HLA-DRB1, HLA-DRB5, HLA-F, HLA-G, HMGCLL1, HPSE, HPSE2, HS3ST2, HS3ST4, HTR1E, IGSF5, INADL, INPP5D, INSR, ITGA1, ITGA11, ITGA2, ITGA9, ITGAE, ITGB5, ITPR1, ITPR2, IYD, KAZN, KCNA6, KCNAB1, KCNB2, KCNH7, KCNIP4, KCNJ12, KCNJ15, KCNJ6, KCNK10, KCNK2, KCNMA1, KCNMB2, KCNMB3, KCNQ3, KCNQ5, KIAA0319, KIAA0528, KIF13A, KIF16B, KIRREL3, KITLG, KL, KLHL14, KLRB1, KNG1, KSR2, LAMA2, LAMP3, LEPR, LGR6, LHCGR, LOXL2, LPGAT1, LPP, LRFN2, LRRC1, LRRC4C, LRRC52, LRRK2, LRRTM4, LSAMP, LYN, LYPD6B, MAGI1, MAGI2, MAN1A1, MAN2A1, MAPT, MARCH1, MARCH4, MBP, MCC, MDGA2, MGAT1, MGAT5, MGAT5B, MGST3, MPP5, MRAP2, MRGPRX4, MRS2, MRTO4, MTCH1, MTHFS, MTUS1, MUC16, MYH9, MYO10, MYO16, MYO1B, MYO5B, MYO7A, MYOF, NAV3, NBEA, NCAM2, NCS1, NDST3, NDST4, NDUFA12, NDUFB1, NDUFB10, NDUFB4, NEDD4, NETO1, NFASC, NKAIN1, NKAIN3, NLGN1, NMNAT2, NMUR1, NMUR2, NOTCH4, NPHS2, NPLOC4, NPSR1, NRCAM, NRG1, NRP1, NRXN1, NSL1, NTM, NTN4, NTRK1, NUP210, NUP35, OCA2, ODZ3, OMA1, OPCML, OPRK1, OR10AD1, OR10AG1, OR10C1, OR12D2, OR13C2, OR13C5, OR14A16, OR1L8, OR1N2, OR2AK2, OR2B11, OR2J2, OR2L13, OR2T4, OR2W3, OR4C3, OR4C45, OR51B2, OR51B6, OR51E1, OR51F1, OR51I2, OR51L1, OR51M1, OR51Q1, OR52D1, OR52E2, OR52E6, OR52J3, OR52N2, OR52N4, OR5AC2, OR5F1, OR5I1, OR5P2, OR5W2, OR8G1, OR8H2, OR8H3, OR8I2, OR8U8, OR9G1, OR9G9, OR9K2, OR9Q1, PAK2, PALM2, PAPPA2, PAQR8, PARD3, PARD3B, PCDH15, PCDH17, PCDH9, PCDHA12, PCDHA13, PCDHA9, PCLO, PDE4D, PDGFA, PDGFD, PDIA6, PEX5L, PGAP3, PGLYRP4, PGS1, PHLDB2, PIGG, PIK3C2B, PIK3C2G, PINK1, PKD1L1, PKDREJ, PKP1, PKP2, PLA2G4C, PLA2G4D, PLA2G4E, PLA2G6, PLA2R1, PLAUR, PLCB1, PLCG2, PLCH1, PLD1, PLOD2, PLSCR1, PLXNA4, PNPLA3, POMT1, PPAP2A, PPP1R14C, PPP2CA, PPP3CA, PPYR1, PREX1, PRICKLE1, PRICKLE2, PRKCA, PRKCB, PRKCE, PRKG1, PROKR2, PROM1, PSD3, PSTPIP2, PTGER3, PTGS1, PTPN13, PTPRN2, PTPRT, PTPRU, PYGL, RAB12, RAB17, RAB27A, RAB31, RAB36, RAB3C, RAC2, RAMP3, RAPGEF4, RECK, RFTN1, RGS3, RGS6, RGS7, RIMBP2, RIMS2, RNF144B, RNPEP, ROBO1, RPSA, RRP12, RTKN2, RYR1, RYR2, RYR3, SBF2, SCARA3, SCARB1, SCD5, SCGN, SCN3B, SDF4, SDHA, SEC22A, SEC24D, SELE, SEMA3A, SEMA3E, SEMA5A, SEMA6D, SERINC5, SGCG, SGCZ, SGIP1, SH3GL2, SIPA1L1, SIRPA, SIRT3, SKAP2, SLC10A2, SLC12A6, SLC14A1, SLC14A2, SLC15A2, SLC16A14, SLC17A5, SLC19A3, SLC1A2, SLC1A6, SLC22A16, SLC22A5, SLC24A2, SLC24A4, SLC25A21, SLC25A25, SLC25A37, SLC26A9, SLC27A6, SLC28A1, SLC28A2, SLC28A3, SLC2A9, SLC35B3, SLC35D2, SLC41A3, SLC7A7, SLC8A1, SLC9A4, SLC9A8, SLC9A9, SLC9C1, SLCO1B1, SLCO1B3, SLCO5A1, SLCO6A1, SNCA, SNTG2, SNX19, SNX24, SNX25, SNX5, SNX7, SNX9, SOAT1, SOAT2, SORCS1, SORCS2, SORCS3, SPCS3, SPINK5, SPPL2C, SPRED1, SPRED3, SPTLC3, SQRDL, SSR1, ST3GAL6, ST8SIA6, STARD13, STT3A, STYK1, SVEP1, SVIL, SWAP70, SYBU, SYK, SYN3, SYNE1, SYNPR, SYT1, SYT6, SYT9, TACSTD2, TANC1, TAP1, TAP2, TAS1R2, TEC, TECTA, TEK, TES, TESC, THSD7A, TIAM1, TMEM106B, TMEM163, TOR1A, TPO, TRPC4, TRPC6, TSHR, TSPAN9, TTLL10, TULP3, TYMS, UGT2B7, UNC13A, UNC13C, UNC5C, UNC93A, UST, VAV1, VAV2, VAV3, VNN1, VOPP1, VPS37C, WBSCR17, WLS, WNK2, WWOX, XXYLT1, XYLT1, XYLT2, ZFYVE28, ZW10*
Cell junction	4.9×10^{-11}	2.0	*ABCB5, ABI1, AFAP1, AJAP1, AMPH, AMTN, ANK2, ANK3, ANKS1B, APBB1IP, ARHGAP24, ARHGEF18, BSPRY, CADM2, CADPS, CADPS2, CCDC85C, CDC42BPA, CDHR2, CDSN, CHRM2, CHRNB3, CLDN1, CLDN10, CLDN14, COL13A1, CTNNA2, CTNNA3, CTNND2, CXADR, DISC1, DLC1, DLG2, DLGAP1, DSC1, DSG1, DSG3, DTNA, EPHX2, ERC2, FAT2, FER, FMN1, FNDC1, FRMD4A, GABBR1, GABRB2, GABRG3, GABRR1, GPHN, GRIA1, GRID2, GRIK2, GRIK3, GRIN2A, GRIP1, IGSF5, INADL, IQSEC1, ITGB5, KAZN, LAMA1, LPP, LRFN2, MAGI2, MPP5, MYH9, NCS1, NETO1, NFASC, NFIA, NRXN1, PARD3B, PCLO, PHACTR1, PKP1, PKP2, PLEKHA7, POLR1E, PSD3, PTPRM, PTPRU, RIMBP2, RIMS2, SEPT11, SHISA9, SLC8A1, SNCA, SYN3, SYNPR, SYT9, TANC1, TEK, TES, TIAM1, TMEM163, TNS1, TRIM9, TRPC4, TRPC6, UNC13C*
MHC class II protein complex	2.8×10^{-10}	86.9	*HLA-DPA1, HLA-DPB1, HLA-DQA1, HLA-DQB1, HLA-DRA, HLA-DRB1*

GO categories in which false discovery rate is less than 0.01.

Table S26 continued.

Description	p-value	Enrichment	Genes
Plasma membrane part	1.7×10^{-9}	1.5	*ABCA1, ABCA4, ABCB5, ABCG8, ADAM29, ADCY3, AJAP1, ALK, ANK2, ANK3, ARHGEF4, ART3, ASIC2, ATP12A, ATP2B4, ATP6V0A4, BBS9, BMPR1B, C8A, CACNA1A, CACNA1C, CAMK2D, CASQ2, CASR, CATSPERB, CDH13, CDH4, CDHR2, CHRM2, CHRNB3, CLCNKB, CLDN1, CLEC1A, CLEC1B, CNR2, CNTNAP2, CTNNA2, CUBN, CX-ADR, CXCL12, CYBRD1, CYP4F12, CYP4F2, DAB2, DCC, DDR1, DISP1, DLC1, DSCAM, DSG1, DYSF, EMR1, ENPP1, EPHA6, EPHA7, EPHB1, ERBB4, EVC, EVC2, FAS, FCER2, FER, FGD5, FLT3, FLVCR1, GABBR1, GABRB2, GABRR1, GOLM1, GPC5, GPC6, GPR39, GRIA1, GRID2, GRIK2, GRIK3, GRIN2A, GRM5, GRM7, GRM8, HLA-A, HLA-B, HLA-C, HLA-DOA, HLA-DPA1, HLA-DPB1, HLA-DQA1, HLA-DQA2, HLA-DQB1, HLA-DQB2, HLA-DRA, HLA-DRB1, HLA-DRB5, HLA-G, IL12RB2, IL1RL2, INADL, ITGA1, ITGA2, ITGB5, ITGBL1, KANK1, KCNA6, KCNK2, KCNMB2, KCNMB3, KCNQ3, KCNQ5, KL, LGR5, LHCGR, LYN, MEGF10, MEGF11, MUC12, MYH9, MYO10, MYOF, NEDD4, NMUR1, NOTCH4, NOXO1, NRCAM, NRG1, NRG3, NRXN1, OPCML, OPRK1, PCDHA1, PCDHA10, PCDHA11, PCDHA2, PCDHA3, PCDHA4, PCDHA5, PCDHA6, PCDHA7, PCDHA8, PCD-HAC1, PCSK9, PKHD1, PLA2R1, PLAUR, PLEK, PLSCR1, PPYR1, PRKAB2, PTPRB, PT-PRD, PTPRF, PTPRM, PTPRU, RAB27A, RAMP3, RASAL1, RGS6, RGS7, RNPEP, ROBO2, SCARB1, SCN3B, SCNN1G, SELE, SGCG, SGCZ, SHISA9, SLC10A2, SLC12A6, SLC14A1, SLC14A2, SLC15A2, SLC17A5, SLC1A2, SLC28A1, SLC2A9, SLC5A12, SLC7A7, SLC8A1, SLC9A4, SLC9C1, SLCO1B1, SLCO1B3, SNTG1, SNTG2, SNX5, SNX9, SPATA13, SPRED1, SPTB, STK39, SYK, TEK, TMEM17, TNFRSF11A, TPO, TRHDE, TRPA1, TRPC4, TSHR, TSPAN9, VWF, WWC1*
Integral to endoplasmic reticulum membrane	2.2×10^{-9}	31.8	*HLA-A, HLA-B, HLA-C, HLA-DPA1, HLA-DPB1, HLA-DQA1, HLA-DQB1, HLA-DRB1*
Cytoplasmic vesicle membrane	6.3×10^{-9}	42.3	*CPE, DMBT1, HLA-A, HLA-C, HLA-DPA1, HLA-DPB1*
Cell projection part	6.5×10^{-9}	1.82	*ABI1, ALS2, AMPH, ANK3, ANKS1B, APBB2, APP, ARHGEF4, ATP6V0A4, ATP8B1, BBS9, CACNA1C, CHRM2, CNTNAP2, CTNND2, CUBN, CXADR, CYBRD1, DCC, DFNB31, DISC1, DLG2, DLGAP1, DNAAF1, DNAH11, DNAH12, DNAH14, DNAH5, DNAH8, DSCAM, DYNC2H1, EMR2, ERBB4, ERC2, ESR1, EVC, EVC2, EZR, FGD5, FOPNL, FSCB, FZD6, GABBR1, GRIA1, GRID2, GRIK2, GRIK3, GRIK4, GRIN2A, GRIN3A, GRM5, GRM7, HAP1, IFT57, KANK1, KCNQ3, KIF5C, KIRREL3, LRRK2, LYN, MAGI2, MAP2, MAPT, MYO10, MYO5A, MYO5B, NCS1, NETO1, NFASC, NLGN1, NRCAM, NRP1, NRXN1, PACRG, PKD1L1, PKHD1, PLCB4, PLEK, PROM1, PSD3, PTPRF, PTPRN2, RAPGEF4, ROBO1, ROBO2, RUFY3, SCARB1, SCN1A, SEPT11, SHISA9, SIPA1L1, SLC1A2, SLC8A1, SLC9C1, SNCA, SNTG1, SPAG16, SPATA13, SYNJ2, TANC1, TEKT1, TEKT4, TIAM2, TMEM17, TTLL11, WWC1*
Clathrin-coated endocytic vesicle membrane	8.0×10^{-9}	55.3	*HLA-DPA1, HLA-DPB1, HLA-DQA1, HLA-DQB1, HLA-DRA, HLA-DRB1*
Vesicle membrane	8.8×10^{-9}	40.6	*CPE, DMBT1, HLA-A, HLA-C, HLA-DPA1, HLA-DPB1*
Intrinsic to endoplasmic reticulum membrane	9.4×10^{-9}	26.8	*HLA-A, HLA-B, HLA-C, HLA-DPA1, HLA-DPB1, HLA-DQA1, HLA-DQB1, HLA-DRB1*
Cell projection	9.8×10^{-9}	2.6	*ACPP, ADCY5, APBB1IP, APBB2, APOA1BP, ARHGAP24, ARPC5, BMPR1B, CACNA1A, CADM2, CATSPERB, CNGA3, CNTN4, CNTNAP2, CTNNA3, DYNC2H1, FAM65B, FGD5, FOPNL, FRMD4B, GRIA1, GRIP1, IFT57, IQGAP2, ITGA1, KALRN, KCNIP4, KIRREL3, KLHL1, KLHL14, KLHL24, LDHC, LRRC16A, MAPT, MYO10, MYO1B, MYO3A, MYO5B, MYRIP, NRCAM, PKHD1, PTPRM, RELN, RPTOR, SEMA3A, SEPT11, SLC9C1, SPATA13, SWAP70, SYNPR, WWOX*
Coated vesicle membrane	1.5×10^{-8}	25.3	*HLA-A, HLA-B, HLA-C, HLA-DPA1, HLA-DPB1, HLA-DQA1, HLA-DQB1, HLA-DRB1*
Trans-Golgi network membrane	3.3×10^{-8}	45.1	*HLA-DPA1, HLA-DPB1, HLA-DQA1, HLA-DQB1, HLA-DRA, HLA-DRB1*
Cytoplasmic vesicle part	3.5×10^{-8}	32.6	*CPE, DMBT1, HLA-A, HLA-C, HLA-DPA1, HLA-DPB1*
Phagocytic vesicle membrane	2.6×10^{-7}	124.3	*DMBT1, HLA-A, HLA-B, HLA-C*
Integral to organelle membrane	2.7×10^{-7}	15.4	*HLA-A, HLA-B, HLA-C, HLA-DPA1, HLA-DPB1, HLA-DQA1, HLA-DQB1, HLA-DRB1*
Intrinsic to organelle membrane	6.1×10^{-7}	13.7	*HLA-A, HLA-B, HLA-C, HLA-DPA1, HLA-DPB1, HLA-DQA1, HLA-DQB1, HLA-DRB1*
Synapse	2.5×10^{-6}	2.3	*ABI1, APBB2, APP, CADM2, CADPS, CAMK2D, CHRM2, CPLX1, CXADR, DFNB31, EGFLAM, EPHA7, GRID2, GRIN3A, GRM7, INSR, MAGI2, MYH9, MYO7A, MYRIP, NETO1, NLGN1, NRCAM, NRG1, NRXN1, NTM, PCDH15, PCLO, PDZRN3, PHACTR1, PTPRF, RAPGEF4, RIMBP2, SEPT11, SERPINE2, SHISA9, SNCA*
Synapse part	6.1×10^{-6}	1.8	*ALS2, AMPH, ANK2, ANK3, ANKS1B, CACNA1C, CADPS2, CHRM2, CHRNB3, CLSTN2, CNTN2, CTNND2, DDC, DISC1, DLG2, DLGAP1, DPYSL2, EPHA7, ERBB4, ERC2, ESR1, GABBR1, GABBR2, GABRB2, GABRG3, GABRR1, GPHN, GRIA1, GRID2, GRIK2, GRIK3, GRIK4, GRIN2A, GRIN3A, GRIP1, GRM5, GRM7, GRM8, LRFN2, LRRK2, LRRTM4, LYN, MAGI2, NCS1, NETO1, NLGN1, NRXN1, PCLO, PLCB4, PSD3, PTPRN2, RAB3C, RIMS2, SIPA1L1, SLC1A2, STON2, SYN3, SYNE1, SYNPR, SYT1, SYT6, SYT9, TANC1, TMEM163, TRIM9, UNC13A, UNC13C*
Synaptic membrane	7.1×10^{-6}	2.4	*ANK2, ANK3, ANKS1B, CADPS2, CHRM2, CHRNB3, CLSTN2, DISC1, DLG2, DLGAP1, EPHA7, ERC2, GABBR1, GABRB2, GABRG3, GABRR1, GPHN, GRIA1, GRID2, GRIK2, GRIK3, GRIN2A, GRIP1, GRM7, GRM8, LRFN2, NCS1, NETO1, NRXN1, PSD3, RIMS2, SLC1A2, SYNE1, TANC1, UNC13C*
MHC class I protein complex	9.2×10^{-6}	135.2	*HLA-A, HLA-B, HLA-C*
Endosome membrane	1.1×10^{-5}	9.6	*HLA-A, HLA-B, HLA-C, HLA-DPA1, HLA-DPB1, HLA-DQA1, HLA-DQB1, HLA-DRB1*

GO categories in which false discovery rate is less than 0.01.

Table S26 continued.

Description	p-value	Enrichment	Genes
Extracellular matrix part	1.3×10^{-5}	2.2	*ADAMTS1, AMTN, C1QTNF3, CCBE1, COL13A1, COL21A1, COL23A1, COL24A1, COL27A1, COL4A1, COL4A2, COL4A3, COL5A1, COL5A2, COL9A1, COLEC12, EGFLAM, EMID1, EMID2, FBN2, FREM2, LAMA1, LAMA2, LAMC1, LAMC2, LEPREL1, LOXL2, MATN2, MBL2, NID2, NTN1, NTN4, SCARA3, SNCA, THBS2, THSD4, TIMP2, TNR*
Endosomal part	1.6×10^{-5}	9.1	*HLA-A, HLA-B, HLA-C, HLA-DPA1, HLA-DPB1, HLA-DQA1, HLA-DQB1, HLA-DRB1*
Cell-cell junction	1.6×10^{-5}	2.3	*5), ABCB5, AMTN, ANK2, ANK3, CCDC85C, CDC42BPA, CDSN, CLDN1, CLDN10, CLDN14, COL13A1, CTNNA3, DSC1, DSG1, DSG3, FAT2, FNDC1, FRMD4A, IGSF5, IN-ADL, KAZN, LAMA1, MAGI2, MPP5, MYH9, PKP1, PKP2, PLEKHA7, PTPRM, PTPRU, SLC8A1, TEK, TIAM1, TRPC4, TRPC6*
Intrinsic to plasma membrane	2.0×10^{-5}	1.9	*ABCA1, ABCA4, ABCB5, ADCY3, ALK, ANK2, ART3, BMPR1B, CDH4, CNR2, EMR1, FCER2, FLVCR1, GPC5, GPC6, GPR39, GRIK2, GRIN2A, HLA-A, HLA-B, HLA-C, HLA-DPA1, HLA-DQA1, HLA-DQA2, HLA-DRA, HLA-DRB1, IL1RL2, KCNMB2, KL, LHCGR, MUC12, NOTCH4, NRCAM, NRG3, NRXN1, OPCML, OPRK1, PCDHA1, PCDHA10, PCDHA11, PCDHA2, PCDHA3, PCDHA4, PCDHA5, PCDHA6, PCDHA7, PCDHA8, PCD-HAC1, PKHD1, PLSCR1, PPYR1, PTPRB, PTPRD, PTPRM, RAMP3, SLC15A2, SLC2A9, SLCO1B1, SLCO1B3, SPTB, TEK, TSHR*
Integral to plasma membrane	2.0×10^{-5}	1.7	*ABCA1, ABCA4, ABCB5, ADCY3, ALK, ANK2, ART3, ATP2B4, BMPR1B, CDH4, CHRM2, CLCNKB, CLDN1, CLEC1A, CNR2, DDR1, EMR1, EPHB1, FCER2, FLT3, FLVCR1, GABRR1, GPC5, GPC6, GPR39, GRID2, GRIK2, GRIN2A, GRM5, GRM8, HLA-A, HLA-B, HLA-C, HLA-DPA1, HLA-DQA1, HLA-DQA2, HLA-DRA, HLA-DRB1, IL1RL2, KCNMB2, KCNMB3, KL, LHCGR, MUC12, NOTCH4, NRCAM, NRG3, NRXN1, OPCML, OPRK1, PCDHA1, PCDHA10, PCDHA11, PCDHA2, PCDHA3, PCDHA4, PCDHA5, PCDHA6, PCDHA7, PCDHA8, PCDHAC1, PLA2R1, PLSCR1, PPYR1, PTPRB, PTPRD, PTPRM, PT-PRU, RAMP3, SCARB1, SLC15A2, SLC17A5, SLC2A9, SLCO1B1, SLCO1B3, TEK, TPO, TRPA1, TSHR*
Cytoskeleton	3.1×10^{-5}	1.6	*ABI1, ABLIM1, ACTA2, ACTL8, ACTR2, ADH4, AFAP1, AKAP12, AMPH, ANK2, ANK3, APBB1IP, ARHGAP24, ARPC5, BICD1, CABP5, CAP1, CDSN, CORO2B, COTL1, CTNNA2, CTNNA3, CTTNBP2NL, DAPK1, DDX20, DOCK2, ELMO1, ERC2, EZR, FAM65B, FER, FGD5, FHOD3, FMN1, FRMD4A, FRMD4B, FRMPD1, GPHN, GYS2, HAP1, INPP5D, IQGAP2, KALRN, KAZN, KITLG, KLHL1, KLHL3, KLHL5, LDB3, LRRFIP1, MAD1L1, MLPH, MTSS1, MYH9, MYO9B, MYOZ2, MYRIP, NME8, NUP35, PCLO, PDLIM1, PHLDB2, PLEKHH2, PPP2CA, PRKCE, PSTPIP2, PTPN13, PTPN14, PTPN21, SELE, SGCG, SGCZ, SHROOM3, SLC30A9, SMARCA2, SNCA, SNTG1, SNTG2, SORBS2, SPRR1B, SPTB, STK39, SVIL, SYBU, SYNE1, TBCA, TEC, TNS1, TRIM9, TRPC4, UBXN11*
Clathrin-coated vesicle membrane	4.6×10^{-5}	14.3	*HLA-DPA1, HLA-DPB1, HLA-DQA1, HLA-DQB1, HLA-DRA, HLA-DRB1*
Neuron projection	7.2×10^{-5}	1.7	*ALS2, ANKS1A, APP, BMPR1B, CACNA1A, CADM2, CDH13, CHL1, CHRM2, CNR2, CNTN4, CNTNAP2, CPLX1, CTNNA2, CXADR, DCC, DDC, DFNB31, DNER, DSCAM, EPHA7, EPHB1, GABBR1, GABBR2, GRIA1, GRID2, GRIK3, GRIK4, GRIN3A, GRIP1, GRM7, GRM8, HAP1, ITGA1, KALRN, KCNIP4, KCNJ6, KIF5C, KIRREL3, KLHL1, KLHL14, KLHL24, LRRK2, MAPT, MYO10, MYO5A, MYO5B, NCAM2, NCS1, NLGN1, NOV, NRCAM, NTM, ODZ4, PEX5L, PRKCA, PTPN13, RAB27A, RAPGEF4, RELN, RP-TOR, SEMA3A, SEPT11, SIPA1L1, SLC1A2, SLC8A1, SNCA, SYNPR, SYT1, TRIM9*
Extracellular matrix	7.3×10^{-5}	1.9	*ABI3BP, ADAMTS12, ADAMTS14, ADAMTS16, ADAMTS18, ADAMTS20, ADAMTSL1, ADAMTSL3, AMTN, CDON, CHL1, COL4A1, COL4A2, COL5A1, COL5A2, DMBT1, DPT, EFEMP1, EPYC, FBN2, FGF1, FRAS1, GPC5, GPC6, GPLD1, HPSE2, IGFBP7, LAMA1, LAMA2, LAMC1, LTBP1, MATN2, MEPE, MMP20, NAV2, NID2, NOV, PLSCR1, PXDN, RELN, SERPINE2, SPARCL1, SPOCK3, SPON1, TECTA, TNN, TNR, VIT, VWF*
Vacuolar membrane	8.0×10^{-5}	8.5	*CUBN, HLA-DPA1, HLA-DPB1, HLA-DQA1, HLA-DQB1, HLA-DRA, HLA-DRB1*
Lysosomal membrane	8.2×10^{-5}	10.1	*CUBN, HLA-DPA1, HLA-DPB1, HLA-DQA1, HLA-DQB1, HLA-DRA, HLA-DRB1*
Postsynaptic membrane	8.8×10^{-5}	2.1	*ANK2, ANK3, ANKS1B, CADPS2, CHRM2, CHRNB3, CLSTN2, DISC1, DLG2, DLGAP1, EPHA7, GABBR1, GABBR2, GABRB2, GABRG3, GABRR1, GPHN, GRIA1, GRID2, GRIK2, GRIK3, GRIK4, GRIN2A, GRIN3A, GRIP1, GRM7, LRFN2, LRRTM4, NCS1, NETO1, NLGN1, PSD3, SIPA1L1, SYNE1, TANC1*
Proteinaceous extracellular matrix	1.3×10^{-4}	2.0	*ABI3BP, ADAMTS12, ADAMTS14, ADAMTS16, ADAMTS18, ADAMTS20, ADAMTSL1, ADAMTSL3, AMTN, CHL1, DMBT1, DPT, EFEMP1, EPYC, FBN2, FGF1, GPC5, GPC6, GPLD1, HPSE2, LAMA1, LTBP1, MATN2, MEPE, MMP20, NAV2, PXDN, RELN, SPARCL1, SPOCK3, SPON1, TECTA, TNN, TNR, VIT, VWF*
Sarcolemma	1.6×10^{-4}	11.2	*ADCY5, ANK2, ANK3, LAMA2, SGCG, SGCZ*
Vacuolar part	2.1×10^{-4}	6.3	*CUBN, GPC5, HLA-DPA1, HLA-DPB1, HLA-DQA1, HLA-DQB1, HLA-DRA, HLA-DRB1*
Golgi membrane	3.2×10^{-4}	17.3	*HLA-A, HLA-C, HLA-DPA1, HLA-DPB1*

GO categories in which false discovery rate is less than 0.01.
