# Supplementary material for: A Model-Based Approach for Identifying Signatures of Ancient Balancing Selection in Genetic Data
Source: PLoS Genet. 2014 Aug 21;10(8):e1004561. doi: 10.1371/journal.pgen.1004561 (PMC4140648; doi:10.1371/journal.pgen.1004561)
Supplement: Table S27 — GO component analysis of ranked signals from YRI population using the test statistic. (PDF) [file pgen.1004561.s053.pdf]

Table S27: GO component analysis of ranked signals from YRI population using the T_2 test statistic.

Description	p-value	Enrichment	Genes
Membrane part	1.9×10^{-13}	1.3	*AADAC*, *ABCA1*, *ABCA12*, *ABCA13*, *ABCA4*, *ABCB11*, *ABCB5*, *ABCC4*, *ABCC8*, *ABCD4*, *ABHD2*, *ABHD6*, *ABO*, *ACBD5*, *ACPP*, *ADAM12*, *ADAM28*, *ADAM29*, *ADAM32*, *ADCY5*, *ADCY8*, *ADRA1A*, *AGMO*, *AGPAT5*, *AKAP6*, *ALG8*, *ALK*, *ALPL*, *ANGPT1*, *ANK2*, *ANK3*, *ANKH*, *ANO2*, *ANO3*, *ANO6*, *AP1S3*, *ART3*, *ASGR2*, *ASIC2*, *ASTN2*, *ATP10A*, *ATP10B*, *ATP10D*, *ATP13A5*, *ATP1A2*, *ATP2C2*, *ATP6V0A4*, *ATP6V0E2*, *ATP8A1*, *ATP8A2*, *ATRNL1*, *BAI3*, *BEST3*, *BMPR1B*, *BRI3BP*, *BST1*, *BTBD11*, *BTN3A1*, *BTNL2*, *BTNL3*, *C1GALT1*, *CACNA1A*, *CACNA1C*, *CACNA2D1*, *CACNA2D3*, *CADM2*, *CALCR*, *CALN1*, *CAMK2D*, *CAPN3*, *CASQ2*, *CATSPERB*, *CD200*, *CD81*, *CD84*, *CDH10*, *CDH12*, *CDH13*, *CDH23*, *CDH4*, *CDH9*, *CDKAL1*, *CDON*, *CELSR2*, *CHODL*, *CHRM5*, *CHRNB3*, *CHST11*, *CHST8*, *CHST9*, *CLCA2*, *CLCNKB*, *CLDN10*, *CLDN14*, *CLDN23*, *CLEC12A*, *CLEC1A*, *CLEC4D*, *CLEC6A*, *CLECL1*, *CLMN*, *CLSTN2*, *CMTM8*, *CNGA3*, *CNIH3*, *CNR2*, *CNTN1*, *CNTN2*, *CNTN4*, *CNTN5*, *CNTN6*, *CNTNAP2*, *CNTNAP4*, *CNTNAP5*, *COL23A1*, *COL25A1*, *COLEC12*, *COPA*, *CPLX1*, *CSMD1*, *CSMD2*, *CSMD3*, *CTNNA2*, *CTNS*, *CUBN*, *CUX1*, *CXADR*, *CYB5A*, *CYBRD1*, *CYP2E1*, *CYP2U1*, *CYP39A1*, *CYP3A43*, *CYP4F11*, *CYP4F12*, *CYP4F3*, *CYP4F8*, *CYP4Z1*, *DAD1*, *DCC*, *DCHS2*, *DDR2*, *DIO1*, *DLC1*, *DMBT1*, *DNER*, *DPP6*, *DRAM1*, *DSC1*, *DSC3*, *DSCAM*, *DSG1*, *DSG3*, *DYSF*, *EGFR*, *ELFN2*, *ELOVL6*, *ELTD1*, *EMR1*, *EMR2*, *ENPP1*, *EPB41L4A*, *EPHA3*, *EPHA6*, *ERAP1*, *ERAP2*, *ERBB4*, *ERO1LB*, *ESR1*, *ESYT2*, *EVC*, *EVC2*, *EXT1*, *FAM155A*, *FAM189A1*, *FAT2*, *FAT3*, *FCAR*, *FCER2*, *FER*, *FER1L6*, *FGD5*, *FLNB*, *FLOT1*, *FLT3*, *FLVCR1*, *FMO2*, *FMO4*, *FMO5*, *FNDC9*, *FRAS1*, *FRRS1*, *FSHR*, *FUT10*, *FUT9*, *GABBR2*, *GABRB3*, *GABRG3*, *GABRR1*, *GALNT10*, *GALNT2*, *GALNTL4*, *GALNTL5*, *GALNTL6*, *GGA1*, *GGT1*, *GLDN*, *GLRA3*, *GNG2*, *GNG4*, *GP5*, *GPC5*, *GPC6*, *GPM6A*, *GPNMB*, *GPR111*, *GPR115*, *GPR137B*, *GPR158*, *GPR39*, *GRAMD1A*, *GRIA1*, *GRID1*, *GRID2*, *GRIK1*, *GRIK2*, *GRIK4*, *GRIN2A*, *GRIP1*, *GRM1*, *GRM5*, *GRM7*, *GRM8*, *GZMB*, *HHAT*, *HHLA2*, *HK1*, *HLA-A*, *HLA-B*, *HLA-C*, *HLA-DOB*, *HLA-DPA1*, *HLA-DPB1*, *HLA-DQA1*, *HLA-DQA2*, *HLA-DQB1*, *HLA-DQB2*, *HLA-DRA*, *HLA-DRB1*, *HLA-DRB5*, *HLA-F*, *HLA-G*, *HMGCLL1*, *HS3ST2*, *HS3ST4*, *IER3*, *IFI27L1*, *IGSF5*, *IL12RB2*, *IL1R2*, *IL1RAP*, *IL1RL1*, *IL1RL2*, *IL28RA*, *IMMP2L*, *INADL*, *IQGAP3*, *ITGA1*, *ITGA11*, *ITGA2*, *ITGA6*, *ITGA8*, *ITGA9*, *ITGAE*, *ITGB3*, *ITGB5*, *ITGBL1*, *KANK1*, *KCNA6*, *KCNAB1*, *KCNC2*, *KCND3*, *KCNH7*, *KCNH8*, *KCNJ12*, *KCNJ6*, *KCNK10*, *KCNK18*, *KCNK2*, *KCNMB2*, *KCNMB3*, *KCNQ5*, *KCNV2*, *KIAA0319*, *KIAA0922*, *KIAA1244*, *KIAA1324L*, *KIRREL*, *KIRREL3*, *KL*, *KLHL14*, *KLRB1*, *LAMP3*, *LAPTM4B*, *LARGE*, *LCT*, *LDLRAD3*, *LGR5*, *LHFP*, *LHFPL2*, *LHFPL3*, *LPHN3*, *LPPR1*, *LPPR5*, *LRFN5*, *LRP1B*, *LRP8*, *LRPPRC*, *LRRC4C*, *LRRC52*, *LRRTM4*, *LYPD6B*, *MAEA*, *MARCH1*, *MARCH4*, *MCTP2*, *MDGA2*, *MEGF11*, *MGAM*, *MGAT1*, *MGAT5*, *MGAT5B*, *MGST3*, *MOGAT2*, *MRGPRX4*, *MS4A12*, *MSR1*, *MTCH1*, *MUC12*, *MUC16*, *MUC22*, *MX1*, *MYH9*, *MYO10*, *MYOF*, *NAALADL2*, *NALCN*, *NAV3*, *NCALD*, *NCAM2*, *NDST4*, *NDUFB1*, *NEDD4*, *NETO1*, *NFAM1*, *NFASC*, *NKAIN1*, *NKAIN3*, *NLGN1*, *NOTCH2*, *NOTCH4*, *NPFFR2*, *NPR3*, *NPSR1*, *NRCAM*, *NRG1*, *NRG3*, *NRXN1*, *NRXN3*, *NTM*, *NTRK2*, *NTRK3*, *NUP88*, *OCA2*, *ODZ3*, *ODZ4*, *OMA1*, *OPCML*, *OPN3*, *OPRK1*, *OR10C1*, *OR12D2*, *OR13C5*, *OR14A16*, *OR1A2*, *OR1N2*, *OR2AK2*, *OR2B11*, *OR2L13*, *OR2L8*, *OR2W3*, *OR4C3*, *OR4C45*, *OR51B6*, *OR51E1*, *OR51F1*, *OR51L1*, *OR51M1*, *OR51Q1*, *OR52B6*, *OR52E2*, *OR52E4*, *OR52E6*, *OR52J3*, *OR52R1*, *OR5B3*, *OR5P2*, *OR6K6*, *OR9K2*, *OSTM1*, *OTOP1*, *PCDH15*, *PCDHA1*, *PCDHA10*, *PCDHA11*, *PCDHA12*, *PCDHA13*, *PCDHA2*, *PCDHA3*, *PCDHA4*, *PCDHA5*, *PCDHA6*, *PCDHA7*, *PCDHA8*, *PCDHA9*, *PCDHAC1*, *PCNXL2*, *PCSK5*, *PCSK9*, *PDE3A*, *PDIA6*, *PEX5L*, *PGAP3*, *PGBD5*, *PIEZO2*, *PIGG*, *PIGL*, *PIKFYVE*, *PKD1L1*, *PKD1L2*, *PKDREJ*, *PKHD1*, *PKHD1L1*, *PLA2G4C*, *PLA2R1*, *PLB1*, *PLD1*, *PLD5*, *PLEKHH2*, *PLOD2*, *PLSCR1*, *PLXDC2*, *PLXNA4*, *PNPLA3*, *PNPLA6*, *PQLC2*, *PRKAB2*, *PRKCA*, *PRKD1*, *PRKG2*, *PRLR*, *PTCH1*, *PTCHD3*, *PTGER3*, *PTGFRN*, *PTGS1*, *PTPRB*, *PTPRD*, *PTPRH*, *PTPRM*, *PTPRN2*, *PTPRQ*, *PTPRT*, *RAB27A*, *RAMP3*, *RAPGEF4*, *RECK*, *RGS6*, *RGS7*, *RGSL1*, *RNF144B*, *RNF150*, *RNFT2*, *RNPEP*, *ROBO2*, *ROR1*, *RRP12*, *RYR2*, *RYR3*, *SCARA5*, *SCARB1*, *SCD5*, *SCN11A*, *SCN1A*, *SCN3A*, *SCN3B*, *SCN9A*, *SCNN1G*, *SCTR*, *SCUBE1*, *SDHA*, *SDHC*, *SDK1*, *SEMA5A*, *SEMA6D*, *SERINC5*, *SERPINE2*, *SGCG*, *SGCZ*, *SGIP1*, *SHISA2*, *SHISA6*, *SHISA9*, *SHROOM3*, *SIRPA*, *SLC12A3*, *SLC12A6*, *SLC12A8*, *SLC14A2*, *SLC15A2*, *SLC15A5*, *SLC16A14*, *SLC16A9*, *SLC17A5*, *SLC19A3*, *SLC1A2*, *SLC1A6*, *SLC1A7*, *SLC22A23*, *SLC22A9*, *SLC24A3*, *SLC24A4*, *SLC25A21*, *SLC25A25*, *SLC25A37*, *SLC26A7*, *SLC27A2*, *SLC27A6*, *SLC28A3*, *SLC2A9*, *SLC35B3*, *SLC35F1*, *SLC35F2*, *SLC35F3*, *SLC35F4*, *SLC36A2*, *SLC37A1*, *SLC38A9*, *SLC39A11*, *SLC39A12*, *SLC5A12*, *SLC6A15*, *SLC7A1*, *SLC9A4*, *SLC9A9*, *SLC9C1*, *SLCO1B3*, *SLCO5A1*, *SLCO6A1*, *SNTB1*, *SNTG1*, *SNTG2*, *SORCS1*, *SORCS2*, *SPATA13*, *SPINK5*, *SPRED1*, *SPTB*, *SPTLC3*, *SRD5A1*, *SSR1*, *ST3GAL6*, *ST6GAL1*, *ST6GALNAC3*, *ST8SIA1*, *ST8SIA6*, *STK39*, *STON1*, *STON2*, *STT3A*, *STXBP6*, *STYK1*, *SV2B*, *SV2C*, *SYK*, *SYNE1*, *SYNGR1*, *SYNJ2*, *SYNJ2BP*, *SYNPR*, *SYT1*, *SYT10*, *SYT5*, *SYT6*, *SYT9*, *TAP2*, *TECRL*, *TEK*, *TESC*, *TEX2*, *TGFA*, *THSD7A*, *THSD7B*, *TIMM21*, *TKT*, *TLR10*, *TMC1*, *TMC2*, *TMC5*, *TMCC3*, *TMCO4*, *TMCO5A*, *TMEM106B*, *TMEM108*, *TMEM110*, *TMEM117*, *TMEM128*, *TMEM132B*, *TMEM132C*, *TMEM132D*, *TMEM156*, *TMEM163*, *TMEM176B*, *TMEM196*, *TMEM220*, *TMEM229B*, *TMEM244*, *TMEM39A*, *TMEM63C*, *TMPRSS15*, *TMPRSS9*, *TMTC1*, *TMTC2*, *TNFRSF10D*, *TNR*, *TREM1*, *TRHDE*, *TRPA1*, *TRPC4*, *TRPC6*, *TRPM3*, *TSHR*, *TSPAN8*, *TSPAN9*, *UCP2*, *UGT1A10*, *UGT1A5*, *UGT1A6*, *UGT1A7*, *UGT1A8*, *UGT1A9*, *UNC5C*, *UNC5D*, *UNC79*, *UTRN*, *VNN1*, *VPS26A*, *VPS45*, *VWF*, *WBSCR17*, *WDFY4*, *WDR11*, *WNT3*, *WSCD2*, *XKR4*, *XKR6*, *XYLT1*, *YIPF1*, *ZDHHC14*, *ZDHHC7*
Integral to lumenal side of endoplasmic reticulum membrane	1.0×10^{-12}	34.9	*HLA-A*, *HLA-B*, *HLA-C*, *HLA-DPA1*, *HLA-DPB1*, *HLA-DQA1*, *HLA-DQA2*, *HLA-DQB1*, *HLA-DRB1*, *HLA-DRB5*

GO categories in which false discovery rate is less than 0.01.

Table S27 continued.

Description	p-value	Enrichment	Genes
Membrane	1.1×10^{-12}	1.3	*AADAC, ABCA1, ABCA12, ABCA4, ABCB11, ABCB5, ABCC4, ABCC6, ABCC8, ABCD4, ABO, ACBD5, ACPP, ACSBG2, ADAM12, ADAM28, ADCY5, ADCY8, ADD2, ADRA1A, AGMO, AGPAT5, AGPAT9, AKAP6, ALDH9A1, ALG8, ALPL, ALS2, AMPH, ANGPT1, ANGPT2, ANK2, ANK3, ANKH, ANKS1B, ANO2, ANO6, AP1S3, APBB1IP, APBB2, APPBP2, ARAP1, ARHGAP15, ARHGAP24, ARHGEF18, ASAP1, ATP10A, ATP10B, ATP10D, ATP12A, ATP1A2, ATP2C2, ATP6V0A4, ATP6V0E2, ATP8A1, AVEN, BAI3, BCR, BEST3, BFSP1, BICD1, BLK, BLNK, BMPR1B, BNC2, BRI3BP, BSPRY, BST1, BTN3A1, C1GALT1, C8A, CACNA1A, CACNA1C, CADM2, CADPS, CADPS2, CALCR, CALN1, CAMK2D, CAP1, CAP2, CAPN2, CAPN3, CASQ2, CBFA2T3, CCDC88A, CCDC91, CCZ1B, CD200, CD81, CD84, CDH10, CDH12, CDH13, CDH18, CDH23, CDH4, CDH9, CDKAL1, CDON, CELSR2, CERS6, CHCHD3, CHMP4C, CHN2, CHRM5, CHRNB3, CHST11, CHST8, CHST9, CLDN10, CLDN14, CLDN18, CLDN23, CLEC12A, CLECL1, CLSTN2, CNGA3, CNIH3, CNR2, CNTLN, CNTN1, CNTN2, CNTN4, CNTN5, CNTN6, CNTNAP2, COG6, COL23A1, COL25A1, COPG1, CORIN, CORO2B, CPE, CSF2RB, CSMD2, CSMD3, CTNS, CUBN, CUX1, CXADR, CYB5A, CYBRD1, CYP2E1, CYP2U1, CYP39A1, CYP3A43, CYP4F11, CYP4F12, CYP4F3, CYP4F8, CYP4Z1, DAD1, DCC, DCHS2, DGKB, DGKH, DGKI, DHRS4, DIO1, DISC1, DLG2, DLGAP1, DLGAP2, DMBT1, DMXL2, DNER, DOCK1, DOCK2, DOCK4, DRAM1, DSC1, DSC3, DSCAM, DSG1, DSG3, DTNA, DYNC2H1, DYSF, DYTN, EBNA1BP2, EGFR, ELMO1, ELOVL6, ELTD1, EMID2, EMR2, ENOX1, ENPP1, ERAP1, ERAP2, ERBB4, ERC2, ERO1LB, ESR1, ESRP1, ESYT2, EXOC2, EXT1, F5, FABP2, FAM188A, FAS, FAT2, FAT3, FCAR, FCRL2, FCRL5, FHIT, FLNB, FLOT1, FLVCR1, FMN1, FMO2, FNDC1, FRAS1, FRMPD1, FSHR, FUT10, FUT9, FXYD6, GAA, GABBR2, GABRB3, GABRG3, GABRR1, GALNT10, GALNT14, GALNT2, GALNTL4, GALNTL5, GALNTL6, GAS2, GGA1, GGT1, GLDN, GLRA3, GNG2, GNG4, GP5, GPC5, GPC6, GPM6A, GPR111, GPR115, GPR137B, GPR158, GPR39, GPR78, GRB10, GRIA1, GRID1, GRID2, GRIK1, GRIK2, GRIK4, GRIN2A, GRIP1, GRK5, GRM1, GRM5, GRM7, GRM8, GUCA1A, GUCA1B, HADHB, HHAT, HK1, HLA-A, HLA-B, HLA-C, HLA-DOB, HLA-DPA1, HLA-DPB1, HLA-DQA1, HLA-DQA2, HLA-DQB1, HLA-DQB2, HLA-DRA, HLA-DRB1, HLA-DRB5, HLA-F, HLA-G, HMGCLL1, HMMR, HOMER1, HPSE, HPSE2, HS3ST2, HS3ST4, IGSF5, IKZF3, IL1R2, IL1RAP, INADL, INPP5D, ITGA1, ITGA11, ITGA2, ITGA6, ITGA8, ITGA9, ITGAE, ITGB3, ITGB5, KCNA6, KCNAB1, KCNB2, KCNC2, KCND3, KCNE1, KCNH5, KCNH7, KCNH8, KCNIP1, KCNIP4, KCNJ12, KCNJ6, KCNK10, KCNK18, KCNK2, KCNMB2, KCNMB3, KCNQ5, KCNV2, KIAA0319, KIF13A, KIF16B, KIRREL, KIRREL3, KL, KLHL14, KLRB1, KNG1, KRT5, KSR2, LAMA2, LAMP3, LCT, LDLRAD3, LHCGR, LOC646627, LOXL2, LPHN3, LPP, LRP8, LRPPRC, LRRC4C, LRRC52, LRRTM4, LYPD6B, MAGI1, MAGI2, MAMDC2, MAPK10, MARC2, MARCH1, MARCH4, MBP, MCC, MDGA2, MGAM, MGAT1, MGAT5, MGAT5B, MGST3, MOGAT2, MPP6, MRAP2, MRGPRX4, MTCH1, MTUS1, MUC16, MX1, MYH9, MYO10, MYO16, MYO1B, MYO5B, MYOF, NAV3, NCAM2, NDST4, NDUFAF6, NDUFB1, NEDD4, NETO1, NFAM1, NFASC, NKAIN1, NKAIN3, NLGN1, NMNAT2, NMUR1, NOTCH2, NOTCH4, NPFFR2, NPSR1, NR3C1, NRCAM, NRG1, NRP1, NRXN1, NTM, NTN4, NTRK2, NTRK3, OCA2, ODZ3, OMA1, OPCML, OPRK1, OR10C1, OR12D2, OR13C5, OR14A16, OR1A2, OR1N2, OR2AK2, OR2B11, OR2L13, OR2L8, OR2W3, OR4C3, OR4C45, OR51B6, OR51E1, OR51F1, OR51I2, OR51L1, OR51M1, OR51Q1, OR52B6, OR52E2, OR52E4, OR52E6, OR52J3, OR52R1, OR5B3, OR5P2, OR6K6, OR9K2, OSBPL5, OSTM1, PALM2, PALMD, PARD3, PARD6G, PARVA, PCDH15, PCDHA12, PCDHA13, PCDHA9, PCLO, PCM1, PDE2A, PDE4D, PDE6C, PDGFD, PDIA6, PDLIM5, PEX5L, PGAP3, PGLYRP4, PGS1, PHLDB2, PIGG, PIGH, PIGL, PIK3C2G, PIK3R6, PIKFYVE, PITPNB, PKD1L1, PKDREJ, PLA2G4C, PLA2G4E, PLA2G6, PLA2R1, PLB1, PLCB1, PLCG2, PLCH1, PLD1, PLOD2, PLSCR1, PLXNA4, PNKD, PNPLA3, PNPLA6, POMP, PPFIBP1, PPP1R14C, PPP2CA, PPP3CA, PQLC2, PRICKLE1, PRKCA, PRKCB, PRKCE, PRKD1, PRKG1, PRLR, PSD3, PSTPIP2, PTCH1, PTGER3, PTGFRN, PTGS1, PTPRN2, PTPRO, PTPRT, PXK, RAB27A, RAB36, RAMP3, RAPGEF4, RECK, RGNEF, RGS1, RGS12, RGS13, RGS6, RGS7, RIMBP2, RIMS1, RIMS2, RNF144B, RNPEP, ROR1, RPSA, RRP12, RYR2, RYR3, SBF2, SCARB1, SCD5, SCG3, SCLT1, SCN3B, SCTR, SDF4, SDHA, SDHC, SEC22A, SEMA3A, SEMA3E, SEMA5A, SEMA6D, SERINC5, SERPINB2, SGCG, SGCZ, SGIP1, SH3GL2, SHISA2, SIRPA, SKAP2, SLC12A3, SLC12A6, SLC14A2, SLC15A2, SLC16A14, SLC16A9, SLC17A5, SLC19A3, SLC1A2, SLC1A6, SLC1A7, SLC22A16, SLC24A3, SLC24A4, SLC25A21, SLC25A25, SLC25A37, SLC26A7, SLC27A2, SLC27A6, SLC28A3, SLC2A9, SLC35B3, SLC36A2, SLC39A14, SLC44A3, SLC6A15, SLC7A1, SLC9A4, SLC9A9, SLC9C1, SLCO1B3, SLCO1C1, SLCO5A1, SLCO6A1, SNTB1, SNTG2, SNX19, SNX7, SORCS1, SORCS2, SORD, SPINK5, SPRED1, SPTLC3, SQRDL, SRD5A1, SSR1, ST3GAL6, ST6GAL1, ST8SIA6, STARD13, STT3A, STYK1, SV2B, SV2C, SVIL, SWAP70, SYK, SYN3, SYNE1, SYNGR1, SYNPR, SYT1, SYT10, SYT5, SYT6, SYT9, TANC1, TAP2, TAS1R2, TEK, TESC, TGFA, THSD7A, TIAM1, TIMM21, TJP2, TKT, TLR10, TMEM106B, TMEM163, TMEM176B, TMEM63A, TMEM70, TNKS, TOM1, TREM1, TRIP11, TRPC4, TRPC6, TSHR, TSPAN9, TST, TUBB, TULP3, TYMS, UBR2, UCP2, UGT1A10, UGT1A5, UGT1A6, UGT1A7, UGT1A8, UGT1A9, UNC13A, UNC13C, UNC5C, USP32, UTRN, VAV1, VAV2, VAV3, VCL, VEPH1, VNN1, VPS26A, VPS36, VPS45, WBSCR17, WDPCP, WDR93, WNT3, WWOX, XXYLT1, XYLT1, ZFYVE28*

GO categories in which false discovery rate is less than 0.01.

Table S27 continued.

Description	p-value	Enrichment	Genes
Intrinsic to membrane	2.8×10^{-12}	1.3	*AADAC, ABCA1, ABCA12, ABCA13, ABCA4, ABCB11, ABCB5, ABCC4, ABCD4, ABHD2, ABHD6, ABO, ACBD5, ACPP, ADAM12, ADAM28, ADAM29, ADAM32, ADCY5, ADCY8, ADRA1A, AGMO, AGPAT5, ALG8, ALK, ALPL, ANK2, ART3, ASGR2, ASIC2, ASTN2, ATP10A, ATP10B, ATP10D, ATP13A5, ATP2C2, ATP6V0A4, ATP6V0E2, ATP8A1, ATP8A2, ATRNL1, BAI3, BMPR1B, BST1, BTBD11, BTN3A1, BTNL2, BTNL3, C1GALT1, CACNA1A, CACNA1C, CACNA2D3, CADM2, CALCR, CALN1, CD200, CD81, CD84, CDH10, CDH12, CDH13, CDH23, CDH4, CDH9, CDKAL1, CDON, CELSR2, CHODL, CHRM5, CHRNB3, CHST11, CHST8, CHST9, CLCA2, CLCNKB, CLDN10, CLDN14, CLDN23, CLEC12A, CLEC1A, CLEC4D, CLEC6A, CLECL1, CLMN, CLSTN2, CMTM8, CNGA3, CNR2, CNTN1, CNTN2, CNTN4, CNTN5, CNTN6, CNTNAP2, CNTNAP4, CNTNAP5, COL23A1, COL25A1, COLEC12, CSMD1, CSMD2, CSMD3, CTNS, CUX1, CXADR, CYB5A, CYBRD1, CYP2E1, CYP2U1, CYP4F11, CYP4F12, CYP4F3, CYP4F8, CYP4Z1, DAD1, DCC, DCHS2, DDR2, DIO1, DNER, DPP6, DRAM1, DSC1, DSC3, DSCAM, DSG1, DSG3, DYSF, EGFR, ELOVL6, ELTD1, EMR1, EMR2, ENPP1, EPHA3, EPHA6, ERAP1, ERAP2, ERBB4, ESR1, ESYT2, EVC, EVC2, EXT1, FAM155A, FAM189A1, FAT2, FAT3, FCAR, FCER2, FER1L6, FLNB, FLOT1, FLT3, FLVCR1, FMO2, FMO4, FMO5, FNDC9, FRAS1, FSHR, FUT10, FUT9, GABBR2, GABRB3, GABRG3, GABRR1, GALNT10, GALNT2, GALNTL4, GALNTL5, GALNTL6, GGT1, GLDN, GLRA3, GP5, GPC5, GPC6, GPM6A, GPNMB, GPR111, GPR115, GPR137B, GPR158, GPR39, GRAMD1A, GRID1, GRID2, GRIK1, GRIK2, GRIK4, GRIN2A, GRM1, GRM5, GRM7, GRM8, HHAT, HHLA2, HLA-A, HLA-B, HLA-C, HLA-DOB, HLA-DPA1, HLA-DPB1, HLA-DQA1, HLA-DQA2, HLA-DQB1, HLA-DQB2, HLA-DRA, HLA-DRB1, HLA-DRB5, HLA-F, HLA-G, HS3ST2, HS3ST4, IER3, IFI27L1, IGSF5, IL12RB2, IL1R2, IL1RAP, IL1RL1, IL1RL2, IL28RA, IMMP2L, ITGB3, KCNAB1, KCNH7, KCNH8, KCNJ12, KCNK10, KCNK18, KCNMB3, KIAA0319, KIAA1324L, KIRREL, KIRREL3, KL, KLRB1, LAMP3, LAPTM4B, LARGE, LCT, LDLRAD3, LGR5, LHFP, LHFPL2, LHFPL3, LPHN3, LPPR1, LPPR5, LRFN5, LRP1B, LRP8, LRRC4C, LRRC52, LRRTM4, LYPD6B, MAEA, MARCH1, MARCH4, MCTP2, MDGA2, MEGF11, MGAM, MGAT1, MGAT5, MGST3, MS4A12, MSR1, MTCH1, MUC12, MUC16, MUC22, MYOF, NAALADL2, NALCN, NCAM2, NDST4, NDUFB1, NETO1, NFAM1, NFASC, NKAIN1, NKAIN3, NLGN1, NOTCH2, NOTCH4, NPFFR2, NPR3, NPSR1, NRCAM, NRG1, NRG3, NRXN1, NRXN3, NTM, NTRK2, NTRK3, OCA2, ODZ3, ODZ4, OMA1, OPCML, OPN3, OPRK1, OR10C1, OR12D2, OR13C5, OR14A16, OR1A2, OR1N2, OR2AK2, OR2B11, OR2L13, OR2L8, OR2W3, OR4C3, OR4C45, OR51B6, OR51E1, OR51F1, OR51M1, OR52B6, OR52E2, OR52E4, OR52E6, OR52J3, OR5B3, OR5P2, OR6K6, OR9K2, OSTM1, OTOP1, PCDH15, PCDHA1, PCDHA10, PCDHA11, PCDHA12, PCDHA13, PCDHA2, PCDHA3, PCDHA4, PCDHA5, PCDHA6, PCDHA7, PCDHA8, PCDHA9, PCDHAC1, PCNXL2, PCSK5, PDE3A, PGAP3, PGBD5, PIEZO2, PIGG, PIGL, PKD1L1, PKD1L2, PKDREJ, PKHD1, PKHD1L1, PLA2R1, PLB1, PLD5, PLEKHH2, PLSCR1, PLXDC2, PLXNA4, PNPLA3, PNPLA6, PQLC2, PRKD1, PRLR, PTCH1, PTCHD3, PTGER3, PTGFRN, PTPRB, PTPRD, PTPRH, PTPRM, PTPRN2, PTPRQ, PTPRT, RAMP3, RECK, RGSL1, RNF144B, RNF150, RNFT2, ROBO2, ROR1, RRP12, RYR3, SCARB1, SCD5, SCN1A, SCN3B, SCNN1G, SCTR, SDHC, SDK1, SEMA5A, SEMA6D, SERINC5, SGCG, SGCZ, SHISA2, SHISA6, SIRPA, SLC12A3, SLC12A6, SLC12A8, SLC14A2, SLC15A2, SLC15A5, SLC16A14, SLC16A9, SLC17A5, SLC19A3, SLC1A2, SLC1A6, SLC1A7, SLC22A23, SLC22A9, SLC24A3, SLC24A4, SLC25A21, SLC25A25, SLC25A37, SLC26A7, SLC27A2, SLC27A6, SLC28A3, SLC2A9, SLC35B3, SLC35F1, SLC35F2, SLC35F3, SLC35F4, SLC36A2, SLC37A1, SLC38A9, SLC39A11, SLC39A12, SLC5A12, SLC6A15, SLC7A1, SLC9A4, SLC9A9, SLC9C1, SLCO1B3, SLCO5A1, SORCS1, SORCS2, SPTB, SPTLC3, SRD5A1, SSR1, ST3GAL6, ST6GAL1, ST6GALNAC3, ST8SIA1, ST8SIA6, STT3A, STXBP6, STYK1, SV2B, SV2C, SYNE1, SYNGR1, SYNJ2BP, SYNPR, SYT1, SYT10, SYT5, SYT6, SYT9, TAP2, TECRL, TEK, TEX2, TGFA, THSD7A, THSD7B, TIMM21, TLR10, TMC1, TMC2, TMC5, TMCC3, TMCO4, TMCO5A, TMEM106B, TMEM110, TMEM117, TMEM128, TMEM132B, TMEM132C, TMEM132D, TMEM163, TMEM176B, TMEM196, TMEM220, TMEM229B, TMEM244, TMEM39A, TMEM63C, TMPRSS15, TMPRSS9, TMTC1, TMTC2, TNFRSF10D, TREM1, TRHDE, TRPA1, TRPC4, TRPC6, TRPM3, TSHR, TSPAN8, TSPAN9, UCP2, UGT1A10, UGT1A5, UGT1A6, UGT1A7, UGT1A8, UGT1A9, UNC5C, UNC5D, UNC79, VNN1, VPS45, WBSCR17, WDFY4, WDR11, XKR4, XKR6, XYLT1, YIPF1, ZDHHC14, ZDHHC7*

GO categories in which false discovery rate is less than 0.01.

Table S27 continued.

Description	p-value	Enrichment	Genes
Integral to membrane	4.4×10^{-12}	1.3	*AADAC, ABCA1, ABCA12, ABCA13, ABCA4, ABCB11, ABCB5, ABCC4, ABCD4, ABHD2, ABHD6, ABO, ACBD5, ACPP, ADAM12, ADAM28, ADAM29, ADAM32, ADCY5, ADCY8, ADRA1A, AGMO, AGPAT5, ALG8, ALK, ALPL, ANK2, ART3, ASGR2, ASIC2, ASTN2, ATP10A, ATP10B, ATP10D, ATP13A5, ATP2C2, ATP6V0A4, ATP6V0E2, ATP8A1, ATP8A2, ATRNL1, BAI3, BMPR1B, BTBD11, BTN3A1, BTNL2, BTNL3, C1GALT1, CACNA1A, CACNA1C, CACNA2D3, CADM2, CALCR, CALN1, CD200, CD81, CD84, CDH10, CDH12, CDH23, CDH4, CDH9, CDKAL1, CDON, CELSR2, CHODL, CHRM5, CHRNB3, CHST11, CHST8, CHST9, CLCA2, CLCNKB, CLDN10, CLDN14, CLDN23, CLEC12A, CLEC1A, CLEC4D, CLEC6A, CLECL1, CLMN, CLSTN2, CMTM8, CNGA3, CNR2, CNTN2, CNTNAP2, CNTNAP4, CNTNAP5, COL23A1, COL25A1, COLEC12, CSMD1, CSMD2, CSMD3, CTNS, CUX1, CXADR, CYB5A, CYBRD1, CYP2U1, CYP4F11, CYP4F12, CYP4F3, CYP4F8, CYP4Z1, DAD1, DCC, DCHS2, DDR2, DIO1, DNER, DPP6, DRAM1, DSC1, DSC3, DSCAM, DSG1, DSG3, DYSF, EGFR, ELOVL6, ELTD1, EMR1, EMR2, ENPP1, EPHA3, EPHA6, ERAP1, ERAP2, ERBB4, ESR1, ESYT2, EVC, EVC2, EXT1, FAM155A, FAM189A1, FAT2, FAT3, FCAR, FCER2, FER1L6, FLNB, FLOT1, FLT3, FLVCR1, FMO2, FMO4, FMO5, FNDC9, FRAS1, FSHR, FUT10, FUT9, GABBR2, GABRB3, GABRG3, GABRR1, GALNT10, GALNT2, GALNTL4, GALNTL5, GALNTL6, GGT1, GLDN, GLRA3, GP5, GPC5, GPC6, GPM6A, GPNMB, GPR111, GPR115, GPR137B, GPR158, GPR39, GRAMD1A, GRID1, GRID2, GRIK1, GRIK2, GRIK4, GRIN2A, GRM1, GRM5, GRM7, GRM8, HHAT, HHLA2, HLA-A, HLA-B, HLA-C, HLA-DOB, HLA-DPA1, HLA-DPB1, HLA-DQA1, HLA-DQA2, HLA-DQB1, HLA-DQB2, HLA-DRA, HLA-DRB1, HLA-DRB5, HLA-F, HLA-G, HS3ST2, HS3ST4, IER3, IFI27L1, IGSF5, IL12RB2, IL1R2, IL1RAP, IL1RL1, IL1RL2, IL28RA, IMMP2L, ITGB3, KCNAB1, KCNH7, KCNH8, KCNJ12, KCNK10, KCNK18, KCNMB3, KIAA0319, KIAA1324L, KIRREL, KIRREL3, KL, KLRB1, LAMP3, LAPTM4B, LARGE, LCT, LDLRAD3, LGR5, LHFP, LHFPL2, LHFPL3, LPHN3, LPPR1, LPPR5, LRFN5, LRP1B, LRP8, LRRC4C, LRRC52, LRRTM4, MAEA, MARCH1, MARCH4, MCTP2, MEGF11, MGAM, MGAT1, MGAT5, MGST3, MS4A12, MSR1, MTCH1, MUC12, MUC16, MUC22, MYOF, NAALADL2, NALCN, NCAM2, NDST4, NDUFB1, NETO1, NFAM1, NFASC, NKAIN1, NKAIN3, NLGN1, NOTCH2, NOTCH4, NPFFR2, NPR3, NPSR1, NRCAM, NRG1, NRG3, NRXN1, NRXN3, NTRK2, NTRK3, OCA2, ODZ3, ODZ4, OMA1, OPCML, OPN3, OPRK1, OR10C1, OR12D2, OR13C5, OR14A16, OR1A2, OR1N2, OR2AK2, OR2B11, OR2L13, OR2L8, OR2W3, OR4C3, OR4C45, OR51B6, OR51E1, OR51F1, OR51M1, OR52B6, OR52E2, OR52E4, OR52E6, OR52J3, OR5B3, OR5P2, OR6K6, OR9K2, OSTM1, OTOP1, PCDH15, PCDHA1, PCDHA10, PCDHA11, PCDHA12, PCDHA13, PCDHA2, PCDHA3, PCDHA4, PCDHA5, PCDHA6, PCDHA7, PCDHA8, PCDHA9, PCDHAC1, PCNXL2, PCSK5, PDE3A, PGAP3, PGBD5, PIEZO2, PIGG, PIGL, PKD1L1, PKD1L2, PKDREJ, PKHD1, PKHD1L1, PLA2R1, PLB1, PLD5, PLEKHH2, PLSCR1, PLXDC2, PLXNA4, PNPLA3, PNPLA6, PQLC2, PRKD1, PRLR, PTCH1, PTCHD3, PTGER3, PTGFRN, PTPRB, PTPRD, PTPRH, PTPRM, PTPRN2, PTPRQ, PTPRT, RAMP3, RGSL1, RNF144B, RNF150, RNFT2, ROBO2, ROR1, RRP12, RYR3, SCARB1, SCD5, SCN1A, SCN3B, SCNN1G, SCTR, SDHC, SDK1, SEMA5A, SEMA6D, SERINC5, SGCG, SGCZ, SHISA2, SHISA6, SIRPA, SLC12A3, SLC12A6, SLC12A8, SLC14A2, SLC15A2, SLC15A5, SLC16A14, SLC16A9, SLC17A5, SLC19A3, SLC1A2, SLC1A6, SLC1A7, SLC22A23, SLC22A9, SLC24A3, SLC24A4, SLC25A21, SLC25A25, SLC25A37, SLC26A7, SLC27A2, SLC27A6, SLC28A3, SLC2A9, SLC35B3, SLC35F1, SLC35F2, SLC35F3, SLC35F4, SLC36A2, SLC37A1, SLC38A9, SLC39A11, SLC39A12, SLC5A12, SLC6A15, SLC7A1, SLC9A4, SLC9A9, SLC9C1, SLCO1B3, SLCO5A1, SORCS1, SORCS2, SPTLC3, SRD5A1, SSR1, ST3GAL6, ST6GAL1, ST6GALNAC3, ST8SIA1, ST8SIA6, STT3A, STXBP6, STYK1, SV2B, SV2C, SYNE1, SYNGR1, SYNJ2BP, SYNPR, SYT1, SYT10, SYT5, SYT6, SYT9, TAP2, TECRL, TEK, TEX2, TGFA, THSD7A, THSD7B, TIMM21, TLR10, TMC1, TMC2, TMC5, TMCC3, TMCO4, TMCO5A, TMEM106B, TMEM110, TMEM117, TMEM128, TMEM132B, TMEM132C, TMEM132D, TMEM163, TMEM176B, TMEM196, TMEM220, TMEM229B, TMEM244, TMEM39A, TMEM63C, TMPRSS15, TMPRSS9, TMTC1, TMTC2, TNFRSF10D, TREM1, TRHDE, TRPA1, TRPC4, TRPC6, TRPM3, TSHR, TSPAN8, TSPAN9, UCP2, UGT1A10, UGT1A5, UGT1A6, UGT1A7, UGT1A8, UGT1A9, UNC5C, UNC5D, UNC79, VNN1, VPS45, WBSCR17, WDFY4, WDR11, XKR4, XKR6, XYLT1, YIPF1, ZDHHC14, ZDHHC7*
ER to Golgi transport vesicle membrane	9.4×10^{-12}	29.1	*HLA-A, HLA-B, HLA-C, HLA-DPA1, HLA-DPB1, HLA-DQA1, HLA-DQA2, HLA-DQB1, HLA-DRB1, HLA-DRB5*
MHC protein complex	4.0×10^{-11}	25.7	*HLA-A, HLA-B, HLA-C, HLA-DPA1, HLA-DPB1, HLA-DQA1, HLA-DQA2, HLA-DQB1, HLA-DRB1, HLA-DRB5*

GO categories in which false discovery rate is less than 0.01.

Table S27 continued.

Description	p-value	Enrichment	Genes
Plasma membrane	9.8×10^{-11}	1.3	*ABCA1, ABCA4, ABCB11, ABCB5, ABCC4, ABCC6, ABCC8, ACPP, ADAM12, ADAM28, ADCY5, ADCY8, ADD2, ADRA1A, ALDH9A1, ALPL, ANGPT1, ANGPT2, ANK2, ANK3, ANKH, ANKS1B, ANO2, ANO6, APBB1IP, ARAP1, ARHGAP24, ARHGEF18, ATP10A, ATP10D, ATP12A, ATP1A2, ATP6V0A4, ATP8A1, BAI3, BCR, BEST3, BFSP1, BLK, BLNK, BMPR1B, BNC2, BST1, BTN3A1, CACNA1A, CACNA1C, CADM2, CALCR, CALN1, CAMK2D, CAP1, CAP2, CAPN2, CAPN3, CCDC88A, CD200, CD81, CD84, CDH10, CDH12, CDH13, CDH18, CDH23, CDH4, CDH9, CDON, CELSR2, CHRM5, CHRNB3, CLDN10, CLDN14, CLDN18, CLDN23, CLEC12A, CLECL1, CLSTN2, CNGA3, CNR2, CNTN1, CNTN2, CNTN4, CNTN5, CNTN6, COL23A1, COL25A1, CORIN, CPE, CSF2RB, CSMD2, CSMD3, CTNS, CUBN, CXADR, CYBRD1, DCC, DCHS2, DGKB, DGKH, DGKI, DIO1, DISC1, DLG2, DLGAP1, DLGAP2, DNER, DSC1, DSC3, DSCAM, DSG1, DSG3, DTNA, DYNC2H1, DYSF, DYTN, EGFR, ELMO1, ELTD1, EMID2, EMR2, ENOX1, ENPP1, ERBB4, ESR1, ESRP1, ESYT2, EXOC2, F5, FABP2, FAS, FAT2, FAT3, FCAR, FCRL2, FCRL5, FHIT, FLNB, FLOT1, FLVCR1, FMN1, FNDC1, FRAS1, FRMPD1, FSHR, FXYD6, GABBR2, GABRB3, GABRG3, GABRR1, GGT1, GLDN, GLRA3, GNG2, GNG4, GP5, GPC5, GPC6, GPM6A, GPR111, GPR115, GPR158, GPR39, GRB10, GRIA1, GRID1, GRID2, GRIK1, GRIK2, GRIK4, GRIN2A, GRIP1, GRK5, GRM1, GRM5, GRM7, GRM8, GUCA1B, HLA-A, HLA-B, HLA-C, HLA-DPA1, HLA-DPB1, HLA-DQA1, HLA-DQA2, HLA-DQB1, HLA-DQB2, HLA-DRA, HLA-DRB1, HLA-DRB5, HLA-F, HLA-G, HMMR, HPSE2, IGSF5, IKZF3, IL1R2, IL1RAP, INADL, INPP5D, ITGA1, ITGA11, ITGA2, ITGA6, ITGA8, ITGA9, ITGAE, ITGB3, ITGB5, KCNA6, KCNAB1, KCNB2, KCNC2, KCND3, KCNE1, KCNH5, KCNH7, KCNH8, KCNIP1, KCNIP4, KCNJ12, KCNJ6, KCNK10, KCNK18, KCNK2, KCNMB2, KCNMB3, KCNQ5, KCNV2, KIAA0319, KIRREL, KIRREL3, KL, KLRB1, KNG1, KRT5, LAMA2, LCT, LDLRAD3, LHCGR, LOC646627, LPHN3, LPP, LRP8, LRRC52, LRRTM4, LYPD6B, MAGI1, MAGI2, MAPK10, MARCH1, MBP, MCC, MDGA2, MGAM, MPP6, MRAP2, MRGPRX4, MTUS1, MUC16, MYH9, MYO10, MYO16, MYO1B, MYOF, NCAM2, NEDD4, NETO1, NFAM1, NFASC, NKAIN1, NKAIN3, NMUR1, NOTCH2, NOTCH4, NPFFR2, NPSR1, NRCAM, NRP1, NRXN1, NTM, NTN4, OPCML, OPRK1, OR10C1, OR12D2, OR13C5, OR14A16, OR1A2, OR1N2, OR2AK2, OR2B11, OR2L13, OR2L8, OR2W3, OR4C3, OR4C45, OR51B6, OR51E1, OR51F1, OR51I2, OR51L1, OR51M1, OR51Q1, OR52B6, OR52E2, OR52E4, OR52E6, OR52J3, OR52R1, OR5B3, OR5P2, OR6K6, OR9K2, PALM2, PARD3, PARD6G, PARVA, PCDH15, PCDHA12, PCDHA13, PCDHA9, PDE2A, PDE6C, PDIA6, PDLIM5, PHLDB2, PIK3C2G, PKDREJ, PLA2R1, PLB1, PLCG2, PLSCR1, PLXNA4, PPFIBP1, PPP2CA, PRKCA, PRKCB, PRKCE, PRKD1, PRKG1, PRLR, PSD3, PTCH1, PTGER3, PTPRO, PTPRT, PXK, RAMP3, RAPGEF4, RECK, RGNEF, RGS1, RGS12, RGS13, RGS6, RGS7, RIMBP2, RIMS1, RIMS2, RNPEP, ROR1, RPSA, RYR2, SCARB1, SCLT1, SCN3B, SCTR, SDF4, SEMA5A, SEMA6D, SGCG, SGCZ, SGIP1, SH3GL2, SIRPA, SKAP2, SLC12A3, SLC12A6, SLC14A2, SLC15A2, SLC16A14, SLC16A9, SLC19A3, SLC1A2, SLC1A6, SLC1A7, SLC22A16, SLC24A3, SLC24A4, SLC26A7, SLC27A6, SLC28A3, SLC2A9, SLC36A2, SLC39A14, SLC44A3, SLC6A15, SLC7A1, SLC9A4, SLCO1B3, SLCO1C1, SLCO5A1, SLCO6A1, SNTB1, SNTG2, SPRED1, STYK1, SVIL, SWAP70, SYK, SYT1, SYT6, TANC1, TAP2, TAS1R2, TEK, TESC, TGFA, THSD7A, TIAM1, TJP2, TLR10, TREM1, TRPC4, TRPC6, TSHR, TST, TUBB, TULP3, UBR2, UNC13A, UNC13C, UNC5C, UTRN, VAV1, VAV2, VAV3, VCL, VEPH1, VNN1, WDPCP, WNT3, WWOX*
Cell junction	1.1×10^{-10}	1.9	*ABCB11, ABCB5, AFAP1, AMPH, AMTN, ANK2, ANK3, ANKS1B, APBB1IP, ARHGAP24, ARHGAP26, ARHGEF18, BSPRY, CADM2, CADPS, CCDC85C, CDC42BPA, CDSN, CHRM5, CHRNB3, CLCA2, CLDN10, CLDN14, CLDN23, CNIH3, CTNNA2, CTNNA3, CTNND2, CXADR, CYFIP2, DISC1, DLC1, DLG2, DLGAP1, DMXL2, DSC1, DSC3, DSG1, DSG3, DTNA, ERC2, FAT2, FER, FLNB, FNDC1, FRMD4A, GABBR2, GABRB3, GABRG3, GABRR1, GLRA3, GRIA1, GRID1, GRID2, GRIK1, GRIK2, GRIK4, GRIN2A, GRIP1, IGSF5, INADL, IQGAP3, IQSEC1, ITGB5, KIRREL, LIMD1, LPP, LRRTM4, MAGI1, MAGI2, MYH9, NETO1, NFASC, NFIA, NLGN1, NRAP, NRXN1, PALLD, PARD3, PARVA, PCLO, PHACTR1, PLEKHA7, POLR1E, PRKD1, PSD3, PTPRM, RIMBP2, RIMS1, RIMS2, SCN1A, SEPT11, SHISA9, SHROOM3, SNTB1, SV2B, SV2C, SVIL, SYN3, SYNGR1, SYNPR, SYT1, SYT10, SYT5, SYT6, SYT9, TANC1, TEK, TIAM1, TJP2, TMEM163, TNS1, TRIM9, TRPC4, TRPC6, UNC13C, UTRN, VCL, XIRP2*
Transport vesicle membrane	3.9×10^{-10}	17.5	*CPE, HLA-B, HLA-C, HLA-DPA1, HLA-DPB1, HLA-DQA1, HLA-DQA2, HLA-DQB1, HLA-DRB1, HLA-DRB5*
MHC class II protein complex	5.3×10^{-10}	14.5	*HLA-DOB, HLA-DPA1, HLA-DPB1, HLA-DQA1, HLA-DQA2, HLA-DQB1, HLA-DQB2, HLA-DRA, HLA-DRB1, HLA-DRB5*
Synapse part	8.4×10^{-10}	2.3	*ABCC8, ALS2, AMPH, ANK2, ANK3, ANKS1B, CACNA1C, CHRM5, CHRNB3, CLSTN2, CNIH3, CTNND2, DDC, DISC1, DLG2, DLGAP1, DMXL2, DNM3, ERBB4, ERC2, ESR1, GABBR2, GABRB3, GABRG3, GABRR1, GLRA3, GRIA1, GRID1, GRID2, GRIK1, GRIK2, GRIK4, GRIN2A, GRIP1, GRM1, GRM5, GRM7, GRM8, LRRTM4, MAGI2, NLGN1, NR3C1, NRXN1, NTRK2, PCLO, PLCB4, PSD3, RIMS1, SLC1A2, STON2, SV2B, SV2C, SYN3, SYNE1, SYNGR1, SYNPR, SYT10, SYT5, SYT6, SYT9, TANC1, TMEM163, TRIM9, UNC13C, UTRN*
Cell projection	1.2×10^{-9}	1.9	*ACPP, ADCY5, ANGPT2, APBB1IP, APBB2, APOA1BP, ARHGAP24, ARPC5, BMPR1B, CACNA1A, CADM2, CATSPERB, CDH13, CDH23, CNGA3, CNR2, CNTN4, CNTNAP2, CPLX1, CTNNA3, CYFIP2, DCC, DDC, DNAH8, DNER, DOCK4, DPYSL3, DSCAM, DYNC2H1, EVC, FAM65B, FGD5, FOPNL, FRMD4B, GAS7, GRIA1, GRID2, GRIP1, GRM1, GRM7, GRM8, IFT57, IQGAP2, ITGA1, KALRN, KCND3, KCNIP1, KCNIP4, KCNJ6, KIRREL3, KLHL1, KLHL14, KLHL24, LDB3, LDHC, LRRC16A, MAGI1, MTSS1, MYH9, MYO10, MYO1B, MYO3A, MYO5B, MYRIP, NCAM2, NLGN1, NOV, NRCAM, NTM, PACRG, PALLD, PARVA, PCDH15, PCSK1, PKHD1, PLD1, PTGS1, PTPRM, RAB27A, RAPGEF4, RELN, RPTOR, SCN11A, SDF4, SEMA3A, SEPT11, SLC1A2, SLC9C1, SORD, SPATA13, SVIL, SWAP70, SYNPR, SYT5, TEK, TIAM2, TRIM9, TTLL11, TULP3, WWOX*

GO categories in which false discovery rate is less than 0.01.

Table S27 continued.

Description	p-value	Enrichment	Genes
Plasma membrane part	1.4×10^{-9}	1.5	*ABCA1, ABCA4, ABCB11, ABCB5, ABCC8, ADAM29, ADRA1A, ALK, ANK2, ANK3, ART3, ASIC2, ATP1A2, ATP6V0A4, BMPR1B, CACNA1A, CACNA1C, CACNA2D1, CALCR, CAMK2D, CAPN3, CASQ2, CATSPERB, CD200, CD81, CD84, CDH13, CDH4, CHRM5, CHRNB3, CLCA2, CLCNKB, CLEC1A, CNIH3, CNR2, CNTN2, CNTNAP2, COL25A1, CTNNA2, CUBN, CXADR, CYBRD1, CYP4F12, DCC, DDR2, DLC1, DSCAM, DSG1, DYSF, EGFR, EMR1, EMR2, ENPP1, EPHA3, EPHA6, ERBB4, ESR1, EVC, EVC2, FCAR, FCER2, FER, FGD5, FLOT1, FLT3, FLVCR1, GABBR2, GABRB3, GABRR1, GGT1, GLRA3, GNG2, GNG4, GP5, GPC5, GPC6, GPNMB, GPR137B, GPR39, GRIA1, GRID2, GRIK1, GRIK2, GRIK4, GRIN2A, GRM1, GRM5, GRM7, GRM8, GZMB, HLA-A, HLA-B, HLA-C, HLA-DOB, HLA-DPA1, HLA-DPB1, HLA-DQA1, HLA-DQA2, HLA-DQB1, HLA-DQB2, HLA-DRA, HLA-DRB1, HLA-DRB5, HLA-F, HLA-G, IL12RB2, IL1RAP, IL1RL1, IL1RL2, IL28RA, INADL, IQGAP3, ITGA1, ITGA11, ITGA2, ITGA6, ITGA8, ITGA9, ITGAE, ITGB3, ITGB5, ITGBL1, KANK1, KCNA6, KCNC2, KCND3, KCNJ6, KCNK18, KCNK2, KCNMB2, KCNMB3, KCNQ5, KCNV2, KIRREL, KL, LCT, LGR5, LRP8, MAEA, MEGF11, MGAM, MSR1, MUC12, MYH9, MYO10, MYOF, NEDD4, NLGN1, NOTCH2, NOTCH4, NPFFR2, NPR3, NRCAM, NRG1, NRG3, NRXN1, NRXN3, NTRK2, NTRK3, OPCML, OPN3, OPRK1, PCDHA1, PCDHA10, PCDHA11, PCDHA2, PCDHA3, PCDHA4, PCDHA5, PCDHA6, PCDHA7, PCDHA8, PCDHAC1, PCSK9, PKHD1, PLA2R1, PLB1, PLSCR1, PRKAB2, PRKD1, PRKG2, PTCH1, PTGER3, PTPRB, PTPRD, PTPRH, PTPRM, PTPRN2, RAB27A, RAMP3, RAPGEF4, RGS6, RGS7, RNPEP, ROBO2, ROR1, SCARA5, SCARB1, SCN11A, SCN1A, SCN3A, SCN3B, SCN9A, SCNN1G, SCTR, SCUBE1, SERPINE2, SGCG, SGCZ, SGIP1, SHISA9, SHROOM3, SLC12A3, SLC12A6, SLC14A2, SLC15A2, SLC17A5, SLC1A2, SLC1A6, SLC26A7, SLC2A9, SLC5A12, SLC6A15, SLC7A1, SLC9A4, SLC9C1, SLCO1B3, SNTB1, SNTG1, SNTG2, SPATA13, SPRED1, SPTB, STK39, SYK, SYNJ2, TEK, TESC, TGFA, TMPRSS9, TRHDE, TRPA1, TRPC4, TSHR, TSPAN9, UTRN, VWF*
Trans-Golgi network membrane	5.1×10^{-9}	25.9	*HLA-DPA1, HLA-DPB1, HLA-DQA1, HLA-DQA2, HLA-DQB1, HLA-DRB1, HLA-DRB5, MARCH1*
Endocytic vesicle membrane	2.5×10^{-8}	10.4	*CUBN, DMBT1, HLA-A, HLA-B, HLA-C, HLA-DPA1, HLA-DPB1, HLA-DQA1, HLA-DQA2, HLA-DQB1, HLA-DRB1, HLA-DRB5*
Clathrin-coated endocytic vesicle membrane	3.4×10^{-8}	27.8	*HLA-DPA1, HLA-DPB1, HLA-DQA1, HLA-DQA2, HLA-DQB1, HLA-DRB1, HLA-DRB5*
Integral to endoplasmic reticulum membrane	5.4×10^{-8}	11.3	*HLA-A, HLA-B, HLA-C, HLA-DPA1, HLA-DPB1, HLA-DQA1, HLA-DQA2, HLA-DQB1, HLA-DRB1, HLA-DRB5, TAP2*
Cytoplasmic vesicle membrane	2.4×10^{-7}	8.0	*CPE, DMBT1, HLA-B, HLA-C, HLA-DPA1, HLA-DPB1, HLA-DQB1, MARCH1, MYO5B, SNX19*
Intrinsic to endoplasmic reticulum membrane	2.4×10^{-7}	8.6	*FMO5, HLA-A, HLA-B, HLA-C, HLA-DPA1, HLA-DPB1, HLA-DQA1, HLA-DQA2, HLA-DQB1, HLA-DRB1, HLA-DRB5, TAP2*
Vesicle membrane	3.9×10^{-7}	7.6	*CPE, DMBT1, HLA-A, HLA-C, HLA-DPA1, HLA-DPB1, HLA-DQB1, HLA-DRB5, MARCH1, MYO5B, SNX19*
Coated vesicle membrane	6.4×10^{-7}	9.0	*HLA-A, HLA-B, HLA-C, HLA-DPA1, HLA-DPB1, HLA-DQA1, HLA-DQA2, HLA-DQB1, HLA-DRB1, HLA-DRB5, SYT9*
Cell projection part	9.1×10^{-7}	1.7	*ALS2, AMPH, ANK3, ANKS1B, APBB2, ATP1A2, ATP6V0A4, C2CD3, CACNA1C, CDH23, CNIH3, CNTN2, CNTNAP2, CTNND2, CUBN, CXADR, CYBRD1, DCC, DFNB31, DISC1, DLG2, DLGAP1, DLGAP2, DNAH11, DNAH12, DNAH17, DNAH5, DNAH8, DNAH9, DNM3, DPYSL2, DPYSL3, DSCAM, DYNC2H1, EMR2, ERBB4, ERC2, ESR1, EVC, EVC2, FGD5, FOPNL, GPM6A, GRIA1, GRID2, GRIK1, GRIK2, GRIK4, GRIN2A, GRM1, GRM5, GRM7, HAP1, HOMER1, IFT57, KANK1, KIRREL, KIRREL3, MAGI2, MAP2, MBP, MYH14, MYO10, MYO5A, MYO5B, NETO1, NFASC, NLGN1, NR3C1, NRCAM, NRP1, NRXN1, NTRK2, PACRG, PALMD, PCSK1, PDLIM5, PKD1L1, PKHD1, PLCB4, PSD3, PTPRN2, PTPRO, RAPGEF4, ROBO2, RUFY3, SCARB1, SCN1A, SEPT11, SHISA9, SLC1A2, SLC9C1, SNTG1, SPAG16, SPATA13, SYNJ2, TANC1, TEKT1, TEKT4, TEKT5, TESC, TIAM2, TTLL11, WDPCP*
Synaptic membrane	3.1×10^{-6}	2.1	*ANK2, ANK3, ANKS1B, CADPS2, CHRM5, CHRNB3, CLSTN2, CNIH3, DISC1, DLG2, DLGAP1, DLGAP2, ERC2, GABBR2, GABRB3, GABRG3, GABRR1, GLRA3, GRIA1, GRID1, GRID2, GRIK1, GRIK2, GRIK4, GRIN2A, GRIP1, GRM1, GRM7, GRM8, HOMER1, LRRTM4, NETO1, NLGN1, NRXN1, PDE2A, PDLIM5, PSD3, RIMS1, RIMS2, SLC1A2, SYNE1, SYT1, TANC1, UNC13A, UNC13C, UTRN*
Cytoplasmic vesicle part	3.6×10^{-6}	2.1	*ABCC4, AMPH, AP1S3, APPBP2, ATP10B, ATP6V0A4, ATP6V0E2, ATP8A1, CADPS, CAMK2D, CPE, CUBN, DMBT1, DMXL2, DYSF, F13A1, F5, GRIA1, HLA-A, HLA-B, HLA-C, HLA-DPA1, HLA-DPB1, HLA-DQA1, HLA-DQA2, HLA-DQB1, HLA-DQB2, HLA-DRA, HLA-DRB1, HLA-DRB5, HLA-F, HLA-G, KNG1, LAMP3, MARCH1, MYO5B, MYOF, NCALD, OCA2, RAB27A, SNX19, SNX7, SV2C, SYN3, SYNGR1, SYNPR, SYT5, SYT6, SYT9, TMEM163, VWF*
Clathrin-coated vesicle membrane	9.6×10^{-6}	3.1	*ABCC8, AMPH, DMXL2, HLA-DPA1, HLA-DPB1, HLA-DQA1, HLA-DQA2, HLA-DQB1, HLA-DQB2, HLA-DRA, HLA-DRB1, HLA-DRB5, SH3GL2, SV2B, SV2C, SYN3, SYNGR1, SYNPR, SYT1, SYT10, SYT5, SYT6, SYT9*
Collagen type IV	9.8×10^{-6}	23.1	*COL4A1, COL4A2, COL4A3, COL4A4*
Intrinsic to organelle membrane	2.0×10^{-5}	4.8	*FMO5, HLA-A, HLA-B, HLA-C, HLA-DPA1, HLA-DPB1, HLA-DQA1, HLA-DQA2, HLA-DQB1, HLA-DRB1, HLA-DRB5, ST8SIA1, TAP2*
Integral to organelle membrane	2.4×10^{-5}	5.1	*HLA-A, HLA-B, HLA-C, HLA-DPA1, HLA-DPB1, HLA-DQA1, HLA-DQA2, HLA-DQB1, HLA-DRB1, HLA-DRB5, ST8SIA1, TAP2*
Neuron projection	2.4×10^{-5}	1.9	*ALS2, BMPR1B, CACNA1A, CADM2, CDH13, CNR2, CNTN4, CNTNAP2, CPLX1, CTNNA2, CYFIP2, DCC, DDC, DNER, DNM3, DSCAM, GABBR2, GRIA1, GRID2, GRIK4, GRIP1, GRM1, GRM7, GRM8, ITGA1, KALRN, KCND3, KCNIP1, KCNIP4, KCNJ6, KIRREL3, KLHL1, KLHL14, KLHL24, MYO10, MYO5A, MYO5B, NCAM2, NLGN1, NOV, NRCAM, NTM, ODZ4, PALMD, PCSK1, PEX5L, PRKCA, RAB27A, RAPGEF4, RELN, RPTOR, SCN11A, SEMA3A, SEPT11, SLC1A2, SYNPR, SYT5, TRIM9*
Synapse	4.6×10^{-5}	2.4	*APBB2, CADM2, CADPS, CAMK2D, CDH23, CPLX1, CYFIP2, DTNA, GRID2, GRM7, MAGI2, MYH9, MYRIP, NLGN1, NRCAM, NRXN1, NTM, NTRK2, PCDH15, PCLO, PHACTR1, RAPGEF4, RIMBP2, SEPT11, SERPINE2, SHISA9, SNTB1, UTRN*

GO categories in which false discovery rate is less than 0.01.

Table S27 continued.

Description	p-value	Enrichment	Genes
Integral to plasma membrane	5.4×10^{-5}	1.5	*ABCA1, ABCA4, ABCB11, ABCB5, ADAM29, ADRA1A, ALK, ANK2, ART3, ASIC2, BMPR1B, CALCR, CD200, CD81, CD84, CDH4, CHRM5, CLCA2, CLCNKB, CLEC1A, CNR2, CNTN2, COL25A1, CXADR, DDR2, DSCAM, EMR1, EPHA3, EPHA6, FCAR, FCER2, FLT3, FLVCR1, GABBR2, GABRB3, GABRR1, GLRA3, GP5, GPC5, GPC6, GPNMB, GPR137B, GPR39, GRID2, GRIK1, GRIK2, GRIK4, GRIN2A, GRM1, GRM5, GRM7, GRM8, HLA-A, HLA-B, HLA-C, HLA-DPA1, HLA-DQA1, HLA-DQA2, HLA-DRA, HLA-DRB1, IL12RB2, IL1RAP, IL1RL2, ITGB3, KCNK18, KCNMB3, KL, LCT, LGR5, MAEA, MSR1, MUC12, NLGN1, NOTCH2, NOTCH4, NPFFR2, NPR3, NRCAM, NRG1, NRG3, NRXN1, NRXN3, NTRK2, NTRK3, OPCML, OPN3, OPRK1, PCDHA1, PCDHA10, PCDHA11, PCDHA2, PCDHA3, PCDHA4, PCDHA5, PCDHA6, PCDHA7, PCDHA8, PCDHAC1, PLA2R1, PLSCR1, PRKD1, PTGER3, PTPRB, PTPRD, PTPRH, PTPRM, PTPRN2, RAMP3, ROR1, SCARB1, SCNN1G, SCTR, SLC12A3, SLC15A2, SLC17A5, SLC1A6, SLC2A9, SLC6A15, SLC7A1, SLCO1B3, TEK, TMPRSS9, TRHDE, TRPA1, TRPC4, TSHR, TSPAN9*
Lysosomal membrane	5.8×10^{-5}	5.6	*CUBN, HLA-DPA1, HLA-DPB1, HLA-DQA1, HLA-DQA2, HLA-DQB1, HLA-DRB1, HLA-DRB5, MARCH1, SLC17A5, TMEM106B*
Sheet-forming collagen	6.1×10^{-5}	18.5	*COL4A1, COL4A2, COL4A3, COL4A4*
Integrin complex	6.3×10^{-5}	4.7	*ITGA1, ITGA11, ITGA2, ITGA6, ITGA8, ITGA9, ITGAE, ITGB3, ITGB5, ITGBL1, MYH9*
Ion channel complex	6.4×10^{-5}	2.0	*ABCC8, AKAP6, ANO2, ANO3, ANO6, BEST3, CACNA1A, CACNA1C, CACNA2D1, CASQ2, CATSPERB, CHRNB3, CLCNKB, CNTN2, CNTNAP2, GABRB3, GABRG3, GABRR1, GLRA3, KCNA6, KCNB2, KCNC2, KCND3, KCNE1, KCNJ6, KCNK2, KCNMB2, KCNMB3, KCNQ5, KCNV2, PEX5L, RYR2, SCN11A, SCN1A, SCN3A, SCN3B, SCN9A, SCNN1G, TRPC4*
Sarcolemma	6.5×10^{-5}	8.1	*ADCY5, ANK3, FLOT1, KCND3, SGCG, SGCZ, SNTG2, [LAMA2*
Extracellular matrix part	8.3×10^{-5}	2.2	*AMTN, CCBE1, COL11A1, COL20A1, COL21A1, COL23A1, COL24A1, COL25A1, COL27A1, COL2A1, COL4A1, COL4A2, COL4A3, COL4A4, COL5A1, COLEC12, EMID1, EMID2, FBLN1, GLDN, LAMA2, LAMA4, LAMC1, LAMC2, LEPREL1, LOXL2, MSR1, NID2, NTN4, SMOC2, THBS2, TNR*
Vacuolar membrane	9.2×10^{-5}	4.8	*CUBN, HLA-DPA1, HLA-DPB1, HLA-DQA1, HLA-DQA2, HLA-DQB1, HLA-DRB1, HLA-DRB5, MARCH1, SLC17A5, TMEM106B*
Phagocytic vesicle membrane	9.5×10^{-5}	28.0	*DMBT1, HLA-A, HLA-B, HLA-C*
Vacuolar part	1.1×10^{-4}	3.3	*ARSB, ASAH1, CUBN, GALC, GPC5, GPC6, HLA-DPA1, HLA-DPB1, HLA-DQA1, HLA-DQA2, HLA-DQB1, HLA-DRB1, HLA-DRB5, MARCH1, OCA2, SLC17A5, TMEM106B*
Intrinsic to plasma membrane	1.2×10^{-4}	1.4	*ABCA1, ABCA4, ABCB11, ABCB5, ADAM29, ADRA1A, ALK, ANK2, ART3, ASIC2, BMPR1B, CALCR, CD200, CD81, CD84, CDH4, CHRM5, CLCA2, CLCNKB, CLEC1A, CNR2, CNTN2, COL25A1, CXADR, DDR2, DSCAM, EMR1, EPHA3, EPHA6, FCAR, FCER2, FLT3, FLVCR1, GABBR2, GABRB3, GABRR1, GGT1, GLRA3, GP5, GPC5, GPC6, GPNMB, GPR137B, GPR39, GRID2, GRIK1, GRIK2, GRIK4, GRIN2A, GRM1, GRM5, GRM7, GRM8, HLA-A, HLA-B, HLA-C, HLA-DPA1, HLA-DQA1, HLA-DQA2, HLA-DRA, HLA-DRB1, IL12RB2, IL1RAP, IL1RL2, ITGB3, KCNK18, KCNMB3, KL, LCT, LGR5, MAEA, MSR1, MUC12, NLGN1, NOTCH2, NOTCH4, NPFFR2, NPR3, NRCAM, NRG1, NRG3, NRXN1, NRXN3, NTRK2, NTRK3, OPCML, OPN3, OPRK1, PCDHA1, PCDHA10, PCDHA11, PCDHA2, PCDHA3, PCDHA4, PCDHA5, PCDHA6, PCDHA7, PCDHA8, PCDHAC1, PKHD1, PLA2R1, PLSCR1, PRKD1, PTGER3, PTPRB, PTPRD, PTPRH, PTPRM, PTPRN2, RAMP3, ROR1, SCARB1, SCNN1G, SCTR, SLC12A3, SLC15A2, SLC17A5, SLC1A6, SLC2A9, SLC6A15, SLC7A1, SLCO1B3, SPTB, TEK, TMPRSS9, TRHDE, TRPA1, TRPC4, TSHR, TSPAN9*
Cytoskeleton	1.4×10^{-4}	1.6	*ABLIM1, ADH4, AFAP1, AMPH, ANK2, ANK3, APBB1IP, ARHGAP24, ARPC5, BICD1, BIN3, CAP1, CDSN, CORO2B, COTL1, CTNNA2, CTNNA3, DENND2A, DNAH9, EPB41L4A, ERC2, FAM65B, FGD5, FLNB, FRMD4A, FRMD4B, FRMPD1, GYS2, IFT43, INPP5D, IQGAP2, KALRN, KLHL1, KLHL5, LDB3, LRPPRC, LRRFIP1, MAEA, MLPH, MTSS1, MYH9, MYO9B, MYPN, MYRIP, NPFFR2, PARVA, PCLO, PDLIM1, PHLDB2, PKNOX2, PLEKHH2, PPP2CA, PRKCE, PSTPIP2, PTPN14, PTPN21, SCLT1, SGCG, SGCZ, SHROOM3, SKA1, SNTB1, SNTG1, SNTG2, SORBS2, SPRR1B, SPTB, STK39, SVIL, SYNE1, TBCA, TNS1, TRIM9, TRIP11, TRPC4, TUBB, UTRN, VCL*
Endoplasmic reticulum part	1.4×10^{-4}	2.6	*ABCD4, ALG8, ATP10D, CASQ2, COL24A1, COL25A1, CYP4Z1, EMID2, ERO1LB, HLA-A, HLA-B, HLA-C, HLA-DPA1, HLA-DPB1, HLA-DQA1, HLA-DQA2, HLA-DQB1, HLA-DRB1, HLA-DRB5, HMGCLL1, KLHL14, MARCH1, RYR2, SPINK5, TAP2*
Presynaptic membrane	1.5×10^{-4}	3.1	*CADPS2, ERC2, GRIK1, GRIK2, GRIK4, GRIN2A, GRM1, GRM7, GRM8, NRXN1, PDE2A, RIMS1, RIMS2, SLC1A2, SYT1, UNC13A, UNC13C*
Cilium	1.7×10^{-4}	3.8	*ADCY5, CDH23, CNGA3, DYNC2H1, FOPNL, IFT57, MYRIP, PACRG, PCDH15, PKHD1, SLC9C1, SORD, TTLL11*
Cell body	1.8×10^{-4}	1.9	*ALS2, APOA1BP, BMPR1B, CACNA1C, CNTN2, CNTNAP2, CPLX1, CXADR, DDC, DFNB31, DISC1, DLG2, DNER, DPYSL2, DPYSL3, GPM6A, GRIA1, GRIK1, GRM8, KALRN, KCND3, KCNIP1, KCNIP4, KLHL1, KLHL14, MAP2, MBP, MYO10, MYO5A, MYO5B, NOV, NTRK2, PACRG, PCSK1, PRKCA, RBFOX3, RPTOR, SCN1A, SERPINE2, SRD5A1, SYT5, TANC1, TUBB*
Cation channel complex	2.1×10^{-4}	2.3	*ABCC8, AKAP6, CACNA1A, CACNA1C, CACNA2D1, CASQ2, CATSPERB, CNTN2, CNTNAP2, KCNA6, KCNB2, KCNC2, KCND3, KCNE1, KCNJ6, KCNK2, KCNMB2, KCNMB3, KCNQ5, KCNV2, PEX5L, RYR2, SCN11A, SCN1A, SCN3A, SCN3B, SCN9A, SCNN1G, TRPC4*
Postsynaptic membrane	2.1×10^{-4}	2.2	*ANK2, ANK3, ANKS1B, CHRM5, CHRNB3, CLSTN2, CNIH3, DISC1, DLG2, DLGAP1, GABBR2, GABRB3, GABRG3, GABRR1, GLRA3, GRIA1, GRID1, GRID2, GRIK1, GRIK2, GRIK4, GRIN2A, GRIP1, GRM7, LRRTM4, NLGN1, PSD3, SYNE1, TANC1, UTRN*
Endoplasmic reticulum lumen	2.2×10^{-4}	2.1	*ARSB, ARSG, ARSJ, CASQ2, COL11A1, COL20A1, COL21A1, COL23A1, COL24A1, COL25A1, COL27A1, COL2A1, COL4A1, COL4A2, COL4A3, COL4A4, COL5A1, EMID2, ERAP1, ERAP2, FLT3, GLT25D2, H6PD, LEPREL1, OLFM1, P4HA1, PDGFD, PDIA6, RCN3, SLC27A2*

GO categories in which false discovery rate is less than 0.01.

Table S27 continued.

Description	p-value	Enrichment	Genes
Flagellum part	2.5×10^{-4}	12.3	*PACRG*, *SLC9C1*, *SPAG16*, *TEKT4*, *TEKT5*
Microtubule-based flagellum part	2.5×10^{-4}	12.3	*PACRG*, *SLC9C1*, *SPAG16*, *TEKT4*, *TEKT5*
Extracellular matrix	2.5×10^{-4}	1.7	*ABI3BP*, *ADAMTS12*, *ADAMTS16*, *ADAMTS18*, *ADAMTS2*, *ADAMTSL2*, *ADAMTSL3*, *AMBN*, *AMTN*, *CDON*, *COL20A1*, *COL21A1*, *COL4A1*, *COL4A2*, *COL5A1*, *CRTAC1*, *DMBT1*, *DPT*, *FBLN1*, *FGF1*, *FRAS1*, *GPC5*, *GPC6*, *GPLD1*, *HMCN1*, *HPSE2*, *IGFBP7*, *LAMA1*, *LAMA2*, *LAMA4*, *LAMC1*, *LMCD1*, *LTBP1*, *MAMDC2*, *MATN1*, *MEPE*, *MMP20*, *NAV2*, *NID2*, *NOV*, *PLSCR1*, *PXDN*, *RELN*, *SERPINE2*, *SMOC2*, *SPARCL1*, *SPON1*, *TNN*, *TNR*, *VIT*, *VWF*, *WNT3*

GO categories in which false discovery rate is less than 0.01.
